# Human Cytomegalovirus Genomes Sequenced Directly From Clinical Material: Variation, Multiple-Strain Infection, Recombination, and Gene Loss

**DOI:** 10.1093/infdis/jiz208

**Published:** 2019-05-02

**Authors:** Nicolás M Suárez, Gavin S Wilkie, Elias Hage, Salvatore Camiolo, Marylouisa Holton, Joseph Hughes, Maha Maabar, Sreenu B Vattipally, Akshay Dhingra, Ursula A Gompels, Gavin W G Wilkinson, Fausto Baldanti, Milena Furione, Daniele Lilleri, Alessia Arossa, Tina Ganzenmueller, Giuseppe Gerna, Petr Hubáček, Thomas F Schulz, Dana Wolf, Maurizio Zavattoni, Andrew J Davison

**Affiliations:** 1 Medical Research Council–University of Glasgow Centre for Virus Research, United Kingdom; 2 Institute of Virology, Hannover Medical School, United Kingdom; 3 German Center for Infection Research, Hannover-Braunschweig site, United Kingdom; 4 Pathogen Molecular Biology Department, London School of Hygiene and Tropical Medicine, United Kingdom; 5 Division of Infection and Immunity, School of Medicine, Cardiff University, United Kingdom; 6 Molecular Virology Unit, Microbiology and Virology Department, Fondazione Istituto di Ricovero e Cura a Carattere Scientifico (IRCCS) Policlinico San Matteo, Italy; 7 Department of Clinical, Surgical, Diagnostic and Pediatric Sciences, University of Pavia, Italy; 8 Laboratory of Genetics-Transplantology and Cardiovascular Diseases, Italy; 9 Departments of Obstetrics and Gynecology, Fondazione IRCCS Policlinico San Matteo, Pavia, Italy; 10 Institute for Medical Virology and Epidemiology of Viral Diseases, University Hospital Tuebingen, Germany; 11 Department of Medical Microbiology, Motol University Hospital, Prague, Czech Republic, Israel; 12 Clinical Virology Unit, Department of Clinical Microbiology and Infectious Diseases, Hadassah University Hospital, Jerusalem, Israel

**Keywords:** human cytomegalovirus, genome sequence, target enrichment, genotype, variation, multiple-strain infection, recombination, gene loss, mutation

## Abstract

The genomic characteristics of human cytomegalovirus (HCMV) strains sequenced directly from clinical pathology samples were investigated, focusing on variation, multiple-strain infection, recombination, and gene loss. A total of 207 datasets generated in this and previous studies using target enrichment and high-throughput sequencing were analyzed, in the process enabling the determination of genome sequences for 91 strains. Key findings were that (*i*) it is important to monitor the quality of sequencing libraries in investigating variation; (*ii*) many recombinant strains have been transmitted during HCMV evolution, and some have apparently survived for thousands of years without further recombination; (iii) mutants with nonfunctional genes (pseudogenes) have been circulating and recombining for long periods and can cause congenital infection and resulting clinical sequelae; and (*iv*) intrahost variation in single-strain infections is much less than that in multiple-strain infections. Future population-based studies are likely to continue illuminating the evolution, epidemiology, and pathogenesis of HCMV.

## Teaser

The genomic characteristics of human cytomegalovirus strains sequenced directly from clinical samples were investigated using 207 datasets generated in this and previously published studies by target enrichment and high-throughput sequencing, focusing on variation, multiple-strain infection, recombination, and gene loss.

**(See the major Article by Suárez et al, on pages 792–801.)**

Human cytomegalovirus (HCMV) poses a risk, particularly to people with immature or compromised immune systems, and can have serious outcomes in congenitally infected children, transplant recipients, and people with human immunodeficiency virus/AIDS. Prior to the advent of high-throughput technologies, studies of HCMV genomes in natural infections were limited to Sanger sequencing of polymerase chain reaction (PCR) amplicons, often focusing on a small number of polymorphic (hypervariable) genes [1]. This left out most of the genome and also restricted the characterization of multiple-strain infections, which may have more serious outcomes.

The first complete HCMV genome sequence to be determined was that of the high-passage strain AD169 [2], from a plasmid library. Over a decade later, additional genomes were sequenced from bacterial artificial chromosomes [3–5], virion DNA [6] and overlapping PCR amplicons [7, 8]. These sequences were also determined using Sanger technology, and were complemented subsequently by many others, increasingly using high-throughput methods [7, 9–13]. With only 3 exceptions [7, 11], all were derived from laboratory strains isolated in cell culture. Mounting evidence of the existence of multiple-strain infections and the propensity of HCMV to mutate during cell culture [6–8, 14, 15] added impetus to sequencing genomes directly from clinical material to define natural populations. One strategy for this involves sequencing overlapping PCR amplicons [7, 16]. Another utilizes an oligonucleotide bait library representing known HCMV diversity to select target sequences from random DNA fragments. This target enrichment technology originated in commercial kits for cellular exome sequencing, and was subsequently applied to various pathogens [17, 18], including HCMV [19–21]. We have applied it to HCMV since 2012 and have systematically released via GenBank many genome sequences that have proved pivotal in other studies [11, 12, 19–21].

The HCMV genome exhibits several evolutionary phenomena, including variation, multiple-strain infection, recombination, and gene loss, all of which were discovered prior to high-throughput sequencing and have since been illuminated by this technology (early references are [22–26]). We explore these and other key genomic features of HCMV, with an emphasis on the strains present in clinical material.

## METHODS

### Samples

For convenience, samples were analyzed as collections 1–3, which are summarized in Table 1 and described in Supplementary Tables 1–3, respectively. Collection 3 represents samples sequenced by others in previous studies using target enrichment with a different oligonucleotide bait library. The features of the samples are shown in Supplementary Tables 1–3 (rows 3–6), and the clinical outcomes of congenital infection are in Supplementary Table 1 (row 205).

**Table 1.** Selected Characteristics on Sample Collections 1–3

Characteristic	Collection 1	Collection 2	Collection 3
Patients, No.^a^	48	29	25
Patient condition	Congenital infection	Mostly transplant recipients	Various
Samples, No.	53	89	57
Sample source, city (prefix)	Pavia (PAV), Jerusalem (JER), Prague (PRA)	Hannover (Child, RTR, SCTR), Pavia (PAV)	Rotterdam (Rot), London (Lon, Pat_)
Datasets, No.	53	97^b^	57^c^
Duplicated libraries, No.	0	7	0
HCMV load, IU/µL^d^	26–559 968	5–194 840	104–18 377
Genome copies for library, No.^e^	225–8 399 520	280–3 896 800	Unknown
Reads in Merlin alignment, %	2–91	0–85	0–90
Coverage ratio in Merlin alignment, % unique/total reads	0.40–83.12	0.00–76.09	0.00–90.21
Genome sequences determined, No.^f^	42	25	24

Details are provided in Supplementary Tables 1–3.

Abbreviation: HCMV, human cytomegalovirus.

^a^Archived diagnostic samples were used, and clinical data were retrieved, with the approval of the institutional review boards of Policlinico San Matteo, Pavia (reference numbers 35853/2010 and 35854/2010), Hadassah University Hospital, Jerusalem (reference number HMO-063911), Motol University Hospital, Prague (reference number EK-701a/16) and Hannover Medical School, Hannover (reference number 2527-2014).

^b^We reported 68 of the Hannover datasets previously [21].

^c^These datasets were reported previously by others, and were either provided by the authors [19] or downloaded from the European Nucleotide Archive (study PRJEB12814) [20].

^d^Viral load in most extracted samples was quantified in the laboratory of origin or the sequencing laboratory. In some instances, the entire sample was used blind to generate a sequencing library.

^e^Assumes that 1 IU is equivalent to 1 genome copy.

**Figure 1.**
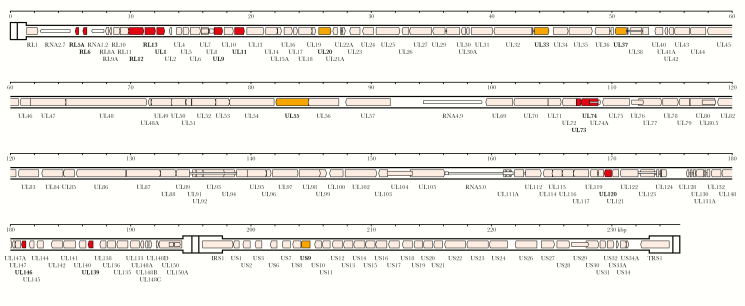
Locations in the human cytomegalovirus strain Merlin genome of genes used for genotyping. The genome consists of 2 unique regions, U_L_ (1325–194 343 bp) and U_S_ (197 627–233 108 bp), the former flanked by inverted repeats TR_L_ (1–1324 bp) and IR_L_ (194 344–195 667 bp), and the latter flanked by inverted repeats IR_S_ (195 090–197 626 bp) and TR_S_ (233 109–235 646 bp). Protein-coding regions are indicated by shaded arrows, and noncoding RNAs as narrower, white arrows, with gene nomenclature below. Introns are shown as narrow white bars. The 12 genes (RL5A, RL6, RL12, RL13, UL1, UL9, UL11, UL73, UL74, UL120, UL146, and UL139) used for motif read-matching are in dark gray (red in online version). Two of these genes (RL13 and UL146) were also used for genotype read-matching. The additional 5 genes (UL20, UL33, UL37, UL55, and US9) used to genotype sequences by alignment are medium gray (orange in online version). All other genes are shown in white (pink in online version).

^f^The trimmed paired-read data were aligned to the UCSC hg19 human reference genome (http://genome.ucsc.edu/) using Bowtie2. Nonmatching reads were assembled de novo into contigs using SPAdes version 3.5.0 [27]. The contigs were ordered using Scaffold_builder version 2.2 [28] by reference to a version of the strain Merlin sequence lacking all but 100 nt of the terminal repeat regions (TR_L_ at the left end and TR_S_ at the right end; Figure 1), and merged into a draft genome sequence. Residual gaps were filled by identifying relevant reads anchored in flanking regions and assembling them manually in a reiterative fashion. TR_L_ and TR_S_ were reinstated, and the complete genome sequence was verified by aligning it against the read data using Bowtie2 and inspecting the alignment in Tablet. An annotated genome sequence was produced using Sequin (https://www.ncbi.nlm.nih.gov/Sequin/).

### DNA Sequencing

Target enrichment and sequencing library preparation were performed using the SureSelect XT version 1.7 system for Illumina paired-end libraries with biotinylated RNA bait libraries (Agilent) [21]. Bait libraries representing known HCMV diversity were designed in February 2012 and April 2014 from 31 and 64 complete genome sequences, respectively. Information on and access to the latter library (55 210 baits of 120 nucleotides [nt] with overrepresentation of G + C–rich regions) are available from the corresponding author. Data on viral loads and library construction are shown in Supplementary Tables 1–3 (rows 9–12). Datasets of 300 or 150 nt paired-end reads were generated using a MiSeq (Illumina). Their names are shown in Supplementary Tables 1–3 (row 7). They were prepared for analysis using Trim Galore version 0.4.0 (program available at http://www.bioinformatics.babraham.ac.uk/projects/trim_galore/; length = 21, quality = 10, and stringency = 3). The numbers of trimmed reads are in Supplementary Tables 1–3 (row 15).

### Library Diversity

Estimating the number of reads in a dataset derived from unique HCMV fragments initially involved using Bowtie2 version 2.2.6 [29] to align the reads against the strain Merlin sequence (GenBank accession number AY446894.2), and, where it could be determined, the consensus genome sequence derived from the dataset. The relevant data are in Supplementary Tables 1–3 (rows 17–19 and 23–26). Reads containing insertions or deletions were removed to preserve coordinate numbering, as were duplicate read pairs sharing both end coordinates and duplicate unpaired reads sharing one end coordinate, thereby producing an alignment file for unique reads derived from unique HCMV fragments (program available at https://centre-for-virus-research.github.io/VATK/AssemblyPostProcessing). This file was viewed using Tablet version 1.14.11.7 [30]. The coverage depth values for total and unique fragment reads are in Supplementary Tables 1–3 (rows 20–21 and 27–28).

### Strain Enumeration

The number of strains represented in a dataset was estimated by 2 strategies: genotype read-matching and motif read-matching (program available at https://centre-for-virus-research.github.io/VATK/HCMV_pipeline). Both strategies utilized datasets concatenated from the paired-end datasets. The genotype designations used were either based on reported phylogenies [6, 12, 25, 31, 32], amended or extended as appropriate, or constructed afresh using Clustal Omega version 1.2.4 [33] and MEGA version 6.0.6 [34] with data for the genomes listed in Supplementary Table 4 and individual genes for which additional sequences were available in GenBank. Alignments and phylogenetic reconstructions are in Supplementary Figures 1 and 2, respectively.

For genotype read-matching, Bowtie2 was used to align the reads to sequences representing the genotypes of 2 hypervariable genes, UL146 and RL13 [6, 12, 35]. The sequences from the entire coding region of UL146 and the central coding region of RL13 are in Supplementary Tables 1–3 (rows 34–58). In contrast to the UL146 genotypes, the RL13 genotypes cross-matched within 4 groups (G1, G2, G3; G4A, G4B; G6, G10; and G7, G8). In these instances, the genotype within the group with most matching reads was scored. The number of reads aligned to each genotype is in Supplementary Tables 1–3 (rows 34–58). A genotype was scored if the number of reads was >10 and represented >2% of the total number detected for all genotypes of that gene. For 14 samples in collection 1 that had been sequenced prior to the availability of ultrapure (TruGrade) oligonucleotides, these values were >25 and >5%, respectively. The number of strains in a sample was scored as the greater of the numbers of genotypes detected for the 2 target genes, and is in Supplementary Tables 1–3 (row 13).

For motif read-matching, conserved genotype-specific motifs (20–31 nt) were identified by visual inspection of alignments (Supplementary Figure 1) for 12 hypervariable genes [6, 12, 19, 35]. Additional motifs for identifying common intergenotypic recombinants were included. The motif sequences and number of reads containing perfect matches to a sequence or its reverse complement are in Supplementary Tables 1–3 (rows 60–170). Genotypes were scored as described above. The number of strains in a sample was estimated as the maximum number of genotypes detected for at least 2 genes, and is in Supplementary Tables 1–3 (row 14).

### Pseudogene Analysis

The genomes of some HCMV strains exhibit gene loss apparent as pseudogenes resulting from mutations causing premature translational termination [7, 11, 12, 26]. These mutations are substitutions that introduce in-frame stop codons or ablate splice sites, or insertions or deletions that cause frameshifting or loss of protein-coding regions. Motif read-matching was used to assess the presence of common mutations and also to determine the prevalence of mutations identified in collection 1. These data are in Supplementary Tables 1–3 (rows 171–178) and Supplementary Table 1 (rows 180–203), respectively.

### Intrahost Variation

Minor genome populations were analyzed by enumerating single-nucleotide polymorphisms (SNPs) in datasets for which consensus genome sequences had been determined. Thus, the term mutant applies hereafter to a strain that has a mutation in the consensus sequence resulting in a pseudogene, and the term SNP applies to a minor variation from the consensus within a population. To enumerate SNPs, original datasets were prepared for analysis using Trim Galore (length = 100, quality = 30, and stringency = 1), and trimmed reads were mapped using Bowtie2. Alignment files in SAM format were converted into BAM format, sorted using SAMtools version 1.3 [36], and analyzed using LoFreq version 2.1.2 [37] and V-Phaser 2 [38].

### Data Deposition

Original datasets were purged of human reads and deposited in the European Nucleotide Archive (ENA; project number PRJEB29585), and consensus genome sequences were deposited in GenBank. The accession numbers are in Supplementary Tables 1–3 (rows 8 and 29, respectively). Updated genome sequence determinations in collection 3 were deposited by the original submitters in GenBank [19] or by us as third-party annotations in ENA (project number PRJEB29374) [20]. Sequence features are in Supplementary Tables 1–3 (rows 30–32).

## RESULTS

### Operational Limitations

A total of 207 datasets from 199 samples and 102 individuals were analyzed (Table 1 and Supplementary Tables 1–3). Library quality was represented in the percentage of HCMV reads and the coverage depth by unique fragment reads. These values were related to sample type, being higher for urine than blood presumably because of a higher proportion of viral to host DNA. They also depended on the number of viral genome copies used to make the library, with >1000 copies generally being needed to determine a complete genome sequence. However, despite high library diversity, it was not possible to assemble complete genome sequences from most datasets in collection 3 because of gaps in RL12 and some G + C–rich regions, perhaps as a result of limitations in the bait library. The use of excessive PCR cycles with some samples in collections 1 and 2 led to high coverage depth by total fragment reads but low coverage depth by unique fragment reads, and thus to highly clonal libraries (eg, PAV2 in collection 1). Genotypes present at subthreshold levels may represent multiple-strain infections or cross-contamination during the complex sample processing pathway (eg, PRA4 reads in PRA6A in collection 1).

### Genome Sequences

A total of 91 complete or almost complete HCMV genome sequences were determined (Table 1). We reported 5 previously [21], and 16 are improvements on published sequences [19]. Most originated from single-strain infections or multiple-strain infections in which one strain was predominant, and some originated from different strains that predominated in a patient at different times. Defining a strain as a viral genome present in an individual, these 91 sequences, plus an additional 49 deposited by our group and 104 by others, brought the number of strains sequenced to 244 (Supplementary Table 4). Of these, 91 were sequenced directly from clinical material, and all but one were determined in this and our previous study [21]. The average size of the HCMV genome, based on the 78 complete sequences in this set, is 235 465 bp (range 234 316–237 120 bp).

### Multiple-Strain Infections

Genotypic differences in hypervariable genes (Figure 1 and Supplementary Figures 1 and 2) were exploited to distinguish single-strain from multiple-strain infections by genotype read-matching and motif read-matching with threshold values. To our knowledge, these methods, employed in the present work and the companion study [39], have not been used previously for categorizing HCMV infections. Single strains were common in congenitally infected patients (n = 43/50 in collections 1 and 2), but significantly less so in transplant recipients (n = 11/25 in collections 2 and 3; χ^2^ = 14.583, *P* < .05). Intrahost variation is discussed below.

### Recombination

The 244 genome sequences were genotyped in the 12 hypervariable genes used for motif read-matching and then in 5 additional genes (Figure 1 and Supplementary Table 4).

Hypervariation in UL55, which encodes glycoprotein B (gB), is located in 2 regions (UL55N near the N terminus, and UL55X encompassing the proteolytic cleavage site) [23, 40]. Five genotypes (G1–G5) have been assigned to each region [23, 40–42], which are separated by 927 bp that are 80% identical in all strains. All genomes had a recognized UL55X genotype (Supplementary Table 5). As reported previously [40], UL55N G2 and G3 could not be distinguished reliably from each other, and 2 additional genotypes (G6–G7) were detected that may have arisen from ancient recombination events within UL55N (Supplementary Tables 4 and 5 and Supplementary Figure 1). There was evidence for recombination in the region between UL55N and UL55X in only 8 genomes. This low proportion of recombination (3.3%) contrasts with the higher levels proposed in UL55 from PCR-based studies [40, 43], which may have been affected by artefactual recombination.

UL73 and UL74, which encode glycoproteins N and O (gN and gO), respectively, are adjacent hypervariable genes that exist as 8 genotypes each [25, 32, 44]. There was evidence for recombination between them in only 7 genomes (2.9%), in accordance with the low levels (2.2%) detected previously in PCR-based studies [25, 32, 45]. In the region containing adjacent hypervariable genes RL12, RL13, and UL1, recombinants were also rare (1.2%) within RL12 and absent from RL13 and UL1. In contrast, hypervariable genes UL146 and UL139, which encode a CXC chemokine and a membrane glycoprotein, respectively, are separated by a well-conserved region of over 5 kbp. The number (66) of the 126 possible genotype combinations represented in the 244 genomes is too large to allow any underlying genotypic linkage to be discerned, consistent with previous conclusions from PCR-based studies [31]. No recombinants were noted within UL146.

In principle, strains in multiple-strain infections have the opportunity to recombine. In our previous analysis of RTR1 in collection 2, we noted that one strain (RTR1A) predominated at earlier times and another (RTR1B) at later times [21]. From the low frequency of SNPs across a large part of the genome, we concluded that the second strain had arisen either by recombination involving the first strain or by reinfection with, or reactivation of, a second strain fortuitously similar to the first. In the present study, recombination was strongly supported by a comparison of the 2 genome sequences, which showed that approximately two-thirds of the genome is almost identical (differing by 3 substitutions in noncoding regions), whereas the remaining third is highly dissimilar.

To investigate whether strains have been transmitted without recombination occurring, identical genotypic constellations were identified among the 244 genomes (Table 2). This revealed the existence of 12 haplotype groups within which multiple strains lack signs of having recombined since diverging from their last common ancestor; these are henceforth termed nonrecombinant strains. As an incidental outcome, the 2 strains in group 1 (PRA8 and CZ/3/2012), which were characterized in different studies, were confirmed as having originated from the same patient, reducing the set of sequenced strains to 243. The results from the other 11 groups suggest that nonrecombinant strains have been circulating, some for periods sufficient to allow the accumulation of >100 substitutions. Among the highly divergent groups, group 9 (3 strains) exhibited 135 differences, with the 50 that would affect protein coding distributed among 38 genes, and group 10 (2 strains) exhibited 138 differences, with the 38 that would affect protein coding distributed among 27 genes. No obvious bias was observed toward greater diversity in any particular gene or group of genes, including those in the hypervariable category.

**Table 2.** Groups of Nonrecombinant Strains

		Genotypes^a^																			
Group	Strain	RL5A	RL6	RL12	RL13	UL1	UL9	UL11	UL20	UL33	UL37	UL55N	UL73	UL74	UL120	UL146	UL139	US9	Mutated Genes	Differences^b^	Shared Mutations
1	PRA8	1	1	6	6	6	6	2	5	1	5	2/3	4C	1C	2B	1	4	1	UL145	0	These strains share a UL145 mutation, were characterized in different studies, and were confirmed as having been derived from the same patient
	CZ/3/2012	1	1	6	6	6	6	2	5	1	5	2/3	4C	1C	2B	1	4	1	UL145		
2	BE/3/2011	2	4	1B	1	1	4	1	6	2	2	2/3	4A	3	1A	8	2	1	None	1	None
	BE/21/2011	2	4	1B	1	1	4	1	6	2	2	2/3	4A	3	1A	8	2	1	None		
3	UK/Lon6/Urine/2011	5	1	7	7	7	1	1	6	2	1	4	3A	1B	3B	13	1A	1	None	23	None
	2CEN15	5	1	7	7	7	1	1	6	2	1	4	3A	1B	3B	13	1A	1	None		
																					
	BE/5/2012	5	1	7	7	7	1	1	6	2	1	4	3A	1B	3B	13	1A	1	None		
4	BE/14/2012	3	5	4A	4A	4	6	6	5	4	5	4	3A	1B	2A	9	5	1	RL6 UL9 UL40 US7	26	These strains share a UL9 mutation and also RL6 and UL40 mutations that are present in other strains
	BE/36/2011	3	5	4A	4A	4	6	6	5	4	5	4	3A	1B	2A	9	5	1	RL6 UL9 UL40		
5	BE/10/2012	6	3	1A	1	1	1	1	2	4	5	4	3A	1B	2A	3	7	1	None	35	None
	BE/26/2011	6	3	1A	1	1	1	1	2	4	5	4	3A	1B	2A	3	7	1	None		
6	BE/1/2011	1	1	4A	4A	4	6	6	7	4	6	2/3	4A	3	2A	1	4	1	UL1 UL9	65	These strains bear a UL9 mutation that is present in other strains, and 2 strains share a UL1 mutation
	BE/8/2010	1	1	4A	4A	4	6	6	7	4	6	2/3	4A	3	2A	1	4	1	UL9		
	BE/9/2012	1	1	4A	4A	4	6	6	7	4	6	2/3	4A	3	2A	1	4	1	UL1 UL9		
7	NAN1LA	3	5	5	5	5	7	3	2	2	6	2/3	4D	5	3B	7	5	2	RL6 US9	73	These strains share RL6 and US9 mutations that are present in other strains
	BE/6/2012	3	5	5	5	5	7	3	2	2	6	2/3	4D	5	3B	7	5	2	RL6 US9 US27		
8	BE/7/2012	2	4	1A	1	1	4	1	7	5	3	2/3	4A	3	2B	13	5	1	RL5A RL13 UL150	125	These strains share a UL150 mutation that is present in other strains
	BE/11/2012	2	4	1A	1	1	4	1	7	5	3	2/3	4A	3	2B	13	5	1	UL150		
	BE/16/2012	2	4	1A	1	1	4	1	7	5	3	2/3	4A	3	2B	13	5	1	UL150		
	BE/26/2010	2	4	1A	1	1	4	1	7	5	3	2/3	4A	3	2B	13	5	1	UL150		
	BE/30/2011	2	4	1A	1	1	4	1	7	5	3	2/3	4A	3	2B	13	5	1	UL150		
9	JER851	1	1	3	3	3	2	1	3	4	6	1	2	2B	3B	7	4	1	UL1 UL9 UL111A	135	These strains share a UL111A mutation that is present in another strain
	JER4041	1	1	3	3	3	2	1	3	4	6	1	2	2B	3B	7	4	1	UL111A		
	BE/25/2010	1	1	3	3	3	2	1	3	4	6	1	2	2B	3B	7	4	1	UL111A		
10	JER5695	1	1	7	7	7	1	1	6	2	1	2/3	3B	2A	4B	13	2	1	UL9 UL111A	138	These strains share a UL111A mutation that is present in other strains, and have different UL9 mutations
	BE/15/2010	1	1	7	7	7	1	1	6	2	1	2/3	3B	2A	4B	13	2	1	RL1 UL9 UL111A		
11	PRA7	1	1	4B	4B	4	9	6	5	5	6	6	4D	5	4B	10	2	1	RL5A UL111A	143	These strains share RL5A and UL111A mutations that are present in other strains
	JP	1	1	4B	4B	4	9	6	5	5	6	6	4D	5	4B	10	2	1	RL5A UL111A		
	BE/4/2010	1	1	4B	4B	4	9	6	5	5	6	6	4D	5	4B	10	2	1	RL5A UL111A		
12	BE/6/2011	5	1	4B	4B	4	9	6	1	5	3	2/3	3A	1B	1B	9	5	1	UL9	155	Two strains share a UL9 mutation that is present in other strains
	BE/18/2011	5	1	4B	4B	4	9	6	1	5	3	2/3	3A	1B	1B	9	5	1	None		
	BE/27/2011	5	1	4B	4B	4	9	6	1	5	3	2/3	3A	1B	1B	9	5	1	UL9		

^a^See Supplementary Figures 1 and 2 for genotype definitions. G prefix omitted.

^b^Total number of differences among all strains in the group, not including size variations in tandem repeats. To exclude repeat regions, sequences were aligned from the TATA box of RL1 to the end of U_S_, omitting the region from the AATAAA polyadenylation signal of UL150A to the beginning of TR_S_.

### Pseudogenes

Among the strains sequenced from clinical material, 77% are mutated in at least one gene (compared with 79% among all sequenced strains), and one is mutated in as many as 6 genes (Pat_D in collection 3) (Supplementary Table 4). The most frequently mutated genes are UL9, RL5A, UL1 and RL6 (members of the RL11 family), US7 and US9 (members of the US6 gene family), and UL111A (encoding viral interleukin 10) (Table 3). In addition, there was evidence from the PAV6 datasets (collection 1) for maternal transmission of a US7 mutant (Supplementary Table 1), and from PCR data (not shown) for maternal transmission of a UL111A mutant to PAV16 (collection 1). Focusing on the most common mutations, strains in which UL9, RL5A, UL1, US9, US7, and UL111A were affected (singly or in combination) were, like strains that were not mutated in any gene, transmitted in congenital infections and, in some cases, linked to defects in neurological development (Supplementary Table 1).

**Table 3.** Mutated Genes in Order of Decreasing Frequency

Gene	Feature(s)	Strains Mutated, No.^a^			Strains Mutated, %^a^		
		Passaged^b^	Clinical^c^	All^d^	Passaged^b^	Clinical^c^	All^d^
UL9	RL11 family; type 1 membrane protein	50	31	81	32.89	34.07	33.33
RL5A	RL11 family	31	27	58	20.39	29.67	23.87
UL1	RL11 family; type 1 membrane protein	20	18	38	13.16	19.78	15.64
RL6	RL11 family	23	14	37	15.13	15.38	15.23
US9	US6 family; type 1 membrane protein	26	11	37	17.11	12.09	15.23
UL111A	Viral interleukin-10	16	7	23	10.53	7.69	9.47
UL150	Unknown	11	3	14	7.24	3.30	5.76
US7	US6 family; type 1 membrane protein	7	7	14	4.61	7.69	5.76
UL40	Type 1 membrane protein	8	2	10	5.26	2.20	4.12
UL30	UL30 family	2	3	5	1.32	3.30	2.06
UL142	MHC family; type 1 membrane protein	2	3	5	1.32	3.30	2.06
RL12	RL11 family; type 1 membrane protein	3	1	4	1.97	1.10	1.65
RL1	RL1 family	1	2	3	0.66	2.20	1.23
UL136	Potential transmembrane domain	3	0	3	1.97	0.00	1.23
US13	US12 family; type 3 membrane protein	3	0	3	1.97	0.00	1.23
UL133	Potential transmembrane domain	2	0	2	1.32	0.00	0.82
US6	US6 family; type 1 membrane protein	1	1	2	0.66	1.10	0.82
US8	US6 family; type 1 membrane protein	0	2	2	0.00	2.20	0.82
US27	GPCR family; type 3 membrane protein	2	0	2	1.32	0.00	0.82
UL11	RL11 family; type 1 membrane protein	1	0	1	0.66	0.00	0.41
UL13	Unknown	0	1	1	0.00	1.10	0.41
UL14	UL14 family; type 1 membrane protein	0	1	1	0.00	1.10	0.41
UL15A	Potential transmembrane domain	0	1	1	0.00	1.10	0.41
UL20	Type 1 membrane protein	1	0	1	0.66	0.00	0.41
UL43	US22 family	0	1	1	0.00	1.10	0.41
UL99	Envelope-associated protein	1	0	1	0.66	0.00	0.41
UL148	Type 1 membrane protein	1	0	1	0.66	0.00	0.41
UL147	CXCL family	1	0	1	0.66	0.00	0.41
UL145	Unknown	0	1	1	0.00	1.10	0.41
UL150A	Unknown	1	0	1	0.66	0.00	0.41
IRS1	US22 family	1	0	1	0.66	0.00	0.41
US1	US1 family	1	0	1	0.66	0.00	0.41
US12	US12 family; type 3 membrane protein	1	0	1	0.66	0.00	0.41
US19	US12 family; type 3 membrane protein	0	1	1	0.00	1.10	0.41

Abbreviations: CXCL, chemokine (CXC motif) ligand; GPCR, G protein–coupled receptor; MHC, major histocompatibility complex.

^a^Omitting mutations that occurred in RL13, UL128, UL130, and UL131A probably during passage, or that were engineered during bacterial artificial chromosome construction.

^b^Strains sequenced from strains passaged in cell culture, not taking into account the minority of mutations confirmed from the clinical samples (n = 152, excludes CZ/3/2012, which is the same strain as PRA8).

^c^Strains sequenced directly from clinical material (n = 91).

^d^Strains sequenced directly from clinical material or passaged virus (n = 243).

### Intrahost Diversity

**Figure 2.**
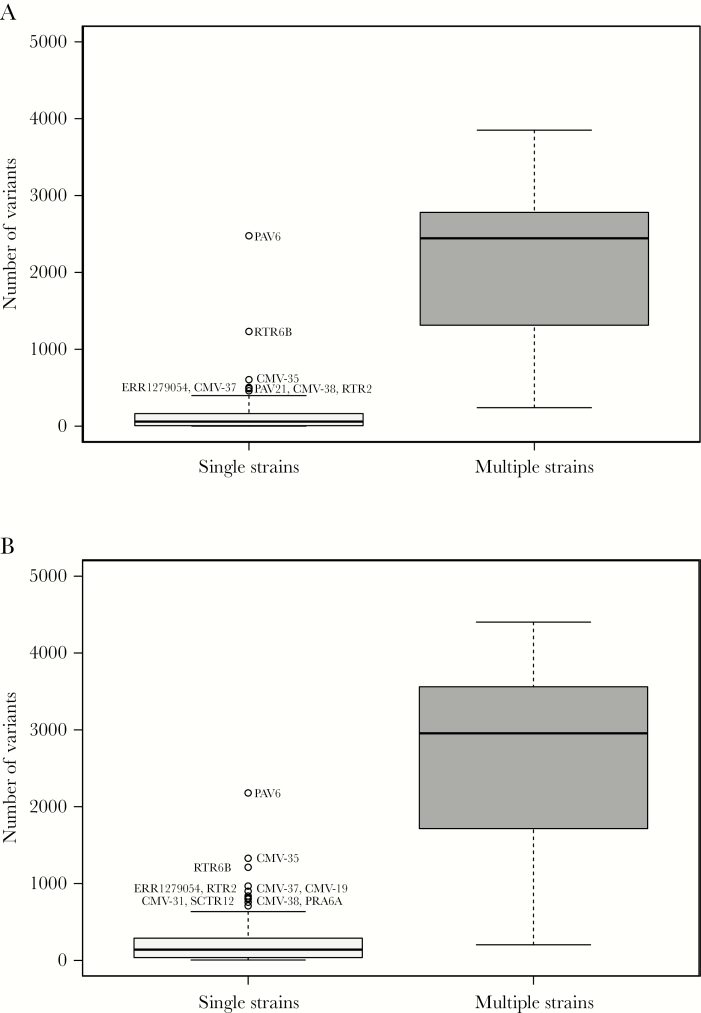
Box-and-whisker graphs created using ggplot2 (https://ggplot2.tidyverse.org) showing the total number of single-nucleotide polymorphisms (SNPs) detected at a frequency of >2% in single-strain and multiple-strain infections using LoFreq (*A*) and V-Phaser (*B*). Single-strain (n = 134 and 131, respectively) and multiple-strain datasets (n = 29 and 29, respectively) for which consensus genome sequences had been derived were identified by motif read-matching, and the total number of SNPs in each dataset was enumerated (insertions, deletions, and length polymorphisms were not considered). LoFreq employed a minimal coverage depth of 10 reads (minimal SNP quality [phred] 64) and strand-bias significance with a false discovery rate correction of *P* < .001. V-Phaser employed phasing with a window size of 500 nucleotides and quality score (phred) 20 for calibrating the significance of strand-bias at *P* < .05. Each box (light gray for single strains and dark gray for multiple strains) encompasses the first to third quartiles (Q1–Q3) and shows the median as a thick line. For each box, the horizontal line at the end of the upper dashed whisker marks the upper extreme (defined as the smaller of Q3 + 1.5 [Q3–Q1] and the highest single value), and the horizontal line at the end of the lower dashed whisker marks indicates the lower extreme (the greater of Q1 – 1.5 [Q3–Q1] and the lowest single value).

LoFreq and V-Phaser analyses showed that single-strain infections contained markedly fewer SNPs (median values of 60 and 140, respectively) than multiple-strain infections (median values of 2444 and 2955, respectively; Figure 2). The differences between the values for single- and multiple-strain infections were significant (Kruskal–Wallis rank-sum test; LoFreq: χ^2^ = 67.918, *P* < 2.2 × 10^-16^; V-Phaser: χ^2^ = 63.536, *P* = 1.6 × 10^-15^).

## DISCUSSION

Advances in high-throughput sequencing technology have made it possible to generate a wealth of viral genome information directly from clinical material. However, operational limitations should be registered. These include sample characteristics (source, viral content and presence of multiple strains), confounding factors (technical limitations, logistical errors and cross-contamination), design of the bait library (ability to enrich all strains and acquire data across the genome), and quality and extent of the sequencing data (library diversity and coverage depth). Since perceived levels of intrahost variation are particularly sensitive to these factors, we proceeded cautiously with this aspect. However, as indicated in our previous study [21], it is clear that the number of SNPs in single-strain infections was markedly less than that in multiple-strain infections. It was also far less than that reported by others in samples from congenital infections [16]. The factors listed above may have been responsible for the outliers observed in single-strain infections; for example, the PAV6 (collection 1) library was made using non-TruGrade oligonucleotides, RTR6B (collection 2) had a low coverage depth and also came from a patient from whom other samples contained multiple strains, and CMV-35 (collection 3) may have contained subthreshold levels of additional strains or cross-contaminants. In our view, accurate estimates of the levels of intrahost variation in single-strain infections are not available from the present and previous studies, and will require sequencing and bioinformatic approaches that are demonstrably reliable, robust, and reproducible [46, 47].

Whole-genome analyses have confirmed the significant role of recombination during HCMV evolution reported in numerous earlier studies [12, 19]. Recombination has occurred over a very long period but nonetheless remains limited in extent, with surviving events being more numerous in long regions, less numerous in short regions, and rare or absent in hypervariable regions, consistent with the role of homologous recombination. Recombination frequency may be restricted in some circumstances by functional interdependence within the same protein (eg, gB) or possibly between separate proteins (eg, gN and gO [25, 32, 44]). However, it is not known whether differential recombination due to sequence relatedness is of general biological significance for the virus. Also, strains have circulated that seem not to have recombined for long periods. Application of an evolutionary rate estimated for herpesviruses (3.5 × 10^−8^ substitutions/nt/year) [48] implies that these periods may have extended to many thousands of years. Moreover, as suggested by the lack of diversity within genotypes in comparison with the marked diversity among them, the distribution of substitutions in nonrecombinant strains fits with the view that intense diversification of the hypervariable genes occurred early in human or pre–human history [25, 31] and has long since ceased.

Assessing the extent to which recombinants arise and survive in individuals with multiple-strain infections is problematic. Except where populations fluctuate significantly and are sampled serially (eg, RTR1 in collection 2), it is difficult to approach this using short-read data, as they are based on PCR methodologies prone to generating recombinational artefacts. Long- or single-read sequencing technologies and demonstrably reliable bioinformatic approaches are needed. Also, conclusions drawn from transplant recipients, who are immunosuppressed and in whom HCMV populations may be diversified by transplantation from HCMV-positive donors or selected with antiviral drugs, are unlikely to represent other situations, such maternal transmission via breast milk [39].

Evidence for pseudogenes was largely derived previously from strains isolated in cell culture, and it was unclear to what extent pseudogenes presented in natural populations. For example, in a study reporting that 75% of strains carry pseudogenes [12], 157 mutations were identified in 101 strains, with all but one of these strains having been passaged in cell culture, although 35 mutations were confirmed by PCR of the clinical material. Nonetheless, we found that the distribution of pseudogenes among the 91 strains sequenced in the present study directly from clinical material is similar to that among strains isolated in cell culture, thus generally validating the earlier suppositions. The likelihood that many of these mutants are ancient is supported by the finding that all were detected at levels very close to 100% in collection 1, and by previous observations identifying the same mutation in different strains [7, 12]. Moreover, 9 of the groups of nonrecombinant strains contained pseudogenes, and some of the mutations were common to group members and even to additional strains among the 243, indicating that they have been transferred by recombination. The implication that some mutants have a selective advantage in certain individuals may be extended to their presence in pathogenic congenital infections, probably in combination with host factors. The genes from which pseudogenes have arisen are involved, or are suspected to be involved, in immune modulation. They include UL111A, which encodes viral interleukin 10 [49]; UL40, which is involved in protecting infected cells against natural killer cell lysis [50] via its cleaved signal peptide, in which mutations occur; and UL9, which bears a potential immunoglobulin-binding domain [2]. These findings also suggest, but do not prove, that maternal HCMV genotyping might be useful in developing strategies for preventing congenital CMV.

Modern approaches offer a powerful means for analyzing HCMV genomes directly from clinical material, with the important proviso that the data should be quality assessed and interpreted in the context of the known evolutionary and biological characteristics of the virus. Extensive high-throughput sequence data are likely to illuminate further the epidemiology, pathogenesis, and evolution of HCMV in clinical and natural settings, thus facilitating the identification of virulence determinants and the development of new interventions.

## Supplementary Data

Supplementary materials are available at *The Journal of Infectious Diseases* online. Consisting of data provided by the authors to benefit the reader, the posted materials are not copyedited and are the sole responsibility of the authors, so questions or comments should be addressed to the corresponding author.

jiz208_suppl_Supplementary_Figure_1

jiz208_suppl_Supplementary_Figure_2

jiz208_suppl_Supplementary_Table_1

jiz208_suppl_Supplementary_Table_2

jiz208_suppl_Supplementary_Table_3

jiz208_suppl_Supplementary_Table_4

jiz208_suppl_Supplementary_Table_5

## Notes

***Acknowledgments.*** We are grateful to Florent Lasalle, Daniel Depledge, and Judith Breuer (University College London) for providing unpublished collection 3 datasets and for updating the associated genome sequences in GenBank. We also thank Jenny Witthuhn (Hannover Medical School) for excellent technical assistance.

***Financial support.*** This work was supported by the Medical Research Council (grant numbers MC_UU_12014/3 and MC_UU_12014/12 to A. J. D.); the Wellcome Trust (grant numbers 204870/Z/16/Z to A. J. D. and WT090323MA to G. W. G. W.); the Ministry of Health of the Czech Republic for conceptual development of research organization (University Hospital, Motol, Prague, Czech Republic, grant number 00064203 to P. H.); the Fondazione Regionale per la Ricerca Biomedica, Regione Lombardia (grant number FRRB 2015-043 to D. L.); the Niedersächsische Ministerium für Wissenschaft und Kultur (grant COALITION–Communities Allied in Infection to T. G.); the Deutsche Forschungsgemeinschaft Collaborative Research Centre 900 (core project Z1, grant number SFB-9001 to T. F. S.); and the German Center of Infection Research Thematic Translational Unit “Infections of the Immunocompromised Host” (grant to T. G. and T. F. S.). Two authors (E. H. and A. D.) were supported by the Infection Biology graduate program of Hannover Biomedical Research School.

***Potential conflicts of interest.*** G. S. W. reports that his part in the present study was completed prior to his present employment. G. W. G. W. has received a grant from the Wellcome Trust. D. L. has received a grant from the Fondazione Regionale per la Ricerca Biomedica, Regione Lombardia. T. G. has received grants from the German Federal Ministry of Education and Research and from the Niedersächsische Ministerium für Wissenschaft und Kultur. P. H. has received a grant from the Ministry of Health of the Czech Republic for the conceptual development of University Hospital, Motol, Prague, Czech Republic; personal fees and nonfinancial support from MSD and from Chimerix; and personal fees from Dynex. T. F. S. has received grants from the Deutsche Forschungsgemeinschaft Collaborative Research Centre 900 and from the German Federal Ministry of Education and Research. A. J. D. has received grants from the Medical Research Council and the Wellcome Trust. All other authors report no potential conflicts of interest.

Presented in part: Seventh International Congenital Cytomegalovirus (CMV) Conference and 17th International CMV Workshop, Birmingham, Alabama, April 2019.

Published as a bioRxiv preprint on 23 December 2018 and revised on 18 February 2019 (https://doi.org/10.1101/505735).
