# Supplementary material for: Human Cytomegalovirus Genomes Sequenced Directly From Clinical Material: Variation, Multiple-Strain Infection, Recombination, and Gene Loss
Source: J Infect Dis. 2019 May 2;220(5):781–91. doi: 10.1093/infdis/jiz208 (PMC6667795; doi:10.1093/infdis/jiz208)
Supplement: jiz208_suppl_Supplementary_Figure_1 [file jiz208_suppl_supplementary_figure_1.docx]

**Supplementary Figure 1.** Nucleotide sequence alignments of genes used for genotyping or that commonly contain mutations.

Sequences are shown in the orientation present in the genome, with gaps introduced by alignment depicted by hyphens.

Sequence names are from **Supplementary Table 1** or GenBank.

Genotype names are highlighted in green.

**RL5A**

Initiation codons (the first and, where present, second ATG codons) and termination codons are highlighted in yellow. Sequences used for motif-matching are indicated in blue font (identifying genotypes) or red, gold or purple font (identifying G1 variants).

Cyan highlighting indicates strains in which this gene is mutated and the mutations responsible: substitutions that introduce in-frame stop codons (underlined) or insertions or deletions that cause frameshifts (the C-tract towards the right end of the alignment is not included).

The scattered locations of second ATG codons suggests that the true initiation codon for all genotypes is the first one. If this is the case, many more strains are mutated because of frameshifts in the C-tract, perhaps indicating that RL5A is essentially a nonfunctional gene that is in the process of being lost.

BE/46/2011 G2 ------TTATTTCACAACTACAAACCATGTACCAACATCCTCTGGACCATTATCAAACCTTCTCCTCAAATCATATGATCCTTCATGTTTAGTTGACACGTTACAAAATCCCAAACTGCTGTGAGTACAGTTACAGATGCCTGTCAAACCCGAATCCGACACCGTATAATTGTTCGTACCCACGACATTACACACGGTTACGTCAGAACCAGTGCCGTTAAAGAATGTCCACGTTGAATTCAACTTAGTATTTAAGGTTGCACTTCTACCAGACGTCATGTCTACACGATGAAAACCCTCAATAACATGGCAGAGTCCCAAAAGTAGGCACGCTGCCAATACCCCCCCCCC---------ACCCAGGAATGTGACTGGACCGGTTCCGAGTAGCCCTAGAAGCCAT

HAN22 ------TTATTTCACAACTACAAACCATGTACCAACATCCTCTGGACCATTATCAAACCTTCTCCTCAAATCATATGATCCTTCATGTTTAGTTGACACGTTACAAAATCCCAAACTGCTGTGAGTACAGTTACAAATGCCTGTCAAACCCGAATCCGACACCGTATAATTGTTCGTACCCACGACATTACACACGGTTACGTCAGAACCAGTGCCGTTAAAGAATGTCCACGTTGAATTCAACTTAGTATTTAAGGTTGCACTTCTACCAGACGTCATGTCTACACGATGAAAACCCTCAATAACATGGCAGAATCCCAAAAGTAGGCACGCTGCCAATACCCCCCCCCC---------ACCCAGGAATGTGACTGGACCGGTTCCGAGTAGCCCTGGGAGCCAT

HAN23 ------TTATTTCACAACTACAAACCATGTACCAACATCCTCTGGACCATTATCAAACCTTCTCCTCAAATCATATGATCCTTCATGTTTAGTTGACACGTTACAAAATCCCAAACTGCTGTGAGTACAGTTACAAATGCCTGTCAAACCCGAATCCGACACCGTATAATTGTTCGTACCCACGACATTACACACGGTTACGTCAGAACCAGTGCCGTTAAAGAATGTCCACGTTGAATTCAACTTAGTATTTAAGGTTGCACTTCTACCAGACGTCATGTCTACACGATGAAAACCCTCAATAACATGGCAGAATCCCAAAAGTAGGCACGCTGCCAATACCCCCCCCCC---------ACCCAGGAATGTGACTGGACCGGTTCCGAGTAGCCCTGGGAGCCAT

BE/40/2011 ------TTATTTCACAACTACAAACCATGTACCAACATCCTCTGGACCATTATCAAACCTTCTCCTCAAATCATATGATCCTTCATGTTTAGTTGACACGTTACAAAATCCCAAACTGCTGTGAGTACAGTTACAGATGCCTGTCAAACCCGAATCCGACACCGTATAATTGTTCGTACCCACGACATTACACACGGTTACGTCAGAACCAGTGCCGTTAAAGAATGTCCACGTTGAATTCAACTTAGTATTTAAGGTTGCACTTTTACCAGACGTCATGTCTACACGATGAAAACCCTCAATAACATGGCAGAGTCCCAAAAGTAGGCACGCTGCCAATACCCCCCCCCC---------ACCCAGGAATGTGACTGGACCGGTTCCGAGTATCCCTGGGAGCCAT

BE/30/2010 ------TTATTTCACAACTACAAACCATGTACCAACATCCTCTGGACCATTATCAAACCTTCTCCTCAAATCATATGATCCTTCATGTTTAGTTGACACGTTACAAAATCCCAAACTGCTGTGAGTACAGTTACAGATGCCTGTCAAACCCGAATCCGACACCGTATAATTGTTCGTACCCACGACATTACACACGGTTACGTCAGAACCAGTGCCGTTAAAGAATGTCCACGTTGAATTCAACTTAGTATTTAAGGTTGCACTTTTACCAGACGTCATGTCTACACGATGAAAACCCTCAATAACATGGCAGAGTCCCAAAAGTAGGCACGCTGCCAATACCCCCCCCCC---------ACCCAGGAATGTGACTGGACCGGTTCCGAGTATCCCTGGGAGCCAT

BE/31/2010 ------TTATTTCACAACTACAAACCATGTACCAACATCCTCTGGACCATTATCAAACCTTCTCCTCAAATCATATGATCCTTCATGTTTAGTTGACACGTTACAAAATCCCAAACTGCTGTGAGTACAGTTACAGATGCCTGTCAAACCCGAATCCGACACCGTATAATTGTTCGTACCCACGACATTACACACGGTTACGTCAGAACCAGTGCCGTTAAAGAATGTCCACGTTGAATTCAACTTAGTATTTAAGGTTGCACTTCTACCAGACGTCATGTCTACACGATGAAAACCCTCAATAACATGGCAGAGTCCCAAAAGTAGGCACGCTGCCAATACCCCCCCCCC---------ACCCAGGAATGTGACTGGACCGGTTCCGAGTAGCCCTGGGAGCCAT

BE/34/2011 ------TTATTTCACAACTACAAACCATGTACCAACATCCTCTGGACCATTATCAAACCTTCTCCTCAAATCATATGATCCTTCATGTTTAGTTGACACGTTACAAAATCCCAAACTGCTGTGAGTACAGTTACAGATGCCTGTCAAACCCGAATCCGACACCGTATAATTGTTCGTACCCACGACATTACACACGGTTACGTCAGAACCAGTGCCGTTAAAGAATGTCCACGTTGAATTCAACTTAGTATTTAAGGTTGCACTTCTACCAGACGTCATGTCTACACGATGAAAACCCTCAATAACATGGCAGAGTCCCAAAAGTAGGCACGCTGCCAATACCCCCCCCCC---------ACCCAGGAATGTGACTGGACCGGTTCCGAGTAGCCCTGGGAGCCAT

BE/16/2012 ------TTATTTCACAACTACAAACCATGTACCAACATCCTCTGGACCATTATCAAACCTTCTCCTCAAATCATATGATCCTTCATGTTTAGTTGACACGTTACAAAATCCCAAACTGCTGTGAGTACAGTTACAGATGCCTGTCAAACCCGAATCCGACACCGTATAATTGTTCGTACCCACGACATTACACACGGTTACGTCAGAACCAGTGCCGTTAAAGAATGTCCACGTTGAATTCAACTTAGTATTTAAGGTTGCACTTCTACCAGACGTCATGTCTACACGATGAAAACCCTCAATAACATGGCAGAGTCCCAAAAGTAGGCACGCTGCCAATACCCCCCCCCC---------ACCCAGGAATGTGACTGGACCGGTTCCGAGTAGCCCTGGGAGCCAT

BE/30/2011 ------TTATTTCACAACTACAAACCATGTACCAACATCCTCTGGACCATTATCAAACCTTCTCCTCAAATCATATGATCCTTCATGTTTAGTTGACACGTTACAAAATCCCAAACTGCTGTGAGTACAGTTACAGATGCCTGTCAAACCCGAATCCGACACCGTATAATTGTTCGTACCCACGACATTACACACGGTTACGTCAGAACCAGTGCCGTTAAAGAATGTCCACGTTGAATTCAACTTAGTATTTAAGGTTGCACTTCTACCAGACGTCATGTCTACACGATGAAAACCCTCAATAACATGGCAGAGTCCCAAAAGTAGGCACGCTGCCAATACCCCCCCCCC---------ACCCAGGAATGTGACTGGACCGGTTCCGAGTAGCCCTGGGAGCCAT

BE/15/2012 ------TTATTTCACAACTACAAACCATGTACCAACATCCTCTGGACCATTATCAAACCTTCTCCTCAAATCATATGATCCTTCATGTTTAGTTGACACGTTACAAAATCCCAAACTGCTGTGAGTACAGTTACAGATGCCTGTCAAACCCGAATCCGACACCGTATAATTGTTCGTACCCACGACATTACACACGGTTACGTCAGAACCAGTGCCGTTAAAGAATGTCCACGTTGAATTCAACTTAGTATTTAAGGTTGCACTTCTACCAGACGTCATGTCTACACGATGAAAACCCTCAATAACATGGCAGAGTCCCAAAAGTAGGCACGCTGCCAATACCCCCCCCCC---------ACCCAGGAATGTGACTGGACCGGTTCCGAGTAGCCCTGGGAGCCAT

U8 ------TTATTTCACAACTACAAACCATGTACCAACATCCTCTGGACCATTATCAAACCTTCTCCTCAAATCATATGATCCTTCATGTTTAGTTGACACGTTACAAAATCCCAAACTGCTGTGAGTACAGTTACAGATGCCTGTCAAACCCGAATCCGACACCGTATAATTGTTCGTACCCACGACATTACACACGGTTACGTCAGAACCAGTGCCGTTAAAGAATGTCCACGTTGAATTCAACTTAGTATTTAAGGTTGCACTTCTACCAGACGTCATGTCTACACGATGAAAACCCTCAATAACATGGCAGAGTCCCAAAAGTAGGCACGCTGCCAATACCCCCCCCCC---------ACCCAGGAATGTGACTGGACCGGTTCCGAGTAGCCCTGGGAGCCAT

BE/3/2012 ------TTATTTCACAACTACAAACCATGTACCAACATCCTCTGGACCATTATCAAACCTTCTCCTCAAATCATATGATCCTTCATGTTTAGTTGACACGTTACAAAATCCCAAACTGCTGTGAGTACAGTTACAGATGCCTGTCAAACCCGAATCCGACACCGTATAATTGTTCGTACCCACGACATTACACACGGTTACGTCAGAACCAGTGCCGTTAAAGAATGTCCACGTTGAATTCAACTTAGTATTTAAGGTTGCACTTCTACCAGACGTCATGTCTACACGATGAAAACCCTCAATAACATGGCAGAGTCCCAAAAGTAGGCACGCTGCCAATACCCCCCCCCC---------ACCCAGGAATGTGACTGGACCGGTTCCGAGTAGCCCTGGGAGCCAT

BE/3/2011 ------TTATTTCACAACTACAAACCATGTACCAACATCCTCTGGACCATTATCAAACCTTCTCCTCAAATCATATGATCCTTCATGTTTAGTTGACACGTTACAAAATCCCAAACTGCTGTGAGTACAGTTACAGATGCCTGTCAAACCCGAATCCGACACCGTATAATTGTTCGTACCCACGACATTACACACGGTTACGTCAGAACCAGTGCCGTTAAAGAATGTCCACGTTGAATTCAACTTAGTATTTAAGGTTGCACTTCTACCAGACGTCATGTCTACACGATGAAAACCCTCAATAACATGGCAGAGTCCCAAAAGTAGGCACGCTGCCAATACCCCCCCCCC---------ACCCAGGAATGTGACTGGACCGGTTCCGAGTAGCCCTGGGAGCCAT

BE/21/2011 ------TTATTTCACAACTACAAACCATGTACCAACATCCTCTGGACCATTATCAAACCTTCTCCTCAAATCATATGATCCTTCATGTTTAGTTGACACGTTACAAAATCCCAAACTGCTGTGAGTACAGTTACAGATGCCTGTCAAACCCGAATCCGACACCGTATAATTGTTCGTACCCACGACATTACACACGGTTACGTCAGAACCAGTGCCGTTAAAGAATGTCCACGTTGAATTCAACTTAGTATTTAAGGTTGCACTTCTACCAGACGTCATGTCTACACGATGAAAACCCTCAATAACATGGCAGAGTCCCAAAAGTAGGCACGCTGCCAATACCCCCCCCCC---------ACCCAGGAATGTGACTGGACCGGTTCCGAGTAGCCCTGGGAGCCAT

HANRTR2 ------TTATTTCACAACTACAAACCATGTACCAACATCCTCTGGACCATTATCAAACCTTCTCCTCAAATCATATGATCCTTCATGTTTAGTTGACACGTTACAAAATCCCAAACTGCTGTGAGTACAGTTACAGATGCCTGTCAAACCCGAATCCGACACCGTATAATTGTTCGTACCCACGACATTACACACGGTTACGTCAGAACCAGTGCCGTTAAAGAATGTCCACGTTGAATTCAACTTAGTATTTAAGGTTGCACTTCTACCAGACGTCATGTCTACACGATGAAAACCCTCAATAACATGGCAGAGTCCCAAAAGTAGGCACGCTGCCAATACCCCCCCCCC---------ACCCAGGAATGTGACTGGACCGGTTCCGAGTAGCCCTGGGAGCCAT

BE/7/2012 ------TTATTTCACAACTACAAACCATGTACCAACATCCTCTGGACCATTATCAAACCTTCTCCTCAAATCATATGATCCTTCATGTTTAGTTGACACGTTACAAAATCCCAAACTGCTGTGAGTACAGTTACAGATGCCTGTCAAACCCGAATCCGACACCGTATAATTGTTCGTACCCACGACATTACACACGGTTACGTCAGAACCAGTGCCGTTAAAGAATGTCCACGTTGAATTCAACTTAGTATTTAAGGTTGCACTTCTACCAGACGTCATGTCTACACGATGAAAACCCTCAATAACATGGCAGAGTCACAAAAGTAGGCACGCTGCCAATACCCCCCCCCC---------ACCCAGGAATGTGACTGGACCGGTTCCGAGTAGCCCTGGGAGCCAT

BE/26/2010 ------TTATTTCACAACTACAAACCATGTACCAACATCCTCTGGACCATTATCAAACCTTCTCCTCAAATCATATGATCCTTCATGTTTAGTTGACACGTTACAAAATCCCAAACTGCTGTGAGTACAGTTACAGATGCCTGTCAAACCCGAATCCGACACCGTATAATTGTTCGTACCCACGACATTACACACGGTTACGTCAGAACCAGTGCCGTTAAAGAATGTCCACGTTGAATTCAACTTAGTATTTAAGGTTGCACTTCTACCAGACGTCATGTCTACACGATGAAAACCCTCAATAACATGGCAGAGTCCCAAAAGTAGGCACGCTGCCAATACCCCCCCCCCC--------ACCCAGGAATGTGACTGGACCGGTTCCGAGTAGCCCTGGGAGCCAT

HAN31 ------TTATTTCACAACTACAAACCATGTACCAACATCCTCTGGACCATTATCAAACCTTCTCCTCAAATCATATGATCCTTCATGTTTAGTTGACACGTTACAAAATCCCAAACTGCTGTGAGTACAGTTACAGATGCCTGTCAAACCCGAATCCGACACCGTATAATTGTTCGTACCCACGACATTACACACGGTTACGTCAGAACCAGTGCCGTTAAAGAATGTCCACGTTGAATTCAACTTAGTATTTAAGGTTGCACTTCTACCAGACGTCATGTCTACACGATGAAAACCCTCAATAACATGGCAGAGTCCCAAAAGTAGGCACGCTGCCAATACCCCCCCCC----------ACCCAGGAATGTGACTGGACCGGTTCCGAGTAGCCCTGGGAGCCAT

PAV25 ------TTATTTCACAACTACAAACCATGTACCAACATCCTCTGGACCATTATCAAACCTTCTCCTCAAATCATATGATCCTTCATGTTTAGTTGACACGTTACAAAATCCCAAACTGCTGTGAGTACAGTTACAGATGCCTGTCAAACCCGAATCCGACACCGTATAATTGTTCGTACCCACGACATTACACACGGTTACGTCAGAACCAGTGCCGTTAAAGAATGTCCACGTTGAATTCAACTTAGTATTTAAGGTTGCACTTCTACCAGACGTCATGTCTACACGATGAAAACCCTCAATAACATGGCAGAGTCCCAAAAGTAGGCACGCTGCCAATACCCCCCCCC----------ACCCAGGAATGTGACTGGACCGGTTCCGAGTAGCCCTGGGAGCCAT

BE/2/2011 ------TTATTTCACAACTACAAACCATGTACCAACATCCTCTGGACCATTATCAAACCTTCTCCTCAAATCATATGATCCTTCATGTTTAGTTGACACGTTACAAAATCCCAAACTGCTGTGAGTACAGTTACAGATGCCTGTCAAACCCGAATCCGACACCGTATAATTGTTCGTACCCACGACATTACACACGGTTACGTCAGAACCAGTGCCGTTAAAGAATGTCCACGTTGAATTCAACTTAGTATTTAAGGTTGCACTTCTACCAGACGTCATGTCTACACGATGAAAACCCTCAATAACATGGCAGAGTCCCAAAAGTAGGCACGCTGCCAATACCCCCCCCC----------ACCCAGGAATGTGACTGGACCGGTTCCGAGTAGCCCTGGGAGCCAT

BE/7/2011 ------TTATTTCACAACTACAAACCATGTACCAACATCCTCTGGACCATTATCAAACCTTCTCCTCAAATCATATGATCCTTCATGTTTAGTTGACACGTTACAAAATCCCAAACTGCTGTGAGTACAGTTACAGATGCCTGTCAAACCCGAATCCGACACCGTATAATTGTTCGTACCCACGACATTACACACGGTTACGTCAGAACCAGTGCCGTTAAAGAATGTCCACGTTGAATTCAACTTAGTATTTAAGGTTGCACTTCTACCAGACGTCATGTCTACACGATGAAAACCCTCAATAACATGGCAGAGTCCCAAAAGTAGGCACGCTGCCAATACCCCCCCCC----------ACCCAGGAATGTGACTGGACCGGTTCCGAGTAGCCCTGGGAGCCAT

PAV18 ------TTATTTCACAACTACAAACCATGTACCAACATCCTCTGGACCATTATCAAACCTTCTCCTCAAATCATATGATCCTTCATGTTTAGTTGACACGTTACAAAATCCCAAACTGCTGTGAGTACAGTTACAGATGCCTGTCAAACCCGAATCCGACACCGTATAATTGTTCGTACCCACGACATTACACACGGTTACGTCAGAACCAGTGCCGTTAAAGAATGTCCACGTTGAATTCAACTTAGTATTTAAGGTTGCACTTCTACCAGACGTCATGTCTACACGATGAAAACCCTCAATAACATGGCAAAGTCCCAAAAGTAGGCACGCTGCCAATACCCCCCCCC----------ACCCAGGAATGTGACTGGACCGGTTCCGAGTAGCCCTGGGAGCCAT

HAN21 ------TTATTTCACAACTACAAACCATGTACCAACATCCTCTGGACCATTATCAAACCTTCTCCTCAAATCATATGATCCTTCATGTTTAGTTGACACGTTACAAAATCCCAAACTGCTGTGAGTACAGTTACAGATGCCTGTCAAACCCGAATCCGACACCGTATAATTGTTCGTACCCACGACATTACACACGGTTACGTCAGAACCAGTGCCGTTAAAGAATGTCCACGTTGAATTCAACTTAGTATTTAAGGTTGCACTTCTACCAGACGTCATGTCTACACGATGAAAACCCTCAATAACATGGCAAAGTCCCAAAAGTAGGCACGCTGCCAATACCCCCCCCC----------ACCCAGGAATGTGACTGGACCGGTTCCGAGTAGCCCTGGGAGCCAT

HANSCTR1A ------TTATTTCACAACTACAAACCATGTACCAACATCCTCTGGACCATTATCAAACCTTCTCCTCAAATCATATGATCCTTCATGTTTAGTTGACACGTTACAAAATCCCAAACTGCTGTGAGTACAGTTACAGATGCCTGTCAAACCCGAATCCGACACCGTATAATTGTTCGTACCCACGACATTACACACGGTTACGTCAGAACCAGTGCCGTTAAAGAATGTCCACGTTGAATTCAACTTAGTATTTAAGGTTGCACTTCTACCAGACGTCATGTCTACACGATGAAAACCCTCAATAACATGGCAAAGTCCCAAAAGTAGGCACGCTGCCAATACCCCCCCCC----------ACCCAGGAATGTGACTGGACCGGTTCCGAGTAGCCCTGGGAGCCAT

BE/11/2012 ------TTATTTCACAACTACAAACCATGTACCAACATCCTCTGGACCATTATCAAACCTTCTCCTCAAATCATATGATCCTTCATGTTTAGTTGACACGTTACAAAATCCCAAACTGCTGTGAGTACAGTTACAGATGCCTGTCAAACCCGAATCCGACACCGTATAATTGTTCGTACCCACGACATTACACACGGTTACGTCAGAACCAGTGCCGTTAAAGAATGTCCACGTTGAATTCAACTTAGTATTTAAGGTTGCACTTCTACCAGACGTCATGTCTACACGATGAAAACCCTCAATAACATGGCAGAGTCCCAAAAGTAGGCACGCTGCCAATACCCCCCCCCCCCCCCCCCCACCCAGGAATGTGACTGGACCGGTTCCGAGTAGCCCTGGGAGCCAT

BE/32/2011 ------TTATTTCACAACTACAAACCATGTACCAACATCCTCTGGACCATTATCAAACCTTCTCCTCAAATCATATGATCCTTCATGTTTAGTTGACACGTTACAAAATCCCAAACTGCTGTGAGTACAGTTACAGATGCCTGTCAAACCCGAATCCGACACCGTATAATTGTTCGTACCCACGACATTACACACGGTTACGTCAGAACCAGTGCCGTTAAAGAATGTCCACGTTGAATTCAACTTAGTATTTAAGGTTGCACTTCTACCAGACGTCATGTCTACACGATGAAAACCCTCAATAACATGGCAGAATCCCAAAAGTAGGCACGCTGCCAATACCCCCCCCCCCC-------ACCCAGGAATGTGACTGGACCGGTTCCGAGTAGCCCTGGGAGCCAT

BE/31/2011 ------TTATTTCACAACTACAAACCATGTACCAACATCCTCTGGACCATTATCAAACCTTCTCCTCAAATCATATGATCCTTCATGTTTAGTTGACACGTTACAAAATCCCAAACTGCTGTGAGTACAGTTACAGATGCCTGTCAAACCCGAATCCGACACCGTATAATTGTTCGTACCCACGACATTACACACGGTTACGTCAGAACCAGTGCCGTTAAAGAATGTCCACGTTGAATTCAACTTAGTATTTAAGGTTGCACTTCTACCAGACGTCATGTCTACACGATGAAAACCCTCAATAACATGGCAGAGTCCCAAAAGTAGGCACGCTGCCAATACCCCCCCCCCCC-------ACCCAGGAATGTGACTGGACCGGTTCCGAGTAGCCCTGGGAGCCAT

BE/17/2011 G4 CTACGATGAACTCACAGAGACCAACCATGTCTCTTTAATTTCACTCGGACCGTTACCGTTTTTCCAAGTCAGATCGTAACCTCCTTCGTTTCCAGACGTAACGTTACACAGCGTCAGATGCACATGACTGCAATTGCACACGCCACCGACCGATCCCAACGTAACATTGTTAACGCCTGTCACGGTACAAACCGTAGTTTCATTATGCTTAGTTCCATCCGTAAGCGTCCAAGTTGACGTAAGACCATAATTTAGATTTGTATTTCCACCAATCTTTATACTAGAACTTTTAAAACTTTCAACTTCAAGGCATAGTGCCAAAATGAGGCACGTTATTAGTCCCCCCCC------------ACCAAGGAATGTGACTGGACCGGTTCCGAGTAGCCCTGGGAGCCAT

JER5550 CTACGATGAACTCACAGAGACCAACCATGTCTCTTTAATTTCACTCGGACCGTTACCGTTTTTCCAAGTCAGATCGTAACCTCCTTCGTTTCCAGACGTAACGTTACACAGCGTCAGATGCACATGACTGCAATTGCACACGCCACCGACCGATCCCAACGTAACATTGTTAACGCCTGTCACGGTACAAACCGTAGTTTCATTATGCTTAGTTCCATCCGTAAGCGTCCAAGTTGACGTAAGACCATAATTTAGATTTGTATTTCCACCAACCTTTATACTAGAACTTTTAAAACTTTCAACTTCAAGGCATAGTGCCAAAATGAGGCACGTTATTAGTCCCCCCCCCCC---------ACCAAGGAATGTGACTGGACCGGTTCCGAGTAGCCCTGGGAGCCAT

HAN28 CTACGATGAACTCACAGAGACCAACCATGTCTCTTTAATTTCACTCGGACCGTTACCGTTTTTCCAAGTCAGATCGTAACCTCCTTCGTTTCCAGACGTAACGTTACACAGCGTCAGATGCACATGACTGCAATTGCACACGCCACCGACCGATCCCAACGTAACATTGTTAACGCCTGTCACGGTACAAACCGTAGTTTCATTATGCTTAGTTCCATCCGTAAGCGTCCAAGTTGACGTAAGACCATAATTTAGATTTGTATTTCCACCAACCTTTATACTAGAACTTTTAAAACTTTCAACTTCAAGGCATAGTGCCAAAATGAGGCACGTTATTAGTCCCCCCCCCCC---------ACCAAGGAATGTGACTGGACCGGTTCCGAGTAGCCCTGGGAGCCAT

HAN39 CTACGATGAACTCACAGAGACCAACCATGTCTCTTTAATTTCACTCGGACCGTTACCGTTTTTCCAAGTCAGATCGTAACCTCCTTCGTTTCCAGACGTAACGTTACACAGCGTCAGATGCACATGACTGCAATTGCACACGCCACCGACCGATCCCAACGTAACATTGTTAACGCCTGTCACGGTACAAACCGTAGTTTCATTATGCTTAGTTCCATCCGTAAGCGTCCAAGTTGACGTAAGACCATAATTTAGATTTGTATTTCCACCAACCTTTATACTAGAACTTTTAAAACTTTCAACTTCAAGGCATAGTGCCAAAATGAGGCACGTTATTAGTCCCCCCCCCCC---------ACCAAGGAATGTGACTGGACCGGTTCCGAGTAGCCCTGGGAGCCAT

HAN38 CTATGATGAACTCACAGAGACCAACCATGTCTCTTTAATTTCACTCGGACCGTTACCGCTTTTCCAAGTCAGATCGTAACCTCCTTCGTTTCCAGACGTAACGTTACACAGCGTCAGATGCACATGACTGCAATTGCACACGCCACCGACCGATCCCAACGTAACATTGTTAACGCCTGTCACAGTACAAACCGTAGTTTCATTATGCTTAGTTCCATCCGTAAGCGTCCAAGTTGACGTAAGACCATAATTTAGATTTGTATTTCCACCAACCTTTATACTAGAACTTTTAAAACTTTCAACTTCAAGGCATAGTGCCAAAATGAGGCACGTTATTAGTCCCCCCCCCCC---------ACCAAGGAATGTGACTGGACCGGTTCCGAGTAGCCCTGGGAGCCAT

BE/33/2010 CTATGATGAACTCACAGAGACCAACCATGTCTCTTTAATTTCACTCGGACCGTTACCGTTTTTCCAAGTCAGATCGTAACCTCCTTCGTTTCCAGACGTAACGTTACACAGCGTCAGATGCACATGACTGCAATTGCACACGCCACCGACCGATCCCAACGTAACATTGTTAACGCCTGTCACAGTACAAACCGTAGTTTCATTATGCTTAGTTCCATCCGTAAGCGTCCAAGTTGACGTAAGACCATAATTTAGATTTGTATTTCCACCGACCTTTATACTAGAACTTTTAAAACTTTCAACTTCAAGGCATAGTGCCAAAATGAGGCACGTTATTAGTCCCCCCCCCCC---------ACCAAGGAATGTGACTGGACCGGTTCCGAGTAGCCCTGGGAGCCAT

UK/Lon1 CTATGATGAACTCACAGAGACCAACCATGTCTCTTTAATTTCACTCGGACCGTTACCGTTTTTCCAAGTCAGATCGTAACCTCCTTCGTTTCCAGACGTAACGTTACACAGCGTCAGATGCACATGACTGCAATTGCACACGCCACCGACCGATCCCAACGTAACATTGTTAACGCCTGTCACAGTACAAACCGTAGTTTCATTATGCTTAGTTCCATCCGTAAGCGTCCAAGTTGACGTAAGACCATAATTTAGATTTGTATTTCCACCAACCTTTATACTAGAACTTTTAAAACTTTCAACTTCAAGGCATAGTGCCAAAATGAGGCACGTTATTAGTCCCCCCCCCCC---------ACCAAGGAATGTGACTGGACCGGTTCCGAGTAGCCCTGGGAGCCAT

BE/43/2011 G3 CTACGATTGACCAACAGAGACCAACCATGTCTCTTTAACCTCGCTTGGGCCGCTACCGTCTTTCCAAACCAGATCGTAACCTCCTTCAT--------------------------------------------TTACATATGCCACCGACCGATCCCAACGTAAAATTGTTTACGCCTGTCACGGTACAAACCGTGGTCTCATTATGTTTAGTTCCATCCGTAAGCTTCCAAGTTGACGTAAGATCGTGTTTTAGATTTGTATTTTCACCAGCCTTTATACTAGAACTTTTAAAACTTTCAACTTCAAGGCATAATGCCAAAATTAAGCACGTTATTAGCCCCCCCCC------------CCCGAGGAATGTGACTGGACCGGTTCTTAGCAGCTTTGGGAGCCAT

BE/38/2011 CTACGATTGACCAACAGAGACCAACCATGTCTCTTTAACCTCGCTTGGGCCGCTACCGTCTTTCCAAACCAGATCGTAACCTCCTTCATTTACAGACGTAACGTTACACAGCGTTAAATGCACATGACTGCAATTACATATGCCACCGACCGATCCCAACGTAAAATTGTTTACGCCTGTCACGGTACAAACCGTGGTCTCATTATGTTTAGTTCCATCCGTAAGCTTCCAAGTTGACGTAAGATCGTGTTTTAGATTTGTATTTTCACCAGCCTTTATACTAGAACTTTTAAAACTTTCAACTTCAAGGCATAATGCCAAAATTAAGCACGTTATTAGCCCCCCCC-------------CCCGAGGAATGTGACTGGACCGGTTCTTAGCAGCTTTGGGAGCCAT

Pat_A CTACGATTGACCAACAGAGACCAACCATGTCTCTTTAACCTCGCTTGGGCCGCTACCGTCTTTCCAAACCAGATCGTAACCTCCTTCATTTACAGACGTAACGTTACACAGCGTTAAATGCACATGACTGCAATTACATATGCCACCGACCGATCCCAACGTAAAATTGTTTACGCCTGTCACGGTACAAACCGTGGTCTCATTATGTTTAGTTCCATCCGTAAGCTTCCAAGTTGACGTAAGATCGTGTTTTAGATTTGTATTTTCACCAGCCTTTATACTAGAACTTTTAAAACTTTCAACTTCAAGGCATAATGCCAAAATTAAGCACGTTATTAGCCCCCCCC-------------CCCGAGGAATGTGACTGGACCGGTTCTTAGCAGCTTTGGGAGCCAT

UKNEQAS2 CTACGATTGACCAACAGAGACCAACCATGTCTCTTTAACCTCGCTTGGGCCGCTACCGTCTTTCCAAACCAGATCGTAACCTCCTTCATTTACAGACGTAACGTTACACAGCGTTAAATGCACATGACTGCAATTACATATGCCACCGACCGATCCCAACGTAAAATTGTTTACGCCTGTCACGGTACAAACCGTGGTCTCATTATGTTTAGTTCCATCCGTAAGCTTCCAAGTTGACGTAAGATCGTGTTTTAGATTTGTATTTTCACCAGCCTTTATACTAGAACTTTTAAAACTTTCAACTTCAAGGCATAATGCCAAAATTAAGCACGTTATTAGCCCCCCCCC------------CCCGAGGAATGTGACTGGACCGGTTCTTAGCAGCTTTGGGAGCCAT

BE/37/2011 CTACGATTGACCAACAGAGACCAACCATGTCTCTTTAACCTCGCTTGGGCCGCTACCGTCTTTCCAAACCAGATCGTAACCTCCTTCATTTACAGACGTAACGTTACACAGCGTTAAATGCACATGACTGCAATTACATATGCCACCGACCGATCCCAACGTAAAATTGTTTACGCCTGTCACGGTACAAACCGTGGTCTCATTATGTTTAGTTCCATCCGTAAGCTTCCAAGTTGACGTAAGATCGTGTTTTAGATTTGTATTTTCACCAGCCTTTATACTAGAACTTTTAAAACTTTCAACTTCAAGGCATAATGCCAAAATTAAGCACGTTATTAGCCCCCCCCC------------CCCGAGGAATGTGACTGGACCGGTTCTTAGCAGCTTTGGGAGCCAT

NAN1LA CTACGATTGACCAACAGAGACCAACCATGTCTCTTTAACCTCGCTTGGGCCGCTACCGTCTTTCCAAACCAGATCGTAACCTCCTTCATTTACAGACGTAACGTTACACAGCGTTAAATGCACATGACTGCAATTACATATGCCACCGACCGATCCCAACGTAAAATTGTTTACGCCTGTCACGGTACAAACCGTGGTCTCATTATGTTTAGTTCCATCCGTAAGCTTCCAAGTTGACGTAAGATCGTGTTTTAGATTTGTATTTTCACCAGCCTTTATACTAGAACTTTTAAAACTTTCAACTTCAAGGCATAATGCCAAAATTAAGCACGTTATTAGCCCCCCCCC------------ACCGAGGAATGTGACTGGACCGGTTCTTAGCAGCTTTGGGAGCCAT

HAN12 CTACGATTGACCAACAGAGACCAACCATGTCTCTTTAACCTCGCTTGGGCCGCTACCGTCTTTCCAAACCAGATCGTAACCTCCTTCATTTACAGACGTAACGTTACACAGCGTTAAATGCACATGACTGCAATTACATATGCCACCGACCGATCCCAACGTAAAATTGTTTACGCCTGTCACGGTACAAACCGTGGTCTCATTATGTTTAGTTCCATCCGTAAGCTTCCAAGTTGACGTAAGATCGTGTTTTAGATTTGTATTTTCACCAGCCTTTATACTAGAACTTTTAAAACTTTCAACTTCAAGGCATAATGCCAAAATTAAGCACGTTATTAGCCCCCCCCCC-----------CCCGAGGAATGTGACTGGACCGGTTCTTAGCAGCTTTGGGAGCCAT

HAN15 CTACGATTGACCAACAGAGACCAACCATGTCTCTTTAACCTCGCTTGGGCCGCTACCGTCTTTCCAAACCAGATCGTAACCTCCTTCATTTACAGACGTAACGTTACACAGCGTTAAATGCACATGACTGCAATTACATATGCCACCGACCGATCCCAACGTAAAATTGTTTACGCCTGTCACGGTACAAACCGTGGTCTCATTATGTTTAGTTCCATCCGTAAGCTTCCAAGTTGACGTAAGATCGTGTTTTAGATTTGTATTTTCACCAGCCTTTATACTAGAACTTTTAAAACTTTCAACTTCAAGGCATAATGCCAAAATTAAGCACGTTATTAGCCCCCCCCCC-----------CCCGAGGAATGTGACTGGACCGGTTCTTAGCAGCTTTGGGAGCCAT

PAV24 CTACGATTGACCAACAGAGACCAACCATGTCTCTTTAACCTCGCTTGGGCCGCTACCGTCTTTCCAAACCAGATCGTAACCTCCTTCATTTACAGACGTAACGTTACACAGCGTTAAATGCACATGACTGCAATTACATATGCCACCGACCGATCCCAACGTAAAATTGTTTACGCCTGTCACGGTACAAACCGTGGTCTCATTATGTTTAGTTCCATCCGTAAGCTTCCAAGTTGACGTAAGATCGTGTTTTAGATTTGTATTTTCACCAGCCTTTATACTAGAACTTTTAAAACTTTCAACTTCAAGGCATAATGCCAAAATTAAGCACGTTATTAGCCCCCCCCCC-----------ACCGAGGAATGTGACTGGACCGGTTCTTAGCAGCTTTGGGAGCCAT

BE/6/2012 CTACGATTGACCAACAGAGACCAACCATGTCTCTTTAACCTCGCTTGGGCCGCTACCGTCTTTCCAAACCAGATCGTAACCTCCTTCATTTACAGACGTAACGTTACACAGCGTTAAATGCACATGACTGCAATTACATATGCCACCGACCGATCCCAACGTAAAATTGTTTACGCCTGTCACGGTACAAACCGTGGTCTCATTATGTTTAGTTCCATCCGTAAGCTTCCAAGTTGACGTAAGATCGTGTTTTAGATTTGTATTTTCACCAGCCTTTATACTAGAACTTTTAAAACTTTCAACTTCAAGGCATAATGCCAAAATTAAGCACGTTATTAGCCCCCCCCCCCCACCC-----ACCGAGGAATGTGACTGGACCGGTTCTTAGCAGCTTTGGGAGCCAT

CZ/2/2013 CTACGATTGACCAACAGAGACCAACCATGTCTCTTTAACCTCGCTTGGGCCGCTACCGTCTTTCCAAACCAGATCGTAACCTCCTTCATTTACAGACGTAACGTTACACAGCGTTAAATGCACATGGCTGCAATTACATATGCCACCGACCAATCCCAACGTAAAATTGTTTACGCCTGTCACGGTACAAACCGTGGTCTCATTATGTTTAGTTCCATCCGTAAGCTTCCAAGTTGACGTAAGATCGTGTTTTAGATTTGTATTTTCACCAACCTTTATACTAGAACTTTTAAAACTTTCAACTTCAAGGCATAATGCCAAAATTAAGCACGTTATTAGTCCCCCCCCCC----------ACCGAGGAATGTGACTGGACCGGTTCTTAGCAGCTTTGGGAGCCAT

BE/5/2010 CTACGATTGACCAACAGAGACCAACCATGTCTCTTTAACCTCGCTTGGGCCGCTACCGTCTTTCCAAACCAGATCGTAACCTCCTTCATTTACAGACGTAACGTTACACAGCGTTAAATGCACATGACTGCAATTACATATGCCACCGACCGATCCCAACGTAAAATTGTTTACGCCTGTCACGGTACAAACCGTGGTCTCATTATGTTTAGTTCCATCCGTAAGCTTCCAAGTTGACGTAAGATCGTGTTTTAGATTTGTATTTTCACCAGCCTTTATACTAGAACTTTTAAAACTTTCAACTTCAAGGCATAATGCCAAAATTAAGCACGTTATTAGTCCCCCCCCCC----------ACCGAGGAATGTGACTGGACCGGTTCTTAGCAGCTTTGGGAGCCAT

BE/12/2011 CTACGATTGACCAACAGAGACCAACCATGTCTCTTTAACCTCGCTTGGGCCGCTACCGTCTTTCCAAACCAGATCGTAACCTCCTTCATTTACAGACGTAACGTTACACAGCGTTAAATGCACATGACTGCAATTACATATGCCACCGACCGATCCCAACGTAAAATTGTTTACGCCTGTCACGGTACAAACCGTGGTCTCATTATGTTTAGTTCCATCCGTAAGCTTCCAAGTTGACGTAAGATCGTGTTTTAGATTTGTATTTTCACCAGCCTTTATACTAGAACTTTTAAAACTTTCAACTTCAAGGCATAATGCCAAAATTAAGCACGTTATTAGCCCCCCCCCCC----------ACCGAGGAATGTGACTGGACCGGTTCTTAGCAGCTTTGGGAGCCAT

Pat_F CTACGATTGACCAACAGAGACCAACCATGTCTCTTTAACCTCGCTTGGGCCGCTACCGTCTTTCCAAACCAGATCGTAACCTCCTTCATTTACAGACGTAACGTTACACAGCGTTAAATGCACATGACTGCAATTACATATGCCACCGACCGATCCCAACGTAAAATTGTTTACGCCTGTCACGGTACAAACCGTGGTCTCATTATGTTTAGTTCCATCCGTAAGCTTCCAAGTTGACGTAAGATCGTGTTTTAGATTTGTATTTTCACCAGCCTTTATACTAGAACTTTTAAAACTTTCAACTTCAAGGCATAATGCCAAAATTAAGCACGTTATTAGCCCCCCCCCCC----------ACCGAGGAATGTGACTGGACCGGTTCTTAGCAGCTTTGGGAGCCAT

BE/36/2011 CTACGATTGACCAACAGAGACCAACCATGTCTCTTTAACCTCGCTTGGGCCGCTACCGTCTTTCCAAACCAGATCGTAACCTCCTTCATTTACAGACGTAACGTTACACAGCGTTAAATGCACATGACTGCAATTACATATGCCACCGACCGATCCCAACGTAAAATTGTTTACGCCTGTCACGGTACAAACCGTGGTCTCATTATGTTTAGTTCCATCCGTAAGCTTCCAAGTTGACGTAAGATCGTGTTTTAGATTTGTATTTTCACCAGCCTTTATACTAGAACTTTTAAAACTTTCAACTTCAAGGCATAATGCCAAAATTAAGCACGTTATTAGTCCCCCCCCCCC---------ACCGAGGAATGTGACTGGACCGGTTCTTAGCAGCTTTGGGAGCCAT

HANSCTR11B CTACGATTGACCAACAGAGACCAATCATGTCTCTTTAACCTCGCTTGGGCCGCTACCGTCTTTCCAAACCAGATCGTAACCTCCTTCATTTACAGACGTAACGTTACACAGCGTTAAATGCACATGGCTGCAATTACATATGCCACCGACCGATCCCAACGTAAAATTGTTTACGCCTGTCACGGTACAAACCGTGGTCTCATTATGTTTAGTTCCATCCGTAAGCTTCCAAGTTGACGTAAGATCGTGTTTTAGATTTGTATTTTCACCAACCTTTATACTAGAACTTTTAAAACTTTCAACTTCAAGGCATAATGCCAAAATTAAGCACGTTATTAGTCCCCCCCCCCC---------ACCGAGGAATGTGACTGGACCGGTTCTTAGCAGCTTTGGGAGCCAT

BE/19/2011 CTACGATTGACCAACAGAGACCAACCATGTCTCTTTAACCTCGCTTGGGCCGCTACCGTCTTTCCAAACCAGATCGTAACCTCCTTCATTTACAGACGTAACGTTACACAGCGTTAAATGCACATGGCTGCAATTACATATGCCACCGACCGATCCCAACGTAAAATTGTTTACGCCTGTCACGGTACAAACCGTGGTCTCATTATGTTTAGTTCCATCCGTAAGCTTCCAAGTTGACGTAAGATCGTGTTTTAGATTTGTATTTTCACCAACCTTTATACTAGAACTTTTAAAACTTTCAACTTCAAGGCATAATGCCAAAATTAAGCACGTTATTAATCCCCCCCCCCC---------ACCGAGGAATGTGACTGGACCGGTTCTTAGCAGCTTTGGGAGCCAT

HAN25 ------TTAACCAACAGAGACCAACCATGTCTCTTTAACCTCGCTTGGGCCGCTACCGTCTTTCCAAACCAGATCGTAACCTCCTTCATTTACAGACGTAACGTTACACAGCGTTAAATGCACATGGCTGCAATTACATATGCCACCGACCGATCCCAACGTAAAATTGTTTACGCCTGTCACGGTACAAACCGTGGTCTCATTATGTTTAGTTCCATCCGTAAGCTTCCAAGTTGACGTAAGATCGTGTTTTAGATTTGTATTTTCACCAACCTTTATACTAGAACTTTTAAAACTTTCAACTTCAAGGCATAATGCCAAAATTAAGCACGTTATTAGTCCCCCCCCCCC---------ACCGAGGAATGTGACTGGACCGGTTCTTAGCAGCTTTGGGAGCCAT

BE/14/2010 CTACGATTGACCAACAGAGACCAACCATGTCTCTTTAACCTCGCTTGGGCCGCTACCGTCTTTCCAAACCAGATCGTAACCTCCTTCATTTACAGACGTAACGTTACACAGCGTTAAATGCACATGGCTGCAATTACATATGCCACCGACCGATCCCAACGTAAAATTGTTTACGCCTGTCACGGTACAAACCGTGGTCTCATTATGTTTAGTTCCATCCGTAAGCTTCCAAGTTGACGTAAGATCGTGTTTTAGATTTGTATTTTCACCAACCTTTATACTAGAACTTTTAAAACTTTCAACTTCAAGGCATAATGCCAAAATTAAGCACGTTATTAGTCCCCCCCCCCC---------ACCGAGGAATGTGACTGGACCGGTTCTTAGCAGCTTTGGGAGCCAT

BE/11/2011 CTACGATTGACCAACAGAGACCAACCATGTCTCTTTAACCTCGCTTGGGCCGCTACCGTCTTTCCAAACCAGATCGTAACCTCCTTCATTTACAGACGTAACGTTACACAGCGTTAAATGCACATGGCTGCAATTACATATGCCACCGACCGATCCCAACGTAAAATTGTTTACGCCTGTCACGGTACAAACCGTGGTCTCATTATGTTTAGTTCCATCCGTAAGCTTCCAAGTTGACGTAAGATCGTGTTTTAGATTTGTATTTTCACCAACCTTTATACTAGAACTTTTAAAACTTTCAACTTCAAGGCATAATGCCAAAATTAAGCACGTTATTAGTCCCCCCCCCCC---------ACCGAGGAATGTGACTGGACCGGTTCTTAGCAGCTTTGGGAGCCAT

PAV32 CTACGATTGACCAACAGAGACCAACCATGTCTCTTTAACCTCGCTTGGGCCGCTACCGTCTTTCCAAACCAGATCGTAACCTCCTTCATTTACAGACGTAACGTTACACAGCGTTAAATGCACATGGCTGCAATTACATATGCCACCGACCGATCCCAACGTAAAATTGTTTACGCCTGTCACGGTACAAACCGTGGTCTCATTATGTTTAGTTCCATCCGTAAGCTTCCAAGTTGACGTAAGATCGTGTTTTAGATTTGTATTTTCACCAACCTTTATACTAGAACTTTTAAAACTTTCAACTTCAAGGCATAATGCCAAAATTAAGCACGTTATTAGTCCCCCCCCCCC---------ACCGAGGAATGTGACTGGACCGGTTCTTAGCAGCTTTGGGAGCCAT

HANSCTR10 CTACGATTGACCAACAGAGACCAACCATGTCTCTTTAACCTCGCTTGGGCCGCTACCGTCTTTCCAAACCAGATCGTAACCTCCTTCATTTACAGACGTAACGTTACACAGCGTTAAATGCACATGGCTGCAATTACATATGCCACCGACCGATCCCAACGTAAAATTGTTTACGCCTGTCACGGTACAAACCGTGGTCTCATTATGTTTAGTTCCATCCGTAAGCTTCCAAGTTGACGTAAGATCGTGTTTTAGATTTGTATTTTCACCAACCTTTATACTAGAACTTTTAAAACTTTCAACTTCAAGGCATAATGCCAAAATTAAGCACGTTATTAGTCCCCCCCCCCC---------ACCGAGGAATGTGACTGGACCGGTTCTTAGCAGCTTTGGGAGCCAT

BE/14/2012 CTACGATTGACCAACAGAGACCAACCATGTCTCTTTAACCTCGCTTGGGCCGCTACCGTCTTTCCAAACCAGATCGTAACCTCCTTCATTTACAGACGTAACGTTACACAGCGTTAAATGCACATGACTGCAATTACATATGCCACCGACCGATCCCAACGTAAAATTGTTTACGCCTGTCACGGTACAAACCGTGGTCTCATTATGTTTAGTTCCATCCGTAAGCTTCCAAGTTGACGTAAGATCGTGTTTTAGATTTGTATTTTCACCAGCCTTTATACTAGAACTTTTAAAACTTTCAACTTCAAGGCATAATGCCAAAATTAAGCACGTTATTAGTCCCCCCCCCCCC--------ACCGAGGAATGTGACTGGACCGGTTCTTAGCAGCTTTGGGAGCCAT

HAN36 ------TTAACCAACAGAGACCAACCATGTCTCTTTAACCTCGCTTGGGCCGCTACCGTCTTTCCAAACCAGATCGTAACCTCCTTCATTTACAGACGTAACGTTACACAGCGTTAAATGCACATGGCTGCAATTACATATGCCACCGACCGATCCCAACGTAAAATTGTTTACGCCTGTCACGGTACAAACCGTGGTCTCATTATGTTTAGTTCCATCCGTAAGCTTCCAAGTTGACGTAAGATCGTGTTTTAGATTTGTATTTTCACCAACCTTTATACTAGAACTTTTAAAACTTTCAACTTCAAGGCATAATGCCAAAATTAAGCACGTTATTAGTCCCCCCCCCCCC--------ACCGAGGAATGTGACTGGACCGGTTCTTAGCAGCTTTGGGAGCCAT

HAN17 CTACGATTGACCAACAGAGACCAACCATGTCTCTTTAACCTCGCTTGGGCCGCTACCGTCTTTCCAAACCAGATCGTAACCTCCTTCATTTACAGACGTAACGTTACACAGCGTTAAATGCACATGGCTGCAATTACATATGCCACCGACCGATCCCAACGTAAAATTGTTTACGCCTGTCACGGTACAAACCGTGGTCTCATTATGTTTAGTTCCATCCGTAAGCTTCCAAGTTGACGTAAGATCGTGTTTTAGATTTGTATTTTCACCAACCTTTATACTAGAACTTTTAAAACTTTCAACTTCAAGGCATAATGCCAAAATTAAGCACGTTATTAGTCCCCCCCCCCCC--------ACCGAGGAATGTGACTGGACCGGTTCTTAGCAGCTTTGGGAGCCAT

HANRTR8 CTACGATTGACCAACAGAGACCAACCATGTCTCTTTAACCTCGCTTGGGCCGCTACCGTCTTTCCAAACCAGATCGTAACCTCCTTCATTTACAGACGTAACGTTACACAGCGTTAAATGCACATGGCTGCAATTACATATGCCACCGACCGATCCCAACGTAAAATTGTTTACGCCTGTCACGGTACAAACCGTGGTCTCATTATGTTTAGTTCCATCCGTAAGCTTCCAAGTTGACGTAAGATCGTGTTTTAGATTTGTATTTTCACCAACCTTTATACTAGAACTTTTAAAACTTTCAACTTCAAGGCATAATGCCAAAATTAAGCACGTTATTAGTCCCCCCCCCCCC--------ACCGAGGAATGTGACTGGACCGGTTCTTAGCAGCTTTGGGAGCCAT

JER2282 CTACGATTGACCAACAGAGACCAACCATGTCTCTTTAACCTCGCTTGGGCCGCTACCGTCTTTCCAAACCAGATCGTAACCTCCTTCATTTACAGACGTAACGTTACACAGCGTTAAATGCACATGACTGCAATTACATATGCCACCGACCGATCCCAACGTAAAATTGTTTACGCCTGTCACGGTACAAACCGTGGTCTCATTATGTTTAGTTCCATCCGTAAGCTTCCAAGTTGACGTAAGATCGTGTTTTAGATTTGTATTTTCACCAGCCTTTATACTAGAACTTTTAAAACTTTCAACTTCAAGGCATAATGCCAAAATTAAGCACGTTATTAGTCCCCCCCCCCCCAC------ACCGAGGAATGTGACTGGACCGGTTCTTAGCAGCTTTGGGAGCCAT

TB40/E ------TTAACCAACAGAGACCAACCATGTCTCTTTAACCTCGCTTGGGCCGCTACCGTCTTTCCAAACCAGATCGTAACCTCCTTCATTTACAGACGTAACGTTACACAGCGTTAAATGCACATGGCTGCAATTACATATGCCACCGACCGATCCCAACGTAAAATTGTTTACGCCTGTCACGGTACAAACCGTGGTCTCATTATGTTTAGTTCCATCCGTAAGCTTCCAAGTTGACGTAAGATCGTGTTTTAGATTTGTATTTTCACCAACCTTTATACTAGAACTTTTAAAACTTTCAACTTCAAGGCATAATGCCAAAATTAAGCACGTTATTAGTCCCCCCCCCCCCCC------ACCGAGGAATGTGACTGGACCGGTTCTTAGCAGCTTTGGGAGCCAT

BE/48/2011 G5 ------TTAAGAAACCGAAACCACCCATAGTTCACCATCCTCTTCATCATTCAACCGATGACCCACTCCGTACAACGACTCAGTCTGCTTCGTCATATTGCAAAGCACAAGCGACGTATGTGAACAACTTGAAACACAGACTGTGTTATTAATGACCGTTGTACCATTACTAGTCACATTGCATAAAGATCCTCCGCCGTCATCCCATCTTTTCCACTCGGTGGAAAACCGATCGCTATCATCAACTATGGTGAGATTTTCACCCTGCGTGGTATTCAGTTTCTTCATATTCATACCTTGGATTCCATTATTAAACCCCAATATTAAGCACGTTATTAGTACCCCCCCCCC---------ACCAAGGAATGTGACTGGACCGGTTCCTAGCAGCTTTGGGAGCCAT

NL/Rot4 ------TTAAGAAACCGAAACCACCCATAGTTCACCATCCTCTTCATCATTCAACCGATGACCCACTCCGTACAACGACTCAGTCTGCTTCGTCATATTGCAAAGCACAAGCGACGTATGTGAACAACTTGAAACACAGACTGTGTTATTAATGACCGTTGTACCATTACTAGTCACATTGCATAAAGATCCTCCGCCGTCATCCCATCTTTTCCACTCGGTGGAAAACCGATCGCTATCATCAACTATGGTGAGATTTTCACCCTGCGTGGTATTCAGTTTCTTCATATTCATACCTTGGATTCCATTATTAAACCCCAATATTAAGCACGTTATTAGTACCCCCCCCCC---------ACCAAGGAATGTGACTGGACCGGTTCCTAGCAGCTTTGGGAGCCAT

BE/35/2011 ------TTAAGAAACCGAAACCACCCATAGTTCACCATCCTCTTCATCATTCAACCGATGACCCACTCCGTACAACGACTCAGTCTGCTTCGTCATATTGCAAAGCACAAGCGACGTATGTGAACAACTTGAAACACAGACTGTGTTATTAATGACCGTTGTACCATTACTAGTCACATTGCATAAAGATCCTCCGCCGTCGTCCCATCTTTTCCACTCGGTGGAAAACCGGTCGCTATCATCAACTATGGTGAGATTTTCACCCTGCGTGGTATTCAGTTTCTTCATATTCATACCTTGGATTCCATTATTAAACCCCAATATTAAGCACGTTATTAGTACCCCCCCCCC---------ACCAAGGAATGTGACTGGACCGGTTCCTAGCAGCTTTGGGAGCCAT

BE/19/2010 ------TTAAGAAACCGAAACCACCCATAGTTCACCATCCTCTTCATCATTCAACCGATGACCCACTCCGTACAACGACTCAGTCTGCTTCGTCATATTGCAAAGCACAAGCGACGTATGTGAACAACTTGAAACACAGACTGTGTTATTAATGACCGTTGTACCATTACTAGTCACATTGCATAAAGATCCTCCGCCGTCGTCCCATCTTTTCCACTCGGTGGAAAACCGGTCGCTATCATCAACTATGGTGAGATTTTCACCCTGCGTGGTATTCAGTTTCTTCATATTCATACCTTGGATTCCATTATTAAACCCCAATATTAAGCACGTTATTAGTACCCCCCCCCC---------ACCAAGGAATGTGACTGGACCGGTTCCTAGCAGCTCTAGGAGCCAT

PAV6 ------TTAAGAAACCGAAACCACCCATAGTTCACCATCCTCTTCATCATTCAACCGATGACCCACTCCGTACAACGACTCAGTCTGCTTCGTCATATTGCAAAGCACAAGCGACGTATGTGAACAACTTGAAACACAGACTGTGTTATTAATGACCGTTGTACCATTACTAGTCACATTGCATAAAGATCCTCCGCCGTCGTCCCATCTTTTCCACTCGGTGGAAAACCGGTCGCTATCATCAACTATGGTGAGATTTTCACCCTGCGTGGTATTCAGTTTCTTCATATTCATACCTTGGATTCCATTATTAAACCCCAATATTAAGCACGTTATTAGTACCCCCCCCCC---------ACCAAGGAATGTGACTGGACCGGTTCCTAGCAGCTCTGGGAGCCAT

BE/18/2011 ------TTAAGAAACCGAAACCACCCATAGTTCACCATCCTCTTCATCATTCAACCGATGACCCACTCCGTACAACGACTCAGTCTGCTTCGTCATATTGCAAAGCACAAGCGACGTATGTGAACAACTTGAAACACAGACTGTGTTATTAATGACCGTTGTACCATTACTAGTCACATTGCATAAAGATCCTCCGCCGTCGTCCCATCTTTTCCACTCGGTGGAAAACCGGTCGCTATCATCAACTATGGTGAGATTTTCACCCTGCGTGGTATTCAGTTTCTTCATATTCATACCTTGGATTCCATTATTAAACCCCAATATTAAGCACGTTATTAGTACCCCCCCCCC---------ACCAAGGAATGTGACTGGACCGGTTCCTAGCAGCTCTGGGAGCCAT

BE/27/2011 ------TTAAGAAACCGAAACCACCCATAGTTCACCATCCTCTTCATCATTCAACCGATGACCCACTCCGTACAACGACTCAGTCTGCTTCGTCATATTGCAAAGCACAAGCGACGTATGTGAACAACTTGAAACACAGACTGTGTTATTAATGACCGTTGTACCATTACTAGTCACATTGCATAAAGATCCTCCGCCGTCGTCCCATCTTTTCCACTCGGTGGAAAACCGGTCGCTATCATCAACTATGGTGAGATTTTCACCCTGCGTGGTATTCAGTTTCTTCATATTCATACCTTGGATTCCATTATTAAACCCCAATATTAAGCACGTTATTAGTACCCCCCCCCC---------ACCAAGGAATGTGACTGGACCGGTTCCTAGCAGCTCTGGGAGCCAT

BE/23/2011 ------TTAAGAAACCGAAACCACCCATAGTTCACCATCCTCTTCATCATTCAACCGATGACCCACTCCGTACAACGACTCAGTCTGCTTCGTCATATTGCAAAGCACAAGCGACGTATGTGAACAACTTGAAACACAGACTGTGTTATTAATGACCGTTGTACCATTACTAGTCACATTGCATAAAGATCCTCCGCCGTCGTCCCATCTTTTCCACTCGGTGGAAAACCGGTCGCTATCATCAACTATGGTGAGATTTTCACCCTGCGTGGTATTCAGTTTCTTCATATTCATACCTTGGATTCCATTATTAAACCCCAATATTAAGCACGTTATTAGTACCCCCCCCCC---------ACCAAGGAATGTGACTGGACCGGTTCCTAGCAGCTCTGGGAGCCAT

HAN27 ------TTAAGAAACCGAAACCACCCATAGTTCACCATCCTCTTCATCATTCAACCGATGACCCACTCCGTACAACGACTCAGTCTGCTTCGTCATATTGCAAAGCACAAGCGACGTATGTGAACAACTTGAAACACAGACTGTGTTATTAATGACCGTTGTACCATTACTAGTCACATTGCATAAAGATCCTCCGCCGTCGTCCCATCTTTTCCACTCGGTGGAAAACCGGTCGCTATCATCAACTATGGTGAGATTTTCACCCTGCGTGGTATTCAGTTTCTTCATATTCATACCTTGGATTCCATTATTAAACCCCAATATTAAGCACGTTATTAGTACCCCCCCCCC---------ACCAAGGAATGTGACTGGACCGGTTCCTAGCAGCTCTGGGAGCCAT

JER3230 ------TTAAGAAACCGAAACCACCCATAGTTCACCATCCTCTTCATCATTCAACCGATGACCCACTCCGTACAACGACTCAGTCTGCTTCGTCATATTGCAAAGCACAAGCGACGTATGTGAACAACTTGAAACACAGACTGTGTTATTAATGACCGTTGTACCATTACTAGTCACATTGCATAAAGATCCTCCGCCGTCGTCCCATCTTTTCCACTCGGTGGAAAACCGGTCGCTATCATCAACTATGGTGAGATTTTCACCCTGCGTGGTATTCAGTTTCTTCATATTCATACCTTGGATTCCATTATTAAACCCCAATATTAAGCACGTTATTAGTACCCCCCCCCC---------ACCAAGGAATGTGACTGGACCGGTTCCTAGCAGCTCTGGGAGCCAT

BE/33/2011 ------TTAAGAAACCGAAACCACCCATAGTTCACCATCCTCTTCATCATTCAACCGATGACCCACTCCGTACAACGACTCAGTCTGCTTCGTCATATTGCAAAGCACAAGCGACGTATGTGAACAACTTGAAACACAGACTGTGTTATTAATGACCGTTGTACCATTACTAGTCACATTGCATAAAGATCCTCCGCCGTCGTCCCATCTTTTCCACTCGGTGGAAAACCGGTCGCTATCATCAACTATGGTGAGATTTTCACCCTGCGTGGTATTCAGTTTCTTCATATTCATACCTTGGATTCCATTATTAAACCCCAATATTAAGCACGTTATTAGTACCCCCCCCCC---------ACCAAGGAATGTGACTGGACCGGTTCCTAGCAGCTCTGGGAGCCAT

NAN2LA ------TTAAGAAACCGAAACCACCCATAGTTCACCATCCTCTTCATCATTCAACCGATGACCCACTCCGTACAACGACTCAGTCTGCTTCGTCATATTGCAAAGCACAAGCGACGTATGTGAACAACTTGAAACACAGACTGTGTTATTAATGACCGTTGTACCATTACTAGTCACATTGCATAAAGATCCTCCGCCGTCGTCCCATCTTTTCCACTCGGTGGAAAACCGGTCGCTATCATCAACTATGGTGAGATTTTCACCCTGCGTGGTATTCAGTTTCTTCATATTCATACCTTGGATTCCATTATTAAACCCCAATATTAAGCACGTTATTAGTACCCCCCCCCC---------ACCAAGGAATGTGACTGGACCGGTTCCTAGCAGCTCTGGGAGCCAT

PAV26 ------TTAAGAAACCGAAACCACCCATAGTTCACCATCCTCTTCATCATTCAACCGATGACCCACTCCGTACAACGACTCAGTCTGCTTCGTCATATTGCAAAGCACAAGCGACGTATGTGAACAACTTGAAACACAGACTGTGTTATTAATGACCGTTGTACCATTACTAGTCACATTGCATAAAGATCCTCCGCCGTCGTCCCATCTTTTCCACTCGGTGGAAAACCGGTCGCTATCATCAACTATGGTGAGATTTTCACCCTGCGTGGTATTCAGTTTCTTCATATTCATACCTTGGATTCCATTATTAAACCCCAATATTAAGCACGTTATTAGTACCCCCCCCCC---------ACCAAGGAATGTGACTGGACCGGTTCCTAGCAGCTCTGGGAGCCAT

BE/5/2012 ------TTAAGAAACCGAAACCACCCATAGTTCACCATCCTCTTCATCATTCAGCCGATGACCCACTCCGTACAACGACTCAGTCTGCTTCGTCATATTGCAAAGCACAAGCGACGTATGTGAACAACTTGAAACACAGACTGTGTTATTAATGACCGTTGTACCATTACTAGTCACATTGCATAAAGATCCTCCGCCGTCGTCCCATCTTTTCCACTCGGTGGAAAACCGGTCGCTATCATCAACTATGGTGAGATTTTCACCCTGCGTGGTATTCAGTTTCTTCATATTCATACCTTGGATTCCATTATTAAACCCCAATATTAAGCACGTTATTAGTACCCCCCCCCC---------ACCAAGGAATGTGACTGGACCGGTTCCTAGCAGCTCTGGGAGCCAT

UK/Lon6 ------TTAAGAAACCGAAACCACCCATAGTTCACCATCCTCTTCATCATTCAGCCGATGACCCACTCCGTACAACGACTCAGTCTGCTTCGTCATATTGCAAAGCACAAGCGACGTATGTGAACAACTTGAAACACAGACTGTGTTATTAATGACCGTTGTACCATTACTAGTCACATTGCATAAAGATCCTCCGCCGTCGTCCCATCTTTTCCACTCGGTGGAAAACCGGTCGCTATCATCAACTATGGTGAGATTTTCACCCTGCGTGGTATTCAGTTTCTTCATATTCATACCTTGGATTCCATTATTAAACCCCAATATTAAGCACGTTATTAGTACCCCCCCCCC---------ACCAAGGAATGTGACTGGACCGGTTCCTAGCAGCTCTGGGAGCCAT

BE/39/2011 ------TTAAGAAACCGAAACCACCCATAGTTCACCATCCTCTTCATCATTCAACCGATGACCCACTCCGTACAACGACTCAGTCTGCTTCGTCATATTGCAAAGCACAAGCGACGTATGTGAACAACTTGAAACACAGACTGTGTTATTAATGACCGTTGTACCATTACTAGTCACATTGCATAAAGATCCTCCGCCGTCATCCCATCTTTTCCACTCGGTGGAAAACCGATCGCTATCATCAACTATGGTGAGATTTTCACCCTGCGTGGTATTCAGTTTCTTCATATTCATACCTTGGATTCCATTATTAAACCCCAATATTAAGCACGTTATTAGTACCCCCCCCCCACC------ACCAAGGAATGTGACTGGACCGGTTCCTAGCAGCTTTGGGAGCCAT

JER4053 ------TTAAGAAACCGAAACCACCCATAGTTCACCATCCTCTTCATCATTCAACCGATGACCCACTCCGTACAACGACTCAGTCTGCTTCGTCATATTGCAAAGCACAAGCGACGTATGTGAACAACTTGAAACACAGACTGTGTTATTAATGACCGTTGTACCATTACTAGTCACATTGCATAAAGATCCTCCGCCGTCATCCCATCTTTTCCACTCGGTGGAAAACCGATCGCTATCATCAACTATGGTGAGATTTTCACCCTGCGTGGTATTCAGTTTCTTCATATTCATACCTTGGATTCCATTATTAAACCCCAATATTAAGCACGTTATTAGTACCCCCCCCCCC--------ACCAAGGAATGTGACTGGACCGGTTCCTAGCAGCTTTGGGAGCCAT

JER1289 ------TTAAGAAACCGAAACCACCCATAGTTCACCATCCTCTTCATCATTCAACCGATGACCCACTCCGTACAACGACTCAGTCTGCTTCGTCATATTGCAAAGCACAAGCGACGTATGTGAACAACTTGAAACACAGACTGTGTTATTAATGACCGTTGTACCATTACTAGTCACATTGCATAAAGATCCTCCGCCGTCATCCCATCTTTTCCACTCGGTGGAAAACCGATCGCTATCATCAACTATGGTGAGATTTTCACCCTGCGTGGTATTCAGTTTCTTCATATTCATACCTTGGATTCCATTATTAAACCCCAATATTAAGCACGTTATTAGTACCCCCCCCCCC--------ACCAAGGAATGTGACTGGACCGGTTCCTAGCAGCTTTGGGAGCCAT

2CEN15 ------TTAAGAAACCGAAACCACCCATAGTTCACCATCCTCTTCATCATTCAGCCGATGACCCACTCCGTACAACGACTCAGTCTGCTTCGTCATATTGCAAAGCACAAGCGACGTATGTGAACAACTTGAAACACAGACTGTGTTATTAATGACCGTTGTACCATTACTAGTCACATTGCATAAAGATCCTCCGCCGTCGTCCCATCTTTTCCACTCGGTGGAAAACCGGTCGCTATCATCAACTATGGTGAGATTTTCACCCTGCGTGGTATTCAGTTTCTTCATATTCATACCTTGGATTCCATTATTAAACCCCAATATTAAGCACGTTATTAGTACCCCCCCCCCC--------ACCAAGGAATGTGACTGGACCGGTTCCTAGCAGCTCTGGGAGCCAT

TR ------TTAAGAAACCGAAACCACCCATAGTTCACCATCCTCTTCATCATTCAGCCGATGACCCACTCCGTACAACGACTCAGTCTGCTTCGTCATATTGCAAAGCACAAGCGACGTATGTGAACAACTTGAAACACAGACTGTGTTATTAATGACCGTTGTACCATTACTAGTCACATTGCATAAAGATCCTCCGCCGTCGTCCCATCTTTTCCACTCGGTGGAAAACCGGTCGCTATCATCAACTATGGTGAGATTTTCACCCTGCGTGGTATTCAGTTTCTTCATATTCATACCTTGGATTCCATTATTAAACCCCAATATTAAGCACGTTATTAGTACCCCCCCCCCC--------ACCAAGGAATGTGACTGGACCGGTTCCTAGCAGCTCTGGGAGCCAT

W ------TTAAGAAACCGAAACCACCCATAGTTCACCATCCTCTTCATCATTCAGCCGATGACCCACTCCGTACAACGACTCAGTCTGCTTCGTCATATTGCAAAGCACAAGCGACGTATGTGAACAACTTGAAACACAGACTGTGTTATTAATGACCGTTGTACCATTACTAGTCACATTGCATAAAGATCCTCCGCCGTCGTCCCATCTTTTCCACTCGGTGGAAAACCGGTCGCTATCATCAACTATGGTGAGATTTTCACCCTGCGTGGTATTCAGTTTCTTCATATTCATACCTTGGATTCCATTATTAAACCCCAATATTAAGCACGTTATTAGTACCCCCCCCCCC--------ACCAAGGAATGTGACTGGACCGGTTCCTAGCAGCTCTGGGAGCCAT

HAN20 ------TTAAGAAACCGAAACCACCCATAGTTCACCATCCTCTTCATCATTCAACCGATGACCCACTCCGTACAACGACTCAGTCTGCTTCGTCATATTGCAAAGCACAAGCGACGTATGTGAACAACTTGAAACACAGACTGTGTTATTAATGACCGTTGTACCATTACTAGTCACATTGCATAAAGATCCTCCACCGTCGTCCCATCTTTTCCACTCGGTGGAAAACCGGTCGCTATCATCAACTATGGTGAGATTTTCACCCTGCGTGGTATTCAGTTTCTTCATATTCATACCTTGGATTCCATTATTAAACCCCAATATTAAGCACGTTATTAGTACCCCCCCCCCC--------ACCAAGGAATGTGACTGGACCGGTTCCTAGCAGCTCTGGGAGCCAT

JER1070 ------TTAAGAAACCGAAACCACCCATAGTTCACCATCCTCTTCATCATTCAACCGATGACCCACTCCGTACAACGACTCAGTCTGCTTCGTCATATTGCAAAGCACAAGCGACGTATGTGAACAACTTGAAACACAGACTGTGTTATTAATGACCGTTGTACCATTACTAGTCACATTGCATAAAGATCCTCCGCCGTCGTCCCATCTTTTCCACTCGGTGGAAAACCGGTCGCTATCATCAACTATGGTGAGATTTTCACCCTGCGTGGTATTCAGTTTCTTCATATTCATACCTTGGATTCCATTATTAAACCCCAATATTAAGCACGTTATTAGTACCCCCCCCCCC--------ACCAAGGAATGTGACTGGACCGGTTCCTAGCAGCTCTGGGAGCCAT

BE/6/2011 ------TTAAGAAACCGAAACCACCCATAGTTCACCATCCTCTTCATCATTCAACCGATGACCCACTCCGTACAACGACTCAGTCTGCTTCGTCATATTGCAAAGCACAAGCGACGTATGTGAACAACTTGAAACACAGACTGTGTTATTAATGACCGTTGTACCATTACTAGTCACATTGCATAAAGATCCTCCGCCGTCGTCCCATCTTTTCCACTCGGTGGAAAACCGGTCGCTATCATCAACTATGGTGAGATTTTCACCCTGCGTGGTATTCAGTTTCTTCATATTCATACCTTGGATTCCATTATTAAACCCCAATATTAAGCACGTTATTAGTACCCCCCCCCCC--------ACCAAGGAATGTGACTGGACCGGTTCCTAGCAGCTCTGGGAGCCAT

DB G6 ------TTAAGAAACCGAAACTCTCCACAGTTCACCATCTTCTTCGTCATTCAATCGATGACCCACTCCGTACAACGAATCAGTCTGCTGCGTCATATTGCAAAGCACAAGCGACGTATGCGAACAACTTGAAACACAGACTGTGGTATTAACGACCGTTGTACCATTACTAGTCACATTGCATAGAGACCCTCCATCGTTATCCCATCTTTTCCATTCAGTGGAAAACCGGCCGCTATCATCAACTATAGTAAGATTTCCACCCTGCGTGGTATTCAGTTTCTTCATATCCATACCCTGGATTCCATCATTAAACCCCAATATTAAACACTTTTTTAGTACCCCCCACCC---------ACCAAAAAATGTGACTGGACCGGTTCCTAGCAGCTCTGGGAGCCAT

BE/26/2011 ------TTAAGAAACCGAAACTCTCCACAGTTCACCATCTTCTTCGTCATTCAATCGATGACCCACTCCGTACAACGAATCAGTCTGCTGCGTCATATTGCAAAGCACAAGCGACGTATGCGAACAACTTGAAACACAGACTGTGGTATTAACGACCGTTGTACCATTACTAGTCACATTGCATAGAGACCCTCCATCGTTATCCCATCTTTTCCATTCAGTGGAAAACCGGCCGCTATCATCAACTATAGTAAGATTTCCACCCTGCGTGGTATTCAGTTTCTTCATATCCATACCCTGGATTCCATCATTAAACCCCAATATTAAGCACGTTATTAGTACCCCCCCCC----------ACCAAGGAATGTGACTGGACCGGTTCTTAGCAGCTTTGGGAGCCAT

HANSCTR13 ------TTAAGAAACCGAAACTCTCCACAGTTCACCATCTTCTTCGTCATTCAACCTATGACCCACTCCGTACAACGAATCAGTCTGCTGCGTCATATTGCAAAGCACAAGCGACGTATGCGAACAACTTGAAACACAGACTGTGGTATTAACGACCGTTGTACCATTACTAGTCACATTGCATAGAGACCCTCCACCGTTATCCCATCTTTTCCATTCAGTGGAAAACCGGCCGCTATCATCAACTATAGTAAGATTTTCACCCTGCGTGGTATTCAGTTTCTTCATATCCATACCCTGGATTCCATCATTAAACCCCAATATTAAGCACGTTATTAGTACCCCCCCCCC---------ACCAAGGAATGTGACTGGACCGGTTCTTAGCAGCTTTGGGAGCCAT

BE/41/2011 ------TTAAGAAACCGAAACTCTCCACAGTTCACCATCTTCTTCGTCATTCAACCGATGACCCACTCCGTACAACGAATCAGTCTGCTGCGTCATATTGCAAAGCACAAGCGACGTATGCGAACAACTTGAAACACAGACTGTGGTATTAACGACCGTTGTACCATTACTAGTCACATTGCATAGAGACCCTCCATCGTCATCCCATCTTTTCCATTCAGTGGAAAACCGGCCGCTATCATCAACTATAGTAAGATTTCCACCCTGCGTGGTATTCAGTTTCTTCATATCCATACCCTGGATTCCATCATTAAACCCCAATATTAAGCACGTTATTAGTACCCCCCCCCC---------ACCAAGGAATGTGACTGGACCGGTTCTTAGCAGCTTTAGGAGCCAT

PH ------TTAAGAAACCGAAACTCTCCACATTTCACCATCTTCTTCGTCATTCAACCGATGACCCACTCCGTACAACGAATCAGTCTGCTGCGTCATATTGCAAAGCACAAGCGACGTATGCGAACAACTTGAAACACAGACTGTGGTATTAACGACCGTTGTACCATTACTAGTCACATTGCATAGAGACCCTCCATCGTTATCCCATCTTTTCCATTCAGTGGAAAACCGGCCGCTATCATCAACTATAGTAAGATTTCCACCCTGCGTGGTATTCAGTTTCTTCATATCCATACCCTGGATTCCATCATTAAACCCCAATATTAAGCACGTTATTAGTACCCCCCCCCC---------ACCAAGGAATGTGACTGGACCGGTTCTTAGCAGCTTTGGGAGCCAT

NL/Rot1 ------TTAAGAAACCGAAACTCTCCACATTTCACCATCTTCTTCGTCATTCAACCGATGACCCACTCCGTACAACGAATCAGTCTGCTGCGTCATATTGCAAAGCACAAGCGACGTATGCGAACAACTTGAAACACAGACTGTGGTATTAACGACCGTTGTACCATTACTAGTCACATTGCATAGAGACCCTCCATCGTTATCCCATCTTTTCCATTCAGTGGAAAACCGGCCGCTATCATCAACTATAGTAAGATTTCCACCCTGCGTGGTATTCAGTTTCTTCATATCCATACCCTGGATTCCATCATTAAACCCCAATATTAAGCACGTTATTAGTACCCCCCCCCC---------ACCAAGGAATGTGACTGGACCGGTTCTTAGCAGCTTTGGGAGCCAT

PAV7 ------TTAAGAAACCGAAACTCTCCACAGTTCACCATCGTCTTCGTCATTCAACCGATGACCCACTCCGTACAACGAATCAGTCTGCTGCGTCATATTGCAAAGCACAAGCGACGTATGCGAACAACTTGAAACACAGACTGTGGTATTAACGACCGTTGTACCATTACTAGTCACATTGCATAGAGACCCTCCATCGTTATCCCATCTTTTCCATTCAGTGGAAAACCGGCCGCTATCATCAACTATAGTAAGATTTCCACCCTGCGTGGTATTCAGTTTCTTCATATCCATACCCTGGATTCCATCATTAAACCCCAATATTAAGCACGTTATTAGTACCCCCCCCCC---------ACCAAGGAATGTGACTGGACCGGTTCTTAGCAGCTTTGGGAGCCAT

BE/10/2011 ------TTAAGAAACCGAAACTCTCCACAGTTCACCATCTTCTTCGTCATTCAATCGATGACCCACTCCGTACAACGAATCAGTCTGCTGCGTCATATTGCAAAGCACAAGCGACGTATGCGAACAACTTGAAACACAGACTGTGGTATTAACGACCGTTGTACCATTACTAGTCACATTGCATAGAGACCCTCCATCGTTATCCCATCTTTTCCATTCAGTGGAAAACCGGCCGCTATCATCAACTATAGTAAGATTTCCACCCTGCGTGGTATTCAGTTTCTTCATATCCATACCCTGGATTCCATCATTAAACCCCAATATTAAGCACGTTATTAGTACCCCCCCCCC---------ACCAAGGAATGTGACTGGACCGGTTCTTAGCAGCTTTGGGAGCCAT

BE/20/2011 ------TTAAGAAACCGAAACTCTCCACAGTTCACCATCTTCTTCGTCATTCAATCGATGACCCACTCCGTACAACGAATCAGTCTGCTGCGTCATATTGCAAAGCACAAGCGACGTATGCGAACAACTTGAAACACAGACTGTGGTATTAACGACCGTTGTACCATTACTAGTCACATTGCATAGAGACCCTCCATCGTTATCCCATCTTTTCCATTCAGTGGAAAACCGGCCGCTATCATCAACTATAGTAAGATTTCCACCCTGCGTGGTATTCAGTTTCTTCATATCCATACCCTGGATTCCATCATTAAACCCCAATATTAAGCACGTTATTAGTACCCCCCCCCC---------ACCAAGGAATGTGACTGGACCGGTTCTTAGCAGCTTTGGGAGCCAT

BE/9/2010 ------TTAAGAAACCGAAACTCTCCACAGTTCACCATCTTCTTCGTCATTCAATCGATGACCCACTCCGTACAACGAATCAGTCTGCTGCGTCATATTGCAAAGCACAAGCGACGTATGCGAACAACTTGAAACACAGACTGTGGTATTAACGACCGTTGTACCATTACTAGTCACATTGCATAGAGACCCTCCATCGTTATCCCATCTTTTCCATTCAGTGGAAAACCGGCCGCTATCATCAACTATAGTAAGATTTCCACCCTGCGTGGTATTCAGTTTCTTCATATCCATACCCTGGATTCCATCATTAAACCCCAATATTAAGCACGTTATTAGTACCCCCCCCCC---------ACCAAGGAATGTGACTGGACCGGTTCTTAGCAGCTTTGGGAGCCAT

UK/Lon9 ------TTAAGAAACCGAAACTCTCCACAGTTCACCATCTTCTTCGTCATTCAATCGATGACCCACTCCGTACAACGAATCAGTCTGCTGCGTCATATTGCAAAGCACAAGCGACGTATGCGAACAACTTGAAACACAGACTGTGGTATTAACGACCGTTGTACCATTACTAGTCACATTGCATAGAGACCCTCCATCGTTATCCCATCTTTTCCATTCAGTGGAAAACCGGCCGCTATCATCAACTATAGTAAGATTTCCACCCTGCGTGGTATTCAGTTTCTTCATATCCATACCCTGGATTCCATCATTAAACCCCAATATTAAGCACGTTATTAGTACCCCCCCCCC---------ACCAAGGAATGTGACTGGACCGGTTCTTAGCAGCTTTGGGAGCCAT

UK/Lon2 ------TTAAGAAACCGAAACTCTCCACAGTTCACCATCTTCTTCGTCATTCAACCGATGACCCACTCCGTACAACGAATCAGTCTGCTGCGTCATATTGCAAAGCACAAGCGACGTATGCGAACAACTTGAAACACAGACTGTGGTATTAACGACCGTTGTACCATTACTAGTCACATTGCATAGAGACCCTCCATCGTTATCCCATCTTTTCCATTCAGTGGAAAACCGGCCGCTATCATCAACTATAGTAAGATTTCCACCCTGCGTGGTATTCAGTTTCTTCATATCCATACCCTGGATTCCATCATTAAACCCCAATATTAAGCACGTTATTAGTACCCCCCCCCC---------ACCAAGGAATGTGACTGGACCGGTTCTTAGCAGCTTTGGGAGCCAT

JHC ------TTAAGAAACCGAAACTCTCCACAGTTCACCATCTTCTTCGTCATTCAACCTATGACCCACTCCGTACAACGAATCAGTCTGCTGCGTCATATTGCAAAGCACAAGCGACGTATGCGAACAACTTGAAACACAGACTGTGGTATTAACGACCGTTGTACCATTACTAGTCACATTGCATAGAGACCCTCCACCGTTATCCCATCTTTTCCATTCAGTGGAAAACCGGCCGCTATCATCAACTATAGTAAGATTTCCACCCTGCGTGGTATTCAGTTTCTTCATATCCATACCCTGGATTCCATCATTAAACCCCAATATTAAGCACGTTATTAGTACCCCCCCCCCC--------ACCAAGGAATGTGACTGGACCGGTTCTTAGCAGCTTTGGGAGCCAT

BE/10/2012 ------TTAAGAAACCGAAACTCTCCACAGTTCACCATCTTCTTCGTCATTCAATCGATGACCCACTCCGTACAACGAATCAGTCTGCTGCGTCATATTGCAAAGCACAAGCGACGTATGCGAACAACTTGAAACACAGACTGTGGTATTAACGACCGTTGTACCATTACTAGTCACATTGCATAGAGACCCTCCATCGTTATCCCATCTTTTCCATTCAGTGGAAAACCGGCCGCTATCATCAACTATAGTAAGATTTCCACCCTGCGTGGTATTCAGTTTCTTCATATCCATACCCTGGATTCCATCATTAAACCCCAATATTAAGCACGTTATTAGTACCCCCCCCCCC--------ACCAAGGAATGTGACTGGACCGGTTCTTAGCAGCTTTGGGAGCCAT

BE/22/2010 ------TTAAGAAACCGAAACTCTCCACAGTTCACCATCTTCTTCGTCATTCAACCGATGACCCACTCCGTACAACGAATCAGTCTGCTGCGTCATATTGCAAAGCACAAGCGACGTATGCGAACAACTTGAAACACAGACTGTGGTATTAACGACCGTTGTACCATTACTAGTCACATTGCATAGAGACCCTCCATCGTTATCCCATCTTTTCCATTCAGTGGAAAACCGGCCGCTATCATCAACTATAGTAAGATTTCCACCCTGCGTGGTATTCAGTTTCTTCATATCCATACCCTGGATTCCATCATTAAACCCCAATATTAAGCACGTTATTAGTACCCCCCCCCCC--------ACCAAGGAATGTGACTGGACCGGTTCTTAGCAGCTTTGGGAGCCAT

CINCY ------TTAAGAAACCGAAACTCTCCACAGTTCACCATCTTCTTCGTCATTCAACCGATGACCCACTCCGTACAACGAATCAGTCTGCTGCGTCATATTGCAAAGCACAAGCGACGTATGCGAACAACTTGAAACACAGACTGTGGTATTAACGACCGTTGTACCATTACTAGTCACATTGCATAGAGACCCTCCATCGTTATCCCATCTTTTCCATTCAGTGGAAAACCGGCCGCTATCATCAACTATAGTAAGATTTCCACCCTGCGTGGTATTCAGTTTCTTCATATCCATACCCTGGATTCCATCATTAAACCCCAATATTAAGCACGTTATTAGTACCCCCCCCCCC--------ACCAAGGAATGTGACTGGACCGGTTCTTAGCAGCTTTGGGAGCCAT

HANRTR6 ------TTAAGAAACCGAAACTCTCCACAGTTCACCATCTTCTTCGTCATTCAACCGATGACCCACTCCGTACAACGAATCAGTCTGCTGCGTCATATTGCAAAGCACAAGCGACGTATGCGAACAACTTGAAACACAGACTGTGGTATTAACGACCGTTGTACCATTACTAGTCACATTGCATAGAGACCCTCCATCGTTATCCCATCTTTTCCATTCAGTGGAAAACCGGCCGCTATCATCAACTATAGTAAGATTTCCACCCTGCGTGGTATTCAGTTTCTTCATATCCATACCCTGGATTCCATCATTAAACCCCAATATTAAGCACGTTATTAGTACCCCCCCCCCC--------ACCAAGGAATGTGACTGGACCGGTTCTTAGCAGCTTTGGGAGCCAT

HANSCTR12 ------TTAAGAAACCGAAACTCTCCACAGTTCACCATCTTCTTCGTCATTCAACCTATGACCCACTCCGTACAACGAATCAGTCTGCTGCGTCATATTGCAAAGCACAAGCGACGTATGCGAACAACTTGAAACACAGACTGTGGTATTAACGACCGTTGTACCATTACTAGTCACATTGCATAGAGACCCTCCACCGTTATCCCATCTTTTCCATTCAGTGGAAAACCGGCCGCTATCATCAACTATAGTAAGATTTTCACCCTGCGTGGTATTCAGTTTCTTCATATCCATACCCTGGATTCCATCATTAAACCCCAATATTAAGCACGTTATTAGTACCCCCCCCCCCC-------ACCAAGGAATGTGACTGGACCGGTTCTTAGCAGCTTTGGGAGCCAT

3157 ------TTAAGAAACCGAAACTCTCCACATTTCACCATCTTCTTCGTCATTCAACCGATGACCCACTCCGTACAACGAATCAGTCTGCTGCGTCATATTGCAAAGCACAAGCGACGTATGCGAACAACTTGAAACACAGACTGTGGTATTAACGACCGTTGTACCATTACTAGTCACATTGCATAGAGACCCTCCATCGTTATCCCATCTTTTCCATTCAGTGGAAAACCGGCCGCTATCATCAACTATAGTAAGATTTCCACCCTGCGTGGTATTCAGTTTCTTCATATCCATACCCTGGATTCCATCATTAAACCCCAATATTAAGCACGTTATTAGTACCCCCCCCCCCC-------ACCAAGGAATGTGACTGGACCGGTTCTTAGCAGCTTTGGGAGCCAT

Pat_E ------TTAAGAAACCGAAACTCTCCACAGTTCACCATCTTCTTCGTCATTCAACCGATGACCCACTCCGTACAACGAATCAGTCTGCTGCGTCATATTGCAAAGCACAAGCGACGTATGCGAACAACTTGAAACACAGACTGTGGTATTAACGACCGTTGTACCATTACTAGTCACATTGCATAGAGACCCTCCATCGTTATCCCATCTTTTCCATTCAGTGGAAAACCGGCCGCTATCATCAACTATAGTAAGATTTCCACCCTGCGTGGTATTCAGTTTCTTCATATCCATACCCTGGATTCCATCATTAAACCCCAATATTAAGCACGTTATTAGTACCCCCCCCCCCC-------ACCAAGGAATGTGACTGGACCGGTTCTTAGCAGCTTTGGGAGCCAT

BE/11/2010 G1 ------TTAAGAAACCGAAACTCTCCACAGTTCACCATCTTCTTCGTCATCCAACCGATGACCCACTCCGTACAACGAATCGGTCTGCTGCGTCATATTGCAAAACACAAGCGACGTATGCGAACAACTTGAAACACAGAC-----------CGCGCGTTGTACCATTACTAGTCACATTGCATACAGACCTTCCACCGTCATCCCATTTTTCCCACCCGATGGAAAACCGTCTTCTATCATAAACTATGGTAAGATTTCGACCCTGCGAGGTATTCAGTTTCCCCATATCCATAACCTGGATTTTATCATTAAACCCCAATATTAAACACCTTTTTAGTATCCCCCACCC---------ACCAAAAAATGTGACTGGACCGGTTCCTAGTAGCTTTGGGAGCCAT

UKNEQAS1 ------TTAAGAAACCGAAACTCTCCACAGTTCACCATCTTCTTCGTCATTCAACCGATGACCCACTCCGTACAACGAATCAGTCTGCTGCGTCATATTGCAAAGCACAAGCGACGTATGCGAACAACTTGAAACACAGAC-----------CGCGCGTTGTACCATTACTAGTCACATTGCATACAGACCTTCCACCGTCATCCCATTTTTCCCACCCGATGGAAAACCGTCTTCTATCATAAACTATGGTAAGATTTCGACCCTGCGAGGTATTCAGTTTCCCCATATCCATAACCTGGATTTTATCATTAAACCCCAATATTAAACACCTTTTTAGTATCCCCCACCC---------ACCAAAAAATGTGACTGGACCGGTTCCTAGTAGCTTTGGGAGCCAT

UK/Lon7 ------TTAAGAAACCGAAACTCTCCACAGTTCACCATCTTCTTCGTCATTCAACCGATGACCCACTCCGTACAACGAATCAGTCTGCTGCGTCATATTGCAAAGCACAAGCGACGTATGCGAACAACTTGAAACACAGAC-----------CGCGCGTTGTACCATTACTAGTCACATTGCATACAGACCTTCCACCGTCATCCCATTTTTCCCACCCGATGGAAAACCGTCTTCTATCATAAACTATGGTAAGATTTCGACCCTGCGAGGTATTCAGTTTCCCCATATCCATAACCTGGATTTTATCATTAAACCCCAATATTAAACACCTTTTTAGTATCCCCCACCC---------ACCAAAAAATGTGACTGGACCGGTTCCTAGTAGCTTTGGGAGCCAT

BE/6/2010 ------TTAAGAAACCGAAACTCTCCACAGTTCACCATCTTCTTCGTCATTCAACCGATGACCCACTCCGTACAACGAATCAGCCTGCTGCGTCATATTGTAAAGCACAAGCGACGTATGCGAACAACTTGAAACACAGAC-----------CGAGCGTTGTACCATTACTAGTCACATTGCATACAGACCTTCCACCGTCATCCCATCTTTCCCACCCGATGGAAAACCGTCTTCTATCATCAACTATGGTAAGATTTCGACCCTGCGAGGTATTCAGTTTCCCCATATCCATAACCTGGATTTTATCATTAAACCCCAATATTAAACACCTTTTTAGTACCCCCCCACCC--------ACCAAAAAATGTGACTGGACCGGTTCCTAGTAGCTTTGGGAGCCAT

BE/42/2011 ------TTAAGAAACCGAAACTCTCCACAGTTCACCATCTTCTTCGTCATTCAACCGATGACCCACTCCGTACAACGAATCAGCCTGCTGCGTCATATTGTAAAGCACAAGCGACGTATGCGAACAACTTGAAACACAGAC-----------CGAGCGTTGTACCATTACTAGTCACATTGCATACAGACCTTCCACCGTCATCCCATCTTTCCCACCCGATGGAAAACCGTCTTCTATCATCAACTATGGTAAGATTTCGACCCTGCGAGGTATTCAGTTTCCCCATATCCATAACCTGGATTTTATCATTAAACCCCAATATTAAACACCTTTTTAGTACCCCCCCACCC--------ACCAAAAAATGTGACTGGACCGGTTCCTAGTAGCTTTGGGAGCCAT

HANSCTR1B ------TTAAGAAACCGAAACTCTCCACAGTTCACCATCTTCTTCGTCATTCAACCGATGACCCACTCCGTACAACGAATCAGCCTGCTGCGTCATATTGTAAAGCACAAGCGACGTATGCGAACAACTTGAAACACAGAC-----------CGAGCGTTGTACCATTACTAGTCACATTGCATACAGACCTTCCACCGTCATCCCATCTTTCCCACCCGATGGAAAACCGTCTTCTATCATCAACTATGGTAAGATTTCGACCCTGCGAGGTATTCAGTTTCCCCATATCCATAACCTGGATTTTATCATTAAACCCCAATATTAAACACCTTTTTAGTACCCCCCCACCC--------ACCAAAAAATGTGACTGGACCGGTTCCTAGTAGCTTTGGGAGCCAT

PAV4 ------TTAAGAAACCGAAACTCTCCACAGTTCACCATCTTCTTCGTCATTCAACCGATGACCCACTCCGTACAACGAATCAGCCTGCTGCGTCATATTGTAAAGCACAAGCGACGTATGCGAACAACTTGAAACACAGAC-----------CGAGCGTTGTACCATTACTAGTCACATTGCATACAGACCTTCCACCGTCATCCCATCTTTCCCACCCAATAAAAAACCATCTTCTATCATCAACTATGGTAAGATTTCGACCCTGCGAGGTATTCAGTTTCCCCATATCCATAACCTGGATTTTATCATTAAACCCCAATATTAAACACCTTTTTAGTACCCCCCACCC---------ACCAAAAAATGTGACTGGACCGGTTCCTAGTAGCTTTGGGAGCCAT

HANChild2&3 ------TTAAGAAACCGAAACTCTCCACAGTTCACCATCTTCTTCGTCATTCAACCGATGACCCACTCCGTACAACGAATCAGTCTGCTGCGTCATATTGCAAAGCACAAGCGACGTATGCGAACAACTTGAAACACAGAC-----------CGAGCGTTGTACCATTACTAGTCACATTGCATACAGACCTTCCACCGTCATCCCATCTTTCCCACCCGATGGAAAACCGTCTTCTATCATAAACTATGGTAAGATTTCGACCCTGCGAGGTATTCAGTTTCCCCATATCCATAACCTGGATTTTATCATTAAACCCCAATATTAAACACCTTTTTAGTACCCCCCACCC---------ACCAAAAAATGTGACTGGACCGGTTCCTAGTAGCTTTGGGAGCCAT

Pat_D ------TTAAGAAACCGAAACTCTCCACAGTTCACCATCTTCTTCGTCATTCAACCGATGACCCACTCCGTACAACGAATCAGTCTGCTGCGTCATATTGCAAAGCACAAGCGACGTATGCGAACAACTTGAAACACAGAC-----------CGAGCGTTGTACCATTACTAGTCACATTGCATACAGACCTTCCACCGTCATCCCATCTTTCCCACCCGATGGAAAACCGTCTTCTATCATAAACTATGGTAAGATTTCGACCCTGCGAGGTATTCAGTTTCCCCATATCCATAACCTGGATTTTATCATTAAACCCCAATATTAAACACCTTTTTAGTACCCCCCACCC---------ACCAAAAAATGTGACTGGACCGGTTCCTAGTAGCTTTGGGAGCCAT

BE/17/2010 ------TTAAGAAACCGAAACTCTCCACAGTTCACCATCTTCTTCGTCATTCAACCGATGACCCACTCCGTACAACGAATCAGCCTGCTGCGTCATATTGTAAAGCACAAGCGACGTATGCGAACAACTTGAAACACAGAC-----------CGAGCGTTGTACCATTACTAGTCACATTGCATACAGACCTTCCACCGTCATCCCATCTTTCCCACCCGATGGAAAACCGTCTTCTATCATAAACTATGGTAAGATTTCGACCCTGCGAGGTATTCAGTTTCCCCATATCCATAACCTGGATTTTATCATTAAACCCCAATATTAAACACCTTTTTAGTACCCCCCACCC---------ACCAAAAAATGTGACTGGACCGGTTCCTAGTAGCTTTGGGAGCCAT

JER5268 ------TTAAGAAACCGAAACTCTCCACAGTTCACCATCTTCTTCGTCATTCAACCGATGACCCACTCCGTACAACGAATCAGCCTGCTGCGTCATATTGTAAAGCACAAGCGACGTATGCGAACAACTTGAAACACAGAC-----------CGAGCGTTGTACCATTACTAGTCACATTGCATACAGACCTTCCACCGTCATCCCATCTTTCCCACCCGATGGAAAACCGTCTTCTATCATAAACTATGGTAAGATTTCGACCCTGCGAGGTATTCAGTTTCCCCATATCCATAACCTGGATTTTATCATTAAACCCCAATATTAAACACCTTTTTAGTACCCCCCACCC---------ACCAAAAAATGTGACTGGACCGGTTCCTAGTAGCTTTGGGAGCCAT

PAV20 ------TTAAGAAACCGAAACTCTCCACAGTTCACCATCTTCTTCGTCATTCAACCGATGACCCACTCCGTACAACGAATCAGCCTGCTGCGTCATATTGTAAAGCACAAGCGACGTATGCGAACAACTTGAAACACAGAC-----------CGAGCGTTGTACCATTACTAGTCACATTGCATACAGACCTTCCACCGTCATCCCATCTTTCCCACCCGATGGAAAACCGTCTTCTATCATAAACTATGGTAAGATTTCGACCCTGCGAGGTATTCAGTTTCCCCATATCCATAACCTGGATTTTATCATTAAACCCCAATATTAAACACCTTTTTAGTACCCCCCACCC---------ACCAAAAAATGTGACTGGACCGGTTCCTAGTAGCTTTGGGAGCCAT

BE/32/2010 ------TTAAGAAACCGAAACTCTCCACAGTTCACCATCTTCTTCGTCATTCAACCGATGACCCACTCCGTACAACGAATCAGCCTGCTGCGTCATATTGTAAAGCACAAGCGACGTATGCGAACAACTTGAAACACAGAC-----------CGAGCGTTGTACCATTACTAGTCACATTGCATACAGACCTTCCACCGTCATCCCATCTTTCCCACCCGATGGAAAACCGTCTTCTATCATAAACTATGGTAAGATTTCGACCCTGCGAGGTATTCAGTTTCCCCATATCCATAACCTGGATTTTATCATTAAACCCCAATATTAAACACCTTTTTAGTACCCCCCACCC---------ACCAAAAAATGTGACTGGACCGGTTCCTAGTAGCTTTGGGAGCCAT

BE/2/2013 ------TTAAGAAACCGAAACTCTCCACAGTTCACCATCTTCTTCGTCATTCAACCGATGACCCACTCCGTACAACGAATCAGCCTGCTGCGTCATATTGTAAAGCACAAGCGACGTATGCGAACAACTTGAAACACAGAC-----------CGAGCGTTGTACCATTACTAGTCACATTGCATACAGACCTTCCACCGTCATCCCATCTTTCCCACCCGATGGAAAACCGTCTTCTATCATAAACTATGGTAAGATTTCGACCCTGCGAGGTATTCAGTTTCCCCATATCCATAACCTGGATTTTATCATTAAACCCCAATATTAAACACCTTTTTAGTACCCCCCACCC---------ACCAAAAAATGTGACTGGACCGGTTCCTAGTAGCTTTGGGAGCCAT

BE/13/2011 ------TTAAGAAACCGAAACTCTCCACAGTTCACCATCTTCTTCGTCATTCAACCGATGACCCACTCCGTACAACGAATCAGCCTGCTGCGTCATATTGTAAAGCACAAGCGACGTATGCGAACAACTTGAAACACAGAC-----------CGAGCGTTGTACCATTACTAGTCACATTGCATACAGACCTTCCACCGTCATCCCATCTTTCCCACCCGATGGAAAACCGTCTTCTATCATCAACTATGGTAAGATTTCGACCCTGCGAGGTATTCAGTTTCCCCATATCCATAACCTAGATTTTATCATTAAACCCCAATATTAAACACCTTTTTAGTACCCCCCACCC---------ACCAAAAAATGTGACTGGACCGGTTCCTAGTAGCTTTGGGAGCCAT

BE/27/2010 ------TTAAGAAACCGAAACTCTCCACAGTTCACCATCTTCTTCGTCATTCAACCGATGACCCACTCCGTACAACGAATCAGCCTGCTGCGTCATATTGTAAAGCACAAGCGACGTATGCGAACAACTTGAAACACAGAC-----------CGAGCGTTGTACCATTACTAGTCACATTGCATACAGACCTTCCACCGTCATCCCATCTTTCCCACCCGATGGAAAACCGTCTTCTATCATCAACTATGGTAAGATTTCGACCCTGCGAGGTATTCAGTTTCCCCATATCCATAACCTGGATTTTATCATTAAACCCCAATATTAAACACCTTTTTAGTACCCCCCACCC---------ACCAAAAAATGTGACTGGACCGGTTCCTAGTAGCTTTGGGAGCCAT

2CEN5 ------TTAAGAAACCGAAACTCTCCACAGTTCACCATCTTCTTCGTCATTCAACCGATGACCCACTCCGTACAACGAATCAGCCTGCTGCGTCATATTGTAAAGCACAAGCGACGTATGCGAACAACTTGAAACACAGAC-----------CGAGCGTTGTACCATTACTAGTCACATTGCATACAGACCTTCCACCGTCATCCCATCTTTCCCACCCGATGGAAAACCGTCTTCTATCATCAACTATGGTAAGATTTCGACCCTGCGAGGTATTCAGTTTCCCCATATCCATAACCTGGATTTTATCATTAAACCCCAATATTAAACACCTTTTTAGTACCCCCCACCC---------ACCAAAAAATGTGACTGGACCGGTTCCTAGTAGCTTTGGGAGCCAT

BE/3/2010 ------TTAAGAAACCGAAACTCTCCACAGTTCACCATCTTCTTCGTCATTCAACCGATGACCCACTCCGTACAACGAATCAGCCTGCTGCGTCATATTGTAAAGCACAAGCGACGTATGCGAACAACTTGAAACACAGAC-----------CGAGCGTTGTACCATTACTAGTCACATTGCATACAGACCTTCCACCGTCATCCCATCTTTCCCACCCGATGGAAAACCGTCTTCTATCATCAACTATGGTAAGATTTCGACCCTGCGAGGTATTCAGTTTCCCCATATCCATAACCTGGATTTTATCATTAAACCCCAATATTAAACACCTTTTTAGTACCCCCCACCC---------ACCAAAAAATGTGACTGGACCGGTTCCTAGTAGCTTTGGGAGCCAT

HAN29 ------TTAAGAAACCGAAACTCTCCACAGTTCACCATCTTCTTCGTCATTCAACCGATGACCCACTCCGTACAACGAATCAGCCTGCTGCGTCATATTGTAAAGCACAAGCGACGTATGCGAACAACTTGAAACACAGAC-----------CGAGCGTTGTACCATTACTAGTCACATTGCATACAGACCTTCCACCGTCATCCCATCTTTCCCACCCGATGGAAAACCGTCTTCTATCATCAACTATGGTAAGATTTCGACCCTGCGAGGTATTCAGTTTCCCCATATCCATAACCTGGATTTTATCATTAAACCCCAATATTAAACACCTTTTTAGTACCCCCCACCC---------ACCAAAAAATGTGACTGGACCGGTTCCTAGTAGCTTTGGGAGCCAT

BE/2/2012 ------TTAAGAAACCGAAACTCTCCACAGTTCACCATCTTCTTCGTCATTCAACCGATGACCCACTCCGTACAACGAATCAGCCTGCTGCGTCATATTGTAAAGCACAAGCGACGTATGCGAACAACTTGAAACACAGAC-----------CGAGCGTTGTACCATTACTAGTCACATTGCATACAGACCTTCCACCGTCATCCCATCTTTCCCACCCGATGGAAAACCGTCTTCTATCATCAACTATGGTAAGATTTCGACCCTGCGAGGTATTCAGTTTCCCCATATCCATAACCTGGATTTTATCATTAAACCCCAATATTAAACACCTTTTTAGTACCCCCCACCC---------ACCAAAAAATGTGACTGGACCGGTTCCTAGTAGCTTTGGGAGCCAT

BE/12/2010 ------TTAAGAAACCGAAACTCTCCACAGTTCACCATCTTCTTCGTCATTCAACCGATGACCCACTCCGTACAACGAATCAGCCTGCTGCGTCATATTGTAAAGCACAAGCGACGTATGCGAACAACTTGAAACACAGAC-----------CGAGCGTTGTACCATTACTAGTCACATTGCATACAGACCTTCCACCGTCATCCCATCTTTCCCACCCGATGGAAAACCGTCTTCTATCATCAACTATGGTAAGATTTCGACCCTGCGAGGTATTCAGTTTCCCCATATCCATAACCTGGATTTTATCATTAAACCCCAATATTAAACACCTTTTTAGTACCCCCCACCC---------ACCAAAAAATGTGACTGGACCGGTTCCTAGTAGCTTTGGGAGCCAT

BE/29/2011 ------TTAAGAAACCGAAACTCTCCACAGTTCACCATCTTCTTCGTCATTCAACCGATGACCCACTCCGTACAACGAATCAGCCTGCTGCGTCATATTGTAAAGCACAAGCGACGTATGCGAACAACTTGAAACACAGAC-----------CGAGCGTTGTACCATTACTAGTCACATTGCATACAGACCTTCCACCGTCATCCCATCTTTCCCACCCGATGGAAAACCGTCTTCTATCATCAACTATGGTAAGATTTCGACCCTGCGAGGTATTCAGTTTCCCCATATCCATAACCTGGATTTTATCATTAAACCCCAATATTAAACACCTTTTTAGTACCCCCCACCC---------ACCAAAAAATGTGACTGGACCGGTTCCTAGTAGCTTTGGGAGCCAT

BE/28/2010 ------TTAAGAAACCGAAACTCTCCACAGTTCACCATCTTCTTCGTCATTCAACCGATGACCCACTCCGTACAACGAATCAGCCTGCTGCGTCATATTGTAAAGCACAAGCGACGTATGCGAACAACTTGAAACACAGAC-----------CGAGCGTTGTACCATTACTAGTCACATTGCATACAGACCTTCCACCGTCATCCCATCTTTCCCACCCGATGGAAAACCGTCTTCTATCATCAACTATGGTAAGATTTCGACCCTGCGAGGTATTCAGTTTCCCCATATCCATAACCTGGATTTTATCATTAAACCCCAATATTAAACACCTTTTTAGTACCCCCCACCC---------ACCAAAAAATGTGACTGGACCGGTTCCTAGTAGCTTTGGGAGCCAT

BE/20/2010 ------TTAAGAAACCGAAACTCTCCACAGTTCACCATCTTCTTCGTCATTCAACCGATGACCCACTCCGTACAACGAATCAGCCTGCTGCGTCATATTGTAAAGCACAAGCGACGTATGCGAACAACTTGAAACACAGAC-----------CGAGCGTTGTACCATTACTAGTCACATTGCATACAGACCTTCCACCGTCATCCCATCTTTCCCACCCGATGGAAAACCGTCTTCTATCATCAACTATGGTAAGATTTCGACCCTGCGAGGTATTCAGTTTCCCCATATCCATAACCTGGATTTTATCATTAAACCCCAATATTAAACACCTTTTTAGTACCCCCCACCC---------ACCAAAAAATGTGACTGGACCGGTTCCTAGTAGCTTTGGGAGCCAT

HANChild1 ------TTAAGAAACCGAAACTCTCCACAGTTCACCATCTTCTTCGTCATTCAACCGATGACCCACTCCGTACAACGAATCAGCCTGCTGCGTCATATTGTAAAGCACAAGCGACGTATGCGAACAACTTGAAACACAGAC-----------CGAGCGTTGTACCATTACTAGTCACATTGCATACAGACCTTCCACCGTCATCCCATCTTTCCCACCCGATGGAAAACCGTCTTCTATCATCAACTATGGTAAGATTTCGACCCTGCGAGGTATTCAGTTTCCCCATATCCATAACCTGGATTTTATCATTAAACCCCAATATTAAACACCTTTTTAGTACCCCCCACCC---------ACCAAAAAATGTGACTGGACCGGTTCCTAGTAGCTTTGGGAGCCAT

PRA6 ------TTAAGAAACCGAAACTCTCCACAGTTCACCATCTTCTTCGTCATTCAACCGATGACCCACTCCGTACAACGAATCAGCCTGCTGCGTCATATTGTAAAGCACAAGCGACGTATGCGAACAACTTGAAACACAGAC-----------CGAGCGTTGTACCATTACTAGTCACATTGCATACAGACCTTCCACCGTCATCCCATCTTTCCCACCCGATGGAAAACCGTCTTCTATCATCAACTATGGTAAGATTTCGACCCTGCGAGGTATTCAGTTTCCCCATATCCATAACCTGGATTTTATCATTAAACCCCAATATTAAACACCTTTTTAGTACCCCCCACCC---------ACCAAAAAATGTGACTGGACCGGTTCCTAGTAGCTTTGGGAGCCAT

HAN ------TTAAGAAACCGAAACTCTCCACAGTTCAC---CATCTTCGTCATTCAACCGATGACCCACTCCGTACAACGAATCAGTCTGCTGCGTCATATTGCAAAGCACAAGCGACGTATGCGAACAACTTGAAAC--AGACTGTGGTATTAACGACCGTTGTACCATTACTAGTCACATTGCATAGAGACCATCCACCGTCATCCCATCTTTCCCACCCGATGGAAAACCGTCTTCTATCACCAACTATGGTAAGATTTTAACCCTGCGAGGTATTCAGTTTCCCCATATCCATAACCTGGATTTTATCATTAAACCCCAATATTAAACACTTTTTTAGTACCCCCCACCC---------ACCAAAAAATGTGACTGGACCGGTTCCTAGCAGCTCTGGGAGCCAT

BE/29/2010 ------TTAAGAAACCGAAACTCTCCACAGTTCAC---CATCTTCGTCATTCAACCGATGACCCACTCCGTACAACGAATCAGTCTGCTGCGTCATATTGTAAAGCACAAGCGACGTATGCGAACAACTTGAAACACAGACTGTGGTATTACCGACCGTTGTACCATTACTAGTCACATTGCATAGAGACCCTCCACCGTCATCCCATCTTTCCCACCCGATGGAAAACCGTCTTCTATCATCAACTATGATAAGATTTCGACCCTGCGAGGTATTCAGTTTCCCCATATCCATAACCTGGATTTTATCATTAAACCCCAATATTAAACACTTTTTTAGTACCCCCCCACCC--------ACCAAAAAATGTGACTGGACCGGTTCCTAGCAGCTCTGGGAGCCAT

BE/1/2010 ------TTAAGAAACCGAAACTCTCCACAGTTCACCATCTTCTTCGTCATTCAACCGATGACCCACTCCGTACAACGAATCAGCCTGCTGCGTCATATTGTAAAGCACAAGCGACGTATGCGAACAACTTGAAACACAGACTGTGGTATTAACGACCGTTGTACCATTACTAGTCACATTGCATAGAGACCCTCCACCGTCATCCCATCTTTCCCACCCGATGGAAAACCGTCTTCTATCATCAACTATGGTAAGATTTTGACCCTGCGAGGT-TTCAGTTTCCCCATATCCATAACCTGGATTTTATCATTAAACCCCAATATTAAACACTTTTTTAGTACCCCCCACCC---------ACCAAAAAATGTGACTGGACCGGTTCCTAGCAGCTCTGGGAGCCAT

NANU ------TTAAGAAACCGAAACTCTCCACAGTTCACCATCTTCTTCGTCATTCAACCGATGACCCACTCCGTACAACGAATCAGTCTGCTGCGTCATATTGCAAAGCACAAGCAACGTATGCGAACAACTTGAAACACAGACTGTGGTATTAACGACCGTTGTACCATTACTAGTCACATTGCATAGAGACCCTCCACCGTCATCCCATCTTTCCCACCCGATGGAAAACCGTCTTCTATCATCAACTATGGTAAGATTTTGACCCTGCGAGGT-TTCAGTTTCCCCATATCCATAACCTGGATTTTATCATTAAACCCCAATATTAAACACTTTTTTAGTACCCCCCACCC---------ACCAAAAAATGTGACTGGACCGGTTCCTAGCAGCTCTGGGAGCCAT

JER4755 ------TTAAGAAACCGAAACTCTCCACAGTTCACCATCTTCTTCGTCATTCAACCGATGACCCACTCCGTACAACGAATCAGTCTGCTGCGTCATATTGCAAAGCACAAGCGACGTATGCGAACAACTTGAAACACAGACTGTGGTATTAACGACCGTTGTACCATTACTAGTCACATTGCATAGAGACCCTCCACCGTCATCCCATCTTTCCCACCCGATGGAAAACCGTCTTCTATCATCAACTATGGTAAGATTTTGACCCTGCGAGGT-TTCAGTTTCCCCATATCCATAACCTGGATTTTATCATTAAACCCCAATATTAAACACTTTTTTAGTACCCCCCACCC---------ACCAAAAAATGTGACTGGACCGGTTCCTAGCAGCTCTGGGAGCCAT

U4 ------TTAAGAAACCGAAACTCTCCACAGTTCACCATCTTCTTCGTCATTCAACCGATGACCCACTCCGTACAACGAATCAGTCTGCTGCGTCATATTGCAAAGCACAAGCGACGTATGCGAACAACTTGAAACACAGACTGTGGTATTAACGACCGTTGTACCATTACTAGTCACATTGCATAGAGACCCTCCACCGTCATCCCATCTTTCCCACCCGATGGAAAACCGTCTTCTATCATCAACTATGGTAAGATTTTGACCCTGCGAGGT-TTCAGTTTCCCCATATCCATAACCTGGATTTTATCATTAAACCCCAATATTAAACACTTTTTTAGTACCCCCCACCC---------ACCAAAAAATGTGACTGGACCGGTTCCTAGCAGCTCTGGGAGCCAT

Pat_K ------TTAAGAAACCGAAACTCTCCACAGTTCACCATCTTCTTCGTCATTCAACCGATGACCCACTCCGTACAACGAATCGGTCTGCTGCATCATATTGCAAAGCATAAGCGACGTATGCGAACAACTTGAAACACAGACTGTGCTATTACCGACCGTTGTACCATTACTAGTCACATTGCATAGAAACCATCCACCGTCATCCCATCTTTCCCACCCGATGGAAAACCGTCTTCTATCATCAACTATGGTAAGATTTCGACCCTGCGAGGTATTCAGTTTCCCCATATCCATAACCTGGATTTTATCATTAAACCCCAATATTAAACACTTTTTTAGTACCCCCCCACCC--------ACCAAAAAATGTGACTGGACCGGTTCCTAGCAGCTCTGGGAGCCAT

BE/8/2011 ------TTAAGAAACCGAAACTCTCCACAGTTCACCATCTTCTTCGTCATTCAACCGATGACCCACTCCGTACAACGAATCAGTCTGCTGCGTCATATTGTAAAGCACAAGCGACGTAT------------------------TGGTATTAACGACCGTTGTACCATTACTAGTCACATTGCATAGAGACCCTCCACCGTCATCCCATCTTTCCCACCCGATGGAAAACCGTCTTCTATCATCAACTATGGTAAGATTTCGACCCTGCGAGGTATTCAGTTTCCCCATATCCATAACCTGGATTTTATCATTAAACCCCAATATTAAACACTTTTTTAGTACCCCCCACCC---------ACCAAACAATGTGACTGGACCGGTTCCTAGCAGCTCTGGGAGCCAT

NL/Rot5 ------TTAAGAAACCGAAACTCTCCACAGTTCACCATCTTCTTCGTCATTCAACCGATGACCCACTCCGTACAACGAATCAGTCTGCTGCGTCATATTGCAAAACACAAGCGACGTATGCGAACAACTTGAAACACAGACTGTGGTATTACCGACCGTTATACCATTACTAGTCACATTGCATAGAGACCATCCACTGTCATCCCATCTTTCCCACCCGATGGAAAACCGTCTTCTATCATCAACTATGGTAAGATTTCGACCCTGCGAGGTATTCAGTTTCCCCATATCCATAACCTGGATTTTATCATTAAACCCCAATATTAAACACTTTTTTAGTACCCCCCACCC---------ACCAAAAAATGTGACTGGACCGGTTCCTAGCAGCTCTGGAAGCCAT

BE/25/2010 ------TTAAGAAACCGAAACTCTCCACAGTTCACCATCTTCTTCGTCATTCAACCGATGACCCACTCCGTACAACGAATCAGTCTGCTGCGTCATATTGCAAAGCACAAGCGACGTATGCGAACAACTTGAAACACAGACTGTGGTATTACCGACCGTTGTAACATTACTAGTCACATTGCATAGAGACCCTCCACCGTCATCCCATCTTTCCCACCAGATGGAAAACCGTCTTCTATCATCAACTATGGTAAGATTTCGACCCTGCGAGGTATTCAGTTTCCCCATATCCATAACCTGGATTTTATCATTAAACCCCAATATTAAACACTTTTTTAGTACCCCCCCACCC--------ACCAAAAAATGTGACTGGACCGGTTCCTAGCAGCTCTGGGAGCCAT

JER5409 ------TTAAGAAACCGAAACTCTCCACAGTTCACCATCTTCTTCGTCATTCAACCGATGACCCACTCCGTACAACGAATCAGTCTGCTGCGTCATATTGCAAAGCACAAGCGACGTATGCGAACAACTTGAAACACAGACTGTGGTATTACCGACCGTTGTAACATTACTAGTCACATTGCATAGAGACCCTCCACCGTCATCCCATCTTTCCCACCAGATGGAAAACCGTCTTCTATCATCAACTATGGTAAGATTTCGACCCTGCGAGGTATTCAGTTTCCCCATATCCATAACCTGGATTTTATCATTAAACCCCAATATTAAACACTTTTTTAGTACCCCCCCACCC--------ACCAAAAAATGTGACTGGACCGGTTCCTAGCAGCTCTGGGAGCCAT

JER4041 ------TTAAGAAACCGAAACTCTCCACAGTTCACCATCTTCTTCGTCATTCAACCGATGACCCACTCCGTACAACGAATCAGTCTGCTGCGTCATATTGCAAAGCACAAGCGACGTATGCGAACAACTTGAAACACAGACTGTGGTATTACCGACCGTTGTAACATTACTAGTCACATTGCATAGAGACCCTCCACCGTCATCCCATCTTTCCCACCAGATGGAAAACCGTCTTCTATCATCAACTATGGTAAGATTTCGACCCTGCGAGGTATTCAGTTTCCCCATATCCATAACCTGGATTTTATCATTAAACCCCAATATTAAACACTTTTTTAGTACCCCCCCACCC--------ACCAAAAAATGTGACTGGACCGGTTCCTAGCAGCTCTGGGAGCCAT

JER851 ------TTAAGAAACCGAAACTCTCCACAGTTCACCATCTTCTTCGTCATTCAACCGATGACCCACTCCGTACAACGAATCAGTCTGCTGCGTCATATTGCAAAGCACAAGCGACGTATGCGAACAACTTGAAACACAGACTGTGGTATTACCGACCGTTGTAACATTACTAGTCACATTGCATAGAGACCCTCCACCGTCATCCCATCTTTCCCACCAGATGGAAAACCGTCTTCTATCATCAACTATGGTAAGATTTCGACCCTGCGAGGTATTCAGTTTCCCCATATCCATAACCTGGATTTTATCATTAAACCCCAATATTAAACACTTTTTTAGTACCCCCCCACCC--------ACCAAAAAATGTGACTGGACCGGTTCCTAGCAGCTCTGGGAGCCAT

PAV5 ------TTAAGAAACCGAAACTCTCCACAGT---TCATCTTCTTCGTCATTCAACCGATGACCCACTCCGTACAACGAATCAGTCTGCTGCGTCATATTGTAAAGCACAAGCGACGTATGCGAACAACTTGAAACACAGACTGTGGTATTACCGACCGTTGTACCATTACTAGTCACATTGCATAGAGACCCTCCACCGTCATCCCATCTTTCCCACCCGATGGAAAACCGTCTTCTATCATCAACTATGGTAAGATTTCGACCCTGCGAGGTATTCAGTTTCCCCATATCCATAACCTGGATTTTATCATTAAACCCCAATATTAAACACTTTTTTAGTACCCCCCACCC---------ACCAAAAAATGTGACTGGACCGGTTCCTAGCAGCTCTGGGAGCCAT

Pat_C ------TTAAGAAACCGAAACTCTCCACAGTTCACCATCTTCTTCGTCATTCAACCGATGACCCACTCCGTACAACGAATCAGTCTGCTGCGTCATATTGTAAAGCACAAGCGACGTATGCGAACAACTTGAAACACAGACTGTGGTATTACCGACCGTTGTAACATTACTAGTCACATTGCATAGAGACCCTCCACCGTCATCCCATCTTTCCCACCAGATGGAAAACCGTCTTCTATCATCAACTATGGTAAGATTTCGACCCTGCGAGGTATTCAGTTTCCCCATATCCATAACCTGGATTTTATCATTAAACCCCAATATTAAAC--TTTTTTAGTACCCCCCACCC---------ACCAAAAAATGTGACTGGACCGGTTCCTAGCAGCTCTGGGAGCCAT

BE/4/2010 ------TTAAGAAACCGAAACTCTCCACAGTTCACCATCTTCTTCGTCATTCAACCGATGACCCACTCCGTACAACGAATCAGTCTGCTGCGTCATATTGTAAAGCACAAGCGACGTATGCGAACAACTTGAAACACAGAC--TGGTATTACCGACCGTTGTAACATTACTAGTCACATTGCATAGAGACCCTCCACCGTCATCCCATCTTTCCCACCAGATGGAAAACCGTCTTCTATCATCAACTATGGTAAGATTTCGACCCTGCGAGGTATTCAGTTTCCCCATATCCATAACCTGGATTTTATCATTAAACCCCAATATTAAACACTTTTTTAGTACCCCCCACCC---------ACCAAAAAATGTGACTGGACCGGTTCCTAGCAGCTCTGGGAGCCAT

PAV16 ------TTAAGAAACCGAAACTCTCCACAGTTCACCATCTTCTTCGTCATTCAACCGATGACCCACTCCGTACAACGAATCAGTCTGCTGCGTCATATTGTAAAGCACAAGCGACGTATGCGAACAACTTGAAACACAGAC--TGGTATTACCGACCGTTGTAACATTACTAGTCACATTGCATAGAGACCCTCCACCGTCATCCCATCTTTCCCACCAGATGGAAAACCGTCTTCTATCATCAACTATGGTAAGATTTCGACCCTGCGAGGTATTCAGTTTCCCCATATCCATAACCTGGATTTTATCATTAAACCCCAATATTAAACACTTTTTTAGTACCCCCCACCC---------ACCAAAAAATGTGACTGGACCGGTTCCTAGCAGCTCTGGGAGCCAT

JP ------TTAAGAAACCGAAACTCTCCACAGTTCACCATCTTCTTCGTCATTCAACCGATGACCCACTCCGTACAACGAATCAGTCTGCTGCGTCATATTGTAAAGCACAAGCGACGTATGCGAACAACTTGAAACACAGAC--TGGTATTACCGACCGTTGTAACATTACTAGTCACATTGCATAGAGACCCTCCACCGTCATCCCATCTTTCCCACCAGATGGAAAACCGTCTTCTATCATCAACTATGGTAAGATTTCGACCCTGCGAGGTATTCAGTTTCCCCATATCCATAACCTGGATTTTATCATTAAACCCCAATATTAAACACTTTTTTAGTACCCCCCACCC---------ACCAAAAAATGTGACTGGACCGGTTCCTAGCAGCTCTGGGAGCCAT

BE/44/2011 ------TTAAGAAACCGAAACTCTCCACAGTTCACCATCTTCTTCGTCATTCAACCGATGACCCACTCCGTACAACGAATCAGTCTGCTGCGTCATATTGTAAAGCACAAGCGACGTATGCGAACAACTTGAAACACAGAC--TGGTATTACCGACCGTTGTAACATTACTAGTCACATTGCATAGAGACCCTCCACCGTCATCCCATCTTTCCCACCAGATGGAAAACCGTCTTCTATCATCAACTATGGTAAGATTTCGACCCTGCGAGGTATTCAGTTTCCCCATATCCATAACCTGGATTTTATCATTAAACCCCAATATTAAACACTTTTTTAGTACCCCCCACCC---------ACCAAAAAATGTGACTGGACCGGTTCCTAGCAGCTCTGGGAGCCAT

HAN19 ------TTAAGAAACCGAAACTCTCCACAGTTCACCATCTTCTTCGTCATTCAACCGATGACCCACTCCGTACAACGAATCAGTCTGCTGCGTCATATTGTAAAGCACAAGCGACGTATGCGAACAACTTGAAACACAGAC--TGGTATTACCGACCGTTGTAACATTACTAGTCACATTGCATAGAGACCCTCCACCGTCATCCCATCTTTCCCACCAGATGGAAAACCGTCTTCTATCATCAACTATGGTAAGATTTCGACCCTGCGAGGTATTCAGTTTCCCCATATCCATAACCTGGATTTTATCATTAAACCCCAATATTAAACACTTTTTTAGTACCCCCCACCC---------ACCAAAAAATGTGACTGGACCGGTTCCTAGCAGCTCTGGGAGCCAT

PRA7 ------TTAAGAAACCGAAACTCTCCACAGTTCACCATCTTCTTCGTCATTCAACCGATGACCCACTCCGTACAACGAATCAGTCTGCTGCGTCATATTGTAAAGCACAAGCGACGTATGCGAACAACTTGAAACACAGAC--TGGTATTACCGACCGTTGTAACATTACTAGTCACATTGCATAGAGACCCTCCACCGTCATCCCATCTTTCCCACCAGATGGAAAACCGTCTTCTATCATCAACTATGGTAAGATTTCGACCCTGCGAGGTATTCAGTTTCCCCATATCCATAACCTGGATTTTATCATTAAACCCCAATATTAAACACTTTTTTAGTACCCCCCACCC---------ACCAAAAAATGTGACTGGACCGGTTCCTAGCAGCTCTGGGAGCCAT

HAN11 ------TTAAGAAACCGAAACTCTCCACAGTTCACCATCTTCTTCGTCATTCAACCGATGACCCACTCCGTACAACGAATCAGTCTGCTGCGTCATATTGCAAAGCACAAGCGACGTATGCGAACAACTTGAAACACAGACTGTGGTATTAACGACCGTTGTACCATTACTAGTCACATTGCATAGAGACCATCCACCGTCATCCCATCTTTCCCACCCGATGGAAAACCGTCTTCTATCACCAACTATGGTAAGATTTTAACCCTGCGAGGTATTCAGTTTCCCCATATCCATAACCTGGATTTTATCATTAAACCCCAATATTAAACACTTTTTTAGTACCCCCCACCC---------ACCAAAAAATGTGACTGGACCGGTTCCTAGCAGCTCTGGGAGCCAT

NL/Rot6 ------TTAAGAAACCGAAACTCTCCACAGTTCACCATCTTCTTCGTCATTCAACCGATGACCCACTCCGTACAACGAATCAGTCTGCTGCGTCATATTGCAAAGCACAAGCGACGTATGCGAACAACTTGAAACACGGACTGTGGTATTAACGACCGTTGTACCATTACTAGTCACATTGCATAGAGACCCTCCACCGTCATCCCATCTTTCCCACCCGATGGAAAACCGTCTTCTATCATCAACTATGGTAAGATTTCGACCCTGCGAGGTATTCAGTTTCCCCATATCCATAACCTGGATTTTATCATTAAACCCCAATATTAAACACTTTTTTAGTACCCCCCACCC---------ACCAAAAAATGTGACTGGACCGGTTCCTAGCAGCTCTGGGAGCCAT

HANRTR5 ------TTAAGAAACCGAAACTCTCCACAGTTCACCATCTTCTTCGTCATTCAACCGATGACCCACTCCGTACAACGAATCAGTCTGCTGCGTCATATTGTAAAGCACAAGCGACGTATGCGAACAACTTGAAACACAGACTGTGGTATTACCGACCGTTGTAACATTACTAGTCACATTGCATAGAGACCCTCCACCGTCATCCCATCTTTCCCACCAGATGGAAAACCGTCTTCTATCATCAACTATGGTAAGATTTCGACCCTGCGAGGTATTCAGTTTCCCCATATCCATAACCTGGATTTTATCATTAAACCCCAATATTAAACACTTTTTTAGTACCCCCCACCC---------ACCAAAAAATGTGACTGGACCGGTTCCTAGCAGCTCTGGGAGCCAT

HANSCTR9 ------TTAAGAAACCGAAACTCTCCACAGTTCACCATCTTCTTCGTCATTCAACCGATGACCCACTCCGTACAACGAATCAGTCTGCTGCGTCATATTGTAAAGCACAAGCGACGTATGCGAACAACTTGAAACACAGACTGTGGTATTACCGACCGTTGTAACATTACTAGTCACATTGCATAGAGACCCTCCACCGTCATCCCATCTTTCCCACCAGATGGAAAACCGTCTTCTATCATCAACTATGGTAAGATTTCGACCCTGCGAGGTATTCAGTTTCCCCATATCCATAACCTGGATTTTATCATTAAACCCCAATATTAAACACTTTTTTAGTACCCCCCACCC---------ACCAAAAAATGTGACTGGACCGGTTCCTAGCAGCTCTGGGAGCCAT

UK/Lon8 ------TTAAGAAACCGAAACTCTCCACAGTTCACCATCTTCTTCGTCATTCAACCGATGACCCACTCCGTACAACGAATCAGTCTGCTGCGTCATATTGTAAAGCACAAGCGACGTATGCGAACAACTTGAAACACAGACTGTGGTATTACAGACCGTTGTACCATTACTAGTCACATTGCATAGAGACCCTCCACCGTCATCCCATCTTTCCCACCAGATGGAAAACCGTCTTCTATCATCAACTATGGTAAGATTTCGACCCTGCGAGGTATTCAGTTTCCCCATATCCATAACCTGGATTTTATCATTAAACCCCAATATTAAACACTTTTTTAGTACCCCCCACCC---------ACCAAAAAATGTGACTGGACCGGTTCCTAGCAGCTCTGGGAGCCAT

HAN1 ------TTAAGAAACCGAAACTCTCCACAGTTCACCATCTTCTTCGTCATTCAACCGATGACCCACTCCGTACAACGAATCAGTCTGCTGCGTCATATTGCAAAGCACAAGCGACGTATGCGAACAACTTGAAACACAGACTGCGGTATTACCGACCGTTGTACCATTACTAGTCACATTGCATAGAGACCCTCCACCGTCATCCCATCTTTCCCACCCGATGGAAAACCGTCTTCTATCATCAACTATGGTAAGATTTCGACCCTGCGAGGTATTCAGTTTCCCCATATCCATAACCTGGATTTTATCATTAAACCCCAATATTAAACACTTTTTTAGTACCCCCCACCC---------ACCAAAAAATGTGACTGGACCGGTTCCTAGCAGCTCTGGGAGCCAT

HAN16 ------TTAAGAAACCGAAACTCTCCACAGTTCACCATCTTCTTCGTCATTCAACCGATGACCCACTCCATACAACGAATCAGTCTGCTGCGTCATATTGTAAAGCACAAGCGACGTATGCGAACAACTTGAAACACAGACCGTGGTATTACCGACCGTTGTACCATTACTAGTCACATTGCATAGAGACCCTCCACCGTCATCCCATCTTTCCCACCCGATGGAAAACCGTCTTCTATCATCAACTATGGTAAGATTTCGACCCTGCGAGGTATTCAGTTTCCCCATATCCATAACCTGGATTTTATCATTAAACCCCAATATTAAACACTTTTTTAGTACCCCCCACCC---------ACCAAAAAATGTGACTGGACCGGTTCCTAGCAGCTCTGGGAGCCAT

BE/28/2011 ------TTAAGAAACCGAAACTCTCCACAGTTCACCATCTTCTTCGTCATTCAACCGATGACCCACTCCATACAACGAATCAGTCTGCTGCGTCATATTGTAAAGCACAAGCGACGTATGCGAACAACTTGAAACACAGACCGTGGTATTACCGACCGTTGTACCATTACTAGTCACATTGCATAGAGACCCTCCACCGTCATCCCATCTTTCCCACCCGATGGAAAACCGTCTTCTATCATCAACTATGGTAAGATTTCGACCCTGCGAGGTATTCAGTTTCCCCATATCCATAACCTGGATTTTATCATTAAACCCCAATATTAAACACTTTTTTAGTACCCCCCACCC---------ACCAAAAAATGTGACTGGACCGGTTCCTAGCAGCTCTGGGAGCCAT

NL/Rot3 ------TTAAGAAACCGAAACTCTCCACAGTTCACCATCTTCTTCGTCATTCAACCGATGACCCACTCCATACAACGAATCAGTCTGCTGCGTCATATTGTAAAGCACAAGCGACGTATGCGAACAACTTGAAACACAGACCGTGGTATTACCGACCGTTGTACCATTACTAGTCACATTGCATAGAGACCCTCCACCGTCATCCCATCTTTCCCACCCGATGGAAAACCGTCTTCTATCATCAACTATGGTAAGATTTCGACCCTGCGAGGTATTCAGTTTCCCCATATCCATAACCTGGATTTTATCATTAAACCCCAATATTAAACACTTTTTTAGTACCCCCCACCC---------ACCAAAAAATGTGACTGGACCGGTTCCTAGCAGCTCTGGGAGCCAT

UK/Lon5 ------TTAAGAAACCGAAACTCTCCACAGTTCACCATCTTCTTCGTCATTCAACCGATGACCCACTCCATACAACGAATCAGTCTGCTGCGTCATATTGTAAAGCACAAGCGACGTATGCGAACAACTTGAAACACAGACCGTGGTATTACCGACCGTTGTACCATTACTAGTCACATTGCATAGAGACCCTCCACCGTCATCCCATCTTTCCCACCCGATGGAAAACCGTCTTCTATCATCAACTATGGTAAGATTTCGACCCTGCGAGGTATTCAGTTTCCCCATATCCATAACCTGGATTTTATCATTAAACCCCAATATTAAACACTTTTTTAGTACCCCCCACCC---------ACCAAAAAATGTGACTGGACCGGTTCCTAGCAGCTCTGGGAGCCAT

HANSCTR11A ------TTAAGAAACCGAAACTCTCCACAGTTCACCATCTTCTTCGTCATTCAACCGATGACCCACTCCATACAACGAATCAGTCTGCTGCGTCATATTGTAAAGCACAAGCGACGTATGCGAACAACTTGAAACACAGACCGTGGTATTACCGACCGTTGTACCATTACTAGTCACATTGCATAGAGACCCTCCACCGTCATCCCATCTTTCCCACCCGATGGAAAACCGTCTTCTATCATCAACTATGGTAAGATTTCGACCCTGCGAGGTATTCAGTTTCCCCATATCCATAACCTGGATTTTATCATTAAACCCCAATATTAAACACTTTTTTAGTACCCCCCACCC---------ACCAAAAAATGTGACTGGACCGGTTCCTAGCAGCTCTGGGAGCCAT

BE/2/2010 ------TTAAGAAACCGAAACTCTCCACAGTTCACCATCTTCTTCGTCATTCAACCGATGACCCACTCCGTACAACGAATCAGTCTGCTGCGTCATATTGTAAAGCACAAGCGACGTATGCGAACAACTTGAAACACAGACTGCGGTATTACCGAGCGTTGTACCATTACTAGTCACATTGCATAGAGACCCTCCACCGTCATCCCATCTTTCCCACCCGATGGAAAACCGTCTTCTATCATCAACTATGGTAAGATTTCGACCCTGCGAGGTATTCAGTTTCCCCATATCCATAACCTGGATTTTATCATTAAACCCCAATATTAAACACTTTTTTAGTACCCCCCACCC---------ACCAAAAAATGTGACTGGACCGGTTCCTAGCAGCTCTGGGAGCCAT

HAN40 ------TTAAGAAACCGAAACTCTCCACAGTTCACCATCTTCTTCGTCATTCAACCGATGACCCACTCCGTACAACGAATCAGTCTGCTGCGTCATATTGTAAAGCACAAGCGACGTATGCGAACAACTTGAAACACAGACTGCGGTATTACCGAGCGTTGTACCATTACTAGTCACATTGCATAGAGACCCTCCACCGTCATCCCATCTTTCCCACCCGATGGAAAACCGTCTTCTATCATCAACTATGGTAAGATTTCGACCCTGCGAGGTATTCAGTTTCCCCATATCCATAACCTGGATTTTATCATTAAACCCCAATATTAAACACTTTTTTAGTACCCCCCACCC---------ACCAAAAAATGTGACTGGACCGGTTCCTAGCAGCTCTGGGAGCCAT

BE/12/2012 ------TTAAGAAACCGAAACTCTCCACAGTTCACCATCTTCTTCGTCATTCAACCGATGACCCACTCCGTACAACGAATCAGTCTGCTGCGTCATATTGTAAAGCACAAGCGACGTATGCGAACAACTTGAAACACAGACTGCGGTATTACCGAGCGTTGTACCATTACTAGTCACATTGCATAGAGACCCTCCACCGTCATCCCATCTTTCCCACCCGATGGAAAACCGTCTTCTATCATCAACTATGGTAAGATTTCGACCCTGCGAGGTATTCAGTTTCCCCATATCCATAACCTGGATTTTATCATTAAACCCCAATATTAAACACTTTTTTAGTACCCCCCACCC---------ACCAAAAAATGTGACTGGACCGGTTCCTAGCAGCTCTGGGAGCCAT

2CEN30 ------TTAAGAAACCGAAACTCTCCACAGTTCACCATCTTCTTCGTCATTCAACCGATGACCCACTCCGTACAACGAATCAGTCTGCTGCGTCATATTGTAAAGCACAAGCGACGTATGCGAACAACTTGAAACACAGACTGTGGTATTACCGACCGTTGTACCATTACTAGTCACATTGCATAGAGACCCTCCACCGTCATCCCATCTTTCCCACCCGATGGAAAACCGTCTTCTATCATCAACTATGGTAAGATTTCGACCCTGCGAGGTATTCAGTTTCCCCATATCCATAACCTGGATTTTATCATTAAACCCCAATATTAAACACTTTTTTAGTACCCCCCACCC---------ACCAAAAAATGTGACTGGACCGGTTCCTAGCAGCTCCGGGAGCCAT

PRA2 ------TTAAGAAACCGAAACTCTCCACAGTTCACCATCTTCTTCGTCATTCAACCGATGACCCACTCCGTACAACGAATCAGTCTGCTGCGTCATATTGTAAAGCACAAGCGACGTATGCGAACAACTTGAAACACAGACTGTGGTATTACCGACCGTTGTACCATTACTAGTCACATTGCATAGAGACCCTCCACCGTCATCCCATCTTTCCCACCCGATGGAAAACCGTCTTCTATCATCAACTATGGTAAGATTTCGACCCTGCGAGGTATTCAGTTTCCCCATATCCATAACCTGGATTTTATCATTAAACCCCAATATTAAACACTTTTTTAGTACCCCCCACCC---------ACCAAAAAATGTGACTGGACCGGTTCCTAGCAGCTCCGGGAGCCAT

BE/15/2010 ------TTAAGAAACCGAAACTCTCCACAGTTCACCATCTTCTTCGTCATTCAACCGATGACCCACTCCGTACAACGAATCAGTCTGCTGCGTCATATTGTAAAGCACAAGCGACGTATGCGAACAACTTGAAACACAGACTGTGGTATTACCGACCGTTGTACCATTACTAGTCACATTGCATAGAGACCCTCCACCGTCATCCCATCTTTCCCACCCGATGGAAAACCGTCTTCTATCATCAACTATGGTAAGATTTCGACCCTGCGAGGTATTCAGTTTCCCCATATCCATAACCTGAATTTTATCATTAAACCCCAATATTAAACACTTTTTTAGTACCCCCCACCC---------ACCAAAAAATGTGACTGGACCGGTTCCTAGCAGCTCTGGGAGCCAT

HAN8 ------TTAAGAAACCGAAACTCTCCACAGTTCACCATCTTCTTCGTCATTCAACCGATGACCCACTCCGTACAACGAATCAGTCTGCTGCGTCATATTGTAAAGCACAAGCGACGTATGCGAACAACTTGAAACACAGACTGTGGTATTACCGACCGTTGTACCATTACTAGTCACATTGCATAGAGACCCTCCACCGTCATCCCATCTTTCCCACCCGATGGAAAACCGTCTTCTATCATCAACTATGGTAAGATTTCGACCCTGCGAGGTATTCAGTTTCCCCATATCCATAACCTGAATTTTATCATTAAACCCCAATATTAAACACTTTTTTAGTACCCCCCACCC---------ACCAAAAAATGTGACTGGACCGGTTCCTAGCAGCTCTGGGAGCCAT

HAN34 ------TTAAGAAACCGAAACTCTCCACAGTTCACCATCTTCTTCGTCATTCAACCGATGACCCACTCCGTACAACGAATCAGTCTGCTGCGTCATATTGTAAAGCACAAGCGACGTATGCGAACAACTTGAAACACAGACTGTGGTATTACCGACCGTTGTACCATTACTAGTCACATTGCATAGAGACCCTCCACCGTCATCCCATCTTTCCCACCCGATGGAAAACCGTCTTCTATCATCAACTATGGTAAGATTTCGACCCTGCGAGGTATTCAGTTTCCCCATATCCATAACCTGAATTTTATCATTAAACCCCAATATTAAACACTTTTTTAGTACCCCCCACCC---------ACCAAAAAATGTGACTGGACCGGTTCCTAGCAGCTCTGGGAGCCAT

JER4035 ------TTAAGAAACCGAAACTCTCCACAGTTCACCATCTTCTTCGTCATTCAACCGATGACCCACTCCGTACAACGAATCAGTCTGCTGCGTCATATTGTAAAGCACAAGCGACGTATGCGAACAACTTGAAACACAGACTGTGGTATTACCGACCGTTGTACCATTACTAGTCACATTGCATAGAGACCCTCCACCGTCATCCCATCTTTCCCACCCGATGGAAAACCGTCTTCTATCATCAACTATGGTAAGATTTCGACCCTGCGAGGTATTCAGTTTCCCCATATCCATAACCTGAATTTTATCATTAAACCCCAATATTAAACACTTTTTTAGTACCCCCCACCC---------ACCAAAAAATGTGACTGGACCGGTTCCTAGCAGCTCTGGGAGCCAT

BE/8/2012 ------TTAAGAAACCGAAACTCTCCACAGTTCACCATCTTCTTCGTCATTCAACCGATGACCCACTCCGTACAACGAATCAGTCTGCTGCGTCATATTGTAAAGCACAAGCGACGTATGCGAACAACTTGAAACACAGACTGTGGTATTACCGACCGTTGTACCATTACTAGTCACATTGCATAGAGACCCTCCACCGTCATCCCATCTTTCCCACCCGATGGAAAACCGTCTTCTATCATCAACTATGGTAAGATTTCGACCCTGCGAGGTATTCAGTTTCCCCATATCCATAACCTGAATTTTATCATTAAACCCCAATATTAAACACTTTTTTAGTACCCCCCACCC---------ACCAAAAAATGTGACTGGACCGGTTCCTAGCAGCTCTGGGAGCCAT

JER5695 ------TTAAGAAACCGAAACTCTCCACAGTTCACCATCTTCTTCGTCATTCAACCGATGACCCACTCCGTACAACGAATCAGTCTGCTGCGTCATATTGTAAAGCACAAGCGACGTATGCGAACAACTTGAAACACAGACTGTGGTATTACCGACCGTTGTACCATTACTAGTCACATTGCATAGAGACCCTCCACCGTCATCCCATCTTTCCCACCCGATGGAAAACCGTCTTCTATCATCAACTATGGTAAGATTTCGACCCTGCGAGGTATTCAGTTTCCCCATATCCATAACCTGAATTTTATCATTAAACCCCAATATTAAACACTTTTTTAGTACCCCCCACCC---------ACCAAAAAATGTGACTGGACCGGTTCCTAGCAGCTCTGGGAGCCAT

CZ/2/2012 ------TTAAGAAACCGAAACTCTCCACAGTTCACCATCTTCTTCGTCATTCAACCGATGACCCACTCCGTACAACGAATCAGTCTGCTGCGTCATATTGTAAAGCACAAGCGACGTATGCGAACAACTTGAAACACAGACTGTGGTATTACCGACCGTTGTACCATTACTAGTCACATTGCATAGAGACCCTCCACCGTCATCCCATCTTTCCCACCCGATGGAAAACCGTCTTCTATCATCAACTATGGTAAGATTTCGACCCTGCGAGGTATTCAGTTTCCCCATATCCATAACCTGAATTTTATCATTAAACCCCAATATTAAACACTTTTTTAGTACCCCCCACCC---------ACCAAAAAATGTGACTGGACCGGTTCCTAGCAGCTCTGGGAGCCAT

BE/5/2011 ------TTAAGAAACCGAAACTCTCCACAGTTCACCATCTTCTTCGTCATTCAACCGATGACCCACTCCGTACAACGAATCAGTCTGCTGCGTCATATTGTAAAGCACAAGCGACGTATGCGAACAACTTGAAACACAGACTGTGGTATTACCGACCGTTGTACCATTACTAGTCACATTGCATAGAGACCCTCCACCGTCATCCCATCTTTCCCACCCGATGGAAAACCGTCTTCTATCATCAACTATGGTAAGATTTCGACCCTGCGAGGTATTCAGTTTCCCCATATCCATAACCTGAATTTTATCATTAAACCCCAATATTAAACACTTTTTTAGTACCCCCCACCC---------ACCAAAAAATGTGACTGGACCGGTTCCTAGCAGCTCTGGGAGCCAT

CZ/1/2012 ------TTAAGAAACCGAAACTCTCCACAGTTCACCATCTTCTTCGTCATTCAACCGATGACCCACTCCGTACAACGAATCAGTCTGCTGCGTCATATTGTAAAGCACAAGCGACGTATGCGAACAACTTGAAACACAGACTGTGGTATTACCGACCGTTGTACCATTACTAGTCACATTGCATAGAGACCCTCCACCGTCATCCCATCTTTCCCACCCGATGGAAAACCATCTTCTATCATCAACTATGGTAAGATTTCGACCCTGCGAGGTATTCAGTTTCCCCATATCCATAACCTGGATTTTATCATTAAACCCCAATATTAAACACTTTTTTAGTACCCCCCACCC---------ACCAAAAAATGTGACTGGACCGGTTCCTAGCAGCTCTGGGAGCCAT

BE/24/2011 ------TTAAGAAACCGAAACTCTCCACAGTTCACCATCTTCTTCGTCATTCAACCGATGACCCACTCCGTACAACGAATCAGTCTGCTGCGTCATATTGTAAAGCACAAGCGACGTATGCGAACAACTTGAAACACAGACTGTGGTATTACCGACCGTTGTACCATTACTAGTCACATTGCATAGAGACCCTCCACCGTCATCCCATCTTTCCCACCCGATGGAAAACCATCTTCTATCATCAACTATGGTAAGATTTCGACCCTGCGAGGTATTCAGTTTCCCCATATCCATAACCTGGATTTTATCATTAAACCCCAATATTAAACACTTTTTTAGTACCCCCCACCC---------ACCAAAAAATGTGACTGGACCGGTTCCTAGCAGCTCTGGGAGCCAT

BE/14/2011 ------TTAAGAAACCGAAACTCTCCACAGTTCACCATCTTCTTCGTCATTCAACCGATGACCCACTCCGTACAACGAATCAGTCTGCTGCGTCATATTGTAAAGCACAAGCGACGTATGCGAACAACTTGAAACACAGACTGTGGTATTACCGACCGTTGTACCATTACTAGTCACATTGCATAGAGACCCTCCACCGTCATCCCATCTTTCCCACCCGATGGAAAACCATCTTCTATCATCAACTATGGTAAGATTTCGACCCTGCGAGGTATTCAGTTTCCCCATATCCATAACCTGGATTTTATCATTAAACCCCAATATTAAACACTTTTTTAGTACCCCCCACCC---------ACCAAAAAATGTGACTGGACCGGTTCCTAGCAGCTCTGGGAGCCAT

BE/9/2011 ------TTAAGAAACCGAAACTCTCCACAGTTCACCATCTTCTTCGTCATTCAACCGATGACCCACTCCGTACAACGAATCAGTCTGCTGCGTCATATTGTAAAGCACAAGCGACGTATGCGAACAACTTGAAACACAGACTGTGGTATTACCGACCGTTGTACCATTACTAGTCACATTGCATAGAGACCCTCCACCGTCATCCCATCTTTCCCACCCGATGGAAAACCATCTTCTATCATCAACTATGGTAAGATTTCGACCCTGCGAGGTATTCAGTTTCCCCATATCCATAACCTGGATTTTATCATTAAACCCCAATATTAAACACTTTTTTAGTACCCCCCACCC---------ACCAAAAAATGTGACTGGACCGGTTCCTAGCAGCTCTGGGAGCCAT

HANRTR1A ------TTAAGAAACCGAAACTCTCCACAGTTCACCATCTTCTTCGTCATTCAACCGATGACCCACTCCGTACAACGAATCAGTCTGCTGCGTCATATTGTAAAGCACAAGCGACGTATGCGAACAACTTGAAACACAGACTGTGGTATTACCGACCGTTGTACCATTACTAGTCACATTGCATAGAGACCCTCCACCGTCATCCCATCTTTCCCACCCGATGGAAAACCATCTTCTATCATCAACTATGGTAAGATTTCGACCCTGCGAGGTATTCAGTTTCCCCATATCCATAACCTGGATTTTATCATTAAACCCCAATATTAAACACTTTTTTAGTACCCCCCACCC---------ACCAAAAAATGTGACTGGACCGGTTCCTAGCAGCTCTGGGAGCCAT

BE/1/2011 ------TTAAGAAACCGAAACTCTCCACAGTTCACCATCTTCTTCGTCATTCAACCGATGACCCACTCCGTACAACGAATCAGTCTGCTGCGTCATATTGTAAAGCACAAGCGACGTATGCGAACAACTTGAAACACAGACTGTGGTATTACCGACCGTTGTACCATTACTAGTCACATTGCATAGAGACCCTCCACCGTCATCCCATCTTTCCCACCCGATGGAAAACCGTCCTCTATCATCAACTATGGTAAGATTTCGACCCTGCGAGGTATTCAGTTTCCCCATATCCATAACCTGGATTTTATCATTAAACCCCAATATTAAACACTTTTTTAGTACCCCCCACCC---------ACCAAAAAATGTGACTGGACCGGTTCCTAGCAGCTCTGGGAGCCAT

BE/9/2012 ------TTAAGAAACCGAAACTCTCCACAGTTCACCATCTTCTTCGTCATTCAACCGATGACCCACTCCGTACAACGAATCAGTCTGCTGCGTCATATTGTAAAGCACAAGCGACGTATGCGAACAACTTGAAACACAGACTGTGGTATTACCGACCGTTGTACCATTACTAGTCACATTGCATAGAGACCCTCCACCGTCATCCCATCTTTCCCACCCGATGGAAAACCGTCCTCTATCATCAACTATGGTAAGATTTCGACCCTGCGAGGTATTCAGTTTCCCCATATCCATAACCTGGATTTTATCATTAAACCCCAATATTAAACACTTTTTTAGTACCCCCCACCC---------ACCAAAAAATGTGACTGGACCGGTTCCTAGCAGCTCTGGGAGCCAT

BE/8/2010 ------TTAAGAAACCGAAACTCTCCACAGTTCACCATCTTCTTCGTCATTCAACCGATGACCCACTCCGTACAACGAATCAGTCTGCTGCGTCATATTGTAAAGCACAAGCGACGTATGCGAACAACTTGAAACACAGACTGTGGTATTACCGACCGTTGTACCATTACTAGTCACATTGCATAGAGACCCTCCACCGTCATCCCATCTTTCCCACCCGATGGAAAACCGTCCTCTATCATCAACTATGGTAAGATTTCGACCCTGCGAGGTATTCAGTTTCCCCATATCCATAACCTGGATTTTATCATTAAACCCCAATATTAAACACTTTTTTAGTACCCCCCACCC---------ACCAAAAAATGTGACTGGACCGGTTCCTAGCAGCTCTGGGAGCCAT

HAN32 ------TTAAGAAACCGAAACTCTCCACAGTTCACCATCTTCTTCGTCATTCAACCGATGACCCACTCCGTACAACGAATCAGTCTGCTGCGTCATATTGTAAAGCACAAGCGACGTATGCGAACAACTTGAAACACAGACTGTGGTATTACCGACCGTTGTACCATTACTAGTCACATTGCATAGAGACCCTCCACCGTCATCCCATCTTTCCCACCCGATGGAAAACCGTCTTCTATCATCAACTATGGTAAGATTTCGACCCTGCGAGGTATTCAGTTTCCCCATATCCATAACCTGGATTTTATCATTAAACCCCAATATTAAACACTTTTTTAGTACCCCCCACCC---------ACCAAAAAATGTGACTGGACCGGTTCCTAGCAGCTCTGGGAGCCAT

PAV8 ------TTAAGAAACCGAAACTCTCCACAGTTCACCATCTTCTTCGTCATTCAACCGATGACCCACTCCGTACAACGAATCAGTCTGCTGCGTCATATTGTAAAGCACAAGCGACGTATGCGAACAACTTGAAACACAGACTGTGGTATTACCGACCGTTGTACCATTACTAGTCACATTGCATAGAGACCCTCCACCGTCATCCCATCTTTCCCACCCGATGGAAAACCGTCTTCTATCATCAACTATGGTAAGATTTCGACCCTGCGAGGTATTCAGTTTCCCCATATCCATAACCTGGATTTTATCATTAAACCCCAATATTAAACACTTTTTTAGTACCCCCCACCC---------ACCAAAAAATGTGACTGGACCGGTTCCTAGCAGCTCTGGGAGCCAT

BE/24/2010 ------TTAAGAAACCGAAACTCTCCACAGTTCACCATCTTCTTCGTCATTCAACCGATGACCCACTCCGTACAACGAATCAGTCTGCTGCGTCATATTGTAAAGCACAAGCGACGTATGCGAACAACTTGAAACACAGACTGTGGTATTACCGACCGTTGTACCATTACTAGTCACATTGCATAGAGACCCTCCACCGTCATCCCATCTTTCCCACCCGATGGAAAACCGTCTTCTATCATCAACTATGGTAAGATTTCGACCCTGCGAGGTATTCAGTTTCCCCATATCCATAACCTGGATTTTATCATTAAACCCCAATATTAAACACTTTTTTAGTACCCCCCACCC---------ACCAAAAAATGTGACTGGACCGGTTCCTAGCAGCTCTGGGAGCCAT

HAN3 ------TTAAGAAACCGAAACTCTCCACAGTTCACCATCTTCTTCGTCATTCAACCGATGACCCACTCCGTACAACGAATCAGTCTGCTGCGTCATATTGTAAAGCACAAGCGACGTATGCGAACAACTTGAAACACAGACTGTGGTATTACCGACCGTTGTACCATTACTAGTCACATTGCATAGAGACCCTCCACCGTCATCCCATCTTTCCCACCCGATGGAAAACCGTCTTCTATCATCAACTATGGTAAGATTTCGACCCTGCGAGGTATTCAGTTTCCCCATATCCATAACCTGGATTTTATCATTAAACCCCAATATTAAACACTTTTTTAGTACCCCCCACCC---------ACCAAAAAATGTGACTGGACCGGTTCCTAGCAGCTCTGGGAGCCAT

BE/45/2011 ------TTAAGAAACCGAAACTCTCCACAGTTCACCATCTTCTTCGTCATTCAACCGATGACCCACTCCGTACAACGAATCAGTCTGCTGCGTCATATTGTAAAGCACAAGCGACGTATGCGAACAACTTGAAACACAGACTGTGGTATTACCGACCGTTGTACCATTACTAGTCACATTGCATAGAGACCCTCCACCGTCATCCCATCTTTCCCACCCGATGGAAAACCGTCTTCTATCATCAACTATGGTAAGATTTCGACCCTGCGAGGTATTCAGTTTCCCCATATCCATAACCTGGATTTTATCATTAAACCCCAATATTAAACACTTTTTTAGTACCCCCCACCC---------ACCAAAAAATGTGACTGGACCGGTTCCTAGCAGCTCTGGGAGCCAT

CZ/3/2012 ------TTAAGAAACCGAAACTCTCCACAGTTCACCATCTTCTTCGTCATTCAACCGATGACCCACTCCGTACAACGAATCAGTCTGCTGCGTCATATTGTAAAGCACAAGCGACGTATGCGAACAACTTGAAACACAGACTGTGGTATTACCGACCGTTGTACCATTACTAGTCACATTGCATAGAGACCCTCCACCGTCATCCCATCTTTCCCACCCGATGGAAAACCGTCTTCTATCATCAACTATGGTAAGATTTCGACCCTGCGAGGTATTCAGTTTCCCCATATCCATAACCTGGATTTTATCATTAAACCCCAATATTAAACACTTTTTTAGTACCCCCCACCC---------ACCAAAAAATGTGACTGGACCGGTTCCTAGCAGCTCTGGGAGCCAT

3301 ------TTAAGAAACCGAAACTCTCCACAGTTCACCATCTTCTTCGTCATTCAACCGATGACCCACTCCGTACAACGAATCAGTCTGCTGCGTCATATTGTAAAGCACAAGCGACGTATGCGAACAACTTGAAACACAGACTGTGGTATTACCGACCGTTGTACCATTACTAGTCACATTGCATAGAGACCCTCCACCGTCATCCCATCTTTCCCACCCGATGGAAAACCGTCTTCTATCATCAACTATGGTAAGATTTCGACCCTGCGAGGTATTCAGTTTCCCCATATCCATAACCTGGATTTTATCATTAAACCCCAATATTAAACACTTTTTTAGTACCCCCCACCC---------ACCAAAAAATGTGACTGGACCGGTTCCTAGCAGCTCTGGGAGCCAT

BE/13/2012 ------TTAAGAAACCGAAACTCTCCACAGTTCACCATCTTCTTCGTCATTCAACCGATGACCCACTCCGTACAACGAATCAGTCTGCTGCGTCATATTGTAAAGCACAAGCGACGTATGCGAACAACTTGAAACACAGACTGTGGTATTACCGACCGTTGTACCATTACTAGTCACATTGCATAGAGACCCTCCACCGTCATCCCATCTTTCCCACCCGATGGAAAACCGTCTTCTATCATCAACTATGGTAAGATTTCGACCCTGCGAGGTATTCAGTTTCCCCATATCCATAACCTGGATTTTATCATTAAACCCCAATATTAAACACTTTTTTAGTACCCCCCACCC---------ACCAAAAAATGTGACTGGACCGGTTCCTAGCAGCTCTGGGAGCCAT

UK/Lon4 ------TTAAGAAACCGAAACTCTCCACAGTTCACCATCTTCTTCGTCATTCAACCGATGACCCACTCCGTACAACGAATCAGTCTGCTGCGTCATATTGTAAAGCACAAGCGACGTATGCGAACAACTTGAAACACAGACTGTGGTATTACCGACCGTTGTACCATTACTAGTCACATTGCATAGAGACCCTCCACCGTCATCCCATCTTTCCCACCCGATGGAAAACCGTCTTCTATCATCAACTATGGTAAGATTTCGACCCTGCGAGGTATTCAGTTTCCCCATATCCATAACCTGGATTTTATCATTAAACCCCAATATTAAACACTTTTTTAGTACCCCCCACCC---------ACCAAAAAATGTGACTGGACCGGTTCCTAGCAGCTCTGGGAGCCAT

PRA8 ------TTAAGAAACCGAAACTCTCCACAGTTCACCATCTTCTTCGTCATTCAACCGATGACCCACTCCGTACAACGAATCAGTCTGCTGCGTCATATTGTAAAGCACAAGCGACGTATGCGAACAACTTGAAACACAGACTGTGGTATTACCGACCGTTGTACCATTACTAGTCACATTGCATAGAGACCCTCCACCGTCATCCCATCTTTCCCACCCGATGGAAAACCGTCTTCTATCATCAACTATGGTAAGATTTCGACCCTGCGAGGTATTCAGTTTCCCCATATCCATAACCTGGATTTTATCATTAAACCCCAATATTAAACACTTTTTTAGTACCCCCCACCC---------ACCAAAAAATGTGACTGGACCGGTTCCTAGCAGCTCTGGGAGCCAT

Pat_G ------TTAAGAAACCGAAACTCTCCACAGTTCACCATCTTCTTCGTCATTCAACCGATGACCCACTCCGTACAACGAATCAGTCTGCTGCGTCATATTGTAAAGCACAAGCGACGTATGCGAACAACTTGAAACACAGACTGTGGTATTACCGACCGTTGTACCATTACTAGTCACATTGCATAGAGACCCTCCACCGTCATCCCATCTTTCCCACCCGATGGAAAACCGTCTTCTATCATCAACTATGGTAAGATTTCGACCCTGCGAGGTATTCAGTTTCCCCATATCCATAACCTGGATTTTATCATTAAACCCCAATATTAAACACTTTTTTAGTACCCCCCACCC---------ACCAAAAAATGTGACTGGACCGGTTCCTAGCAGCTCTGGGAGCCAT

PRA1 ------TTAAGAAACCGAAACTCTCCACAGTTCACCATCTTCTTCGTCATTCAACCGATGACCCACTCCGTACAACGAATCAGTCTGCTGCGTCATATTGTAAAGCACAAGCGACGTATGCGAACAACTTGAAACACAGACTGTGGTATTACCGACCGTTGTACCATTACTAGTCACATTGCATAGAGACCCTCCACCGTCATCCCATCTTTCCCACCCGATGGAAAACCGTCTTCTATCATCAACTATGGTAAGATTTCGACCCTGCGAGGTATTCAGTTTCCCCATATCCATAACCTGGATTTTATCATTAAACCCCAATATTAAACACTTTTTTAGTACCCCCCACCC---------ACCAAAAAATGTGACTGGACCGGTTCCTAGCAGCTCTGGGAGCCAT

BE/18/2010 ------TTAAGAAACCGAAACTCTCCACAGTTCACCATCTTCTTCGTCATTCAACCGATGACCCACTCCGTACAACGAATCAGTCTGCTGCGTCATATTGTAAAGCACAAGCGACGTATGCGAACAACTTGAAACACAGACTGTGGTATTACCGACCGTTGTACCATTACTAGTCACATTGCATAGAGACCCTCCACCGTCATCCCATCTTTCCCACCCGATGGAAAACCGTCTTCTATCATCAACTATGGTAAGATTTCGACCCTGCGAGGTATTCAGTTTCCCCATATCCATAACCTGGATTTTATCATTAAACCCCAATATTAAACACTTTTTTAGTACCCCCCACCC---------ACCAAAAAATGTGACTGCACCGGTTCCTAGCAGCTCTGGGAGCCAT

BE/4/2012 ------TTAAGAAACCGAAACTCTCCACAGTTCACCATCCTCTTCGTCATTCAACCGATGACCCACTCCGTACAACGAATCAGTCTGCTGTGTCACATTGCAAACTACTAGCGACGTATGCGAACAACTTGAAACACGGGCTGTTGTATTGACGACCGTTGTACCATTACTAGTCACATTGCATAGAGACCATCCACCGTCATCCCATCTTTCCCACCCGATGGAAAACCGTCTTCTATCATCAACTATGGTAAGATTTCAACCCTGCGAGGTATTCAGTTTCCCCATATCCATAACCTGGATTTTATCATTAAACCCCAATATTAAACACTTTTTTAGTACCCCCCACCC---------ACCAAAAAATGTGACTGGACCGGTTCCTAGCAGCTCTGGGAGCCAT

Toledo ------TTAAGAAACCGAAACTCTCCACAGTTCACCATCTTCTTCGTCATTCAACCGATGACCCACTCCGTACAACGAATCAGTCTGCTGTGTCACACTGCAAACTACTAGCGACGTATGCAAACAACTTGAAACACGGGCTGTTGTATTGACGACCGTTGTACCATTACTAGTCACATTGCATAGAGACCATCCACCGTCATCCCATCTTTCCCACCCGATGGAAAACCGTCTTCTATCATCAACTATGGTAAGATTTCGACCCTGCGAGGTATTCAGTTTCCCCATATCCATAACCTGGATTTTATCATTAAACCCCAATATTAAACACTTTTTTAGTACCCCCCCACCC--------ACCAAAAAATGTGACTGGACCGGTTCCTAGCAGCTCTGGGAGCCAT

NL/Rot2 ------TTAAGAAACCGAAACTCTCCACAGTTCACCATCTTCTTCGTCATTCAACCGATGACCCACTCCGTACAACGAATCAGTCTGCTGCGTCATATTGCAAAGCACAAGA----------------------------------------------------------------------------------------------------------------AAAACCGTCTTCTATCATCAACTATGGTAAGATTTCGACCCTGCGAGGTATTCAGTTTCCCCATATCCATAACCTGGATTTTATCATTAAACCCCAATATTAAACACCTTTTTAGTACCCCCCACCC---------ACCAAAAAATGTGACTGGACCGGTTCCTAGCAGCTCTGGGAGCCAT

BE/13/2010 ------TTAAGAAACCGAAACTCTCCACAGTTCACCATCCTCTTCGTCATTCAACCGATGACCCACTCCGTACAACGAATCAGTCTGCTGTGTCACATTGCAAACTACTAGCGACGTATGCGAACAACTTGAAACACAGGCTGTTGTATTGACGACCGTTGTACCATTATTAGTCACATCGTATAGAGACTCTCCACCATCATCCCATGTTTCCCACCCGATGGAAAACCGTCTTCTATCATCAACTGTGGTAAGATTTCGACCCTGCGAGGTATTCAGTTTCCCCATATCCATAACCTGGATTTTATCATTAAACCCCAATATTAAACACTTTTTTAGTACCCCCCACCC---------ACCAAAAAATGTGACTGAACCGGTTCCTAGCAGCTCTGGGAGCCAT

HAN33 ------TTAAGAAACCGAAACTCTCCACAGTTCACCATCCTCTTCGTTATTCAACCGATGACCCACTCCGTACAACGAATCAGTCTGCTGTGTCACATTGCAAACTACTAGCGACGTATGCGAACAACTTGAAACACAGGCTGTTGTATTGACGACCGTTGTACCATTATTAGTCACATCGTATAGAGACTCTCCACCGTCATCCCATGTCTCCCACCCGATGGAAAACCGTCTTCTATCATCAACTGTGGTAAGATTTCGACCCTGCGAGGTATTCAGTTTCCTCATATCCATAACCTGGATTTTATCATTAAACCCCAATATTAAACACTTTTTTAGTACCCCCCACCC---------ACCAAAAAATGTGACTGAACCGGTTCCTAGCAGCTCTGGGAGCCAT

JER3855 ------TTAAGAAACCGAAACTCTCGTCA--------------------------------------------------------------------------------------------------------------------------------------------------------------------CCACCGTCATCCCATGTTTCCCACCCGATGGAAAACCGTCTTCTATCATCAACTGTGGTAAGATTTCGACCCTGCGAGGTATTCAGTTTCCTCATATCCATAACCTGGATTTTATCATTAAACCCCAATATTAAACACTTTTTTAGTACCCCCCACCC---------ACCAAAAAATGTGACTGGACCGGTTCCTAGCAGCTCTGGGAGCCAT

AD169 ------TTAAGAAACCGAAACTCTCCACAGTTCACCATCTTCTTCGTCATTCAACCGATGACCCACTCGGTACAACGAATCAGTCTGCTGCGTCATATTGCAAAGCACAAGCGACGTATGCGAACAACTTGAAACACAGGCTGTTGTATTGACGACCGTTGTACCATTATTAGTCA-----------------CCACCGTTATCCCATGTTTCCCACCCGATGGAAAACCGTCTTCTATCATCAACTGTGGTAAGATTTCGACCCTGCGAGGTATTCAGTTTCCTCATATCCATAACCTGGATTTTATCATTAAACCCCAATATTAAACACTTTTTTAGTACCCCCCACCC---------ACCAAAAAATGTGACTGGACCGGTTCCTAGCAGCTCTGGGAGCCAT

HANSCTR2 ------TTAAGAAACCGAAACTCTCCACAGTTCACCATCTTCTTCGTCATTCAACCGATGACCCACTCCGTACAACGAATCAGTCTGCTGCGTCATATTGTAAAGCACAAGCGACGTATGCGAACAACTTGAAACACAGGCTGTTGTATTGACGACCGTTGTACCATTATTAGTCA-----------------CCACCGTCATCCCATGTTTCCCACCCGATGGAAAACCGTCTTCTATCATCAACTGTGGTAAGATTTCGACCCTGCGAGGTATTCAGTTTCCTCATATCCATAACCTGGATTTTATCATTAAACCCCAATATTAAACACTTTTTTAGTACCCCCCACCC---------ACCAAAAAATGTGACTGGACCGGTTCCTAGCAGCTCTGGGAGCCAT

PAV23 ------TTAAGAAACCGAAACTCTCCACAGTTCACCATCTTCTTCGTCATTCAACCGATGACCCACTCCGTACAACGAATCAGTCTGCTGCGTCATATTGCAAAGCACAAGCGACGTATGCGAACAACTTGAAACACAGGCTGTTGTATTGACGACCGTTGTACCATTATTAGTCA-----------------CCACCGTCATCCCATGTTTCCCACCCGATGGAAAACCGTCTTCTATCATCAACTGTGGTAAGATTTCGACCCTGCGAGGTATTCAGTTTCCTCATATCCATAACCTGGATTTTATCATTAAACCCCAATATTAAACACTTTTTTAGTACCCCCCACCC---------ACCAAAAAATGTGACTGGACCGGTTCCTAGCAGCTCTGGGAGCCAT

BE/49/2011 ------TTAAGAAACCGAAACTCTCCACAGTTCACCATCTTCTTCGTCATTCAACCGATGACCCACTCCGTACAACGAATCAGTCTGCTGCGTCATATTGCAAAGCACAAGCGACGTATGCGAACAACTTGAAACACAGGCTGTTGTATTGACGACCGTTGTACCATTATTAGTCA-----------------CCACCGTCATCCCATGTTTCCCACCCGATGGAAAACCGTCTTCTATCATCAACTGTGGTAAGATTTCGACCCTGCGAGGTATTCAGTTTCCTCATATCCATAACCTGGATTTTATCATTAAACCCCAATATTAAACACTTTTTTAGTACCCCCCACCC---------ACCAAAAAATGTGACTGGACCGGTTCCTAGCAGCTCTGGGAGCCAT

BE/21/2010 ------TTAAGAAACCGAAACTCTCCACAGTTCACCATCTTCTTCGTCATTCAACCGATGACCCACTCCGTACAACGAATCAGTCTGCTGCGTCATATTGCAAAGCACAAGCGACGTATGCGAACAACTTGAAACACAGGCTGTTGTATTGACGACCGTTGTACCATTATTAGTCA-----------------CCACCGTCATCCCATGTTTCCCACCCGATGGAAAACCGTCTTCTATCATCAACTGTGGTAAGATTTCGACCCTGCGAGGTATTCAGTTTCCTCATATCCATAACCTGGATTTTATCATTAAACCCCAATATTAAACACTTTTTTAGTACCCCCCACCC---------ACCAAAAAATGTGACTGGACCGGTTCCTAGCAGCTCTGGGAGCCAT

HAN13 ------TTAAGAAACCGAAACTCTCCACAGTTCACCATCTTCTTCGTCATTCAACCGATGACCCACTCCGTACAACGAATCAGTCTGCTGCGTCATATTGCAAAGCACAAGCGACGTATGCGAACAACTTGAAACACAGGCTGTTGTATTGACGACCGTTGTACCATTATTAGTCA-----------------CCACCGTCATCCCATGTTTCCCACCCGATGGAAAACCGTCTTCTATCATCAACTGTGGTAAGATTTCGACCCTGCGAGGTATTCAGTTTCCTCATATCCATAACCTGGATTTTATCATTAAACCCCAATATTAAACACTTTTTTAGTACCCCCCACCC---------ACCAAAAAATGTGACTGGACCGGTTCCTAGCAGCTCTGGGAGCCAT

PAV12 ------TTAAGAAACCGAAACTCTCCACAGTTCACCATCTTCTTCGTCATTCAACCGATGACCCACTCCGTACAACGAATCAGTCTGCTGCGTCATATTGCAAAGCACAAGCGACGTATGCGAACAACTTGAAACACAGGCTGTTGTATTGACGACCGTTGTACCATTATTAGTCA-----------------CCACCGTCATCCCATGTTTCCCACCCGATGGAAAACCGTCTTCTATCATCAACTGTGGTAAGATTTCGACCCTGCGAGGTATTCAGTTTCCTCATATCCATAACCTGGATTTTATCATTAAACCCCAATATTAAACACTTTTTTAGTACCCCCCACCC---------ACCAAAAAATGTGACTGGACCGGTTCCTAGCAGCTCTGGGAGCCAT

PAV1 ------TTAAGAAACCGAAACTCTCCACAGTTCACCATCTTCTTCGTCATTCAACCGATGACCCACTCCGTACAACGAATCAGTCTGCTGCGTCATATTGCAAAGCACAAGCGACGTATGCGAACAACTTGAAACACAGGCTGTTGTATTGACGACCGTTGTACCATTATTAGTCA-----------------CCACCGTCATCCCATGTTTCCCACCCGATGGAAAACCGTCTTCTATCATCAACTGTGGTAAGATTTCGACCCTGCGAGGTATTCAGTTTCCTCATATCCATAACCTGGATTTTATCATTAAACCCCAATATTAAACACTTTTTTAGTACCCCCCACCC---------ACCAAAAAATGTGACTGGACCGGTTCCTAGCAGCTCTGGGAGCCAT

PAV21 ------TTAAGAAACCGAAACTCTCCACAGTTCACCATCTTCTTCGTCATTCAACCGATGACCCACTCCGTACAACGAATCAGTCTGCTGCGTCATATTGCAAAGCACAAGCGACGTATGCGAACAACTTGAAACACAGGCTGTTGTATTGACGACCGTTGTACCATTATTAGTCA-----------------CCACCGTCATCCCATGTTTCCCACCCGATGGAAAACCGTCTTCTATCATCAACTGTGGTAAGATTTCGACCCTGCGAGGTATTCAGTTTCCTCATATCCATAACCTGGATTTTATCATTAAACCCCAATATTAAACACTTTTTTAGTACCCCCCACCC---------ACCAAAAAATGTGACTGGACCGGTTCCTAGCAGCTCTGGGAGCCAT

JER2002 ------TTAAGAAACCGAAACTCTCCACAGTTCACCATCTTCTTCGTCATTCAACCGATGACCCACTCCGTACAACGAATCAGTCTGCTGCGTCATATTGCAAAGCACAAGCGACGTATGCGAACAACTTGAAACACAGGCTGTTGTATTGACGACCGTTGTACCATTATTAGTCA-----------------CCACCGTCATCCCATGTTTCCCACCCGATGGAAAACCGTCTTCTATCATCAACTGTGGTAAGATTTCGACCCTGCGAGGTATTCAGTTTCCTCATATCCATAACCTGGATTTTATCATTAAACCCCAATATTAAACACTTTTTTAGTACCCCCCACCC---------ACCAAAAAATGTGACTGGACCGGTTCCTAGCAGCTCTGGGAGCCAT

Davis ------TTAAGAAACCGAAACTCTCCACAGTTCACCATCTTCTTCGTCATTCAACCGATGACCCACTCCGTACAACGAATCAGTCTGCTGCGTCATATTGCAAAGCACAAGCGACGTATGCGAACAACTTGAAACACAGGCTGTTGTATTGACGACCGTTGTACCATTATTAGTCA-----------------CCACCGTCATCCCATGTTTCCCACCCGATGGAAAACCGTCTTCTATCATCAACTGTGGTAAGATTTCGACCCTGCGAGGTATTCAGTTTCCTCATATCCATAACCTGGATTTTATCATTAAACCCCAATATTAAACACTTTTTTAGTACCCCCCACCC---------ACCAAAAAATGTGACTGGACCGGTTCCTAGCAGCTCTGGGAGCCAT

PAV31 ------TTAAGAAACCGAAACTCTCCACAGTTCACCATCTTCTTCGTCATTCAACCGATGACCCACTCCGTACAACGAATCAGTCTGCTGCGTCATATTGCAAAGCACAAGCGACGTATGCGAACAACTTGAAACACAGGCTGTTGTATTGACGACCGTTGTACCATTATTAGTCA-----------------CCACCGTCATCCCATGTTTCCCACCCGATGGAAAACCGTCTTCTATCATCAACTGTGGTAAGATTTCGACCCTGCGAGGTATTCAGTTTCCTCATATCCATAACCTGGATTTTATCATTAAACCCCAATATTAAACACTTTTTTAATACCCCCCACCC---------ACCAAAAAATGTGACTGGACCGGTTCCTAGCAGCTCTGGGAGCCAT

JER4559 ------TTAAGAAACCGAAACTCTCCACAGTTCACCATCTTCTTCGTCATTCAACCGATGACCCACTCCGTACAACGAATCAGTCTGCTGCGTCATATTGCAAAGCACAAGCGACGTATGCGAACAACTTGAAACACAGGCTGTTGTATTGATGACCGTTGTACCATTATTAGTCACATCGTATA----CTCTCCACCGTCATCCCATGTTTCCCACCCGATGGAAAACCGTCTTCTATCATCAACTGTGGTAAGATTTCAACCCTGCGAGGTATTCAGTTTCCTCATATCCATAACCTGGATTTTATCATTAAACCCCAATATTAAACACTTTTTTAGTACCCCCCACCC---------ACCAAAAAATGTGACTGGACCGGTTCCTAGCAGCTCTGGGAGCCAT

BE/15/2011 ------TTAAGAAACCGAAACTCTCCACAGTTCACCATCTTCTTCGTCATTCAACCGATGACCCACTCCGTACAACGAATCAGTCTGCTGCGTCATATTGTAAAGCACAAGCGACGTATGCGAACAACTTGAAACACAGGCTGTTGTATTGATGACCGTTGTACCATTATTAGTCACATCGTATAGAGACTATCCACCGTCATCCCATGTTTCCCACCCGATGGAAAACCGTCTTCTATCATCAACTGTGGTAAGATTTCGACCCTGCGAGGTATTCAGTTTCCTCATATCCATAACCTGGATTTTATCATTAAACCCCAATATTAAACACTTTTTTAGTACCCCCCACCC---------ACCAAAAAATGTGACTGGACCGGTTCCTAGCAGCTCTGGGAGCCAT

Pat_H ------TTAAGAAACCGAAACTCTCCACAGTTCACCATCTTCTTCGTCATTCAACCGATGACCCACTCCGTACAACGAATCAGTCTGCTGCGTCATATTGCAAAGCACAAGCGACGTATGCGAACAACTTGAAACACAGGCTGTTGTATTGACGACCGTTGTACCATTATTAGTCACATCGTATAGAGACTCTCCACCGTCATCCCATGTTTCCCACCCGATGGAAAACCGTCTTCTATCATCAACTGTGGTAAGATTTCAACCCTGCGAGGTATTCAGTTTCCTCATATCCATAACCTGGATTTTATCATTAAACCCCAATATTAAACACTTTTTTAGTACCCCCCACCC---------ACCAAAAAATGTGACTGGACCGGTTCCTAGCAGCTCTGGGAGCCAT

U11 ------TTAAGAAACCGAAACTCTCCACAGTTCACCATCTTCTTCGTCATTCAACCGATGACCCACTCCGTACAACGAATCAGTCTGCTGCGTCATATTGCAAAGCACAAGCGACGTATGCGAACAACTTGAAACACAGGCTGTTGTATTGACGACCGTTGTACCATTATTAGTCACATCGTATAGAGACTCTCCACCGTCATCCCATGTTTCCCACCCGATGGAAAACCGTCTTCTATCACCAACTGTGGTAAGATTTCGACCCTGCGAGGTATTCAGTTTCCTCATATCCATAACCTGGATTTTATCATTAAACCCCAATATTAAACACTTTTTTAGTACCCCCCACCC---------ACCAAAAAATGTGACTGGACCGGTTCCTAGCAGCTCTGGGAGCCAT

PAV11 ------TTAAGAAACCGAAACTCTCCACAGTTCACCATCTTCTTCGTCATTCAACCGATGACCCACTCCGTACAACGAATCAGTCTGCTGCGTCATATTGCAAAGCACAAGCGACGTATGCGAACAACTTGAAACACAGGCTGTTGTATTGATGACCGTTGTACCATTATTAGTCACATCGTATAGAGACTCTCCACCGTCATCCCATGTTTCCCACCCGATGGAAAACCGTCTTCTATCATCAACTGTGGTAAGATTTCGACCCTGCGAGGTATTCAGTTTCCTCATATCCATAACCTGGATTTTATCATTAAACCCCAATATTAAATACTTTTTTAGTACCCCCCACCC---------ACCAAAAAATGTGACTGGACCGGTTCCTAGCAGCTCTGGGAGCCAT

VR1814 ------TTAAGAAACCGAAACTCTCCACAGTTCACCATCTTCTTCGTCATTCAACCGATGACCCACTCCGTACAACGAATCAGTCTGCTGCGTCATATTGCAAAGCACAAGCGACGTATGCGAACAACTTGAAACACAGGCTGTTGTATTGATGACCGTTGTACCATTATTAGTCACATCGTATAGAGACTCTCCACCGTCATCCCATGTTTCCCACCCGATGGAAAACCGTCTTCTATCATCAACTGTGGTAAGATTTCGACCCTGCGAGGTATTCAGTTTCCTCATATCCATAACCTGGATTTTATCATTAAACCCCAATATTAAATACTTTTTTAGTACCCCCCACCC---------ACCAAAAAATGTGACTGGACCGGTTCCTAGCAGCTCTGGGAGCCAT

HAN30 ------TTAAGAAACCGAAACTCTCCACAGTTCACCATCTTCTTCGTCATTCAACCGATGACCCACTCCGTACAACGAATCAGTCTGCTGCGTCATATTGCAAAGCACAAGCGACGTATGCGAACAACTTGAAACACAGGCTGTTGTATTGATGACCGTTGTACCATTATTAGTCACATCGTATAGAGACTCTCCACCGTCATCCCATGTTTCCCACCCGATGGAAAACCGTCTTCTATCATCAACTGTGGTAAGATTTCGACCCTGCGAGGTATTCAGTTTCCTCATATCCATAACCTGGATTTTATCATTAAACCCCAATATTAAATACTTTTTTAGTACCCCCCACCC---------ACCAAAAAATGTGACTGGACCGGTTCCTAGCAGCTCTGGGAGCCAT

Merlin ------TTAAGAAACCGAAACTCTCCACAGTTCACCATCTTCTTCGTCATTCAACCGATGACCCACTCCGTACAACGAATCAGTCTGCTGCGTCATATTGCAAAGCACAAGCGACGTATGCGAACAACTTGAAACACAGGCTGTTGTATTGATGACCGTTGTACCATTATTAGTCACATCGTATAGAGACTCTCCACCGTCATCCCATGTTTCCCACCCGATGGAAAACCGTCTTCTATCATCAACTGTGGTAAGATTTCGACCCTGCGAGGTATTCAGTTTCCTCATATCCATAACCTGGATTTTATCATTAAACCCCAATATTAAATACTTTTTTAGTACCCCCCACCC---------ACCAAAAAATGTGACTGGACCGGTTCCTAGCAGCTCTGGGAGCCAT

CZ/1/2013 ------TTAAGAAACCGAAACTCTCCACAGTTCACCATCTTCTTCGTCATTCAACCGATGACCCACTCCGTACAACGAATCAGTCTGCTGCGTCATATTGCAAAGCACAAGCGACGTATGCGAACAACTTGAAACACAGGCTGTTGTATTGATGACCGTTGTACCATTATTAGTCACATCGTATAGAGACTCTCCACCGTCATCCCATGTTTCCCACCCGATGGAAAACCGTCTTCTATCATCAACTGTGGTAAGATTTCGACCCTGCGAGGTATTCAGTTTCCTCATATCCATAACCTGGATTTTATCATTAAACCCCAATATTAAATACTTTTTTAGTACCCCCCACCC---------ACCAAAAAATGTGACTGGACCGGTTCCTAGCAGCTCTGGGAGCCAT

BE/23/2010 ------TTAAGAAACCGAAACTCTCCACAGTTCACCATCTTCTTCGTCATTCAACCGATGACCCACTCCGTACAACGAATCAGTCTGCTGCGTCATATTGCAAAGCACAAGCGACGTATGCGAACAACTTGAAACACAGGCTGTTGTATTGATGACCGTTGTACCATTATTAGTCACATCGTATAGAGACTCTCCACCGTCATCCCATGTTTCCCACCCGATGGAAAACCGTCTTCTATCATCAACTGTGGTAAGATTTCGACCCTGCGAGGTATTCAGTTTCCTCATATCCATAACCTGGATTTTATCATTAAACCCCAATATTAAATACTTTTTTAGTACCCCCCACCC---------ACCAAAAAATGTGACTGGACCGGTTCCTAGCAGCTCTGGGAGCCAT

NL/Rot7 ------TTAAGAAACCGAAACTCTCCACAGTTCACCATCTTCTTCGTCATTCAACCGATGACCCACTCCGTACAACGAATCAGTCTGCTGCGTCATATTGCAAAGCACAAGCGACGTATGCGAACAACTTGAAACACAGGCTGTTGTATTGATGACCGTTGTACCATTATTAGTCACATCGTATAGAGACTCTCCACCGTCATCCCATGTTTCCCACCCGATGGAAAACCGTCTTCTATCATCAACTGTGGTAAGATTTCGACCCTGCGAGGTATTCAGTTTCCTCATATCCATAACCTGGATTTTATCATTAAACCCCAATATTAAATACTTTTTTAGTACCCCCCACCC---------ACCAAAAAATGTGACTGGACCGGTTCCTAGCAGCTCTGGGAGCCAT

UK/Lon3 ------TTAAGAAACCGAAACTCTCCACAGTTCACCATCTTCTTCGTCATTCAACCGATGACCCACTCCGTACAACGAATCAGTCTGCTGCGTCATATTGCAAAGCACAAGCGACGTATGCGAACAACTTGAAACACAGGCTGTTGTATTGATGACCGTTGTACCATTATTAGTCACATCGTATAGAGACTCTCCACCGTCATCCCATGTTTCCCACCCGATGGAAAACCGTCTTCTATCATCAACTGTGGTAAGATTTCGACCCTGCGAGGTATTCAGTTTCCTCATATCCATAACCTGGATTTTATCATTAAACCCCAATATTAAATACTTTTTTAGTACCCCCCACCC---------ACCAAAAAATGTGACTGGACCGGTTCCTAGCAGCTCTGGGAGCCAT

HANChild4 ------TTAAGAAACCGAAACTCTCCACAGTTCACCATCTTCTTCGTCATTCAACCGATGACCCACTCCGTACAACGAATCAGTCTGCTGCGTCATATTGCAAAGCACAAGCGACGTATGCGAACAACTTGAAACACAGGCTGTTGTATTGATGACCGTTGTACCATTATTAGTCACATCGTATAGAGACTCTCCACCGTCATCCCATGTTTCCCACCCGATGGAAAACCGTCTTCTATCATCAACTGTGGTAAGATTTCGACCCTGCGAGGTATTCAGTTTCCTCATATCCATAACCTGGATTTTATCATTAAACCCCAATATTAAATACTTTTTTAGTACCCCCCACCC---------ACCAAAAAATGTGACTGGACCGGTTCCTAGCAGCTCTGGGAGCCAT

PRA4 ------TTAAGAAACCGAAACTCTCCACAGTTCACCATCTTCTTCGTCATTCAACCGATGACCCACTCCGTACAACGAATCAGTCTGCTGCGTCATATTGCAAAGCACAAGCGACGTATGCGAACAACTTGAAACACAGGCTGTTGTATTGATGACCGTTGTACCATTATTAGTCACATCGTATAGAGACTCTCCACCGTCATCCCATGTTTCCCACCCGATGGAAAACCGTCTTCTATCATCAACTGTGGTAAGATTTCGACCCTGCGAGGTATTCAGTTTCCTCATATCCATAACCTGGATTTTATCATTAAACCCCAATATTAAATACTTTTTTAGTACCCCCCACCC---------ACCAAAAAATGTGACTGGACCGGTTCCTAGCAGCTCTGGGAGCCAT

HANRTR1B ------TTAAGAAACCGAAACTCTCCACAGTTCACCATCTTCTTCGTCATTCAACCGATGACCCACTCCGTACAACGAATCAGTCTGCTGCGTCATATTGCAAAGCACAAGCGACGTATGCGAACAACTTGAAACACAGGCTGTTGTATTGATGACCGTTGTACCATTATTAGTCACATCGTATAGAGACTCTCCACCGTCATCCCATGTTTCCCACCCGATGGAAAACCGTCTTCTATCATCAACTGTGGTAAGATTTCGACCCTGCGAGGTATTCAGTTTCCTCATATCCATAACCTGGATTTTATCATTAAACCCCAATATTAAATACTTTTTTAGTACCCCCCACCC---------ACCAAAAAATGTGACTGGACCGGTTCCTAGCAGCTCTGGGAGCCAT

2CEN2 ------TTAAGAAACCGAAACTCTCCACAGTTCACCATCTTCTTCGTCATTCAACCGATGACCCACTCCGTACAACGAATCAGTCTGCTGCGTCATATTGCAAAGCACAAGCGACGTATGCGAACAACTTGAAACACAGGCTGTTGTATTGACGACCGTTGTACCATTATTAGTCACATCGTATAGAGACTCTCCACCGTCATCCCATGTTTCCCACCCGATGGAAAACCGTCTTCTATCATCAACTGTGGTAAGATTTCGACCCTGCGAGGTATTCAGTTTCCTCATATCCATAACCTGGATTTTATCATTAAACCCCAATATTAAACACTTTTTTAGTACCCCCCACCC---------ACCAAAAAATGTGACTGGACCGGTTCCTAGCAGCTCTGGGAGCCAT

BE/16/2010 ------TTAAGAAACCGAAACTCTCCACAGTTCACCATCTTCTTCGTCATTCAACCGATGACCCACTCCGTACAACGAATCAGTCTGCTGCGTCATATTGCAAAGCACAAGCGACGTATGCGAACAACTTGAAACACAGGCTGTTGTATTGACGACCGTTGTACCATTATTAGTCACATCGTATAGAGACTCTCCACCGTCATCCCATGTTTCCCACCCGATGGAAAACCGTCTTCTATCATCAACTGTGGTAAGATTTCGACCCTGCGAGGTATTCAGTTTCCTCATATCCATAACCTGGATTTTATCATTAAACCCCAATATTAAACACTTTTTTAGTACCCCCCACCC---------ACCAAAAAATGTGACTGGACCGGTTCCTAGCAGCTCTGGGAGCCAT

HAN2 ------TTAAGAAACCGAAACTCTCCACAGTTCACCATCTTCTTCGTCATTCAACCGATGACCCACTCCGTACAACGAATCAGTCTGCTGCGTCATATTGCAAAGCACAAGCGACGTATGCGAACAACTTGAAACACAGGCTGTTGTATTGACGACCGTTGTACCATTATTAGTCACATCGTATAGAGACTCTCCACCGTCATCCCATGTTTCCCACCCGATGGAAAACCGTCTTCTATCATCAACTGTGGTAAGATTTCGACCCTGCGAGGTATTCAGTTTCCTCATATCCATAACCTGGATTTTATCATTAAACCCCAATATTAAACACTTTTTTAGTACCCCCCACCC---------ACCAAAAAATGTGACTGGACCGGTTCCTAGCAGCTCTGGGAGCCAT

JER893 ------TTAAGAAACCGAAACTCTCCACAGTTCACCATCTTCTTCGTCATTCAACCGATGACCCACTCCGTACAACGAATCAGTCTGCTGCGTCATATTGCAAAGCACAAGCGACGTATGCGAACAACTTGAAACACAGGCTGTTGTATTGACGACCGTTGTACCATTATTAGTCACATCGTATAGAGACTCTCCACCGTCATCCCATGTTTCCCACCCGATGGAAAACCGTCTTCTATCATCAACTGTGGTAAGATTTCGACCCTGCGAGGTATTCAGTTTCCTCATATCCATAACCTGGATTTTATCATTAAACCCCAATATTAAACACTTTTTTAGTACCCCCCACCC---------ACCAAAAAATGTGACTGGACCGGTTCCTAGCAGCTCTGGGAGCCAT

CZ/1/2011 ------TTAAGAAACCGAAACTCTCCACAGTTCACCATCTTCTTCGTCATTCAACCGATGACCCACTCCGTACAACGAATCAGTCTGCTGCGTCATATTGCAAAGCACAAGCGACGTATGCGAACAACTTGAAACACAGGCTGTTGTATTGACGACCGTTGTACCATTATTAGTCACATCGTATAGAGACTCTCCACCGTCATCCCATGTTTCCCACCCGATGGAAAACCGTCTTCTATCATCAACTGTGGTAAGATTTCGACCCTGCGAGGTATTCAGTTTCCTCATATCCATAACCTGGATTTTATCATTAAACCCCAATATTAAACACTTTTTTAGTACCCCCCACCC---------ACCAAAAAATGTGACTGGACCGGTTCCTAGCAGCTCTGGGAGCCAT

BE/10/2010 ------TTAAGAAACCGAAACTCTCCACAGTTCACCATCTTCTTCGTCATTCAACCGATGACCCACTCCGTACAACGAATCAGTCTGCTGCGTCATATTGCAAAGCACAAGCGACGTATGCGAACAACTTGAAACACAGGCTGTTGTATTGACGACCGTTGTACCATTATTAGTCACATCGTATAGAGACTCTCCACCGTCATCCCATGTTTCCCACCCGATGGAAAACCGTCTTCTATCATCAACTGTGGTAAGATTTCGACCCTGCGAGGTATTCAGTTTCCTCATATCCATAACCTGGATTTTATCATTAAACCCCAATATTAAACACTTTTTTAGTACCCCCCACCC---------ACCAAAAAATGTGACTGGACCGGTTCCTAGCAGCTCTGGGAGCCAT

PRA3 ------TTAAGAAACCGAAACTCTCCACAGTTCACCATCTTCTTCGTCATTCAACCGATGACCCACTCCGTACAACGAATCAGTCTGCTGCGTCATATTGCAAAGCACAAGCGACGTATGCGAACAACTTGAAACACAGGCTGTTGTATTGACGACCGTTGTACCATTATTAGTCACATCGTATAGAGACTCTCCACCGTCATCCCATGTTTCCCACCCGATGGAAAACCGTCTTCTATCATCAACTGTGGTAAGATTTCGACCCTGCGAGGTATTCAGTTTCCTCATATCCATAACCTGGATTTTATCATTAAACCCCAATATTAAACACTTTTTTAGTACCCCCCACCC---------ACCAAAAAATGTGACTGGACCGGTTCCTAGCAGCTCTGGGAGCCAT

PRA5 ------TTAAGAAACCGAAACTCTCCACAGTTCACCATCTTCTTCGTCATTCAACCGATGACCCACTCCGTACAACGAATCAGTCTGCTGCGTCATATTGCAAAGCACAAGCGACGTATGCGAACAACTTGAAACACAGGCTGTTGTATTGACGACCGTTGTACCATTATTAGTCACATCGTATAGAGACTCTCCACCGTCATCCCATGTTTCCCACCCGATGGAAAACCGTCTTCTATCATCAACTGTGGTAAGATTTCGACCCTGCGAGGTATTCAGTTTCCTCATATCCATAACCTGGATTTTATCATTAAACCCCAATATTAAACACTTTTTTAGTACCCCCCACCC---------ACCAAAAAATGTGACTGGACCGGTTCCTAGCAGCTCTGGGAGCCAT

HANRTR10 ------TTAAGAAACCGAAACTCTCCACAGTTCACCATCTTCTTCGTCATTCAACCGATGACCCACTCCGTACAACGAATCAGTCTGCTGCGTCATATTGCAAAGCACAAGCGACGTATGCGAACAACTTGAAACACAGGCTGTTGTATTGACGACCGTTGTACCATTATTAGTCACATCGTATAGAGACTCTCCACCGTCATCCCATGTTTCCCACCCGATGGAAAACCGTCTTCTATCATCAACTGTGGTAAGATTTCGACCCTGCGAGGTATTCAGTTTCCTCATATCCATAACCTGGATTTTATCATTAAACCCCAATATTAAACACTTTTTTAGTACCCCCCACCC---------ACCAAAAAATGTGACTGGACCGGTTCCTAGCAGCTCTGGGAGCCAT

HANRTR4 ------TTAAGAAACCGAAACTCTCCACAGTTCACCATCTTCTTCGTCATTCAACCGATGACCCACTCCGTACAACGAATCAGTCTGCTGCGTCATATTGCAAAGCACAAGCGACGTATGCGAACAACTTGAAACACAGGCTGTTGTATTGACGACCGTTGTACCATTATTAGTCACATCGTATAGAGACTCTCCACCGTCATCCCATGTTTCCCACCCGATGGAAAACCGTCTTCTATCATCAACTGTGGTAAGATTTCGACCCTGCGAGGTATTCAGTTTCCTCATATCCATAACCTGGATTTTATCATTAAACCCCAATATTAAACACTTTTTTAGTACCCCCCACCC---------ACCAAAAAATGTGACTGGACCGGTTCCTAGCAGCTCTGGGAGCCAT

HANSCTR8 ------TTAAGAAACCGAAACTCTCCACAGTTCACCATCTTCTTCGTCATTCAACCGATGACCCACTCCGTACAACGAATCAGTCTGCTGCGTCATATTGCAAAGCACAAGCGACGTATGCGAACAACTTGAAACACAGGCTGTTGTATTGACGACCGTTGTACCATTATTAGTCACATCGTATAGAGACTCTCCACCGTCATCCCATGTTTCCCACCCGATGGAAAACCGTCTTCTATCATCAACTGTGGTAAGATTTCGACCCTGCGAGGTATTCAGTTTCCTCATATCCATAACCTGGATTTTATCATTAAACCCCAATATTAAACACTTTTTTAGTACCCCCCACCC---------ACCAAAAAATGTGACTGGACCGGTTCCTAGCAGCTCTGGGAGCCAT

HANRTR9 ------TTAAGAAACCGAAACTCTCCACAGTTCACCATCTTCTTCGTCATTCAACCGATGACCCACTCCGTACAACGAATCAGTCTGCTGCGTCATATTGCAAAGCACAAGCGACGTATGCGAACAACTTGAAACACAGGCTGTTGTATTGACGACCGTTGTACCATTATTAGTCACATCGTATAGAGACTCTCCACCGTCATCCCATGTTTCCCACCCGATGGAAAACCGTCTTCTATCATCAACTGTGGTAAGATTTCGACCCTGCGAGGTATTCAGTTTCCTCATATCCATAACCTGGATTTTATCATTAAACCCCAATATTAAACACTTTTTTAGTACCCCCCACCC---------ACCAAAAAATGTGACTGGACCGGTTCCTAGCAGCTCTGGGAGCCAT

BE/22/2011 ------TTAAGAAACCGAAACTCTCCACAGTTCACCATCTTCTTCGTCATTCAACCGATGACCCACTCCGTACAACGAATCAGTCTGCTGCGTCATATTGCAAAGCACAAGCGACGTATGCGAACAACTTGAAACACAGGCTGTTGTATTGATGACCGTTGTACCATTATTAGTCACATCGTATAGAGACTCTCCACCGTCATCCCATGTTTCCCACCCGATGGAAAACCGTCTTCTATCATCAACTGTGGTAAGATTTCGACCCTGCGAGGTATTCAGTTTCCTCATATCCATAACCTGGATTTTATCATTAAACCCCAATATTAAACACTTTTTTAGTACCCCCCACCC---------ACCAAAAAATGTGACTGGACCGGTTCCTAGCAGCTCTGGGAGCCAT

AF1 ------TTAAGAAACCGAAACTCTCCACAGTTCACCATCTTCTTCGTCATTCAACCGATGACCCACTCCGTACAACGAATCAGTCTGCTGCGTCATATTGCAAAGCACAAGCGACGTATGCGAACAACTTGAAACACAGGCTGTTGTATTGATGACCGTTGTACCATTATTAGTCACATCGTATAGAGACTCTCCACCGTCATCCCATGTTTCCCACCCGATGGAAAACCGTCTTCTATCATCAACTGTGGTAAGATTTCGACCCTGCGAGGTATTCAGTTTCCTCATATCCATAACCTGGATTTTATCATTAAACCCCAATATTAAACACTTTTTTAGTACCCCCCACCC---------ACCAAAAAATGTGACTGGACCGGTTCCTAGCAGCTCTGGGAGCCAT

BE/1/2012 ------TTAAGAAACCGAAACTCTCCACAGTTCACCATCTTCTTCGTCATTCAACCGATGACCCACTCCGTACAACGAATCAGTCTGCTGCGTCATATTGCAAAGCACAAGCGACGTATGCGAACAACTTGAAACACAGGCTGTTGTATTGATGACCGTTGTACCATTATTAGTCACATCGTATAGAGACTCTCCACCGTCATCCCATGTTTCCCACCCGATGGAAAACCGTCTTCTATCATCAACTGTGGTAAGATTTCGACCCTGCGAGGTATTCAGTTTCCTCATATCCATAACCTGGATTTTATCATTAAACCCCAATATTAAACACTTTTTTAGTACCCCCCACCC---------ACCAAAAAATGTGACTGGACCGGTTCCTAGCAGCTCTGGGAGCCAT

HAN24 ------TTAAGAAACCGAAACTCTCCACAGTTCACCATCTTCTTCGTCATTCAACCGATGACCCACTCCGTACAACGAATCAGTCTGCTGCGTCATATTGCAAAGCACAAGCGACGTATGCGAACAACTTGAAACACAGGCTGTTGTATTGATGACCGTTGTACCATTATTAGTCACATCGTATAGAGACTCTCCACCGTCATCCCATGTTTCCCACCCGATGGAAAACCGTCTTCTATCATCAACTGTGGTAAGATTTCGACCCTGCGAGGTATTCAGTTTCCTCATATCCATAACCTGGATTTTATCATTAAACCCCAATATTAAACACTTTTTTAGTACCCCCCACCC---------ACCAAAAAATGTGACTGGACCGGTTCCTAGCAGCTCTGGGAGCCAT

BE/4/2011 ------TTAAGAAACCGAAACTCTCCACAGTTCACCATCTTCTTCGTCATTCAACCGATGACCCACTCCGTACAACGAATCAGTCTGCTGCGTCATATTGCAAAGCACAAGCGACGTATGCGAACAACTTGAAACACAGGCTGTTGTATTGATGACCGTTGTACCATTATTAGTCACATCGTATAGAGACTCTCCACCGTCATCCCATGTTTCCCACCCGATGGAAAACCGTCTTCTATCATCAACTGTGGTAAGATTTCGACCCTGCGAGGTATTCAGTTTCCTCATATCCATAACCTGGATTTTATCATTAAACCCCAATATTAAACACTTTTTTAGTACCCCCCACCC---------ACCAAAAAATGTGACTGGACCGGTTCCTAGCAGCTCTGGGAGCCAT

Towne ------TTAAGAAACCGAAACTCTCCACAGTTCACCATCTTCTTCGTCATTCAACCGATGACCCACTCCGTACAACGAATCAGTCTGCTGCGTCATATTGCAAAGCACAAGCGACGTATGCGAACAACTTGAAACACAGGCTGTTGTATTGATGACCGTTGTACCATTATTAGTCACATCGTATAGAGACTCTCCACCGTCATCCCATGTTTCCCACCCGATGGAAAACCGTCTTCTATCATCAACTGTGGTAAGATTTCGACCCTGCGAGGTATTCAGTTTCCTCATATCCATAACCTGGATTTTATCATTAAACCCCAATATTAAACACTTTTTTAGTACCCCCCACCC---------ACCAAAAAATGTGACTGGACCGGTTCCTAGCAGCTCTGGGAGCCAT

6397 ------TTAAGAAACCGAAACTCTCCACAGTTCACCATCTTCTTCGTCATTCAACCGATGACCCACTCCGTACAACGAATCAGTCTGCTGCGTCATATTGCAAAGCACAAGCGACGTATGCGAACAACTTGAAACACAGGCTGTTGTATTGATGACCGTTGTACCATTATTAGTCACATCGTATAGAGACTCTCCACCGTCATCCCATGTTTCCCACCCGATGGAAAACCGTCTTCTATCATCAACTGTGGTAAGATTTCGACCCTGCGAGGTATTCAGTTTCCTCATATCCATAACCTGGATTTTATCATTAAACCCCAATATTAAACACTTTTTTAGTACCCCCCACCC---------ACCAAAAAATGTGACTGGACCGGTTCCTAGCAGCTCTGGGAGCCAT

JER847 ------TTAAGAAACCGAAACTCTCCACAGTTCACCATCTTCTTCGTCATTCAACCGATGACCCACTCCGTACAACGAATCAGTCTGCTGCGTCATATTGCAAAGCACAAGCGACGTATGCGAACAACTTGAAACACAGGCTGTTGTATTGATGACCGTTGTACCATTATTAGTCACATCGTATAGAGACTCTCCACCGTCATCCCATGTTTCCCACCCGATGGAAAACCGTCTTCTATCATCAACTGTGGTAAGATTTCGACCCTGCGAGGTATTCAGTTTCCTCATATCCATAACCTGGATTTTATCATTAAACCCCAATATTAAACACTTTTTTAGTACCCCCCACCC---------ACCAAAAAATGTGACTGGACCGGTTCCTAGCAGCTCTGGGAGCCAT

NAN4LA ------TTAAGAAACCGAAACTCTCCACAGTTCACCATCTTCTTCGTCATTCAACCGATGACCCACTCCGTACAACGAATCAGTCTGCTGCGTCATATTGCAAAGCACAAGCGACGTATGCGAACAACTTGAAACACAGGCTGTTGTATTGATGACCGTTGTACCATTATTAGTCACATCGTATAGAGACTCTCCACCGTCATCCCATGTTTCCCACCCGATGGAAAACCGTCTTCTATCATCAACTGTGGTAAGATTTCGACCCTGCGAGGTATTCAGTTTCCTCATATCCATAACCTGGATTTTATCATTAAACCCCAATATTAAACACTTTTTTAGTACCCCCCACCC---------ACCAAAAAATGTGACTGGACCGGTTCCTAGCAGCTCTGGGAGCCAT

HANSCTR4 ------TTAAGAAACCGAAACTCTCCACAGTTCACCATCTTCTTCGTCATTCAACCGATGACCCACTCCGTACAACGAATCAGTCTGCTGCGTCATATTGCAAAGCACAAGCGACGTATGCGAACAACTTGAAACACAGGCTGTTGTATTGATGACCGTTGTACCATTATTAGTCACATCGTATAGAGACTCTCCACCGTCATCCCATGTTTCCCACCCGATGGAAAACCGTCTTCTATCATCAACTGTGGTAAGATTTCGACCCTGCGAGGTATTCAGTTTCCTCATATCCATAACCTGGATTTTATCATTAAACCCCAATATTAAACACTTTTTTAGTACCCCCCACCC---------ACCAAAAAATGTGACTGGACCGGTTCCTAGCAGCTCTGGGAGCCAT

**RL6**

Initiation codons (the first and, where present, second ATG codons) and termination codons are highlighted in yellow. Sequences used for motif-matching are indicated in blue font (identifying genotypes) or red font (identifying a common recombinant, with the region of recombination highlighted in grey).

Cyan highlighting indicates strains in which this gene is mutated and the mutations responsible: substitutions that introduce in-frame stop codons (underlined) or insertions or deletions that cause frameshifts (the C-tract towards the right end of the alignment is not included). The great majority of the gene is absent from G5.

The scattered locations of second ATG codons suggests that the true initiation codon for all genotypes is the first one. If this is the case, many more strains are mutated because of frameshifts in the C-tract, perhaps indicating that RL6 is essentially a nonfunctional gene that is in the process of being lost.

BE/29/2010 G1 TTAGCTGACTTCCAAGTGCCACACATCACCACTGTATTCATCCATGTTTTCACCGAACCAACGAGACAGATCGAAGAAGCCAGAATCTCCCGACTTTAAATTACATAATTCCAACGTATTATGACCACAGCTCGACACACAAATAGTTGCGTTACTATTCACAGTAGCATTACCTATACCCGTAACGTTGCACAACCACTGATCACCATTGTTACCAAAAACGGTTTTCCACTTAGTTGTCAACGGATCTTTCCCATGCGTAATGGTCAAATTACTACCAGTCGTCGCTTTTAGCTCATTACGAGTATTATCCGCATCCACATATATCAACGTCATAGCTAGGCACGCTATAAGTACCCCCCCCCCCCCACACAATGGAATGTTGCCAAACCGGTTCTTTCCCGTTATAGCCAT

HAN3 TTAGCTGACTTCCAAGTACCACACATCACCACTGTATTCATCCATGTTTTCACCGAACCAACGAGACAGATCGAAGAAGCCAGAATCTCCCGACTTTAAATTACATAAATCCAACGTATTATGACCACAGCTCGACACACAAATAGTTGCGTTACTATTCACAGTAGCATTACCTATACCCGTGACGTTGCACAACCACTGATCACCATTGTTACCAAAAACGGTTTTCCACTTAGTTGTCAACGGATCTTTCCTATGCGTAATGGTTAAATTACTACCAGTCGTCGCTTTTAGCTCATTACGAGTATTATCCGCATCCACATATATCAACGTCATAGCTAGGCACGCTATAAGTACCCCCCCCCC-----ACAATGGAATGCTGCCAAACCGGTTATTTCCCGTTATAGCCAT

NL/Rot52 TTAGCTGACTTCCAAGTACCACACATCACCACTGTATTCATCCATGTTTTCACCGAACCAACGAGACAGATCGAAGAAGCCAGAATCTCCCGACTTTAAATTACATAAATCCAACGTATTATGACCACAGCTCGACACACAAATAGTTGCGTTACTATTCACAGTAGCATTACCTATACCCGTGACGTTGCACAACCACTGATCACCATTGTTACCAAAAACGGTTTTCCACTTAGTTGTCAACGGATCTTTCCTATGCGTAATGGTTAAATTACTACCAGTCGTCGCTTTTAGCTCATTACGAGTATTATCCGCATCCACATATATCAACGTCATAGCTAGGCACGCTATAAGTACCCCCCCCCC-----ACAATGGAATGCTGCCAAACCGGTTATTTCCCGTTATAGCCAT

BE/1/2011 TTAGCTGACTTCCAAGTACCACACATCACCACTGTATTCATCCATGTTTTCACCGAACCAACGAGACAGATCGAAGAAGCCAGAATCTCCCGACTTTAAATTACATAAATCCAACGTATTATGACCACAGCTCGACACACAAATAGTTGCGTTACTATTCACAGTAGCATCACCTATACCCGTAACGTTGCACAACCACTGATCACCATTGTTACCAAAAACGGTTTTCCACTTAGTTGTCAACGGATCTTTCCTATGCGTAATGGTAAAATTACTACCAGTCGTCGCTTTTAGCTCATTACGAGTATTATCCGCATCCACATATATCAACGTCATAGCTAGGCACGCTATAAGTACCCCCCCCCC-----ACAATGGAATGCTGCCAAACCGGTTATTTCCCGTTATAGCCAT

BE/9/2012 TTAGCTGACTTCCAAGTACCACACATCACCACTGTATTCATCCATGTTTTCACCGAACCAACGAGACAGATCGAAGAAGCCAGAATCTCCCGACTTTAAATTACATAAATCCAACGTATTATGACCACAGCTCGACACACAAATAGTTGCGTTACTATTCACAGTAGCATCACCTATACCCGTAACGTTGCACAACCACTGATCACCATTGTTACCAAAAACGGTTTTCCACTTAGTTGTCAACGGATCTTTCCTATGCGTAATGGTAAAATTACTACCAGTCGTCGCTTTTAGCTCATTACGAGTATTATCCGCATCCACATATATCAACGTCATAGCTAGGCACGCTATAAGTACCCCCCCCCC-----ACAATGGAATGCTGCCAAACCGGTTATTTCCCGTTATAGCCAT

BE/8/2010 TTAGCTGACTTCCAAGTACCACACATCACCACTGTATTCATCCATGTTTTCACCGAACCAACGAGACAGATCGAAGAAGCCAGAATCTCCCGACTTTAAATTACATAAATCCAACGTATTATGACCACAGCTCGACACACAAATAGTTGCGTTACTATTCACAGTAGCATCACCTATACCCGTAACGTTGCACAACCACTGATCACCATTGTTACCAAAAACGGTTTTCCACTTAGTTGTCAACGGATCTTTCCTATGCGTAATGGTAAAATTACTACCAGTCGTCGCTTTTAGCTCATTACGAGTATTATCCGCATCCACATATATCAACGTCATAGCTAGGCACGCTATAAGTACCCCCCCCCC-----ACAATGGAATGCTGCCAAACCGGTTATTTCCCGTTATAGCCAT

BE/8/2011 TTAGCTGACTTCCAAATGCCACACATCACCACTGTATTCATCCATATTTTCACCGAACCAACGAGACAGATCGAAGAAGCCAGAATCTCCCGACTTTAGATTACATAAATCCAACGTATTATGACCACAGCTCGACACACAAATAGTTGCGTTACTATTCACAGTAGCATTACCTATACCCGTAACGTTGCACAACCACTGATCACCATTGTTACCAAAAACGGTTTTCCACTTAGTTGTCAACGGATCTTTCCTATGCGTAATGGTAAAATTACTACCACTCGTCGCTTTTAGCTCATTACGAGTATTATCCGCATCCACATATATCAACGTCATAGCTAGGCACGCTATAAGTACCCCCCCCCC-----ACAATGGAATGTTGCCGAACCGGTTCTTTCCCGTTATAGCCAT

HAN1 TTAGCTGACTTCCAAGTGCCACACATCACCACTGTATTCATCCATGTTTTCACCGAACCAACGAGACAGATCGAAGAAGCCAGAATCTCTCGACTTTAAATTACATAAATCCAACGTATTATGACCACAGCTCGACACACAAATAGTTGCATTACTATTCACAGTAACATTACCTATACCCGTAACGTTGCACAACCACTGATCACCATTGTTACCAAAAACGGTTTTCCACTTAGTTGTCAACGGATCTTTCCTATGCGTAATGGTAAAATTACTACCAGTCGTCGCTTTTAGCTCATTACGAGTATTATCCGCATCCACATATATCAACGTCATAGCTAGGCACGCTATAAGTACCCCCCCCCC-----ACAATGGAATGTTGCCAAACCGGTTCTTCCCCGTTATAGCCAT

HAN30 TTAGCTGACTTCCAAGTGCCACACATCACCACTGTATTCATCCATGTTTTCACCGAACCAACGAGACAGATCGAAGAAGCCAGAATCTCCCGACTTTAAATTACATAAATCCAACGTATTATGACCACAGCTCGACACACAAATAGTTGCGTTACCATTCACAGTAGCATTACCTATACCCGTAACGTTGCACAACCACTGATCACCATTGTTACCAAAAACGGTTTTCCACTTAGTTGTCAACGGATCTTTCCTATGCGTAATGGTAAAATTACTCCCAGTCGTCGCTTTTAGCTCATTACGAGTATTATCCGCATCCACATATATCAACGTCATAGCTAGGCACGCTATAAGTACCCCCCCCCC-----ACAATGGAATGTTGCCAAACCGGTTCTTTCCCGTTATAGCCAT

Merlin TTAGCTGACTTCCAAGTGCCACACATCACCACTGTATTCATCCATGTTTTCACCGAACCAACGAGACAGATCGAAGAAGCCAGAATCTCCCGACTTTAAATTACATAAATCCAACGTATTATGACCACAGCTCGACACACAAATAGTTGCGTTACCATTCACAGTAGCATTACCTATACCCGTAACGTTGCACAACCACTGATCACCATTGTTACCAAAAACGGTTTTCCACTTAGTTGTCAACGGATCTTTCCTATGCGTAATGGTAAAATTACTCCCAGTCGTCGCTTTTAGCTCATTACGAGTATTATCCGCATCCACATATATCAACGTCATAGCTAGGCACGCTATAAGTACCCCCCCCCC-----ACAATGGAATGTTGCCAAACCGGTTCTTTCCCGTTATAGCCAT

CZ/1/2013 TTAGCTGACTTCCAAGTGCCACACATCACCACTGTATTCATCCATGTTTTCACCGAACCAACGAGACAGATCGAAGAAGCCAGAATCTCCCGACTTTAAATTACATAAATCCAACGTATTATGACCACAGCTCGACACACAAATAGTTGCGTTACCATTCACAGTAGCATTACCTATACCCGTAACGTTGCACAACCACTGATCACCATTGTTACCAAAAACGGTTTTCCACTTAGTTGTCAACGGATCTTTCCTATGCGTAATGGTAAAATTACTCCCAGTCGTCGCTTTTAGCTCATTACGAGTATTATCCGCATCCACATATATCAACGTCATAGCTAGGCACGCTATAAGTACCCCCCCCCC-----ACAATGGAATGTTGCCAAACCGGTTCTTTCCCGTTATAGCCAT

BE/23/2010 TTAGCTGACTTCCAAGTGCCACACATCACCACTGTATTCATCCATGTTTTCACCGAACCAACGAGACAGATCGAAGAAGCCAGAATCTCCCGACTTTAAATTACATAAATCCAACGTATTATGACCACAGCTCGACACACAAATAGTTGCGTTACCATTCACAGTAGCATTACCTATACCCGTAACGTTGCACAACCACTGATCACCATTGTTACCAAAAACGGTTTTCCACTTAGTTGTCAACGGATCTTTCCTATGCGTAATGGTAAAATTACTCCCAGTCGTCGCTTTTAGCTCATTACGAGTATTATCCGCATCCACATATATCAACGTCATAGCTAGGCACGCTATAAGTACCCCCCCCCC-----ACAATGGAATGTTGCCAAACCGGTTCTTTCCCGTTATAGCCAT

NL/Rot7 TTAGCTGACTTCCAAGTGCCACACATCACCACTGTATTCATCCATGTTTTCACCGAACCAACGAGACAGATCGAAGAAGCCAGAATCTCCCGACTTTAAATTACATAAATCCAACGTATTATGACCACAGCTCGACACACAAATAGTTGCGTTACCATTCACAGTAGCATTACCTATACCCGTAACGTTGCACAACCACTGATCACCATTGTTACCAAAAACGGTTTTCCACTTAGTTGTCAACGGATCTTTCCTATGCGTAATGGTAAAATTACTCCCAGTCGTCGCTTTTAGCTCATTACGAGTATTATCCGCATCCACATATATCAACGTCATAGCTAGGCACGCTATAAGTACCCCCCCCCC-----ACAATGGAATGTTGCCAAACCGGTTCTTTCCCGTTATAGCCAT

UK/Lon3 TTAGCTGACTTCCAAGTGCCACACATCACCACTGTATTCATCCATGTTTTCACCGAACCAACGAGACAGATCGAAGAAGCCAGAATCTCCCGACTTTAAATTACATAAATCCAACGTATTATGACCACAGCTCGACACACAAATAGTTGCGTTACCATTCACAGTAGCATTACCTATACCCGTAACGTTGCACAACCACTGATCACCATTGTTACCAAAAACGGTTTTCCACTTAGTTGTCAACGGATCTTTCCTATGCGTAATGGTAAAATTACTCCCAGTCGTCGCTTTTAGCTCATTACGAGTATTATCCGCATCCACATATATCAACGTCATAGCTAGGCACGCTATAAGTACCCCCCCCCC-----ACAATGGAATGTTGCCAAACCGGTTCTTTCCCGTTATAGCCAT

HANChild4 TTAGCTGACTTCCAAGTGCCACACATCACCACTGTATTCATCCATGTTTTCACCGAACCAACGAGACAGATCGAAGAAGCCAGAATCTCCCGACTTTAAATTACATAAATCCAACGTATTATGACCACAGCTCGACACACAAATAGTTGCGTTACCATTCACAGTAGCATTACCTATACCCGTAACGTTGCACAACCACTGATCACCATTGTTACCAAAAACGGTTTTCCACTTAGTTGTCAACGGATCTTTCCTATGCGTAATGGTAAAATTACTCCCAGTCGTCGCTTTTAGCTCATTACGAGTATTATCCGCATCCACATATATCAACGTCATAGCTAGGCACGCTATAAGTACCCCCCCCCC-----ACAATGGAATGTTGCCAAACCGGTTCTTTCCCGTTATAGCCAT

PRA4 TTAGCTGACTTCCAAGTGCCACACATCACCACTGTATTCATCCATGTTTTCACCGAACCAACGAGACAGATCGAAGAAGCCAGAATCTCCCGACTTTAAATTACATAAATCCAACGTATTATGACCACAGCTCGACACACAAATAGTTGCGTTACCATTCACAGTAGCATTACCTATACCCGTAACGTTGCACAACCACTGATCACCATTGTTACCAAAAACGGTTTTCCACTTAGTTGTCAACGGATCTTTCCTATGCGTAATGGTAAAATTACTCCCAGTCGTCGCTTTTAGCTCATTACGAGTATTATCCGCATCCACATATATCAACGTCATAGCTAGGCACGCTATAAGTACCCCCCCCCC-----ACAATGGAATGTTGCCAAACCGGTTCTTTCCCGTTATAGCCAT

HANRTR1B TTAGCTGACTTCCAAGTGCCACACATCACCACTGTATTCATCCATGTTTTCACCGAACCAACGAGACAGATCGAAGAAGCCAGAATCTCCCGACTTTAAATTACATAAATCCAACGTATTATGACCACAGCTCGACACACAAATAGTTGCGTTACCATTCACAGTAGCATTACCTATACCCGTAACGTTGCACAACCACTGATCACCATTGTTACCAAAAACGGTTTTCCACTTAGTTGTCAACGGATCTTTCCTATGCGTAATGGTAAAATTACTCCCAGTCGTCGCTTTTAGCTCATTACGAGTATTATCCGCATCCACATATATCAACGTCATAGCTAGGCACGCTATAAGTACCCCCCCCCC-----ACAATGGAATGTTGCCAAACCGGTTCTTTCCCGTTATAGCCAT

Toledo TTAGCTGACTTCCAAGTGCCACACATCACCACTGTATTCATCCATGTTTTCACCGAACCAACGAGACAGATCGAAGAAGCCAGAATCTCCCGACTTTAAATTACATAAATCCAACGTATTATGACCACAGCTCGACACACAAATAGTTGCGTTACTATTCACAGTAGCATTACCTATACCCGTAACGTTGCACAACCACTGATCACCATTGTTACCAAAAACGGTTTTCCACTTAGTTGTCAACGGATCTTTCCCATGCGTAATGGTCAAATTACTACCAGTCGTCGCTTTTAGCTCATTACGAGTATTATCCGCATCCACATATATCAACGTCATAGCTAGGCACGCTATAAGTACCCCCCCCCC-----ACAATGGAATGTTGCCAAACCGGTTCTTTCCCGTTATAGCCAT

CZ/3/2012 TTAGCTAACTTCCAAGTACCACACATCACCACTGTATTCATCCATGTTTTCACCGAACCAACGAGACAGATCGAAGAAGCCAGAATCTCCCGACTTTAAATTACATAAATCCAACGTATTATGACCACAGCTCGACACACAAATAGTTGCGTTACTATTCACAGTAGCATTACCTATACCCGTAACGTTGCACAACCACTGATCACCATTGTTACCAAAAACGGTTTTCCACTTAGTTGTCAACGGATCTTTCCTATGCGTAATGGTAAAATTACTACCAGTCGTCGCTTTTAGCTCATTACGAGTATTATCCGCATCCACATATATCAACGTCATAGCTAGGCACGCTATAAGTACCCCCCCCCC-----ACAATGGAATGTTGCCAAACCGGTTCTTTCCCGTTATAGCCAT

3301 TTAGCTAACTTCCAAGTACCACACATCACCACTGTATTCATCCATGTTTTCACCGAACCAACGAGACAGATCGAAGAAGCCAGAATCTCCCGACTTTAAATTACATAAATCCAACGTATTATGACCACAGCTCGACACACAAATAGTTGCGTTACTATTCACAGTAGCATTACCTATACCCGTAACGTTGCACAACCACTGATCACCATTGTTACCAAAAACGGTTTTCCACTTAGTTGTCAACGGATCTTTCCTATGCGTAATGGTAAAATTACTACCAGTCGTCGCTTTTAGCTCATTACGAGTATTATCCGCATCCACATATATCAACGTCATAGCTAGGCACGCTATAAGTACCCCCCCCCC-----ACAATGGAATGTTGCCAAACCGGTTCTTTCCCGTTATAGCCAT

BE/13/2012 TTAGCTAACTTCCAAGTACCACACATCACCACTGTATTCATCCATGTTTTCACCGAACCAACGAGACAGATCGAAGAAGCCAGAATCTCCCGACTTTAAATTACATAAATCCAACGTATTATGACCACAGCTCGACACACAAATAGTTGCGTTACTATTCACAGTAGCATTACCTATACCCGTAACGTTGCACAACCACTGATCACCATTGTTACCAAAAACGGTTTTCCACTTAGTTGTCAACGGATCTTTCCTATGCGTAATGGTAAAATTACTACCAGTCGTCGCTTTTAGCTCATTACGAGTATTATCCGCATCCACATATATCAACGTCATAGCTAGGCACGCTATAAGTACCCCCCCCCC-----ACAATGGAATGTTGCCAAACCGGTTCTTTCCCGTTATAGCCAT

PAV5 TTAGCTAACTTCCAAGTACCACACATCACCACTGTATTCATCCATGTTTTCACCGAACCAACGAGACAGATCGAAGAAGCCAGAATCTCCCGACTTTAAATTACATAAATCCAACGTATTATGACCACAGCTCGACACACAAATAGTTGCGTTACTATTCACAGTAGCATTACCTATACCCGTAACGTTGCACAACCACTGATCACCATTGTTACCAAAAACGGTTTTCCACTTAGTTGTCAACGGATCTTTCCTATGCGTAATGGTAAAATTACTACCAGTCGTCGCTTTTAGCTCATTACGAGTATTATCCGCATCCACATATATCAACGTCATAGCTAGGCACGCTATAAGTACCCCCCCCCC-----ACAATGGAATGTTGCCAAACCGGTTCTTTCCCGTTATAGCCAT

PRA8 TTAGCTAACTTCCAAGTACCACACATCACCACTGTATTCATCCATGTTTTCACCGAACCAACGAGACAGATCGAAGAAGCCAGAATCTCCCGACTTTAAATTACATAAATCCAACGTATTATGACCACAGCTCGACACACAAATAGTTGCGTTACTATTCACAGTAGCATTACCTATACCCGTAACGTTGCACAACCACTGATCACCATTGTTACCAAAAACGGTTTTCCACTTAGTTGTCAACGGATCTTTCCTATGCGTAATGGTAAAATTACTACCAGTCGTCGCTTTTAGCTCATTACGAGTATTATCCGCATCCACATATATCAACGTCATAGCTAGGCACGCTATAAGTACCCCCCCCCC-----ACAATGGAATGTTGCCAAACCGGTTCTTTCCCGTTATAGCCAT

HAN32 TTAGCTGACTTCCAAGTACCACACATCACCACTGTATTCATCCATGTTTTCACCGAACCAACGAGACAGATCGAAGAAGCCAGAATCTCCCGACTTTAAATTACATAAATCCAACGTATTATGACCACAGCTCGACACACAAATAGTTGCGTTACTATTCACAGTAGCATTACCTATACCCGTAACGTTGCACAACCACTGATCACCATTGTTACCAAAAACGGTTTTCCACTTAGTTGTCAACGGATCTTTCCTATGCGTAATGGTAAAATTACTACCAGTCGTCGCTTTTAGCTCATTACGAGTATTATCCGCATCCACATATATCAACGTCATAGCTAGGCACGCTATAAGTACCCCCCCCCC-----ACAATGGAATGTTGCCAAACCGGTTCTTTCCCGTTATAGCCAT

PAV8 TTAGCTGACTTCCAAGTACCACACATCACCACTGTATTCATCCATGTTTTCACCGAACCAACGAGACAGATCGAAGAAGCCAGAATCTCCCGACTTTAAATTACATAAATCCAACGTATTATGACCACAGCTCGACACACAAATAGTTGCGTTACTATTCACAGTAGCATTACCTATACCCGTAACGTTGCACAACCACTGATCACCATTGTTACCAAAAACGGTTTTCCACTTAGTTGTCAACGGATCTTTCCTATGCGTAATGGTAAAATTACTACCAGTCGTCGCTTTTAGCTCATTACGAGTATTATCCGCATCCACATATATCAACGTCATAGCTAGGCACGCTATAAGTACCCCCCCCCC-----ACAATGGAATGTTGCCAAACCGGTTCTTTCCCGTTATAGCCAT

BE/18/2010 TTAGCTGACTTCCAAGTACCACACATCACCACTGTATTCATCCATGTTTTCACCGAACCAACGAGACAGATCGAAGAAGCCAGAATCTCCCGACTTTAAATTACATAAATCCAACGTATTATGACCACAGCTCGACACACAAATAGTTGCGTTACTATTCACAGTAGCATTACCTATACCCGTAACGTTGCACAACCACTGATCACCATTGTTACCAAAAACGGTTTTCCACTTAGTTGTCAACGGATCTTTCCTATGCGTAATGGTAAAATTACTACCAGTCGTCGCTTTTAGCTCATTACGAGTATTATCCGCATCCACATATATCAACGTCATAGCTAGGCACGCTATAAGTACCCCCCCCCC-----ACAATGGAATGTTGCCAAACCGGTTCTTTCCCGTTATAGCCAT

BE/24/2010 TTAGCTGACTTCCAAGTACCACACATCACCACTGTATTCATCCATGTTTTCACCGAACCAACGAGACAGATCGAAGAAGCCAGAATCTCCCGACTTTAAATTACATAAATCCAACGTATTATGACCACAGCTCGACACACAAATAGTTGCGTTACTATTCACAGTAGCATTACCTATACCCGTAACGTTGCACAACCACTGATCACCATTGTTACCAAAAACGGTTTTCCACTTAGTTGTCAACGGATCTTTCCTATGCGTAATGGTAAAATTACTACCAGTCGTCGCTTTTAGCTCATTACGAGTATTATCCGCATCCACATATATCAACGTCATAGCTAGGCACGCTATAAGTACCCCCCCCCC-----ACAATGGAATGTTGCCAAACCGGTTCTTTCCCGTTATAGCCAT

2CEN30 TTAGCTGACTTCCAAGTACCACACATCACCACTGTATTCATCCATGTTTTCACCGAACCAACGAGACAGATCGAAGAAGCCAGAATCTCCCGACTTTAAATTACATAAATCCAACGTATTATGACCACAGCTCGACACACAAATAGTTGCGTTACTATTCACAGTAGCATTACCTATACCCGTAACGTTGCACAACCACTGATCACCATTGTTACCAAAAACGGTTTTCCACTTAGTTGTCAACGGATCTTTCCTATGCGTAATGGTAAAATTACTACCAGTCGTCGCTTTTAGCTCATTACGAGTATTATCCGCATCCACATATATCAACGTCATAGCTAGGCACGCTATAAGTACCCCCCCCCC-----ACAATGGAATGTTGCCAAACCGGTTCTTTCCCGTTATAGCCAT

UK/Lon4 TTAGCTGACTTCCAAGTACCACACATCACCACTGTATTCATCCATGTTTTCACCGAACCAACGAGACAGATCGAAGAAGCCAGAATCTCCCGACTTTAAATTACATAAATCCAACGTATTATGACCACAGCTCGACACACAAATAGTTGCGTTACTATTCACAGTAGCATTACCTATACCCGTAACGTTGCACAACCACTGATCACCATTGTTACCAAAAACGGTTTTCCACTTAGTTGTCAACGGATCTTTCCTATGCGTAATGGTAAAATTACTACCAGTCGTCGCTTTTAGCTCATTACGAGTATTATCCGCATCCACATATATCAACGTCATAGCTAGGCACGCTATAAGTACCCCCCCCCC-----ACAATGGAATGTTGCCAAACCGGTTCTTTCCCGTTATAGCCAT

PRA1 TTAGCTGACTTCCAAGTACCACACATCACCACTGTATTCATCCATGTTTTCACCGAACCAACGAGACAGATCGAAGAAGCCAGAATCTCCCGACTTTAAATTACATAAATCCAACGTATTATGACCACAGCTCGACACACAAATAGTTGCGTTACTATTCACAGTAGCATTACCTATACCCGTAACGTTGCACAACCACTGATCACCATTGTTACCAAAAACGGTTTTCCACTTAGTTGTCAACGGATCTTTCCTATGCGTAATGGTAAAATTACTACCAGTCGTCGCTTTTAGCTCATTACGAGTATTATCCGCATCCACATATATCAACGTCATAGCTAGGCACGCTATAAGTACCCCCCCCCC-----ACAATGGAATGTTGCCAAACCGGTTCTTTCCCGTTATAGCCAT

PRA2 TTAGCTGACTTCCAAGTACCACACATCACCACTGTATTCATCCATGTTTTCACCGAACCAACGAGACAGATCGAAGAAGCCAGAATCTCCCGACTTTAAATTACATAAATCCAACGTATTATGACCACAGCTCGACACACAAATAGTTGCGTTACTATTCACAGTAGCATTACCTATACCCGTAACGTTGCACAACCACTGATCACCATTGTTACCAAAAACGGTTTTCCACTTAGTTGTCAACGGATCTTTCCTATGCGTAATGGTAAAATTACTACCAGTCGTCGCTTTTAGCTCATTACGAGTATTATCCGCATCCACATATATCAACGTCATAGCTAGGCACGCTATAAGTACCCCCCCCCC-----ACAATGGAATGTTGCCAAACCGGTTCTTTCCCGTTATAGCCAT

PAV23 TTAGCTGACTTCCAAGTGCCACACATCACCACTGTATTCATCCATGTTTTCACCGAACCAACGAGACAGATCGAAGAAGCCAGAATCTCCCGACTTTAAATTACATAAATCCAACGTATTATGACCACAGCTCGACACACAAATAGTTGCGTTACTATTCACAGTAGCATTACCTATACCCGTAACGTTGCACAACCACTGATCACCATTGTTACCAAAAACGGTTTTCCACTTAGTTGTCAACGGATCTTTCCTATGCGTAATGGTAAAATTACTACCAGTCGTCGCTTTTAGCTCATTACGAGTATTATCCGCATCCACATATATCAACGTCATAGCTAGGCACGCTATAAGTACCCCCCCCCC-----ACAATGGAATGTTGCCAAACCGGTTCTTTCCCGTTATAGCCAT

DB TTAGCTGACTTCCAAGTGCCACACATCACCACTGTATTCATCCATGTTTTCACCGAACCAACGAGACAGATCGAAGAAGCCAGAATCTCCCGACTTTAAATTACATAAATCCAACGTATTATGACCACAGCTCGACACACAAATAGTTGCGTTACTATTCACAGTAGCATTACCTATACCCGTAACGTTGCACAACCACTGATCACCATTGTTACCAAAAACGGTTTTCCACTTAGTTGTCAACGGATCTTTCCTATGCGTAATGGTAAAATTACTACCAGTCGTCGCTTTTAGCTCATTACGAGTATTATCCGCATCCACATATATCAACGTCATAGCTAGGCACGCTATAAGTACCCCCCCCCC-----ACAATGGAATGTTGCCAAACCGGTTCTTTCCCGTTATAGCCAT

AD169 TTAGCTGACTTCCAAGTGCCACACATCACCACTGTATTCATCCATGTTTTCACCGAACCAACGAGACAGATCGAAGAAGCCAGAATCTCCCGACTTTAAATTACATAAATCCAACGTATTATGACCACAGCTCGACACACAAATAGTTGCGTTACTATTCACAGTAGCATTACCTATACCCGTAACGTTGCACAACCACTGATCACCATTGTTACCAAAAACGGTTTTCCACTTAGTTGTCAACGGATCTTTCCTATGCGTAATGGTAAAATTACTACCAGTCGTCGCTTTTAGCTCATTACGAGTATTATCCGCATCCACATATATCAACGTCATAGCTAGGCACGCTATAAGTACCCCCCCCCC-----ACAATGGAATGTTGCCAAACCGGTTCTTTCCCGTTATAGCCAT

BE/10/2010 TTAGCTGACTTCCAAGTGCCACACATCACCACTGTATTCATCCATGTTTTCACCGAACCAACGAGACAGATCGAAGAAGCCAGAATCTCCCGACTTTAAATTACATAAATCCAACGTATTATGACCACAGCTCGACACACAAATAGTTGCGTTACTATTCACAGTAGCATTACCTATACCCGTAACGTTGCACAACCACTGATCACCATTGTTACCAAAAACGGTTTTCCACTTAGTTGTCAACGGATCTTTCCTATGCGTAATGGTAAAATTACTACCAGTCGTCGCTTTTAGCTCATTACGAGTATTATCCGCATCCACATATATCAACGTCATAGCTAGGCACGCTATAAGTACCCCCCCCCC-----ACAATGGAATGTTGCCAAACCGGTTCTTTCCCGTTATAGCCAT

HAN8 TTAGCTGACTTCCAAGTGCCACACATCACCACTGTATTCATCCATGTTTTCACCGAACCAACGAGACAGATCGAAGAAGCCAGAATCTCCCGACTTTAAATTACATAAATCCAACGTATTATGACCACAGCTCGACACACAAATAGTTGCGTTACTATTCACAGTAGCATTACCTATACCCGTAACGTTGCACAACCACTGATCACCATTGTTACCAAAAACGGTTTTCCACTTAGTTGTCAACGGATCTTTCCTATGCGTAATGGTAAAATTACTACCAGTCGTCGCTTTTAGCTCATTACGAGTATTATCCGCATCCACATATATCAACGTCATAGCTAGGCACGCTATAAGTACCCCCCCCCC-----ACAATGGAATGTTGCCAAACCGGTTCTTTCCCGTTATAGCCAT

JER4035 TTAGCTGACTTCCAAGTGCCACACATCACCACTGTATTCATCCATGTTTTCACCGAACCAACGAGACAGATCGAAGAAGCCAGAATCTCCCGACTTTAAATTACATAAATCCAACGTATTATGACCACAGCTCGACACACAAATAGTTGCGTTACTATTCACAGTAGCATTACCTATACCCGTAACGTTGCACAACCACTGATCACCATTGTTACCAAAAACGGTTTTCCACTTAGTTGTCAACGGATCTTTCCTATGCGTAATGGTAAAATTACTACCAGTCGTCGCTTTTAGCTCATTACGAGTATTATCCGCATCCACATATATCAACGTCATAGCTAGGCACGCTATAAGTACCCCCCCCCC-----ACAATGGAATGTTGCCAAACCGGTTCTTTCCCGTTATAGCCAT

BE/8/2012 TTAGCTGACTTCCAAGTGCCACACATCACCACTGTATTCATCCATGTTTTCACCGAACCAACGAGACAGATCGAAGAAGCCAGAATCTCCCGACTTTAAATTACATAAATCCAACGTATTATGACCACAGCTCGACACACAAATAGTTGCGTTACTATTCACAGTAGCATTACCTATACCCGTAACGTTGCACAACCACTGATCACCATTGTTACCAAAAACGGTTTTCCACTTAGTTGTCAACGGATCTTTCCTATGCGTAATGGTAAAATTACTACCAGTCGTCGCTTTTAGCTCATTACGAGTATTATCCGCATCCACATATATCAACGTCATAGCTAGGCACGCTATAAGTACCCCCCCCCC-----ACAATGGAATGTTGCCAAACCGGTTCTTTCCCGTTATAGCCAT

CZ/2/2012 TTAGCTGACTTCCAAGTGCCACACATCACCACTGTATTCATCCATGTTTTCACCGAACCAACGAGACAGATCGAAGAAGCCAGAATCTCCCGACTTTAAATTACATAAATCCAACGTATTATGACCACAGCTCGACACACAAATAGTTGCGTTACTATTCACAGTAGCATTACCTATACCCGTAACGTTGCACAACCACTGATCACCATTGTTACCAAAAACGGTTTTCCACTTAGTTGTCAACGGATCTTTCCTATGCGTAATGGTAAAATTACTACCAGTCGTCGCTTTTAGCTCATTACGAGTATTATCCGCATCCACATATATCAACGTCATAGCTAGGCACGCTATAAGTACCCCCCCCCC-----ACAATGGAATGTTGCCAAACCGGTTCTTTCCCGTTATAGCCAT

VR1814 TTAGCTGACTTCCAAGTGCCACACATCACCACTGTATTCATCCATGTTTTCACCGAACCAACGAGACAGATCGAAGAAGCCAGAATCTCCCGACTTTAAATTACATAAATCCAACGTATTATGACCACAGCTCGACACACAAATAGTTGCGTTACTATTCACAGTAGCATTACCTATACCCGTAACGTTGCACAACCACTGATCACCATTGTTACCAAAAACGGTTTTCCACTTAGTTGTCAACGGATCTTTCCTATGCGTAATGGTAAAATTACTACCAGTCGTCGCTTTTAGCTCATTACGAGTATTATCCGCATCCACATATATCAACGTCATAGCTAGGCACGCTATAAGTACCCCCCCCCC-----ACAATGGAATGTTGCCAAACCGGTTCTTTCCCGTTATAGCCAT

HANSCTR9 TTAGCTGACTTCCAAGTGCCACACATCACCACTGTATTCATCCATGTTTTCACCGAACCAACGAGACAGATCGAAGAAGCCAGAATCTCCCGACTTTAAATTACATAAATCCAACGTATTATGACCACAGCTCGACACACAAATAGTTGCGTTACTATTCACAGTAGCATTACCTATACCCGTAACGTTGCACAACCACTGATCACCATTGTTACCAAAAACGGTTTTCCACTTAGTTGTCAACGGATCTTTCCTATGCGTAATGGTAAAATTACTACCAGTCGTCGCTTTTAGCTCATTACGAGTATTATCCGCATCCACATATATCAACGTCATAGCTAGGCACGCTATAAGTACCCCCCCCCC-----ACAATGGAATGTTGCCAAACCGGTTCTTTCCCGTTATAGCCAT

BE/49/2011 TTAGCTGACTTCCAAGTGCCACACATCACCACTGTATTCATCCATGTTTTCACCGAACCAACGAGACAGATCGAAGAAGCCAGAATCTCCCGACTTTAAATTACATAAATCCAACGTATTATGACCACAGCTCGACACACAAATAGTTGCGTTACTATTCACAGTAGCATTACCTATACCCGTAACGTTGCACAACCACTGATCACCATTGTTACCAAAAACGGTTTTCCACTTAGTTGTCAACGGATCTTTCCTATGCGTAATGGTAAAATTACTACCAGTCGTCGCTTTTAGCTCATTACGAGTATTATCCGCATCCACATATATCAACGTCATAGCTAGGCACGCTATAAGTACCCCCCCCCC-----ACAATGGAATGTTGCCAAACCGGTTATTTCCCGTTATAGCCAT

HAN34 TTAGCTGACTTCCAAGTGCCACACATCACCACTGTATTCATCCATGTTTTCACCGAACCAACGAGACAGATCGAAGAAGCCAGAATCTCCCGACTTTAAATTACATAAATCCAACGTATTATGACCACAGCTCGACACACAAATAGTTGCGTTACTATTCACAGTAGCATTACCTATACCCGTAACGTTGCACAACCACTGATCACCATTGTTACCAAAAACGGTTTTCCACTTAGTTGTCAACGGATCTTTCCTATGCGTAATGGTAAAATTACTACCAGTCGTCGCTTTTAGCTCATTACGAGTATTATCCGCATCCACATATATCAACGTCATAGCTAAGCACGCTATAAGTACCCCCCCCCCCC---ACAATGGAATGTTGCCAAACCGGTTCTTTCCCGTTATAGCCAT

Pat_G TTAGCTGACTTCCAAGTACCACACATCACCACTGTATTCATCCATGTTTTCACCGAACCAACGAGACAGATCGAAGAAGCCAGAATCTCCCGACTTTAAATTACATAAATCCAACGTATTATGACCACAGCTCGACACACAAATAGTTGCGTTACTATTCACAGTAGCATTACCTATACCCGTAACGTTGCACAACCACTGATCACCATTGTTACCAAAAACGGTTTTCCACTTAGTTGTCAACGGATCTTTCCTATGCGTAATGGTAAAATTACTACCAGTCGTCGCTTTTAGCTCATTACGAGTATTATCCGCATCCACATATATCAACGTCATAGCTAGGCACGCTATAAGTACCCCCCCCCCCC---ACAATGGAATGTTGCCAAACCGGTTCTTTCCCGTTATAGCCAT

PAV11 TTAGCTGACTTCCAAGTGCCACACATCACCACTGTATTCATCCATGTTTTCACCGAACCAACGAGACAGATCGAAGAAGCCAGAATCTCCCGACTTTAAATTACATAAATCCAACGTATTATGACCACAGCTCGACACACAAATAGTTGCGTTACCATTCACAGTAGCATTACCTATACCCGTAACGTTGCACAACCACTGATCACCATTGTTACCAAAAACGGTTTTCCACTTAGTTGTCAACGGATCTTTCCTATGCGTAATGGTAAAATTACTCCCAGTCGTCGCTTTTAGCTCATTACGAGTATTATCCGCATCCACATATATCAACGTCATAGCTAGGCACGCTATAAGTACCCCCCCCCCCC---ACAATGGAATGTTGCCAAACCGGTTCTTTCCCGTTATAGCCAT

Pat_K TTAGCTGACTTCCAAGTACCACACATCACCACTGTATTCATCCATGTTTTCACCGAACCAACGAGACAGATCGAAGAAGCCAGAATCTCCCGACTTTAAATTACATAAATCCAACGTATTATGACCACAGCTCGACACACAAATAGTTGCGTTACTATTCACAGTAGCATTACCTATACCCGTAACGTTGCACAACCACTGATCACCATTGTTACCAAAAACGGTTTTCCACTTAGTTGTCAACGGATCTTTCCTATGCGTAATGGTAAGATTACTACCAGTCGTCGCTTTTAGCTCATTACGAGTATTATCCGCATCCACATATATCAACGTCATAGCTAGGCACGCTATAAGTACCCCCCCCC------ACAATGGAATGTTGCCAAACCGGTTCTTTCCCGTTATAGCCAT

JP TTAGCTGACTTCCAAGTGCCACACATCACCACTGTATTCATCCATGTTTTCACCGAACCAACGAGACAGATCGAAGAAGCCAGAATCTCCCGACTTTAAATTACATAAATCCAACGTATTATGACCACAGCTCGACACACAAATAGTTGCGTTACTATTCACAGTAGCATTACCTATACCCGTAACGTTGCACAACCACTGATCACCATTGTTACCAAAAACGGTTTTCCACTTAGTTGTCAACGGATCTTTCCTATGCGTAATGGTAAAATTACTACCAGTCGTCGCTTTTAGCTCATTACGAGTATTATCCGCATCCACATATATCAACGTCATAGCTAGGCACGCTATAAGTACCCCCCCCC------ACAATGGAATGTTGCCAAACCGGTTCTTTCCCGTTATAGCCAT

BE/2/2010 TTAGCTGACTTCCAAGTGCCACACATCACCACTGTATTCATCCATGTTTTCACCGAACCAACGAGACAGATCGAAGAAGCCAGAATCTCCCGACTTTAAATTACATAAATCCAACGTATTATGACCACAGCTCGACACACAAATAGTTGCGTTACTATTCACAGTAGCATTACCTATACCCGTAACGTTGCACAACCACTGATCACCATTGTTACCAAAAACGGTTTTCCACTTAGTTGTCAACGGATCTTTCCTATGCGTAATGGTAAAATTACTACCAGTCGTCGCTTTTAGCTCATTACGAGTATTATCCGCATCCACATATATCAACGTCATAGCTAGGCACGCTATAAGTACCCCCCCCC------ACAATGGAATGTTGCCAAACCGGTTCTTTCCCGTTATAGCCAT

PAV12 TTAGCTGACTTCCAAGTGCCACACATCACCACTGTATTCATCCATGTTTTCACCGAACCAACGAGACAGATCGAAGAAGCCAGAATCTCCCGACTTTAAATTACATAAATCCAACGTATTATGACCACAGCTCGACACACAAATAGTTGCGTTACTATTCACAGTAGCATTACCTATACCCGTAACGTTGCACAACCACTGATCACCATTGTTACCAAAAACGGTTTTCCACTTAGTTGTCAACGGATCTTTCCTATGCGTAATGGTAAAATTACTACCAGTCGTCGCTTTTAGCTCATTACGAGTATTATCCGCATCCACATATATCAACGTCATAGCTAGGCACGCTATAAGTACCCCCCCCC------ACAATGGAATGTTGCCAAACCGGTTCTTTCCCGTTATAGCCAT

JER5409 TTAGCTGACTTCCAAGTGCCACACATCACCACTGTATTCATCCATGTTTTCACCGAACCAACGAGACAGATCGAAGAAGCCAGAATCTCCCGACTTTAAATTACATAAATCCAACGTATTATGACCACAGCTCGACACACAAATAGTTGCGTTACTATTCACAGTAGCATTACCTATACCCGTAACGTTGCACAACCACTGATCACCATTGTTACCAAAAACGGTTTTCCACTTAGTTGTCAACGGATCTTTCCTATGCGTAATGGTAAAATTACTACCAGTCGTCGCTTTTAGCTCATTACGAGTATTATCCGCATCCACATATATCAACGTCATAGCTAGGCACGCTATAAGTACCCCCCCCC------ACAATGGAATGTTGCCAAACCGGTTCTTTCCCGTTATAGCCAT

JER4041 TTAGCTGACTTCCAAGTGCCACACATCACCACTGTATTCATCCATGTTTTCACCGAACCAACGAGACAGATCGAAGAAGCCAGAATCTCCCGACTTTAAATTACATAAATCCAACGTATTATGACCACAGCTCGACACACAAATAGTTGCGTTACTATTCACAGTAGCATTACCTATACCCGTAACGTTGCACAACCACTGATCACCATTGTTACCAAAAACGGTTTTCCACTTAGTTGTCAACGGATCTTTCCTATGCGTAATGGTAAAATTACTACCAGTCGTCGCTTTTAGCTCATTACGAGTATTATCCGCATCCACATATATCAACGTCATAGCTAGGCACGCTATAAGTACCCCCCCCC------ACAATGGAATGTTGCCAAACCGGTTCTTTCCCGTTATAGCCAT

JER851 TTAGCTGACTTCCAAGTGCCACACATCACCACTGTATTCATCCATGTTTTCACCGAACCAACGAGACAGATCGAAGAAGCCAGAATCTCCCGACTTTAAATTACATAAATCCAACGTATTATGACCACAGCTCGACACACAAATAGTTGCGTTACTATTCACAGTAGCATTACCTATACCCGTAACGTTGCACAACCACTGATCACCATTGTTACCAAAAACGGTTTTCCACTTAGTTGTCAACGGATCTTTCCTATGCGTAATGGTAAAATTACTACCAGTCGTCGCTTTTAGCTCATTACGAGTATTATCCGCATCCACATATATCAACGTCATAGCTAGGCACGCTATAAGTACCCCCCCCC------ACAATGGAATGTTGCCAAACCGGTTCTTTCCCGTTATAGCCAT

UK/Lon8 TTAGCTGACTTCCAAGTGCCACACATCACCACTGTATTCATCCATGTTTTCACCGAACCAACGAGACAGATCGAAGAAGCCAGAATCTCCCGACTTTAAATTACATAAATCCAACGTATTATGACCACAGCTCGACACACAAATAGTTGCGTTACTATTCACAGTAGCATTACCTATACCCGTAACGTTGCACAACCACTGATCACCATTGTTACCAAAAACGGTTTTCCACTTAGTTGTCAACGGATCTTTCCTATGCGTAATGGTAAAATTACTACCAGTCGTCGCTTTTAGCTCATTACGAGTATTATCCGCATCCACATATATCAACGTCATAGCTAGGCACGCTATAAGTACCCCCCCCC------ACAATGGAATGTTGCCAAACCGGTTCTTTCCCGTTATAGCCAT

HANRTR5 TTAGCTGACTTCCAAGTGCCACACATCACCACTGTATTCATCCATGTTTTCACCGAACCAACGAGACAGATCGAAGAAGCCAGAATCTCCCGACTTTAAATTACATAAATCCAACGTATTATGACCACAGCTCGACACACAAATAGTTGCGTTACTATTCACAGTAGCATTACCTATACCCGTAACGTTGCACAACCACTGATCACCATTGTTACCAAAAACGGTTTTCCACTTAGTTGTCAACGGATCTTTCCTATGCGTAATGGTAAAATTACTACCAGTCGTCGCTTTTAGCTCATTACGAGTATTATCCGCATCCACATATATCAACGTCATAGCTAGGCACGCTATAAGTACCCCCCCCC------ACAATGGAATGTTGCCAAACCGGTTCTTTCCCGTTATAGCCAT

PRA7 TTAGCTGACTTCCAAGTGCCACACATCACCACTGTATTCATCCATGTTTTCACCGAACCAACGAGACAGATCGAAGAAGCCAGAATCTCCCGACTTTAAATTACATAAATCCAACGTATTATGACCACAGCTCGACACACAAATAGTTGCGTTACTATTCACAGTAGCATTACCTATACCCGTAACGTTGCACAACCACTGATCACCATTGTTACCAAAAACGGTTTTCCACTTAGTTGTCAACGGATCTTTCCTATGCGTAATGGTAAAATTACTACCAGTCGTCGCTTTTAGCTCATTACGAGTATTATCCGCATCCACATATATCAACGTCATAGCTAGGCACGCTATAAGTACCCCCCCCC------ACAATGGAATGTTGCCAAACCGGTTCTTTCCCGTTATAGCCAT

HAN19 TTAGCTGACTTCCAAGTGCCACACATCACCACTGTATTCATCCATGTTTTCACCGAACCAACGAGACAGATCGAAGAAGCCAGAATCTCCCGACTTTAAATTACATAAATCCAACGTATTATGACCACAGCTCGACACACAAATAGTTGCGTTACTATTCACAGTAGCATTACCTATACCCGTAACGTTGCACAACCACTGATCACCATTGTTACCAAAAACGGTTTTCCACTTAGTTGTCAACGGATCTTTCCTATGCGTAATGGTAAAATTACTACCAGTCGTCACTTTTAGCTCATTACGAGTATTATCCGCATCCACATATATCAACGTCATAGCTAGGCACGCTATAAGTACCCCCCCCC------ACAATGGAATGTTGCCAAACCGGTTCTTTCCCGTTATAGCCAT

Pat_C TTAGCTGACTTCCAAGTGCCACACATCACCACTGTATTCATCCATGTTTTCACCGAACCAACGAGACAGATCGAAGAAGCCAGAATCTCCCGACTTTAAATTACATAAATCCAACGTATTATGACCACAGCTCGACACACAAATAGTTGCGTTACTATTCACAGTAGCATTACCTATACCCGTAACGTTGCACAACCACTGATCACCATTGTTACCAAAAACGGTTTTCCACTTAGTTGTCAACGGATCTTTCCTATGCGTAATGGTAAAATTACTACCAGTCGTCGCTTTTAGCTCATTACGAGTATTATCCGCATCCACATATATCAACGTCATAGCTAGGCACGCTATAAGTACCCCCCCCCCCAC--ACAATGGAATGTTGCCAAACCGGTTCTTTCCCGTTATAGCCAT

BE/4/2010 TTAGCTGACTTCCAAGTGCCACACATCACCACTGTATTCATCCATGTTTTCACCGAACCAACGAGACAGATCGAAGAAGCCAGAATCTCCCGACTTTAAATTACATAAATCCAACGTATTATGACCACAGCTCGACACACAAATAGTTGCGTTACTATTCACAGTAGCATTACCTATACCCGTAACGTTGCACAACCACTGATCACCATTGTTACCAAAAACGGTTTTCCACTTAGTTGTCAACGGATCTTTCCTATGCGTAATGGTAAAATTACTACCAGTCGTCGCTTTTAGCTCATTACGAGTATTATCCGCATCCACATATATCAACGTCATAGCTAGGCACGCTATAAGTACCCCCCCCCCC----ACAATGGAATGTTGCCAAACCGGTTCTTTCCCGTTATAGCCAT

PAV16 TTAGCTGACTTCCAAGTGCCACACATCACCACTGTATTCATCCATGTTTTCACCGAACCAACGAGACAGATCGAAGAAGCCAGAATCTCCCGACTTTAAATTACATAAATCCAACGTATTATGACCACAGCTCGACACACAAATAGTTGCGTTACTATTCACAGTAGCATTACCTATACCCGTAACGTTGCACAACCACTGATCACCATTGTTACCAAAAACGGTTTTCCACTTAGTTGTCAACGGATCTTTCCTATGCGTAATGGTAAAATTACTACCAGTCGTCGCTTTTAGCTCATTACGAGTATTATCCGCATCCACATATATCAACGTCATAGCTAGGCACGCTATAAGTACCCCCCCCCCC----ACAATGGAATGTTGCCAAACCGGTTCTTTCCCGTTATAGCCAT

BE/44/2011 TTAGCTGACTTCCAAGTGCCACACATCACCACTGTATTCATCCATGTTTTCACCGAACCAACGAGACAGATCGAAGAAGCCAGAATCTCCCGACTTTAAATTACATAAATCCAACGTATTATGACCACAGCTCGACACACAAATAGTTGCGTTACTATTCACAGTAGCATTACCTATACCCGTAACGTTGCACAACCACTGATCACCATTGTTACCAAAAACGGTTTTCCACTTAGTTGTCAACGGATCTTTCCTATGCGTAATGGTAAAATTACTACCAGTCGTCGCTTTTAGCTCATTACGAGTATTATCCGCATCCACATATATCAACGTCATAGCTAGGCACGCTATAAGTACCCCCCCCCCC----ACAATGGAATGTTGCCAAACCGGTTCTTTCCCGTTATAGCCAT

BE/15/2010 TTAGCTGACTTCCAAGTGCCACACATCACCACTGTATTCATCCATGTTTTCACCGAACCAACGAGACAGATCGAAGAAGCCAGAATCTCCCGACTTTAAATTACATAAATCCAACGTATTATGACCACAGCTCGACACACAAATAGTTGCGTTACTATTCACAGTAGCATTACCTATACCCGTAACGTTGCACAACCACTGATCACCATTGTTACCAAAAACGGTTTTCCACTTAGTTGTCAACGGATCTTTCCTATGCGTAATGGTAAAATTACTACCAGTCGTCGCTTTTAGCTCATTACGAGTATTATCCGCATCCACATATATCAACGTCATAGCTAGGCACGCTATAAGTACCCCCCCCCCC----ACAATGGAATGTTGCCAAACCGGTTCTTTCCCGTTATAGCCAT

BE/5/2011 TTAGCTGACTTCCAAGTGCCACACATCACCACTGTATTCATCCATGTTTTCACCGAACCAACGAGACAGATCGAAGAAGCCAGAATCTCCCGACTTTAAATTACATAAATCCAACGTATTATGACCACAGCTCGACACACAAATAGTTGCGTTACTATTCACAGTAGCATTACCTATACCCGTAACGTTGCACAACCACTGATCACCATTGTTACCAAAAACGGTTTTCCACTTAGTTGTCAACGGATCTTTCCTATGCGTAATGGTAAAATTACTACCAGTCGTCGCTTTTAGCTCATTACGAGTATTATCCGCATCCACATATATCAACGTCATAGCTAGGCACGCTATAAGTACCCCCCCCCCC----ACAATGGAATGTTGCCAAACCGGTTCTTTCCCGTTATAGCCAT

BE/25/2010 TTAGCTGACTTCCAAGTGCCACACATCACCACTGTATTCATCCATGTTTTCACCGAACCAACGAGACAGATCGAAGAAGCCAGAATCTCCCGACTTTAAATTACATAAATCCAACGTATTATGACCACAGCTCGACACACAAATAGTTGCGTTACTATTCACAGTAGCATTACCTATACCCGTAACGTTGCACAACCACTGATCACCATTGTTACCAAAAACGGTTTTCCACTTAGTTGTCAACGGATCTTTCCTATGCGTAATGGTAAAATTACTACCAGTCGTCGCTTTTAGCTCATTACGAGTATTATCCGCATCCACATATATCAACGTCATAGCTAGGCACGCTATAAGTACCCCCCCCCCC----ACAATGGAATGTTGCCAAACCGGTTCTTTCCCGTTATAGCCAT

NL/Rot3 TTAGCTGACTTCCAAGTGCCACACATCACCACTGTATTCATCCATGTTTTCACCGAACCAACGAGACAGATCGAAGAAGCCAGAATCTCCCGACTTTAAATTACATAAATCCAACGTATTATGACCACAGCTCGACACACAAATAGTTGCGTTACTATTCACAGTAGCATTACCTATACCCGTAACGTTGCACAACCACTGATCACCATTGTTACCAAAAACGGTTTTCCACTTAGTTGTCAACGGATCTTTCCTATGCGTAATGGTAAAATTACTACCAGTCGTCGCTTTTAGCTCATTACGAGTATTATCCGCATCCACATATATCAACGTCATAGCTAGGCACGCTATAAGTACCCCCCCCCCC----ACAATGGAATGTTGCCAAACCGGTTCTTTCCCGTTATAGCCAT

JER5695 TTAGCTGACTTCCAAGTGCCACACATCACCACTGTATTCATCCATGTTTTCACCGAACCAACGAGACAGATCGAAGAAGCCAGAATCTCCCGACTTTAAATTACATAAATCCAACGTATTATGACCACAGCTCGACACACAAATAGTTGCGTTACTATTCACAGTAGCATTACCTATACCCGTAACGTTGCACAACCACTGATCACCATTGTTACCAAAAACGGTTTTCCACTTAGTTGTCAACGGATCTTTCCTATGCGTAATGGTAAAATTACTACCAGTCGTCGCTTTTAGCTCATTACGAGTATTATCCGCATCCACATATATCAACGTCATAGCTAGACACGCTATAAGTACCCCCCCCCCC----ACAATGGAATGTTGCCAAACCGGTTCTTTCCCGTTATAGCCAT

BE/45/2011 TTACTTGACTTCCAAGTGCCACACATCACCACTATATTCATCCATGTTTTCACCGAACCAACGAGACAGATCGAAAAAGCCAGAATCTTCCGACTTTAAATTACATAAATCCAACGTATTATGACCACAGCTCGACACACAAATAGTTGCGTTACTATTCACAGTAGCATTACCTATACCCGTAACGTTGCACAACCACTGATCACCATTGTTACCAAAAACGGTTTTCCACTTAGTTGTCAATGGATCTTTCCTATGCGTAATGGTAAAATTACTACCAGGCGTCGCTTTTAGCTCATTACGAGTATTATCCGCATCCACATATATCAACGTCATAGCTAGGTACGCTATAAGTACCCCCCCCCC-----ACAATGGAATGTTGCCAAACCGGTTCTTTCCCGTTATAGCCAT

HAN16 TTACTTGACTTCCAAGTGCCACACATCACCACTATATTCATCCATGTTTTCACCGAACCAACGAGACAGATCGAAGAAGCCAGAATCTTCCGACTTTAAATTACATAAATTCAACGTATTATGACCACAGTTCGACACACAAATAGTTGCGTTACTATTCACAGTAGCATTACCTATACCCGTAACGTTGCACAACCACTGATCACCATTGTTACCAAAAACGGTTCTCCACTTGGTTGTCAATGGATCTTTCCTATGCGTAATGGTAAAATTACTACCAGTCGTCGCTTTTAGCTCATTACGAGTATTATCCGCATCCACATATATCAACGTCATAGCTAGGTACGCTATAAGTACCCCCCCCCC-----ACAATGGAATGTTGCCAAACCGGTTCTTTCCCGTTATAGCCAT

BE/28/2011 TTACTTGACTTCCAAGTGCCACACATCACCACTATATTCATCCATGTTTTCACCGAACCAACGAGACAGATCGAAGAAGCCAGAATCTTCCGACTTTAAATTACATAAATTCAACGTATTATGACCACAGTTCGACACACAAATAGTTGCGTTACTATTCACAGTAGCATTACCTATACCCGTAACGTTGCACAACCACTGATCACCATTGTTACCAAAAACGGTTCTCCACTTGGTTGTCAATGGATCTTTCCTATGCGTAATGGTAAAATTACTACCAGTCGTCGCTTTTAGCTCATTACGAGTATTATCCGCATCCACATATATCAACGTCATAGCTAGGTACGCTATAAGTACCCCCCCCCC-----ACAATGGAATGTTGCCAAACCGGTTCTTTCCCGTTATAGCCAT

HANSCTR11A TTACTTGACTTCCAAGTGCCACACATCACCACTATATTCATCCATGTTTTCACCGAACCAACGAGACAGATCGAAGAAGCCAGAATCTTCCGACTTTAAATTACATAAATTCAACGTATTATGACCACAGTTCGACACACAAATAGTTGCGTTACTATTCACAGTAGCATTACCTATACCCGTAACGTTGCACAACCACTGATCACCATTGTTACCAAAAACGGTTCTCCACTTGGTTGTCAATGGATCTTTCCTATGCGTAATGGTAAAATTACTACCAGTCGTCGCTTTTAGCTCATTACGAGTATTATCCGCATCCACATATATCAACGTCATAGCTAGGTACGCTATAAGTACCCCCCCCC------ACAATGGAATGTTGCCAAACCGGTTCTTTCCCGTTATAGCCAT

BE/48/2011 TTATTTGACTTCCAAGTGCCACACATCACCACTATATTCATCCATGTTTTCACCGAACCAACGAGACAGATCGAAGAAGCCAGAATCTTCCGACTTTAAATTACATAAATCCAACGTATTATGACCACAGCTCGACACACAAATAGTTGCGTTACTATTCACAGTAGCATTACCTATACCCGTAACGTTGCACAACCACTGATCACCATTGTCACCAAAAACGGTTTTCCACTTAGTTGTCAACGGATCTTTCCTATGTGTAATGGTAAAATTACTACCAGTCGTCGCTTTTAGCTCATTACGAGTATTATCCGCATCCACATATATCAACGTCATAGCTAGGCACGCTATAAGTACCCCCCCC-------ACAATGGAATGTTGCCAAACCGGTTCTTTCCCGTTATAGCCAT

BE/33/2011 TTATTTGACTTCCAAGTGCCACACATCACCACTATATTCATCCATGTTTTCACCGAACCAACGAGACAGATCGAAGAAGCCAGAATCTTCCGACTTTAAATTACATAAATCCAACGTATTATGACCACAGCTCGACACACAAATAGTTGCGTTACTATTCACAGTGGCATTACCTATACCCGTAACGTTGCACAACCACTGATCACCATTGTCACCAAAAACGGTTTTCCACTTAGTTGTCAACGGATCTTTCCTATGCGTAATGGTAAAATTACTACCAGTCGTCGCTTTTAGCTCATTACGAGTATTATCCGCATCCACATATATCAACGTCATAGCTAGGCACGCTATAAGTACCCCCCCT-------ACAATGGAATGTTGCCAAACCGGTTCTTTCCCGTTATAGCCAT

NAN2LA TTATTTGACTTCCAAGTGCCACACATCACCACTATATTCATCCATGTTTTCACCGAACCAACGAGACAGATCGAAGAAGCCAGAATCTTCCGACTTTAAATTACATAAATCCAACGTATTATGACCACAGCTCGACACACAAATAGTTGCGTTACTATTCACAGTGGCATTACCTATACCCGTAACGTTGCACAACCACTGATCACCATTGTCACCAAAAACGGTTTTCCACTTAGTTGTCAACGGATCTTTCCTATGCGTAATGGTAAAATTACTACCAGTCGTCGCTTTTAGCTCATTACGAGTATTATCCGCATCCACATATATCAACGTCATAGCTAGGCACGCTATAAGTACCCCCCCT-------ACAATGGAATGTTGCCAAACCGGTTCTTTCCCGTTATAGCCAT

BE/19/2010 TTATTTGACTTCCAAGTGCCACACATCACCACTATATTCATCCATGTTTTCACCGAACCAACGAGACAAATCGAAGAAGCCAGAATCTTCCGACTTTAAATTACATAAATCCAACGTATTATGACCACAGCTCGACACACAAATAGTTGCGTTACTATTCACAGTGGCATTACCTATACCCGTAACGTTGCACAACCACTGATCACCATTGTCACCAAAAACGGTTTTCCACTTAGTTGTCAACGGATCTTTCCTATGCGTAATGGTAAAATTACTACCAGTCGTCGCTTTTAGCTCATTACGAGTATTATCCGCATCCACATATATCAACGTCATAGCTAGGCACGCTATAAGTACCCCCCCT-------ACAATGGAATGTTGCCAAACCGGTTCTTTCCCGTTATAGCCAT

BE/18/2011 TTATTTGACTTCCAAGTGCCACACATCACCACTATATTCATCCATGTTTTCACCGAACCAACGAGACAGATCGAAGAAGCCAGAATCTTCCGACTTTAAATTACATAAATCCAACGTATTATGACCACAGCTCGACACACAAATAGTTGCGTTACTATTCACAGTGGCATTACCTATACCCGTAACGTTGCACAACCACTGATCACCATTGTCACCAAAAACGGTTTTCCACTTAGTTGTCAACGGATCTTTCCTATGCGTAATGGTAAAATTACTACCAGTCGTCGCTTTTAGCTCATTACGAGTATTATCCGCATCCACATATATCAACGTCATAGCTAGGCACGCTATAAGTACCCCCCCCCC-----ACAATGGAATGTTGCCAAACCGGTTCTTTCCCGTTATAGCCAT

BE/27/2011 TTATTTGACTTCCAAGTGCCACACATCACCACTATATTCATCCATGTTTTCACCGAACCAACGAGACAGATCGAAGAAGCCAGAATCTTCCGACTTTAAATTACATAAATCCAACGTATTATGACCACAGCTCGACACACAAATAGTTGCGTTACTATTCACAGTGGCATTACCTATACCCGTAACGTTGCACAACCACTGATCACCATTGTCACCAAAAACGGTTTTCCACTTAGTTGTCAACGGATCTTTCCTATGCGTAATGGTAAAATTACTACCAGTCGTCGCTTTTAGCTCATTACGAGTATTATCCGCATCCACATATATCAACGTCATAGCTAGGCACGCTATAAGTACCCCCCCCCC-----ACAATGGAATGTTGCCAAACCGGTTCTTTCCCGTTATAGCCAT

BE/6/2011 TTATTTGACTTCCAAGTGCCACACATCACCACTATATTCATCCATGTTTTCACCGAACCAACGAGACAGATCGAAGAAGCCAGAATCTTCCGACTTTAAATTACATAAATCCAACGTATTATGACCACAGCTCGACACACAAATAGTTGCGTTACTATTCACAGTGGCATTACCTATACCCGTAACGTTGCACAACCACTGATCACCATTGTCACCAAAAACGGTTTTCCACTTAGTTGTCAACGGATCTTTCCTATGCGTAATGGTAAAATTACTACCAGTCGTCGCTTTTAGCTCATTACGAGTATTATCCGCATCCACATATATCAACGTCATAGCTAGGCACGCTATAAGTACCCCCCCCCC-----ACAATGGAATGTTGCCAAACCGGTTCTTTCCCGTTATAGCCAT

BE/23/2011 TTATTTGACTTCCAAGTGCCACACATCACCACTATATTCATCCATGTTTTCACCGAACCAACGAGACAGATCGAAGAAGCCAGAATCTTCCGACTTTAAATTACATAAATCCAACGTATTATGACCACAGCTCGACACACAAATAGTTGCGTTACTATTCACAGTGGCATTACCTATACCCGTAACGTTGCACAACCACTGATCACCATTGTCACCAAAAACGGTTTTCCACTTAGTTGTCAACGGATCTTTCCTATGCGTAATGGTAAAATTACTACCAGTCGTCGCTTTTAGCTCATTACGAGTATTATCCGCATCCACATATATCAACGTCATAGCTAGGCACGCTATAAGTACCCCCCCCCC-----ACAATGGAATGTTGCCAAACCGGTTCTTTCCCGTTATAGCCAT

2CEN15 TTATTTGACTTCCAAGTGCCACACATCACCACTATATTCATCCATGTTTTCACCGAACCAACGAGACAGATCGAAGAAGCCAGAATCTTCCGACTTTAAATTACATAAATCCAACGTATTATGACCACAGCTCGACACACAAATAGTTGCGTTACTATTCACAGTGGCATTACCTATACCCGTAACGTTGCACAACCACTGATCACCATTGTCACCAAAAACGGTTTTCCACTTAGTTGTCAACGGATCTTTCCTATGCGTAATGGTAAAATTACTACCAGTCGTCGCTTTTAGCTCATTACGAGTATTATCCGCATCCACATATATCAACGTCATAGCTAGGCACGCTATAAGTACCCCCCCCCC-----ACAATGGAATGTTGCCAAACCGGTTCTTTCCCGTTATAGCCAT

BE/5/2012 TTATTTGACTTCCAAGTGCCACACATCACCACTATATTCATCCATGTTTTCACCGAACCAACGAGACAGATCGAAGAAGCCAGAATCTTCCGACTTTAAATTACATAAATCCAACGTATTATGACCACAGCTCGACACACAAATAGTTGCGTTACTATTCACAGTGGCATTACCTATACCCGTAACGTTGCACAACCACTGATCACCATTGTCACCAAAAACGGTTTTCCACTTAGTTGTCAACGGATCTTTCCTATGCGTAATGGTAAAATTACTACCAGTCGTCGCTTTTAGCTCATTACGAGTATTATCCGCATCCACATATATCAACGTCATAGCTAGGCACGCTATAAGTACCCCCCCCCC-----ACAATGGAATGTTGCCAAACCGGTTCTTTCCCGTTATAGCCAT

TR TTATTTGACTTCCAAGTGCCACACATCACCACTATATTCATCCATGTTTTCACCGAACCAACGAGACAGATCGAAGAAGCCAGAATCTTCCGACTTTAAATTACATAAATCCAACGTATTATGACCACAGCTCGACACACAAATAGTTGCGTTACTATTCACAGTGGCATTACCTATACCCGTAACGTTGCACAACCACTGATCACCATTGTCACCAAAAACGGTTTTCCACTTAGTTGTCAACGGATCTTTCCTATGCGTAATGGTAAAATTACTACCAGTCGTCGCTTTTAGCTCATTACGAGTATTATCCGCATCCACATATATCAACGTCATAGCTAGGCACGCTATAAGTACCCCCCCCCC-----ACAATGGAATGTTGCCAAACCGGTTCTTTCCCGTTATAGCCAT

HAN27 TTATTTGACTTCCAAGTGCCACACATCACCACTATATTCATCCATGTTTTCACCGAACCAACGAGACAGATCGAAGAAGCCAGAATCTTCCGACTTTAAATTACATAAATCCAACGTATTATGACCACAGCTCGACACACAAATAGTTGCGTTACTATTCACAGTGGCATTACCTATACCCGTAACGTTGCACAACCACTGATCACCATTGTCACCAAAAACGGTTTTCCACTTAGTTGTCAACGGATCTTTCCTATGCGTAATGGTAAAATTACTACCAGTCGTCGCTTTTAGCTCATTACGAGTATTATCCGCATCCACATATATCAACGTCATAGCTAGGCACGCTATAAGTACCCCCCCCCC-----ACAATGGAATGTTGCCAAACCGGTTCTTTCCCGTTATAGCCAT

JER3230 TTATTTGACTTCCAAGTGCCACACATCACCACTATATTCATCCATGTTTTCACCGAACCAACGAGACAGATCGAAGAAGCCAGAATCTTCCGACTTTAAATTACATAAATCCAACGTATTATGACCACAGCTCGACACACAAATAGTTGCGTTACTATTCACAGTGGCATTACCTATACCCGTAACGTTGCACAACCACTGATCACCATTGTCACCAAAAACGGTTTTCCACTTAGTTGTCAACGGATCTTTCCTATGCGTAATGGTAAAATTACTACCAGTCGTCGCTTTTAGCTCATTACGAGTATTATCCGCATCCACATATATCAACGTCATAGCTAGGCACGCTATAAGTACCCCCCCCCC-----ACAATGGAATGTTGCCAAACCGGTTCTTTCCCGTTATAGCCAT

UK/Lon6 TTATTTGACTTCCAAGTGCCACACATCACCACTATATTCATCCATGTTTTCACCGAACCAACGAGACAGATCGAAGAAGCCAGAATCTTCCGACTTTAAATTACATAAATCCAACGTATTATGACCACAGCTCGACACACAAATAGTTGCGTTACTATTCACAGTGGCATTACCTATACCCGTAACGTTGCACAACCACTGATCACCATTGTCACCAAAAACGGTTTTCCACTTAGTTGTCAACGGATCTTTCCTATGCGTAATGGTAAAATTACTACCAGTCGTCGCTTTTAGCTCATTACGAGTATTATCCGCATCCACATATATCAACGTCATAGCTAGGCACGCTATAAGTACCCCCCCCCC-----ACAATGGAATGTTGCCAAACCGGTTCTTTCCCGTTATAGCCAT

JER1289 TTATTTGACTTCCAAGTGCCACACATCACCACTATATTCATCCATGTTTTCACCGAACCAACGAGACAGATCGAAGAAGCCAGAATCTTCCGACTTTAAATTACATAAATCCAACGTATTATGACCACAGCTCGACACACAAATAGTTGCGTTACTATTCACAGTAGCATTACCTATACCCGTAACGTTGCACAACCACTGATCACCATTGTCACCAAAAACGGTTTTCCACTTAGTTGTCAACGGATCTTTCCTATGTGTAATGGTAAAATTACTACCAGTCGTCGCTTTTAGCTCATTACGAGTATTATCCGCATCCACATATATCAACGTCATAGCTAGGCACGCTATAAGTACCCCCCCCCC-----ACAATGGAATGTTGCCAAACCGGTTCTTTCCCGTTATAGCCAT

NL/Rot4 TTATTTGACTTCCAAGTGCCACACATCACCACTATATTCATCCATGTTTTCACCGAACCAACGAGACAGATCGAAGAAGCCAGAATCTTCCGACTTTAAATTACATAAATCCAACGTATTATGACCACAGCTCGACACACAAATAGTTGCGTTACTATTCACAGTAGCATTACCTATACCCGTAACGTTGCACAACCACTGATCACCATTGTCACCAAAAACGGTTTTCCACTTAGTTGTCAACGGATCTTTCCTATGTGTAATGGTAAAATTACTACCAGTCGTCGCTTTTAGCTCATTACGAGTATTATCCGCATCCACATATATCAACGTCATAGCTAGGCACGCTATAAGTACCCCCCCCCC-----ACAATGGAATGTTGCCAAACCGGTTCTTTCCCGTTATAGCCAT

W TTATTTGACTTCCAAGTGCCACACATCACCACTATATTCATCCATGTTTTCACCGAACCAACGAGACAGATCGAAGAAGCCAGAATCTTCCGACTTTAAATTACATAAATCCAACGTATTATGACCACAGCTCGACACACAAATAGTTGCGTTACTATTCACAGTGGCATTACCTATACCCGTAACGTTGCACAACCACTGATCACCATTGTCACCAAAAACGGTTTTCCACTTAGTTGTCAACGGATCTTTCCTATGCGTAATGGTAAAATTACTACCAGTCGTCGCTTTTAGCTCATTACGAGTATTATCCGCATCCACATATATCAACGTCATAGCTAGGCACGCTATAAGTACCCCCCCCCCCC---ACAATGGAATGTTGCCAAACCGGTTCTTTCCCGTTATAGCCAT

JER1070 TTATTTGACTTCCAAGTGCCACACATCACCACTATATTCATCCATGTTTTCACCGAACCAACGAGACAGATCGAAGAAGCCAGAATCTTCCGACTTTAAATTACATAAATCCAACGTATTATGACCACAGCTCGACACACAAATAGTTGCGTTACTATTCACAGTGGCATTACCTATACCCGTAACGTTGCACAACCACTGATCACCATTGTCACCAAAAACGGTTTTCCACTTAGTTGTCAACGGATCTTTCCTATGCGTAATGGTAAAATTACTACCAGTCGTCGCTTTTAGCTCATTACGAGTATTATCCGCATCCACATATATCAACGTCATAGCTAGGCACGCTATAAGTACCCCCCCCCCC----ACAATGGAATGTTGCCAAACCGGTTCTTTCCCGTTATAGCCAT

PAV26 TTATTTGACTTCCAAGTGCCACACATCACCACTATATTCATCCATGTTTTCACCGAACCAACGAGACAGATCGAAGAAGCCAGAATCTTCCGACTTTAAATTACATAAATCCAACGTATTATGACCACAGCTCGACACACAAATAGTTGCGTTACTATTCACAGTGGCATTACCTATACCCGTAACGTTGCACAACCACTGATCACCATTGTCACCAAAAACGGTTTTCCACTTAGTTGTCAACGGATCTTTCCTATGCGTAATGGTAAAATTACTACCAGTCGTCGCTTTTAGCTCATTACGAGTATTATCCGCATCCACATATATCAACGTCATAGCTAGGCACGCTATAAGTACCCCCCCCCCC----ACAATGGAATGTTGCCAAACCGGTTCTTTCCCGTTATAGCCAT

JER4053 TTATTTGACTTCCAAGTGCCACACATCACCACTATATTCATCCATGTTTTCACCGAACCAACGAGACAGATCGAAGAAGCCAGAATCTTCCGACTTTAAATTACATAAATCCAACGTATTATGACCACAGCTCGACACACAAATAGTTGCGTTACTATTCACAGTAGCATTACCTATACCCGTAACGTTGCACAACCACTGATCACCATTGTCACCAAAAACGGTTTTCCACTTAGTTGTCAACGGATCTTTCCTATGTGTAATGGTAAAATTACTACCAGTCGTCGCTTTTAGCTCATTACGAGTATTATCCGCATCCACATATATCAACGTCATAGCTAGGCACGCTATAAGTACCCCCCCCCCC----ACAATGGAATGTTGCCAAACCGGTTCTTTCCCGTTATAGCCAT

HAN20 TTATTTGACTTCCAAGTGCCACACATCACCACTATATTCATCCATGTTTTCACCGAACCAACGAGACAGATCGAAGAAGCCAGAATCTTCCGACTTTAAATTACATAAATCCAACGTATTATGACCACAGCTCGACACACAAATAGTTGCGTTACTATTCACAGTGGCATTACCTATACCCGTAACGTTGCACAACCACTGATCACCATTGTCACCAAAAACGGTTTTCCACTTAGTTGTCAACGGATCTTTCCTATGCGTAATGGTAAAATTACTACCAGTCGTCGCTTTTAGCTCATTACGAGTATTATCCGCATCCACATATATCAACGTCATAGCTAGGCACGCTATAAGTACCCCCCC--------ACAATGGAATGTTGCCAAACCGGTTCTTTCCCGTTATAGCCAT

PAV6 TTATTTGACTTCCAAGTGCCACACATCACCACTATATTCATCCATGTTTTCACCGAACCAACGAGACAGATCGAAGAAGCCAGAATCTTCCGACTTTAAATTACATAAATCCAACGTATTATGACCACAGCTCGACACACAAATAGTTGCGTTACTATTCACAGTGGCATTACCTATACCCGTAACGTTGCACAACCACTGATCACCATTGTCACCAAAAACGGTTTTCCACTTAGTTGTCAACGGATCTTTCCTATGCGTAATGGTAAAATTACTACCAGTCGTCGCTTTTAGCTCATTACGAGTATTATCCGCATCCACATATATCAACGTCATAGCTAGGCACGCTATAAGTACCCCCCCCC------ACAATGGAATGTTGCCAAACCGGTTCTTTCCCGTTATAGCCAT

BE/39/2011 TTATTTGACTTCCAAGTACCACACATCACCACTATATTCATCCATGTTTTCACCGAACCAACGAGACAGATCGAAGAAGCCAGAATCTTCCGACTTTAAATTACATAAATCCAACGTATTATGACCACAGCTCGACACACAAATAGTTGCGTTACTATTCACAGTAGCATTACCTATACCCGTAACGTTGCACAACCACTGATCACCATTGTCACCAAAAACGGTTTTCCACTTAGTTGTCAACGGATCTTTCCTATGTGTAATGGTAAAATTACTACCAGTCGTCGCTTTTAGCTCATTACGAGTATTATCCGCATCCACATATATCAACGTCATAGCTAGGCACGCTATAAGTACCCCCCCCC------ACAATGGAATGTTGCCAAACCGGTTCTTTCCCGTTATAGCCAT

BE/35/2011 TTATTTGACTTCCAAGTGCCACACATCACCACTATATTCATCCATGTTTTCACCGAACCAACGAGACAGATCGAAGAAGCCAGAATCTTCCGACTTTAAATTACATAAATCCAACGTATTATGACCACAGCTCGACACACAAATAGTTGCGTTACTATTCACAGTAGCATTACCTATACCCGTAACGTTGCACAACCACTGATCACCATTGTCACCAAAAACGGTTTTCCACTTAGTTGTCAACGGATCTTTCCTATGTGTAATGGTAAAATTACTACCAGTCGTCGCTTTTAGCTCATTACGAGTATTATCCGCATCCACATATATCAACGTCATAGCTAGGCACGCTATAAGTACCCCCCCCC------ACAATGGAATGTTGCCAAACCGGTTCTTTCCCGTTATAGCCAT

BE/12/2012 G2 CTAATTTTTAGTAGTCAAATACCATAATTCACCGCTGTACTCGTCTTTATTCTCTCCGAACCAACGACCAAGGTCGACAACGCCATCGTTACCCTTCGTGATATTGCACAGATCTAAAGATGTATGAGTACAATTCGTTACACAAACGCTTGATCCATTATTCACTAGAGGTGCATGTCTCCCTGTTACATTACATAACCATCCTTGATTCAGATGGCTCCATTTCGTAGTCATCGGGTCGTTTTTGTGTTCAATGGTTACATTGTCACCAC---------------TTTTAACCTCT-----ATTGAAACCCGCACCTTCATGTATAAACACCGTCATGAAACACGCTATAAGTACCCCCC---------ACAATGGAATGCTGCCAAACCGGTTATTTCCCGTTATAGCCAT

Pat_H CTAATTTTTAGCAGTCAAATACCATAATTCACCGCTGTACTCGTCTCTATTCTCTCCAAACCAACGACCAAGATCGACAACGCCATCGTTACCCTTCGTGATATTGCACAGATCTAGAGATGTATGAGTACAGTTCGTTACACAAACGCTTGATCCATTATTCACTAGAGGTGCATGTCTCCCTGTTACATTACATAACCATCCTTGATCCAGATGGCTCCATTTCGTAGTCATCGGGTCGTTTTTGTGTTCAATGGTTACATTGTCACCAC---------------TTTTAACCTCTACTTTATTGAAACCCGCACCTTCATGTATAAACACCGTCATGAAACACGCTATAAGTACCCCCCCCCC-----ACAATGGAATGTTGCCAAACCGGTTATTTCCCGTTATAGCCAT

JER4755 CTAATTTTTAGTAGTCAAATACCATAATTCACCGCTGTACTCGTCTTTATTCTCTCCGAGCCAACGACCAAGGTCGACAACGCCATCGTCACCCTTCGTGATATTGCACAGATCTAGAGATGTATGAGTACAGTTCGTTACACAAACGCTTGATCCATTATTCACTAGAGGTGCATGTCTCCCTGTTACATTACATAACCATCCTTGATCCAGATGGCTCCATTTCGTAGTCATCGGGTCGTTTTTGTGTTCAATGGTTACATTGTCACCAC---------------TTTTAACCTCTACTTTATTGAAACCCGCACCTTCATGTATAAACACCGTCATGAAATACGCTATAAGTACCCCCCCCCC-----ACAATGGAATGTTGCCAAACCGGTTCTTTCCCGTTATAGCCAT

U4 CTAATTTTTAGTAGTCAAATACCATAATTCACCGCTGTACTCGTCTTTATTCTCTCCGAGCCAACGACCAAGGTCGACAACGCCATCGTCACCCTTCGTGATATTGCACAGATCTAGAGATGTATGAGTACAGTTCGTTACACAAACGCTTGATCCATTATTCACTAGAGGTGCATGTCTCCCTGTTACATTACATAACCATCCTTGATCCAGATGGCTCCATTTCGTAGTCATCGGGTCGTTTTTGTGTTCAATGGTTACATTGTCACCAC---------------TTTTAACCTCTACTTTATTGAAACCCGCACCTTCATGTATAAACACCGTCATGAAACACGCTATAAGTACCCCCCCCCC-----ACAATGGAATGTTGCCAAACCGGTTCTTTCCCGTTATAGCCAT

AF1 CTAATTTTTAGTAGTCAAATACCATAATTCACCGCTGTACTCGTCTTTATTCTCTCCGAACCAACGACCAAGGTCGACAACGCCATCGTTACCCTTCGTGATATTGCACAGATCTAAAGATGTATGAGTACAATTCGTTACACAAACGCTTGATCAATTATTCACTAGAGGTGCATGTCTCCTTGTTACATTACATAACCATCCTTGATTCAGATGGCTCCATTTCGTAGTCATCGGGTCGTTTTTGTGTTCAATGGTTACATTGTCACCAC---------------TTTTAACCTCTACTTTATTGAAACCCGCACCTTCATGTATAAACACCGTCATGAAACACGCTATAAGTACCCCCCCC-------ACAATGGAATGCTGCCAAACCGGTTATTTCCCGTTATAGCCAT

NL/Rot2 CTAATTTTTAGTAGTCAAATACCATAATTCACCGCTGTACTCGTCTTTATTCTCTCCGAACCAACGACCAAGGTCGACGACGCCATCGTTACCCTTCGTGATATTGCACAGATCTAGAGATGTATGAGTACAATTCGTTACACAAACGCTTGATCCATTATTCACTAGAGGTGCATGTCTCCCTGTTACATTACATAACCATCCTTGATTCAGATGGCTCCATTTCGTAGTCATCGGGTCGTTTTTGTGTTCAATGGTTACATTGTCACCAC---------------TTTTAACCTCTACTTTATTGAAACCCGCAACTTCATGTATAAACACCGTCATGAAACACGCTATAAGTACCCCCCCCCC-----ACGATGGAATGCTGCCAAACCGGTTATTTCCCGTTATAGCCAT

BE/1/2012 CTAATTTTTAGTAGTCAAATACCATAATTCACCGCTGTACTCGTCTTTATTCTCTCCGAACCAACGACCAAGGTCGACAACGCCATCGTTACCCTTCGTGATATTGCACAGATCTAAAGATGTATGAGTACAATTCGTTACACAAACGCTTAATCCATTATTCACTAGAGATGCATGTCTCCCTGTTACATTACATAACCATCCTTGATTCAGATGGCTCCATTTCGTAGTCATCGGGTCGTTTTTGTGTTCAATGGTTACATTGTCACCAC---------------TTTTAACCTCTACTTTATTGAAACCCGCACCTTCATGTATAAACACCGTCATGAAACACGCTATAAGTACCCCCCCTCC-----ACAATGGAATGCTGCCAAACCGGTTATTTCCCGTTATAGCCAT

JER847 CTAATTTTTAGTAGTCAAATACCATAATTCACCGCTGTACTCGTCTTTATTCTCTCCGAACCAACGACCAAGGTCGACAACGCCATCGTTACCCTTCGTGATATTGCACAGATCTAAAGATGTATGAGTACAATTCGTTACACAAACGCTTGATCAATTATTCACTAGAGGTGCATGTCTCCTTGTTACATTACATAACCATCCTTGATTCAGATGGCTCCATTTCGTAGTCATCGGGTCGTTTTTGTGTTCAATGGTTACATTGTCACCAC---------------TTTTAACCTCTACTTTATTGAAACCCGCACCTTCATGTATAAACACCGTCATGAAACACGCTATAAGTACCCCCCCCCC-----ACAATGGAATGCTGCCAAACCGGTTATTTCCCGTTATAGCCAT

NAN4LA CTAATTTTTAGTAGTCAAATACCATAATTCACCGCTGTACTCGTCTTTATTCTCTCCGAACCAACGACCAAGGTCGACAACGCCATCGTTACCCTTCGTGATATTGCACAGATCTAAAGATGTATGAGTACAATTCGTTACACAAACGCTTGATCAATTATTCACTAGAGGTGCATGTCTCCTTGTTACATTACATAACCATCCTTGATTCAGATGGCTCCATTTCGTAGTCATCGGGTCGTTTTTGTGTTCAATGGTTACATTGTCACCAC---------------TTTTAACCTCTACTTTATTGAAACCCGCACCTTCATGTATAAACACCGTCATGAAACACGCTATAAGTACCCCCCCCCC-----ACAATGGAATGCTGCCAAACCGGTTATTTCCCGTTATAGCCAT

BE/4/2012 CTAATTTTTAGTAGTCAAATACCATAATTCACCGCTGTACTCGTCTTTATTCTCTCCGAACCAACGACCAAGGTCGACAACGCCATCGTTACCCTTCGTGATATTGCACAAATCTAGAGATGTATGAGTACAATTCGTTACACAAACGCTTGATCCATTATTCACTAGAGGTGCATGTCTCCCTGTTACATTACATAACCATCCTTGATTCAGATGGCTCCATTTCGTAGTCATCGGGTCGTTTTTGTGTTCAATGGTTACATTGTCACCAC---------------TTTTAACCTCTACTTTATTGAAACCCGCACCTTCATGTATAAACACCGTCATGAAACACGCTATAAGTACCCCCCCCCC-----ACAATGGAATGCTGCCAAACCGGTTATTTCCCGTTATAGCCAT

JER4559 CTAATTTTTAGTAGTCAAATACCATAATTCACCGCTGTACTCGTCTTTATTCTCTCCGAACCAACGACCAAGGTCGACAACGCCATCGTTACCCTTCGTGATATTGCACAGATCTAAAGATGTATGAGTACAATTCGTTACACAAACGCTTGATCCATTATTCACTAGAGGTGCATGTCTCCCTGTTACATTACATAACCATCCTTGATTCAGATGGCTCCATTTCGTAGTCATCGGGTCGTTTTTGTGTTCAATGGTTACATTGTCACCAC---------------TTTTAACCTCTACTTTATTGAAACCCGCACCTTCATGTATAAACACCGTCATGAAACACGCTATAAGTACCCCCCCCCC-----ACAATGGAATGCTGCCAAACCGGTTATTTCCCGTTATAGCCAT

BE/15/2011 CTAATTTTTAGTAGTCAAATACCATAATTCACCGCTGTACTCGTCTTTATTCTCTCCGAACCAACGACCAAGGTCGACAACGCCATCGTTACCCTTCGTGATATTGCACAGATCTAAAGATGTATGAGTACAATTCGTTACACAAACGCTTGATCCATTATTCACTAGAGGTGCATGTCTCCCTGTTACATTACATAACCATCCTTGATTCAGATGGCTCCATTTCGTAGTCATCGGGTCGTTTTTGTGTTCAATGGTTACATTGTCACCAC---------------TTTTAACCTCTACTTTATTGAAACCCGCACCTTCATGTATAAACACCGTCATGAAACACGCTATAAGTACCCCCCCCCC-----ACAATGGAATGCTGCCAAACCGGTTATTTCCCGTTATAGCCAT

HAN24 CTAATTTTTAGTAGTCAAATACCATAATTCACCGCTGTACTCGTCTTTATTCTCTCCGAACCAACGACCAAGGTCGACAACGCCATCGTTACCCTTCGTGATATTGCACAGATCTAAAGATGTATGAGTACAATTCGTTACACAAACGCTTGATCCATTATTCACTAGAGGTGCATGTCTCCCTGTTACATTACATAACCATCCTTGATTCAGATGGCTCCATTTCGTAGTCATCGGGTCGTTTTTGTGTTCAATGGTTACATTGTCACCAC---------------TTTTAACCTCTACTTTATTGAAACCCGCACCTTCATGTATAAACACCGTCATGAAACACGCTATAAGTACCCCCCCCCC-----ACAATGGAATGCTGCCAAACCGGTTATTTCCCGTTATAGCCAT

Towne CTAATTTTTAGTAGTCAAATACCATAATTCACCGCTGTACTCGTCTTTATTCTCTCCGAACCAACGACCAAGGTCGACAACGCCATCGTTACCCTTCGTGATATTGCACAGATCTAAAGATGTATGAGTACAATTCGTTACACAAACGCTTGATCCATTATTCACTAGAGGTGCATGTCTCCCTGTTACATTACATAACCATCCTTGATTCAGATGGCTCCATTTCGTAGTCATCGGGTCGTTTTTGTGTTCAATGGTTACATTGTCACCAC---------------TTTTAACCTCTACTTTATTGAAACCCGCACCTTCATGTATAAACACCGTCATGAAACACGCTATAAGTACCCCCCCCCC-----ACAATGGAATGCTGCCAAACCGGTTATTTCCCGTTATAGCCAT

6397 CTAATTTTTAGTAGTCAAATACCATAATTCACCGCTGTACTCGTCTTTATTCTCTCCGAACCAACGACCAAGGTCGACAACGCCATCGTTACCCTTCGTGATATTGCACAGATCTAAAGATGTATGAGTACAATTCGTTACACAAACGCTTGATCCATTATTCACTAGAGGTGCATGTCTCCCTGTTACATTACATAACCATCCTTGATTCAGATGGCTCCATTTCGTAGTCATCGGGTCGTCTTTGTGTTCAATGGTTACATTGTCACCAC---------------TTTTAACCTCTACTTTATTGAAACCCGCACCTTCATGTATAAACACCGTCATGAAACACGCTATAAGTACCCCCCCCCC-----ACAATGGAATGCTGCCAAACCGGTTATTTCCCGTTATAGCCAT

CZ/1/2012 CTAATTTTTAGTAGTCAAATACCATAATTCACCGCTGTACTCGTCTTTATTCTCTCCGAACCAACGACCAAGGTCGACAACGCCGTCGTTACCCTTCGTGATATTGCACAGATCTAGAGATGTATGAGTACAATTCGTTACACAAACGCTTGATCCATTATTCACTAGAGGTGCATGCCTCCCTGTCACATTACATAACCATCCTTGATTCAGATGGCTCCATTTCGTAGTCATCGGGTCGTTTTTGTGTTCAATGGTTACATTGTCACCAC---------------CTTTAACCTCTACTTTATTGAAACCCGCACCTTCATGTATAAACACCGTCATGAAACACGCTATAAGTACCCCCCCCCCC----ACAATGGAATGTTGCCAAACCGGTTCTTTCCCGTTATAGCCAT

HANRTR1A CTAATTTTTAGTAGTCAAATACCATAATTCACCGCTGTACTCGTCTTTATTCTCTCCGAACCAACGACCAAGGTCGACAACGCCGTCGTTACCCTTCGTGATATTGCACAGATCTAGAGATGTATGAGTACAATTCGTTACACAAACGCTTGATCCATTATTCACTAGAGGTGCATGCCTCCCTGTCACATTACATAACCATCCTTGATTCAGATGGCTCCATTTCGTAGTCATCGGGTCGTTTTTGTGTTCAATGGTTACATTGTCACCAC---------------CTTTAACCTCTACTTTATTGAAACCCGCACCTTCATGTATAAACACCGTCATGAAACACGCTATAAGTACCCCCCCCCCCC---ACAATGGAATGTTGCCAAACCGGTTCTTTCCCGTTATAGCCAT

BE/24/2011 CTAATTTTTAGTAGTCAAATACCATAATTCACCGCTGTACTCGTCTTTATTCTCTCCGAACCAACGACCAAGGTCGACAACGCCGTCGTTACCCTTCGTGATATTGCACAGATCTAGAGATGTATGAGTACAATTCGTTACACAAACGCTTGATCCATTATTCACTAGAGGTGCATGCCTCCCTGTCACATTACATAACCATCCTTGATTCAGATGGCTCCATTTCGTAGTCATCGGGTCGTTTTTGTGTTCAATGGTTACATTGTCACCAC---------------CTTTAACCTCTACTTTATTGAAACCCGCACCTTCATGTATAAACACCGTCATGAAACACGCTATAAGTACCCCCCCCCC-----ACAATGGAATGTTGCCAAACCGGTTCTTTCCCGTTATAGCCAT

BE/14/2011 CTAATTTTTAGTAGTCAAATACCATAATTCACCGCTGTACTCGTCTTTATTCTCTCCGAACCAACGACCAAGGTCGACAACGCCGTCGTTACCCTTCGTGATATTGCACAGATCTAGAGATGTATGAGTACAATTCGTTACACAAACGCTTGATCCATTATTCACTAGAGGTGCATGCCTCCCTGTCACATTACATAACCATCCTTGATTCAGATGGCTCCATTTCGTAGTCATCGGGTCGTTTTTGTGTTCAATGGTTACATTGTCACCAC---------------CTTTAACCTCTACTTTATTGAAACCCGCACCTTCATGTATAAACACCGTCATGAAACACGCTATAAGTACCCCCCCCCC-----ACAATGGAATGTTGCCAAACCGGTTCTTTCCCGTTATAGCCAT

BE/9/2011 CTAATTTTTAGTAGTCAAATACCATAATTCACCGCTGTACTCGTCTTTATTCTCTCCGAACCAACGACCAAGGTCGACAACGCCGTCGTTACCCTTCGTGATATTGCACAGATCTAGAGATGTATGAGTACAATTCGTTACACAAACGCTTGATCCATTATTCACTAGAGGTGCATGCCTCCCTGTCACATTACATAACCATCCTTGATTCAGATGGCTCCATTTCGTAGTCATCGGGTCGTTTTTGTGTTCAATGGTTACATTGTCACCAC---------------CTTTAACCTCTACTTTATTGAAACCCGCACCTTCATGTATAAACACCGTCATGAAACACGCTATAAGTACCCCCCCCC------ACAATGGAATGTTGCCAAACCGGTTCTTTCCCGTTATAGCCAT

BE/1/2010 CTAATTTTTAGTAGTCAAATACCATAATTCACCGCTGTACTCGTCTTTATTCTCTCCGAACCAACGACCAAGGTCGACAACGCCATCGTCACCCTTCGTGATATTGCACAGATCTAGAGATGTATGAGTACAGTTCGTTACACAAACGCTTGATCCATTATTCACTAGAGGTGCATGTCTCCCTGTTACATTACATAACCATCCTTGATCCAGATGGCTCCATTTCGTAGTCATCGGGTCGTTTTTGTGTTCAATGGTTACATTGTCACCAC---------------TTTTAACCTCTACTTTATTGAAACCCGCACCTTCATGTATAAACACCGTCATGAAACACGCTATAAGTACCCCCCCCC------ACAATGGAATGCTGCCAAACCGGTTATTTCCCGTTATAGCCAT

BE/4/2011 CTAATTTTTAGTAGTCAAATACCATAATTCACCGCTGTACTCGTCTTTATTCTCTCCGAACCAACGACCAAGGTCGACAACGCCATCGTTACCCTTCGTGATATTGCACAGATCTAAAGATGTATGAGTACAATTCGTTACACAAACGCTTGATCCATTATTCACTAGAGGTGCATGTCTCCCTGTTACATTACATAACCATCCTTGATTCAGATGGCTCCATTTCGTAGTCATCGGGTCGTTTTTGTGTTCAATGGTTACATTGTTACCAC---------------TTTTAACCTCTACTTTATTGAAACCCGCACCTTCATGTATAAACACCGTCATGAAACACGCTATAAGTACCCCCCCCC------ACAATGGAATGCTGCCAAACCGGTTATTTCCCGTTATAGCCAT

UK/Lon5 CTAATTTTTAGTAGTCAAATACCATAATTCACCGCTGTACTCGTCTTTATTCTCTCCGAACCAACGACCAAGGTCGACAACGCCATCGTTACCCTTCGTGATATTGCACAGATCTAAAGATGTATGAGTACAATTCGTTACACAAACGCTTGATCCATTATTCACTAGAGGTGCATGTCTCCCTGTTACATTACATAACCATCCTTGATTCAGATGGCTCCATTTCGTAGTCATCGGGTCGTTTTTGTGTTCAATGGTTACATTGTCACCAC---------------TTTTAACCTCTACTTTATTGAAACCCGCACCTTCATGTATAAACACTGTCATGAAACACGCTATAAGTACCCCCCCCC------ACAATGGAATGCTGCCAAACCGGTTATTTCCCGTTATAGCCAT

NL/Rot6 CTAATTTTTAGTAGTCAAATACCATAACTCACCGCTGTACTCGTCTTTATTCTCTCCGAACCAACGACCAAGGTCGACAACGCCATCGTTACCCTTCGTGATATTGCACAGATCTAGAGATGTATGAGTACAATTCGTTACACAAACGCTTGATCCATTATTCACTAGAGGTGCATGTCTCCCTGTTACATTACATAACCATCCTTGATTCAGATGGCTCCATTTCGTAGTCATCGGGTCGTTTTTGTGTTCAATGGTTACATTGTCACCAC---------------TTTTAACCTCTACTTTATTGAAACCCGCACCTTCATGTATAAACACCGTCATGAAACACGCTATAAGTACCCCCCCCC------ACAATGGAATGCTGCCAAACCGGTTATTTCCCGTTATAGCCAT

BE/22/2011 CTAATTTTTAGTAGTCAAATACCATAATTCACCGCTGTACTCGTCTTTATTCTCTCCGAACCAACGACCAAGGTCGACAACGCCATCGTTACCCTTCGTGATATTGCACAGATCTAAAGATGTATGAGTACAATTCGTTACACAAACGCTTGATCCATTATTCACTAGAGGTGCATGTCTCCCTGTTACATTACATAACCATCCTTGATTCAGATGGCTCCATTTCGTAGTCATCGGGTCGTTT--GTGTTCAATGGTTACATTGTCACCAC---------------TTTTAACCTCTACTTTATTGAAACCCGCACCTTCATGTATAAACACCGTCATGAAACACGCTATAAGTACCCCCCCCCCC----ACAATGGAATGCTGCCAAACCGGTTATTTCCCGTTATAGCCAT

HAN40 CTAATTTTTAGTAGTCAAATACCATAATTCACCGCTGTACTCGTCTTTATTCTCTCCGAACCAACGACCAAGGTCGACAACGCCATCGTTACCCTTCGTGATATTGCACAGATCTAAAGATGTATGAGTACAATTCGTTACACAAACGCTTGATCCATTATTCACTAGAGGTGCATGTCTCCCTGTTACATTACATAACCATCCTTGATTCAGATGGCTCCATTTCGTAGTCATCGGGTCGTTTTTGTGTTCAATGGTTACATTGTCACCAC---------------TTTTAACCTCTACTTTATTGAAACCCGCACCTTCATGTATAAACACCGTCATGAAACACGCTATAAGTACCCCCCCCCCCC---ACAATGGAATGCTGCCAAACCGGTTATTTCCCGTTATAGCCAT

HAN33 CTAATTTTTAGCAGTCAAATACCATAATTCACCGCTGTACTCGTCTCTATTCTCTCCGAACCAACGACCAAGGTCGACAACGCCATCGTTACCCTTCGTGATATTGCACAGATCTAGAGATGTATGAGTACAATTCGTTACACAAACGCTTGATCCATTATTCACTAGAGGTGCATGTCTCCCTGTTACATTACATAACCATCCTTGATTCAGATGGCTCCATTTCGTAGTCATCGGGTCGTTTTTGTGTTCAATGGTTACATTGTCACCAC---------------TTTTAACCTCTACCTTATTGAAACCCGCACCTTCATGTATAAACACCGTCATGAAACACGCTATAAGTACCCCCCCCCCC----ACAATGGAATGCTGCCAAACCGGTTATTTCCCGTTATAGCCAT

HANSCTR4 CTAATTTTTAGTAGTCAAATACCATAATTCACCGCTGTACTCGTCTTTATTCTCTCCGAACCAACGACCAAGGTCGACAACGCCATCGTTACCCTTCGTGATATTGCACAGATCTAAAGATGTATGAGTACAATTCGTTACACAAACGCTTGATCCATTATTCACTAGAGGTGCATGTCTCCCTGTTACATTACATAACCATCCTTGATTCAGATGGCTCCATTTCGTAGTCATCGGGTCGTTTTTGTGTTCAATGGTTACATTGTCACCAC---------------TTTTAACCTCTACTTTATTGAAACCCGCACCTTCATGTATAAACACCGTCATGAAACACGCTATAAGTACCCCCCCCCCC----ACAATGGAATGCTGCCAAACCGGTTATTTCCCGTTATAGCCAT

BE/21/2010 G6 CTAATTTTTAGCAGTCAAATACCATAATTCACCGCTGTACTCGTCTCTATTCTCTCCAAACCAACGACCAAGATCGACAACGCCGTCATTACCCTTCGTAATATTACACAAATCTAGAGACGTATGGGTACAATTCGTTACACAGACGCTTGATCCGTTATTCACTAAAGGTGCATGCCTCCCGGTTACATTACATAACCATCCTTTATCCCAATGGCTCCATCTAGTAGTCATCGGATCTTTTTTGTGTTCAATGGTTACATTATTACCAC---------------TGGTAACTTCCACTCTATTAAAACCCGCACTTTCATGTATAAACACCGTTATGAAACACGCTATAAGTACCCCCCCCC------ACAATGGAATGCTGCCAAACTGGTTATTTCCCATTATAGCCAT

NANU CTAATTTTTAGCAGTCAAATACCATAATTCACCGCTGTACTCGTCTCTATTCTCTCCAAACCAACGACCAAGATCGACAACGCCGTCATTACCCTTCGTAATATTACACAAATCTAGAGACGTATGGGTACAATTCGTTACACAGACGCTTGATCCGTTATTCACTAAAGGTGCATGCCTCCCGGTTACATTACATAACCATCCTTTATCCCAATGGCTCCATCTAGTAGTCATCGGATCTTTTTTGTGTTCAATGGTTACATTATTACCAC---------------TGGTAACTTCCACTCTATTAAAACCCGCACTTTCATGTATAAACACCGTTATGAAACACGCTATAAGTACCCCCCCCCCC----ACAATGGAATGCTGCCAAACTGGTTATTTCCCATTATAGCCAT

JER2002 CTAATTTTTAGCAGTCAAATACCATAATTCACCGCTGTACTCGTCTCTATTCTCTCCAAACCAACGACCAAGATCGACAACGCCGTCATTACCCTTCGTAATATTACACAAATCTAGAGACGTATGGGTACAATTCGTTACACAGACGCTTGATCCGTTATTCACTAAAGGTGCATGCCTCCCGGTTACATTACATAACCATCCTTTATCCCAATGGCTCCATCTAGTAGTCATCGGATCTTTTTTGTGTTCAATGGTTACATTATTACCAC---------------TGGTAACTTCCACTCTATTAAAACCCGCACTTTCATGTATAAACACCGTTATGAAACACGCTATAAGTACCCCCCCCCCC----ACAATGGAATGCTGCCAAACTGGTTATTTCCCATTATAGCCAT

JER3855 CTAATTTTTAGCAGTCAAATACCATAATTCACCGCTGTACTCGTCTCTATTCTCTCCAAACCAACGACCAAGATCGACAACGCCGTCATTACCCTTCGTAATATTACACAAATCTAGAGACGTATGGGTACAATTCGTTACACAGACGCTTGATCCGTTATTCACTAAAGGTGCATGCCTCCCGGTTACATTACATAACCATCCTTTATCCCAATGGCTCCATCTAGTAGTCATCGGATCTTTTTTGTGTTCAATGGTTACATTATTACCAC---------------TGGTAACTTCCACTCTATTAAAACCCGCACTTTCATGTATAAACACCGTTATGAAACACGCTATAAGTACCCCCCCCCCC----ACAATGGAATGCTGCCAAACTGGTTATTTCCCATTATAGCCAT

Davis CTAATTTTTAGCAGTCAAATACCATAATTCACCGCTGTACTCGTCTCTATTCTCTCCAAACCAACGACCAAGATCGACAACGCCGTCATTACCCTTCGTAATATTACACAAATCTAGAGACGTATGGGTACAATTCGTTACACAGACGCTTGATCCGTTATTCACTAAAGGTGCATGCCTCCCGGTTACATTACATAACCATCCTTTATCCCAATGGCTCCATCTAGTAGTCATCGGATCTTTTTTGTGTTCAATGGTTACATTATTACCAC---------------TGGTAACTTCCACTCTATTAAAACCCGCACTTTCATGTATAAACACCGTTATGAAACACGCTATAAGTACCCCCCCCCCC----ACAATGGAATGCTGCCAAACTGGTTATTTCCCATTATAGCCAT

PAV31 CTAATTTTTAGCAGTCAAATACCATAATTCACCGCTGTACTCGTCTCTATTCTCTCCAAACCAACGACCAAGATCGACAACGCCGTCATTACCCTTCGTAATATTACACAAATCTAGAGACGTATGGGTACAATTCGTTACACAGACGCTTGATCCGTTATTCACTAAAGGTGCATGCCTCCCGGTTACATTACATAACCATCCTTTATCCCAATGGCTCCATCTAGTAGTCATCGGATCTTTTTTGTGTTCAATGGTTACATTATTACCAC---------------TGGTAACTTCCACTCTATTAAAACCCGCACTTTCATGTATAAACACCGTTATGAAACACGCTATAAGTACCCCCCCCCC-----ACAATGGAATGCTGCCAAACTGGTTATTTCCCATTATAGCCAT

HAN13 CTAATTTTTAGCAGTCAAATACCATAATTCACCGCTGTACTCGTCTCTATTCTCTCCAAACCAACGACCAAGATCGACAACGCCGTCATTACCCTTCGTAATATTACACAAATCTAGAGACGTATGGGTACAATTCGTTACACAGACGCTTGATCCGTTATTCACTAAAGGTGCATGCCTCCCGGTTACATTACATAACCATCCTTTATCCCAATGGCTCCATCTAGTAGTCATCGGATCTTTTTTGTGTTCAATGGTTACATTATTACCAC---------------TGGTAACTTCCACTCTATTAAAACCCGCACTTTCATGTATAAACACCGTTATGAAACACGCTATAAGTACCCCCCCCCC-----ACAATGGAATGCTGCCAAACTGGTTATTTCCCATTATAGCCAT

PAV1 CTAATTTTTAGCAGTCAAATACCATAATTCACCGCTGTACTCGTCTCTATTCTCTCCAAACCAACGACCAAGATCGACAACGCCGTCATTACCCTTCGTAATATTACACAAATCTAGAGACGTATGGGTACAATTCGTTACACAGACGCTTGATCCGTTATTCACTAAAGGTGCATGCCTCCCGGTTACATTACATAACCATCCTTTATCCCAATGGCTCCATCTAGTAGTCATCGGATCTTTTTTGTGTTCAATGGTTACATTATTACCAC---------------TGGTAACTTCCACTCTATTAAAACCCGCACTTTCATGTATAAACACCGTTATGAAACACGCTATAAGTACCCCCCCCCC-----ACAATGGAATGCTGCCAAACTGGTTATTTCCCATTATAGCCAT

PAV21 CTAATTTTTAGCAGTCAAATACCATAATTCACCGCTGTACTCGTCTCTATTCTCTCCAAACCAACGACCAAGATCGACAACGCCGTCATTACCCTTCGTAATATTACACAAATCTAGAGACGTATGGGTACAATTCGTTACACAGACGCTTGATCCGTTATTCACTAAAGGTGCATGCCTCCCGGTTACATTACATAACCATCCTTTATCCCAATGGCTCCATCTAGTAGTCATCGGATCTTTTTTGTGTTCAATGGTTACATTATTACCAC---------------TGGTAACTTCCACTCTATTAAAACCCGCACTTTCATGTATAAACACCGTTATGAAACACGCTATAAGTACCCCCCCCCC-----ACAATGGAATGCTGCCAAACTGGTTATTTCCCATTATAGCCAT

HANSCTR2 CTAATTTTTAGCAGTCAAATACCATAATTCACCGCTGTACTCGTCTCTATTCTCTCCAAACCAACGACCAAGATCGACAACGCCGTCATTACCCTTCGTAATATTACACAAATCTAGAGACGTATGGGTACAATTCGTTACACAGACGCTTGATCCGTTATTCACTAAAGGTGCATGCCTCCCGGTTACATTACATAACCATCCTTTATCCCAATGGCTCCATCTAGTAGTCATCGGATCTTTTTTGTGTTCAATGGTTACATTATTACCAC---------------TGGTAACTTCCACTCTATTAAAACCCGCACTTTCATGTATAAACACCGTTATGAAACACGCTATAAGTACCCCCCCCCC-----ACAATGGAATGCTGCCAAACTGGTTATTTCCCATTATAGCCAT

JER893 G6^2 CTAATTTTTAGCAGTCAAATACCATAATTCACCGCTGTACTCGTCTCTATTCTCTCCAAACCAACGACCAAGATCGACAACGCCGTCATTACC---------------------AAGAGACGTATGGGTACAATTCGTTACACAGACGCTTGATGCGTTATTCACTAAAGGTGCATGCCTCCCGGTTACATTACATAACCATCCTTTATCCCAATGGCTCCATCTAGTAGTCATCGGATCTTTTTTGTGTTCAATGGTTACATTGTCACCAC---------------TTTTAACCTCTACTTTATTGAAACCCGCACCTTCATGTATAAACACCGTCATGAAACACGCTATAAGTACCCCCCCCCC-----ACAATGGAATGCTGCCAAACCGGTTATTTCCCGTTATAGCCAT

PRA3 CTAATTTTTAGCAGTCAAATACCATAATTCACCGCTGTACTCGTCTCTATTCTCTCCAAACCAACGACCAAGATCGACAACGCCGTCATTACCCTTCGTAATATTACACAAATCTAGAGACGTATGGGTACAATTCGTTACACAGACGCTTGATGCGTTATTCACTAAAGGTGCATGCCTCCCGGTTACATTACATAACCATCCTTTATCCCAATGGCTCCATCTAGTAGTCATCGGATCTTTTTTGTGTTCAATGGTTACATTGTCACCAC---------------TTTTAACCTCTACTTTATTGAAACCCGCACCTTCATGTATAAACACCGTCATGAAACACGCTATAAGTACCCCCC---------ACAATGGAATGCTGCCAAACCGGTTATTTCCCGTTATAGCCAT

HANRTR10 CTAATTTTTAGCAGTCAAATACCATAATTCACCGCTGTACTCGTCTCTATTCTCTCCAAACCAACGACCAAGATCGACAACGCCGTCATTACCCTTCGTAATATTACACAAATCTAGAGACGTATGGGTACAATTCGTTACACAGACGCTTGATGCGTTATTCACTAAAGGTGCATGCCTCCCGGTTACATTACATAACCATCCTTTATCCCAATGGCTCCATCTAGTAGTCATCGGATCTTTTTTGTGTTCAATGGTTACATTGTCACCAC---------------TTTTAACCTCTACTTTATTGAAACCCGCACCTTCATGTATAAACACCGTCATGAAACACGCTATAAGTACCCCCCCCC------ACAATGGAATGCTGCCAAACCGGTTATTTCCCGTTATAGCCAT

HANRTR4 CTAATTTTTAGCAGTCAAATACCATAATTCACCGCTGTACTCGTCTCTATTCTCTCCAAACCAACGACCAAGATCGACAACGCCGTCATTACCCTTCGTAATATTACACAAATCTAGAGACGTATGGGTACAATTCGTTACACAGACGCTTGATGCGTTATTCACTAAAGGTGCATGCCTCCCGGTTACATTACATAACCATCCTTTATCCCAATGGCTCCATCTAGTAGTCATCGGATCTTTTTTGTGTTCAATGGTTACATTGTCACCAC---------------TTTTAACCTCTACTTTATTGAAACCCGCACCTTCATGTATAAACACCGTCATGAAACACGCTATAAGTACCCCCCCCC------ACAATGGAATGCTGCCAAACCGGTTATTTCCCGTTATAGCCAT

U11 CTAATTTTTAGCAGTCAAATACCATAATTCACCGCTGTACTCGTCTCTATTCTCTCCAAACCAACGACCAAGATCGACAACGCCGTCATTACCCTTCGTAATATTACACAAATCTAGAGACGTATGGGTACAATTCGTTACACAGACGCTTGATGCGTTATTCACTAAAGGTGCATGCCTCCCGGTTACATTACATAACCATCCTTTATCCCAATGGCTCCATCTAGTAGTCATCGGATCTTTTTTGTGTTCAATGGTTACATTGTCACCAC---------------TTTTAACCTCTACTTTATTGAAACCCGCACCTTCATGTATAAACACCGTCATGAAACACGCTATAAGTACCCCCCCCCCCC---ACAATGGAATGCTGCCAAACCGGTTATTTCCCGTTATAGCCAT

BE/13/2010 CTAATTTTTAGCAGTCAAATACCATAATTCACCGCTGTACTCGTCTCTATTCTCTCCGAACCAACGACCAAGATCGACAACGCCGTCATTACCCTTCGTAATATTACACAAATCTAGAGACGTATGGGTACAATTCGTTACACAGACGCTTGATCCGTTATTCACTAAAGGTGCATGCCTCCCGGTTACATTACATAACCATCCTTTATCCCAATGGCTCCATCTAGTAGTCATCGGATCTTTTTTGTGTTCAATGGTTACATTGTCACCAC---------------TTTTAACCTCTACTTTATTGAAACCCGCACCTTCATGTATAAACACCGTCATGAAACACGCTATAAGTACCCCCCCCCC-----ACAATGGAATGCTGCCAAACCGGTTATTTCCCGTTATAGCCAT

HAN2 CTAATTTTTAGCAGTCAAATACCATAATTCACCGCTGTACTCGTCTCTATTCTCTCCAAACCAACGACCAAGATCGACAACGCCGTCATTACCCTTCGTAATATTACACAAATCTAGAGACATATGGGTACAATTCGTTACACAGACGCTTGATGCGTTATTCACTAAAGGTGCATGCCTCCCGGTTACATTACATAACCATCCTTTATCCCAATGGCTCCATCTAGTAGTCATCGGATCTTTTTTGTGTTCAATGGTTACATTGTCACCAC---------------TTTTAACCTCTACTTTATTGAAACCCGCACCTTCATGTATAAACACCGTCATGAAACACGCTATAAGTACCCCCCCCCC-----ACAATGGAATGCTGCCAAACCGGTTATTTCCCGTTATAGCCAT

2CEN2 CTAATTTTTAGCAGTCAAATACCATAATTCACCGCTGTACTCGTCTCTATTCTCTCCAAACCAACGACCAAGATCGACAACGCCGTCATTACCCTTCGTAATATTACACAAATCTAGAGACGTATGGGTACAATTCGTTACACAGACGCTTGATGCGTTATTCACTAAAGGTGCATGCCTCCCGGTTACATTACATAACCATCCTTTATCCCAATGGCTCCATCTAGTAGTCATCGGATCTTTTTTGTGTTCAATGGTTACATTGTCACCAC---------------TTTTAACCTCTACTTTATTGAAACCCGCACCTTCATGTATAAACACCGTCATGAAACACGCTATAAGTACCCCCCCCCC-----ACAATGGAATGCTGCCAAACCGGTTATTTCCCGTTATAGCCAT

CZ/1/2011 CTAATTTTTAGCAGTCAAATACCATAATTCACCGCTGTACTCGTCTCTATTCTCTCCAAACCAACGACCAAGATCGACAACGCCGTCATTACCCTTCGTAATATTACACAAATCTAGAGACGTATGGGTACAATTCGTTACACAGACGCTTGATGCGTTATTCACTAAAGGTGCATGCCTCCCGGTTACATTACATAACCATCCTTTATCCCAATGGCTCCATCTAGTAGTCATCGGATCTTTTTTGTGTTCAATGGTTACATTGTCACCAC---------------TTTTAACCTCTACTTTATTGAAACCCGCACCTTCATGTATAAACACCGTCATGAAACACGCTATAAGTACCCCCCCCCC-----ACAATGGAATGCTGCCAAACCGGTTATTTCCCGTTATAGCCAT

PRA5 CTAATTTTTAGCAGTCAAATACCATAATTCACCGCTGTACTCGTCTCTATTCTCTCCAAACCAACGACCAAGATCGACAACGCCGTCATTACCCTTCGTAATATTACACAAATCTAGAGACGTATGGGTACAATTCGTTACACAGACGCTTGATGCGTTATTCACTAAAGGTGCATGCCTCCCGGTTACATTACATAACCATCCTTTATCCCAATGGCTCCATCTAGTAGTCATCGGATCTTTTTTGTGTTCAATGGTTACATTGTCACCAC---------------TTTTAACCTCTACTTTATTGAAACCCGCACCTTCATGTATAAACACCGTCATGAAACACGCTATAAGTACCCCCCCCCC-----ACAATGGAATGCTGCCAAACCGGTTATTTCCCGTTATAGCCAT

HANSCTR8 CTAATTTTTAGCAGTCAAATACCATAATTCACCGCTGTACTCGTCTCTATTCTCTCCAAACCAACGACCAAGATCGACAACGCCGTCATTACCCTTCGTAATATTACACAAATCTAGAGACGTATGGGTACAATTCGTTACACAGACGCTTGATGCGTTATTCACTAAAGGTGCATGCCTCCCGGTTACATTACATAACCATCCTTTATCCCAATGGCTCCATCTAGTAGTCATCGGATCTTTTTTGTGTTCAATGGTTACATTGTCACCAC---------------TTTTAACCTCTACTTTATTGAAACCCGCACCTTCATGTATAAACACCGTCATGAAACACGCTATAAGTACCCCCCCCCC-----ACAATGGAATGCTGCCAAACCGGTTATTTCCCGTTATAGCCAT

BE/16/2010 CTAATTTTTAGCAGTCAGATACCATAATTCACCGCTGTACTCGTCTCTATTCTCTCCAAACCAACGACCAAGATCGACAACGCCGTCATTACCCTTCGTAATATTACACAAATCTAGAGACGTATGGGTACAATTCGTTACACAGACGCTTGATGCGTTATTCACTAAAGGTGCATGCCTCCCGGTTACATTACATAACCATCCTTTATCCCAATGGCTCCATCTAGTAGTCATCGGATCTTTTTTGTGTTCAATGGTTACATTGTCACCAC---------------TTTTAACCTCTACTTTATTGAAACCCGCACCTTCATGTATAAACACCGTCATGAAACACGCTATAAGTACCCCCCCCCC-----ACAATGGAATGCTGCCAAACCGGTTATTTCCCGTTATAGCCAT

HANRTR9 CTAATTTTTAGCAGTCAGATACCATAATTCACCGCTGTACTCGTCTCTATTCTCTCCAAACCAACGACCAAGATCGACAACGCCGTCATTACCCTTCGTAATATTACACAAATCTAGAGACGTATGGGTACAATTCGTTACACAGACGCTTGATGCGTTATTCACTAAAGGTGCATGCCTCCCGGTTACATTACATAACCATCCTTTATCCCAATGGCTCCATCTAGTAGTCATCGGATCTTTTTTGTGTTCAATGGTTACATTGTCACCAC---------------TTTTAACCTCTACTTTATTGAAACCCGCACCTTCATGTATAAACACCGTCATGAAACACGCTATAAGTACCCCCCCCCC-----ACAATGGAATGCTGCCAAACCGGTTATTTCCCGTTATAGCCAT

BE/9/2010 G3 TTACTTAGTTACAATAAGAAACCAAGTATCCACTTGTTCAGGACCGTTATCAACTCTCCGCTTTAAATCATAAGATCCTTCGTTGCCTTGCGTTACATTGCAAAACGTCAATCCTGTATGACTACAATTACACACACCGTGAATCGGCGGCGTTGCTAGCGTGTAATTCTTCGTCCCCTCCACACTACACACGTCGGTATCATTTTTACCATCAAAAAACGACCAAATCGTCTTCAGGTTAGCACTAACGGTTTTATCATCACCGG---------------------TCTTTACATGAACATTAGCCATTCCCTCGACTCTGTAATTCAACGTCAAAATTATACACGCTATAAGTACCCCCCCCC------ACAATGGAATGCTGCCAAACTGGTTCTTTCCCGTTATAGCCAT

BE/41/2011 TTACTTAGTTACAATAAGGAACCAAGTATCCACTTGTTCAGGACCGTTATCAACTCTCCGCTTTAAATCATAAGATCCTTCGTTGCCTTGCGTTACGTTGCAAAACGTCAATCCTGTATGACTACAATTACACACACCGTGAATCGGCGGCGTTGCTAGCGTGTAATTCTTCGTCCCCTCCACACTACACACGTCGGTATCATTTTTACCATCAAAAAACGACCAAATCGTTTTCAGGTTAGCACTAACAGTTTTATCATCACCGG---------------------TCTTTACATGAACATTAGCCATTCCCTCGACTCTGTAATTCAACGTCAAAATTATACACGCTATAAGTACCCCCCCCC------ACAATGGAATGCTGCCAAACTGGTTCTTTCCCGTTATAGCCAT

NL/Rot1 TTACTTAGTTACAATAAGAAACCAAGTATCCACTTGTTCAGGACCGTTATCAACTCTCCGCTTTAAATCATAAGATCCTTCGTTGCCTTGCGTTACGTTGCAAAACGTCAATCCTGTATGACTACAATTACACACACCGTGAATCGGCGGCGTTGCTAGCGTGTAATTCTTCGTCCCCTCCACACTACACACGTCGGTATCATTTTTACCATCAAAAAACGACCAAACCGTTTTCAGGTTAGCACTAACGGTTTTATCATCACCGG---------------------TCTTTACATGAACATTAGCCATTCCCTCGACTCTGTAATTCAACGTCAAAATTATACACGCTATAAGTACCCCCCCCC------ACAATGGAATGCTGCCAAACTGGTTCTTTCCCGTTATAGCCAT

UK/Lon7 TTACTTAGTTACAATAAGAAACCAGGTATCCACTTGTTCAGGACCGTTATCAACTCTCCGCTTTAAATCATAAGATCCTTCGTTGCCTTGCGTTACGTTGCAAAACGTCAATCCTGTATGACTACAATTACACACACCGTGAATCGGCGGCGTTGCTAGCGTGTAATTCTTCGTCCCCTCCACACTACATACGTCGGTATCATTTTTACCGTCAAAAAACGACCAAATCGTTTTCAGGTTAGCACTAACGGTTTTATCATCACCGG---------------------TCTTTACATGAACATTAGCCATTCCCTCGACTCCGTAATTCAACGTCAAAATTATACACGCTATAAGTACCCCCCCCC------ACAATGGAATGCTGCCAAACTGGTTCTTTCCCGTTATAGCCAT

HANSCTR1B TTACTTAGTTACAATAAGAAACCAAGTATCCACTTGTTCAGGACCGTTATCAACTCTCCGCTTTAAATCATAAGATCCTTCGTTGCCTTGCGTTACGTTGCAAAACGTCAATCCTGTATGACTACAATTACACACACCGTGAATCGGCGGCGTTGCTAGCGTGTAATTCTTCGTCCCCTCCACACTACATACGTCGGTATCATTTTTACCGTCAAAAAACGACCAAATCGTTTTCAGGCTAGCACTAACGGTTCTATCATCACCGG---------------------TCTTTACACGAACATTAGCCATTCCCTCGACTCCGTAATTCAACGTCAAAATTATACACGCTATAAGTACCCCCCCCC------ACAATGGAATGCTGCCAAACTGGTTCTTTCCCGTTATAGCCAT

PAV7 TTACTTAGTTACAATAAGAAACCAAGTATCCACTTGTTCAGGACCGTTATCAACTCTCCGCTTTAAATCATAAGATCCTTCGTTGCCTTGCGTTATGTTGCAAAACGTCAATCCTGTATGACTACAATTACACACACCGTGAATCGGCGGCGTTGCTAGCGTGTAATTCTTCGTCCCCTCCACACTACACACGTCGGTATCATTTTTGCCATCAAAAAACGACCAAATCGTTTTCAGGTTAGCACTAACGGTTTTATCATCACCGG---------------------TCTTTACACGAACATTAGCCATTCCCTCGACTCTGTAATTCAACGTCAAAATTACACACGCTATAAGTACCCCCCCCCC-----ACAATGGAATGCTGCCAAACTGGTTCTTTCCCGTTATAGCCAT

Pat_E TTACTTAGTTACAATAAGAAACCAAGTATCCACTTGTTCAGGACCGTTATCAACTCTCCGCTTTAAATCATAAGATCCTTCGTTGCCTTGCGTTATGTTGCAAAACGTCAATCCTGTATGACTACAATTACACACACCGTGAATCGGCGGCGTTGCTAGCGTGTAATTCTTCGTCCCCTCCACACTACACACGTCGATATCATTTTTGCCATCAAAAAACGACCAAATCGTTTTCAGGTTAGCACTAACGGTTTTATCATCACCGG---------------------TCTTTACACGAACATTAGCCATTCCCTCGACTCTGTAATTCAACGTCAAAATTACACACGCTATAAGTACCCCCCCCCC-----ACAATGGAATGCTGCCAAACTGGTTCTTTCCCGTTATAGCCAT

BE/10/2011 TTACTTAGTTACAATAAGAAACCAAGTATCCACTTGTTCAGGACCGTTATCAACTCTCCGCTTTAAATCATAAGATCCTTCGTTGCCTTGCGTTACATTGCAAAACGTCAATCCTGTATGACTACAATTACACACACCGTGAATCGGCGGCGTTGCTAGCGTGTAATTCTTCGTCCCCTCCACACTACACACGTCGGTATCATTTTTACCATCAAAAAACGACCAAATCGTCTTCAGGTTAGCACTAACGGTTTTATCATCACCGG---------------------TCTTTACATGAACATTAGCCATTCCCTCGACTCTGTAATTCAACGTCAAAATTATACACGCTATAAGTACCCCCCCCCC-----ACAATGGAATGCTGCCAAACTGGTTCTTTCCCGTTATAGCCAT

BE/20/2011 TTACTTAGTTACAATAAGAAACCAAGTATCCACTTGTTCAGGACCGTTATCAACTCTCCGCTTTAAATCATAAGATCCTTCGTTGCCTTGCGTTACATTGCAAAACGTCAATCCTGTATGACTACAATTACACACACCGTGAATCGGCGGCGTTGCTAGCGTGTAATTCTTCGTCCCCTCCACACTACACACGTCGGTATCATTTTTACCATCAAAAAACGACCAAATCGTCTTCAGGTTAGCACTAACGGTTTTATCATCACCGG---------------------TCTTTACATGAACATTAGCCATTCCCTCGACTCTGTAATTCAACGTCAAAATTATACACGCTATAAGTACCCCCCCCCC-----ACAATGGAATGCTGCCAAACTGGTTCTTTCCCGTTATAGCCAT

BE/10/2012 TTACTTAGTTACAATAAGAAACCAAGTATCCACTTGTTCAGGACCGTTATCAACTCTCCGCTTTAAATCATAAGATCCTTCGTTGCCTTGCGTTACATTGCAAAACGTCAATCCTGTATGACTACAATTACACACACCGTGAATCGGCGGCGTTGCTAGCGTGTAATTCTTCGTCCCCTCCACACTACACACGTCGGTATCATTTTTACCATCAAAAAACGACCAAATCGTCTTCAGGTTAGCACTAACGGTTTTATCATCACCGG---------------------TCTTTACATGAACATTAGCCATTCCCTCGACTCTGTAATTCAACGTCAAAATTATACACGCTATAAGTACCCCCCCCCC-----ACAATGGAATGCTGCCAAACTGGTTCTTTCCCGTTATAGCCAT

UK/Lon9 TTACTTAGTTACAATAAGAAACCAAGTATCCACTTGTTCAGGACCGTTATCAACTCTCCGCTTTAAATCATAAGATCCTTCGTTGCCTTGCGTTACATTGCAAAACGTCAATCCTGTATGACTACAATTACACACACCGTGAATCGGCGGCGTTGCTAGCGTGTAATTCTTCGTCCCCTCCACACTACACACGTCGGTATCATTTTTACCATCAAAAAACGACCAAATCGTCTTCAGGTTAGCACTAACGGTTTTATCATCACCGG---------------------TCTTTACATGAACATTAGCCATTCCCTCGACTCTGTAATTCAACGTCAAAATTATACACGCTATAAGTACCCCCCCCCC-----ACAATGGAATGCTGCCAAACTGGTTCTTTCCCGTTATAGCCAT

BE/26/2011 TTACTTAGTTACAATAAGAAACCAAGTATCCACTTGTTCAGGACCGTTATCAACTCTCCGCTTTAAATCATAAGATCCTTCGTTGCCTTGCGTTACATTGCAAAACGTCAATCCTGTATGACTACAATTACACACACCGTGAATCGGCGGCGTTGCTAGCGTGTAATTCTTCGTCCCCTCCACACTACACACGTCGGTATCATTTTTACCATCAAAAAACAACCAAATCGTCTTCAGGTTAGCACTAACGGTTTTATCATCACCGG---------------------TCTTTACATGAACATTAGCCATTCCCTCGACTCTGTAATTCAACGTCAAAATTATACACGCTATAAGTACCCCCCCCCC-----ACAATGGAATGCTGCCAAACTGGTTCTTTCCCGTTATAGCCAT

HANSCTR12 TTACTTAGTTACAATAAGAAACCAAGTATCCACTTGTTCAGGACCGTTATCAACTCTCCGCTTTAAATCATAAGATCCTTCGTTGCCTTGCGTTACGTTGCAAAACGTCAATCCTGTATGACTACAATTACACACACCGTGAATCGGCGGCGTTGCTAGCGTGTAATTCTTCGTCCCCTCCACACTACACACGTCGGTATAATTTTTACCATCAAAAAACGACCAAATCGTTTTCAGGTTAGCACTAACGGTTTTATCATCACCGG---------------------TCTTTACATGAACATTAGCCATTCCCTCGACTCTGTAATTCAACGTCAAAATTATACACGCTATAAGTACCCCCCCCCC-----ACAATGGAATGCTGCCAAACTGGTTCTTTCCCGTTATAGCCAT

HAN TTACTTAGTTACAATAAGAAACCAAGTATCCACTTGTTCAGGACCGTTATCAACTCTCCGCTTTAAATCATAAGATCCTTCGTTGCCTTGCGTTACGTTGCAAAACGTCAATCCTGTATGACTACAATTACACACACCGTGAATCGGCGGCGTTGCTAGCGTGTAATTATTCGTCCCCTCCACACTACACACGTCGGTATCATTTTTACCATCAAAAAACGACCAAATCGTTTTCAGGTTAGCACTAACGGTTTTATCATCACCGG---------------------TCTTTACATGAACATTAGCCATTCCCTCGACTCTGTAATTCAACGTCAAAATTATACACGCTATAAGTACCCCCCCCCC-----ACAATGGAATGCTGCCAAACTGGTTCTTTCCCGTTATAGCCAT

PH TTACTTAGTTACAATAAGAAACCAAGTATCCACTTGTTCAGGACCGTTATCAACTCTCCGCTTTAAATCATAAGATCCTTCGTTGCCTTGCGTTACGTTGCAAAACGTCAATCCTGTATGACTACAATTACACACACCGTGAATCGGCGGCGTTGCTAGCGTGTAATTCTTCGTCCCCTCCACACTACACACGTCGGTATCATTTTTACCATCAAAAAACGACCAAACCGTTTTCAGGTTAGCACTAACGGTTTTATCATCACCGG---------------------TCTTTACATGAACATTAGCCATTCCCTCGACTCTGTAATTCAACGTCAAAATTATACACGCTATAAGTACCCCCCCCCC-----ACAATGGAATGCTGCCAAACTGGTTCTTTCCCGTTATAGCCAT

HAN11 TTACTTAGTTACAATAAGAAACCAAGTATCCACTTGTTCAGGACCGTTATCAACTCTCCGCTTTAAATCATAAGATCCTTCGTTGCCTTGCGTTACGTTGCAAAACGTCAATCCTGTATGACTACAATTACACACACCGTGAATCGGCGGCGTTGCTAGCGTGTAATTCTTCGTCCCCTCCACACTACACACGTCGGTATCATTTTTACCATCAAAAAACGACCAAATCGTTTTCAGGTTAGCACTAACGGTTTTATCATCACCGG---------------------TCTTTACATGAACATTAGCCATTCCCTCGACTCTGTAATTGAACGTCAAAATTATACACGCTATAAGTACCCCCCCCCC-----ACAATGGAATGCTGCCAAACTGGTTCTTTCCCGTTATAGCCAT

JHC TTACTTAGTTACAATAAGAAACCAAGTATCCACTTGTTCAGGACCGTTATCAACTCTCCGCTTTAAATCATAAGATCCTTCGTTGCCTTGCGTTACGTTGCAAAACGTCAATCCTGTATGACTACAATTACACACACCGTGAATCGGCGGCGTTGCTAGCGTGTAATTCTTCGTCCCCTCCACACTACACACGTCGGTATCATTTTTACCATCAAAAAACGACCAAATCGTTTTCAGGTTAGCACTAACGGTTTTATCATCACCGG---------------------TCTTTACATGAACATTAGCCATTCCCTCGACTCTGTAATTCAACGTCAAAATTATACACGCTATAAGTACCCCCCCCCC-----ACAATGGAATGCTGCCAAACTGGTTCTTTCCCGTTATAGCCAT

BE/6/2010 TTACTTAGTTACAATAAGAAACCAAGTATCCACTTGTTCAGGACCGTTATCAACTCTCCGCTTTAAATCATAAGATCCTTCGTTGCCTTGCGTTACGTTGCAAAACGTCAATCCTGTATGACTACAATTACACACACCGTGAATCGGCGGCGTTGCTAGCGTGTAATTCTTCGTCCCCTCCACACTACATACGTCGGTATCATTTTTACCGTCAAAAAACGACCAAATCGTTTTCAGGTTAGCACTAACGGTTTTATCATCACCGG---------------------TCTTTACACGAACATTAGCCATTCCCTCGACTCCGTAATTCAACGTCAAAATTATACACGCTATAAGTACCCCCCCCCCCC---ACAATGGAATGCTGCCAAACTGGTTCTTTCCCGTTATAGCCAT

BE/11/2010 TTACTTAGTTACAATAAGAAACCAGGTATCCACTTGTTCAGGACCGTTATCAACTCTCCGCTTTAAATCATAAGATCCTTCGTTGCCTTGCGTTACGTTGCAAAACGTCAATCCTGTATGACTACAATTACACACACCGTGAATCGGCGGCGTTGCTAGCGTGTAATTCTTCGTCCCCTCCACACTACATACGTCGGTATCATTTTTACCGTCAAAAAACGACCAAATCGTTTTCAGGTTAGCACTAACGGTTTTATCATCACCGG---------------------TCTTTACATGAACATTAGCCATTCCCTCGACTCCGTAATTCAACGTCAAAATTATACACGCTATAAGTACCCCCCCCCCC----ACAATGGAATGCTGCCAAACTGGTTCTTTCCCGTTATAGCCAT

BE/20/2010 ----TTAGTTACAATAAGAAACCAAGTATCCACTTGTTCAGGACCGTTATCAACTCTCCGCTTTAAATCATAAGATCCTTCGTTGCCTTGCGTTACGTTGCAAAACGTCAATCCTGTATGACTACAATTACACACACCGTGAATCGGCGGCGTTGCTAGCGTGTAATTCTTCGTCCCCTCCACACTACATACGTCGGTATCATTTTTACCGTCAAAAAACGACCAAATCGTTTTCAGGTTAGCACTAACGGTTTTATCATCACCGG---------------------TCTTTACACGAACATTAGCCATTCCCTCGACTCCGTAATTCAACGTCAAAAGTATACACGCTATAAGTACCCCCCCCCCC----ACAATGGAATGCTGCCAAACTGGTTCTTTCCCGTTATAGCCAT

BE/28/2010 TTACTTAGTTACAATAAGAAACCAAGTATCCACTTGTTCAGGACCGTTATCAACTCTCCGCTTTAAATCATAAGATCCTTCGTTGCCTTGCGTTACGTTGCAAAACGTCAATCCTGTATGACTACAATTACACACACCGTGAATCGGCGGCGTTGCTAGCGTGTAATTCTTCGTCCCCTCCACACTACATACGTCGGTATCATTTTTACCGTCAAAAAACGACCAAATCGTTTTCAGGTTAGCACTAACGGTTTTATCATCACCGG---------------------TCTTTACACGAACATTAGCCATTCCCTCGACTCCGTAATTCAACGTCAAAATTATACACGCTATGAGTACCCCCCCCCCC----ACAATGGAATGCTGCCAAACTGGTTCTTTCCCGTTATAGCCAT

BE/12/2010 TTACTTAGTTACAATAAGAAACCAAGTATCCACTTGTTCAGGACCGTTATCAACTCTCCGCTTTAAATCATAAGATCCTTCGTTGCCTTGCGTTACGTTGCAAAACGTCAATCCTGTATGACTACAATTACACACACCGTGAATCGGCGGCGTTGCTAGCGTGTAATTCTTCGTCCCCTCCACACTACATACGTCGGTATCATTTTTACCGTCAAAAAACGACCAAATCGTTTTCAGGTTAGCACTAACGGTTTTATCATCACCGG---------------------TCTTTTCACGAACATTAGCCATTCCCTCGACTCCGTAATTCAACGTCAAAATTATACACGCTATAAGTACCCCCCCCCCC----ACAATGGAATGCTGCCAAACTGGTTCTTTCCCGTTATAGCCAT

PAV20 TTACTTAGTTACAATAAGAAACCAAGTATCCACTTGTTCAGGACCGTTATCAACTCTCCGCTTTAAATCATAAGATCCTTCGTTGCCTTGCGTTACGTTGCAAAACGTCAATCCTGTATGACTACAATTACACACACCGTGAATCGGCGGCGTTGCTAGCGTGTAATTCTTCGTCCCCTCCACACTACATACGTCGGTATCATTTTTACCGTCAAAAAACGACCAAATCGTTTTCAGGTTAGCACTAACGGTTTTATCATCACCGG---------------------TCTTTACACGAACATTAGCCATTCCCTCGACTCCGTAATTCAACGTCAAAATTATACACGCTATAAGTACCCCCCCCCCC----ACAATGGAATGCTGCCAAACTGGTTCTTTCCCGTTATAGCCAT

BE/2/2013 TTACTTAGTTACAATAAGAAACCAAGTATCCACTTGTTCAGGACCGTTATCAACTCTCCGCTTTAAATCATAAGATCCTTCGTTGCCTTGCGTTACGTTGCAAAACGTCAATCCTGTATGACTACAATTACACACACCGTGAATCGGCGGCGTTGCTAGCGTGTAATTCTTCGTCCCCTCCACACTACATACGTCGGTATCATTTTTACCGTCAAAAAACGACCAAATCGTTTTCAGGTTAGCACTAACGGTTTTATCATCACCGG---------------------TCTTTACACGAACATTAGCCATTCCCTCGACTCCGTAATTCAACGTCAAAATTATACACGCTATAAGTACCCCCCCCCCC----ACAATGGAATGCTGCCAAACTGGTTCTTTCCCGTTATAGCCAT

HANChild1 TTACTTAGTTACAATAAGAAACCAAGTATCCACTTGTTCAGGACCGTTATCAACTCTCCGCTTTAAATCATAAGATCCTTCGTTGCCTTGCGTTACGTTGCAAAACGTCAATCCTGTATGACTACAATTACACACACCGTGAATCGGCGGCGTTGCTAGCGTGTAATTCTTCGTCCCCTCCACACTACATACGTCGGTATCATTTTTACCGTCAAAAAACGACCAAATCGTTTTCAGGTTAGCACTAACGGTTTTATCATCACCGG---------------------TCTTTACACGAACATTAGCCATTCCCTCGACTCCGTAATTCAACGTCAAAATTATACACGCTATAAGTACCCCCCCCCCC----ACAATGGAATGCTGCCAAACTGGTTCTTTCCCGTTATAGCCAT

BE/22/2010 TTACTTAGTTACAATAAGAAACCAAGTATCCACTTGTTCAGGACCGTTATCAACTCTCCGCTTTAAATCATAAGATCCTTCGTTGCCTTGCGTTATGTTGCAAAACGTCAATCCTGTATGACTACAATTACACACACCGTGAATCGGCGGCGTTGCTAGCGTGTAATTCTTCGTCCCCTCCACACTACACACGTCGGTATCATTTTTGCCATCAAAAAACGACCAAATCGTTTTCAGGTTAGCACTAACGGTTTTATCATCACCGG---------------------TCTTTACACGAACATTAGCCATTCCCTCGACTCTGTAATTCAACGTCAAAATTACACACGCTATAAGTACCCCCCCCCCC----ACAATGGAATGCTGCCAAACTGGTTCTTTCCCGTTATAGCCAT

HANRTR6 TTACTTAGTTACAATAAGAAACCAAGTATCCACTTGTTCAGGACCGTTATCAACTCTCCGCTTTAAATCATAAGATCCTTCGTTGCCTTGCGTTATGTTGCAAAACGTCAATCCTGTATGACTACAATTACACACACCGTGAATCGGCGGCGTTGCTAGCGTGTAATTCTTCGTCCCCTCCACACTACACACGTCGGTATCATTTTTGCCATCAAAAAACGACCAAATCGTTTTCAGGTTAGCACTAACGGTTTTATCATCACCGG---------------------TCTTTACACGAACATTAGCCATTCCCTCGACTCTGTAATTCAACGTCAAAATTACACACGCTATAAGTACCCCCCCCCCC----ACAATGGAATGCTGCCAAACTGGTTCTTTCCCGTTATAGCCAT

UK/Lon2 TTACTTAGTTACAATAAGAAACCAAGTATCCACTTGTTCAGGACCGTTATCAACTCTCCGCTTTAAATCATAAGATCCTTCGTTGCCTTGCGTTATGTTGCAAAACGTCAATCCTGTATGACTACAATTACACACACCGTGAATCGGCGGCGTTGCTAGCGTGTAATTCTTCGTCCCCTCCACACTACACACGTCGATATCATTTTTGCCATCAAAAAACGACCAAATCGTTTTCAGGTTAGCACTAACGGTTTTATCATCACCGG---------------------TCTTTACACGAACATTAGCCATTCCCTCGACTCTGTAATTCAACGTCAAAATTACACACGCTATAAGTACCCCCCCCCCC----ACAATGGAATGCTGCCAAACTGGTTCTTTCCCGTTATAGCCAT

CINCY TTACTTAGTTACAATAAGAAACCAAGTATCCACTTGTTCAGGACCGTTATCAACTCTCCGCTTTAAATCATAAGATCCTTCGTTGCCTTGCGTTACATTGCAAAACGTCAATCCTGTATGACTACAATTACACACACCGTGAATCGGCGGCGTTGCTAGCGTGTAATTCTTCGTCCCCTCCACACTACACACGTCGGTATCATTTTTACCATCAAAAAACGACCAAATCGTCTTCAGGTTAGCACTAACGGTTTTATCATCACCGG---------------------TCTTTACATGAACATTAGCCATTCCCTCGACTCTGTAATTCAACGTCAAAATTATACACGCTATAAGTACCCCCCCCCCC----ACAATGGAATGCTGCCAAACTGGTTCTTTCCCGTTATAGCCAT

3157 TTACTTAGTTACAATAAGAAACCAAGTATCCACTTGTTCAGGACCGTTATCAACTCTCCGCTTTAAATCATAAGATCCTTCGTTGCCTTGCGTTACGTTGCAAAACGTCAATCCTGTATGACTACAATTACACACACCGTGAATCGGCGGCGTTGCTAGCGTGTAATTCTTCGTCCCCTCCACACTACACACGTCGGTATCATTTTTACCATCAAAAAACGACCAAACCGTTTTCAGGTTAGCACTAACGGTTTTATCATCACCGG---------------------TCTTTACATGAACATTAGCCATTCCCTCGACTCTGTAATTCAACGTCAAAATTATACACGCTATAAGTACCCCCCCCCCC----ACAATGGAATGCTGCCAAACTGGTTCTTTCCCGTTATAGCCAT

HANSCTR13 TTACTTAGTTACAATAAGAAACCAAGTATCCACTTGTTCAGGACCGTTATCAACTCTCCGCTTTAAATCATAAGATCCTTCGTTGCCTTGCGTTACGTTGCAAAACGTCAATCCTGTATGACTACAATTACACACACCGTGAATCGGCGGCGTTGCTAGCGTGTAATTCTTCGTCCCCTCCACACTACACACGTCGGTATCATTTTTACCATCAAAAAACGACCAAATCGTTTTCAGGTTAGCACTAACGGTTTTATCATCACCGG---------------------TCTTTACATGAACATTAGCCATTCCCTCGACTCTGTAATTCAACGTCAAAATTATACACGCTATAAGTACCCCCCCCCCC----ACAATGGAATGCTGCCAAACTGGTTCTTTCCCGTTATAGCCAT

HANChild2&3 TTACTTAGTTACAATAAGAAACCAAGTATCCACTTGTTCAGGACCGTTATCAACTCTCCGCTTTAAATCATAAGATCCTTCGTTGCCTTGCGTTACGTTGCAAAACGTCAATCCTGTATGACTACAATTACACACACCGTGAATCGGCGGCGTTGCTAGCGTGTAATTCTTCGTCCCCTCCACGCTACATACGTCGGTATCATTTTTACCGTCAAAAAACGACCAAATCGTTTTCAGGTTAGCACTAACGGTTTTATCATCACCGG---------------------TCTTTACACGAACATTAGCCATTCCCTCGACTCCGTAATTCAACGTCAAAATTATACACGCTATAAGTACCCCCCCC-------ACAATGGAATGCTGCCAAACTGGTTCTTTCCCGTTATAGCCAT

BE/17/2010 TTACTTAGTTACAATAAGAAACCAGGTATCCACTTGTTCAGGACCGTTATCAACTCTCCGCTTTAAATCATAAGATCCTTCGTTGCCTTGCGTTACGTTGCAAAACGTCAATCCTGTATGACTACAATTACACACACCGTGAATCGGCGGCGTTGCTAGTGTGTAATTCTTCGTCCCCTCCACACTACATACGTCGGTATCATTTTTACCGTCGAAAAACGACCAAATCGTTTTCAGGTTAGCACTAACGGTTTTATCATCACCGG---------------------TCTTTACACGAACATTAGCCATTCCCTCGACTCCGTAATTCAACGTCAAAATTATACACGCTATAAGTACCCCCCCCCC-----ACAATGGAATGCTGCCAAACTGGTTCTTTCCCGTTATAGCCAT

UKNEQAS1 TTACTTAGTTACAATAAGAAACCAGGTATCCACTTGTTCAGGACCGTTATCAACTCTCCGCTTTAAATCATAAGATCCTTCGTTGCCTTGCGTTACGTTGCAAAACGTCAATCCTGTATGACTACAATTACACACACCGTGAATCGGCGGCGTTGCTAGCGTGTAATTCTTCGTCCCCTCCACACTACATACGTCGGTATCATTTTTACCGTCAAAAAACGACCAAATCGTTTTCAGGTTAGCACTAACGGTTTTATCATCACCGG---------------------TCTTTACATGAACATTAGCCATTCCCTCGACTCCGTAATTCAACGTCAAAATTATACACGCTATAAGTACCCCCCCCCC-----ACAATGGAATGCTGCCAAACTGGTTCTTTCCCGTTATAGCCAT

BE/42/2011 TTACTTAGTTACAATAAGAAACCAAGTATCCACTTGTTCAGGACCGTTATCAACTCTCCGCTTTAAATCATAAGATCCTTCGTTGCCTTGCGTTACGTTGCAAAACGTCAATCCTGTATGACTACAATTACACACACCGTGAATCGGCGGCGTTGCTAGCGTGTAATTCTTCGTCCCCTCCACACTACATACGTCGGTATCATTTTTACCGTCAAAAAACGACCAAATCGTTTTCAGGCTAGCACTAACGGTTCTATCATCACCGG---------------------TCTTTACACGAACATTAGCCATTCCCTCGACTCCGTAATTCAACGTCAAAATTATACACGCTATAAGTACCCCCCCCCC-----ACAATGGAATGCTGCCAAACTGGTTCTTTCCCGTTATAGCCAT

BE/13/2011 TTACTTAGTTACAATAAGAAACCAAGTATCCACTTGTTCAGGACCGTTATCAACTCTCCGCTTTAAATCATAAGATCCTTCGTTGCCTTGCGTTACGTTGCAAAACGTCAATCCTGTATGACTACAATTACACACACCGTGAATCGGCGGCGTTGCTAGCGTGTAATTCTTCGTCCCCTCCACACTACATACGTCGGTATCATTTTTACCGTCAAAAAACGACCAAATCGTTTTCAGGTTAGCACTAACGGTTTTATCATCACCGG---------------------TCTTTACACGAACATTAGCCATTCCCTCGACTCCGTAATTCAACGTCAAAATTATACACGCTATAAGTACCCCCCCCCC-----ACAATGGAATGCTGCCAAACTGGTTCTTTCCCGTTATAGCCAT

BE/27/2010 TTACTTAGTTACAATAAGAAACCAAGTATCCACTTGTTCAGGACCGTTATCAACTCTCCGCTTTAAATCATAAGATCCTTCGTTGCCTTGCGTTACGTTGCAAAACGTCAATCCTGTATGACTACAATTACACACACCGTGAATCGGCGGCGTTGCTAGCGTGTAATTCTTCGTCCCCTCCACACTACATACGTCGGTATCATTTTTACCGTCAAAAAACGACCAAATCGTTTTCAGGTTAGCACTAACGGTTTTATCATCACCGG---------------------TCTTTACACGAACATTAGCCATTCCCTCGACTCCGTAATTCAACGTCAAAATTATACACGCTATAAGTACCCCCCCCCC-----ACAATGGAATGCTGCCAAACTGGTTCTTTCCCGTTATAGCCAT

2CEN5 TTACTTAGTTACAATAAGAAACCAAGTATCCACTTGTTCAGGACCGTTATCAACTCTCCGCTTTAAATCATAAGATCCTTCGTTGCCTTGCGTTACGTTGCAAAACGTCAATCCTGTATGACTACAATTACACACACCGTGAATCGGCGGCGTTGCTAGCGTGTAATTCTTCGTCCCCTCCACACTACATACGTCGGTATCATTTTTACCGTCAAAAAACGACCAAATCGTTTTCAGGTTAGCACTAACGGTTTTATCATCACCGG---------------------TCTTTACACGAACATTAGCCATTCCCTCGACTCCGTAATTCAACGTCAAAATTATACACGCTATAAGTACCCCCCCCCC-----ACAATGGAATGCTGCCAAACTGGTTCTTTCCCGTTATAGCCAT

BE/3/2010 TTACTTAGTTACAATAAGAAACCAAGTATCCACTTGTTCAGGACCGTTATCAACTCTCCGCTTTAAATCATAAGATCCTTCGTTGCCTTGCGTTACGTTGCAAAACGTCAATCCTGTATGACTACAATTACACACACCGTGAATCGGCGGCGTTGCTAGCGTGTAATTCTTCGTCCCCTCCACACTACATACGTCGGTATCATTTTTACCGTCAAAAAACGACCAAATCGTTTTCAGGTTAGCACTAACGGTTTTATCATCACCGG---------------------TCTTTACACGAACATTAGCCATTCCCTCGACTCCGTAATTCAACGTCAAAATTATACACGCTATAAGTACCCCCCCCCC-----ACAATGGAATGCTGCCAAACTGGTTCTTTCCCGTTATAGCCAT

HAN29 TTACTTAGTTACAATAAGAAACCAAGTATCCACTTGTTCAGGACCGTTATCAACTCTCCGCTTTAAATCATAAGATCCTTCGTTGCCTTGCGTTACGTTGCAAAACGTCAATCCTGTATGACTACAATTACACACACCGTGAATCGGCGGCGTTGCTAGCGTGTAATTCTTCGTCCCCTCCACACTACATACGTCGGTATCATTTTTACCGTCAAAAAACGACCAAATCGTTTTCAGGTTAGCACTAACGGTTTTATCATCACCGG---------------------TCTTTACACGAACATTAGCCATTCCCTCGACTCCGTAATTCAACGTCAAAATTATACACGCTATAAGTACCCCCCCCCC-----ACAATGGAATGCTGCCAAACTGGTTCTTTCCCGTTATAGCCAT

BE/2/2012 TTACTTAGTTACAATAAGAAACCAAGTATCCACTTGTTCAGGACCGTTATCAACTCTCCGCTTTAAATCATAAGATCCTTCGTTGCCTTGCGTTACGTTGCAAAACGTCAATCCTGTATGACTACAATTACACACACCGTGAATCGGCGGCGTTGCTAGCGTGTAATTCTTCGTCCCCTCCACACTACATACGTCGGTATCATTTTTACCGTCAAAAAACGACCAAATCGTTTTCAGGTTAGCACTAACGGTTTTATCATCACCGG---------------------TCTTTACACGAACATTAGCCATTCCCTCGACTCCGTAATTCAACGTCAAAATTATACACGCTATAAGTACCCCCCCCCC-----ACAATGGAATGCTGCCAAACTGGTTCTTTCCCGTTATAGCCAT

BE/29/2011 TTACTTAGTTACAATAAGAAACCAAGTATCCACTTGTTCAGGACCGTTATCAACTCTCCGCTTTAAATCATAAGATCCTTCGTTGCCTTGCGTTACGTTGCAAAACGTCAATCCTGTATGACTACAATTACACACACCGTGAATCGGCGGCGTTGCTAGCGTGTAATTCTTCGTCCCCTCCACACTACATACGTCGGTATCATTTTTACCGTCAAAAAACGACCAAATCGTTTTCAGGTTAGCACTAACGGTTTTATCATCACCGG---------------------TCTTTACACGAACATTAGCCATTCCCTCGACTCCGTAATTCAACGTCAAAATTATACACGCTATAAGTACCCCCCCCCC-----ACAATGGAATGCTGCCAAACTGGTTCTTTCCCGTTATAGCCAT

PAV4 TTACTTAGTTACAATAAGAAACCAAGTATCCACTTGTTCAGGACCGTTATCAACTCTCCGCTTTAAATCATAAGATCCTTCGTTGCCTTGCGTTACGTTGCAAAACGTCAATCCTGTATGACTACAATTACACACACCGTGAATCGGCGGCGTTGCTAGCGTGTAATTCTTCGTCCCCTCCACACTACATACGTCGGTATCATTTTTACCGTCAAAAAACGACCAAATCGTTTTCAGGTTAGCACTAACGGTTTTATCATCACCGG---------------------TCTTTACACGAACATTAGCCATTCCCTCGACTCCGTAATTCAACGTCAAAATTATACACGCTATAAGTACCCCCCCCCC-----ACAATGGAATGCTGCCAAACTGGTTCTTTCCCGTTATAGCCAT

PRA6 TTACTTAGTTACAATAAGAAACCAAGTATCCACTTGTTCAGGACCGTTATCAACTCTCCGCTTTAAATCATAAGATCCTTCGTTGCCTTGCGTTACGTTGCAAAACGTCAATCCTGTATGACTACAATTACACACACCGTGAATCGGCGGCGTTGCTAGCGTGTAATTCTTCGTCCCCTCCACACTACATACGTCGGTATCATTTTTACCGTCAAAAAACGACCAAATCGTTTTCAGGTTAGCACTAACGGTTTTATCATCACCGG---------------------TCTTTACACGAACATTAGCCATTCCCTCGACTCCGTAATTCAACGTCAAAATTATACACGCTATAAGTACCCCCCCCCC-----ACAATGGAATGCTGCCAAACTGGTTCTTTCCCGTTATAGCCAT

Pat_D TTACTTAGTTACAATAAGAAACCAAGTATCCACTTGTTCAGGACCGTTATCAACTCTCCGCTTTAAATCATAAGATCCTTCGTTGCCTTGCGTTACGTTGCAAAACGTCAATCCTGTATGACTACAATTACACACACCGTGAATCGGCGGCGTTGCTAGCGTGTAATTCTTCGTCCCCTCCACACTACATACGTCGGTATCATTTTTACCGTCAAAAAACGACCAAATCGTTTTCAGGTTAGCACTAACGGTTTTATCATCACCGG---------------------TCTTTACACGAACATTAGCCATTCCCTCGACTCCGTAATTCAACGTCAAAATTATACACGCTATAAGTACCCCCCCCCC-----ACAATGGAATGCTGCCAAACTGGTTCTTTCCCGTTATAGCCAT

JER5268 TTACTTAGTTACAATAAGAAACCAAGTATCCACTTGTTCAGGACCGTTATCAACTCTCCGCTTTAAATCATAAGATCCTTCGTTGCCTTGCGTTACGTTGCAAAACGTCAATCCTGTATGACTACAATTACACACACCGTGAATCGGCGGCGTTGCTAGCGTGTAATTCTTCGTCCCCTCCACGCTACATACGTCGGTATCATTTTTACCGTCAAAAAACGACCAAATCGTTTTCAGGTTAGCACTAACGGTTTTATCATCACCGG---------------------TCTTTACACGAACATTAGCCATTCCCTCGACTCCGTAATTCAACGTCAAAATTATACACGCTATAAGTACCCCCCCCCC-----ACAATGGAATGCTGCCAAACTGGTTCTTTCCCGTTATAGCCAT

BE/32/2010 TTACTTAGTTACAATAAGAAACCAAGTATCCACTTGTTCAGGACCGTTATCAACTCTCCGCTTTAAATCATAAGATCCTTCGTTGCCTTGCGTTACGTTGCAAAACGTCAATCCTGTATGACTACAATTACACACACCGTGAATCGGCGGCGTTGCTAGCGTGTAATTCTTCGTCCCCTCCACGCTACATACGTCGGTATCATTTTTACCGTCAAAAAACGACCAAATCGTTTTCAGGTTAGCACTAACGGTTTTATCATCACCGG---------------------TCTTTACACGAACATTAGCCATTCCCTCGACTCCGTAATTCAACGTCAAAATTATACACGCTATAAGTACCCCCCCCCC-----ACAATGGAATGCTGCCAAACTGGTTCTTTCCCGTTATAGCCAT

BE/38/2011 G5 TTACTTAGTTACAATAAGAAACCAGGTATCCACT------------------------------------------------------------------------------------------------------------------------------------------------------------------------------------------------------------------------------------------------------------------------------------------------------------------------------------------------------TGAATGTTGCCAAACTGGTTCTTCCCCGTTATAGTCAT

HAN12 TTACTTAGTTACAATAAGAAACCAGGTATCCACT------------------------------------------------------------------------------------------------------------------------------------------------------------------------------------------------------------------------------------------------------------------------------------------------------------------------------------------------------TGAATGTTGCCAAACTGGTTCTTCCCCGTTATAGTCAT

HAN15 TTACTTAGTTACAATAAGAAACCAGGTATCCACT------------------------------------------------------------------------------------------------------------------------------------------------------------------------------------------------------------------------------------------------------------------------------------------------------------------------------------------------------TGAATGTTGCCAAACTGGTTCTTCCCCGTTATAGTCAT

BE/14/2012 TTACTTAGTTACAATAAGAAACCAGGTATCCACT------------------------------------------------------------------------------------------------------------------------------------------------------------------------------------------------------------------------------------------------------------------------------------------------------------------------------------------------------TGAATGTTGCCAAACTGGTTCTTCCCCGTTATAGTCAT

BE/36/2011 TTACTTAGTTACAATAAGAAACCAGGTATCCACT------------------------------------------------------------------------------------------------------------------------------------------------------------------------------------------------------------------------------------------------------------------------------------------------------------------------------------------------------TGAATGTTGCCAAACTGGTTCTTCCCCGTTATAGTCAT

PAV24 TTACTTAGTTACAATAAGAAACCAGGTATCCACT------------------------------------------------------------------------------------------------------------------------------------------------------------------------------------------------------------------------------------------------------------------------------------------------------------------------------------------------------TGAATGTTGCCAAACTGGTTCTTCCCCGTTATAGTCAT

UKNEQAS2 TTACTTAGTTACAATAAGAAACCAGGTATCCACT------------------------------------------------------------------------------------------------------------------------------------------------------------------------------------------------------------------------------------------------------------------------------------------------------------------------------------------------------TGAATGTTGCCAAACTGGTTCTTCCCCGTTATAGTCAT

BE/37/2011 TTACTTAGTTACAATAAGAAACCAGGTATCCACT------------------------------------------------------------------------------------------------------------------------------------------------------------------------------------------------------------------------------------------------------------------------------------------------------------------------------------------------------TGAATGTTGCCAAACTGGTTCTTCCCCGTTATAGTCAT

BE/5/2010 TTACTTAGTTACAATAAGAAACCAGGTATCCACT------------------------------------------------------------------------------------------------------------------------------------------------------------------------------------------------------------------------------------------------------------------------------------------------------------------------------------------------------TGAATGTTGCCAAACTGGTTCTTCCCCGTTATAGTCAT

BE/43/2011 TTACTTAGTTACAATAAGAAACCAGGTATCCACT------------------------------------------------------------------------------------------------------------------------------------------------------------------------------------------------------------------------------------------------------------------------------------------------------------------------------------------------------TGAATGTTGCCAAACTGGTTCTTCCCCGTTATAGTCAT

BE/12/2011 TTACTTAGTTACAATAAGAAACCAGGTATCCACT------------------------------------------------------------------------------------------------------------------------------------------------------------------------------------------------------------------------------------------------------------------------------------------------------------------------------------------------------TGAATGTTGCCAAACTGGTTCTTCCCCGTTATAGTCAT

BE/6/2012 TTACTTAGTTACAATAAGAAACCAGGTATCCACT------------------------------------------------------------------------------------------------------------------------------------------------------------------------------------------------------------------------------------------------------------------------------------------------------------------------------------------------------TGAATGTTGCCAAACTGGTTCTTCCCCGTTATAGTCAT

NAN1LA TTACTTAGTTACAATAAGAAACCAGGTATCCACT------------------------------------------------------------------------------------------------------------------------------------------------------------------------------------------------------------------------------------------------------------------------------------------------------------------------------------------------------TGAATGTTGCCAAACTGGTTCTTCCCCGTTATAGTCAT

Pat_A TTACTTAGTTACAATAAGAAACCAGGTATCCACT------------------------------------------------------------------------------------------------------------------------------------------------------------------------------------------------------------------------------------------------------------------------------------------------------------------------------------------------------TGAATGTTGCCAAACTGGTTCTTCCCCGTTATAGTCAT

Pat_F TTACTTAGTTACAATAAGAAACCAGGTATCCACT------------------------------------------------------------------------------------------------------------------------------------------------------------------------------------------------------------------------------------------------------------------------------------------------------------------------------------------------------TGAATGTTGTCAAACTGGTTCTTCCCCGTTATAGTCAT

HAN36 TTACTTAGTTACAATAAGGAACCATGTATCCACT------------------------------------------------------------------------------------------------------------------------------------------------------------------------------------------------------------------------------------------------------------------------------------------------------------------------------------------------------TGAATGTTGCCCAACTGGGTCTTCCCCGTTATAGTCAT

TB40/E TTACTTAGTTACAATAAGGAACCATGTATCCACT------------------------------------------------------------------------------------------------------------------------------------------------------------------------------------------------------------------------------------------------------------------------------------------------------------------------------------------------------TGAATGTTGCCCAACTGGGTCTTCCCCGTTATAGTCAT

HAN25 TTACTTAGTTACAATAAGGAACCATGTATCCACT------------------------------------------------------------------------------------------------------------------------------------------------------------------------------------------------------------------------------------------------------------------------------------------------------------------------------------------------------TGAATGTTGCCCAACTGGGTCTTCCCCGTTATAGTCAT

BE/19/2011 TTACTTAGTTACAATAAGGAACCATGTATCCACT------------------------------------------------------------------------------------------------------------------------------------------------------------------------------------------------------------------------------------------------------------------------------------------------------------------------------------------------------TGAATGTTGCCCAACTGGGTCTTCCCCGTTATAGTCAT

HAN17 TTACTTAGTTACAATAAGGAACCATGTATCCACT------------------------------------------------------------------------------------------------------------------------------------------------------------------------------------------------------------------------------------------------------------------------------------------------------------------------------------------------------TGAATGTTGCCCAACTGGGTCTTCCCCGTTATAGTCAT

CZ/2/2013 TTACTTAGTTACAATAAGGAACCATGTATCCACT------------------------------------------------------------------------------------------------------------------------------------------------------------------------------------------------------------------------------------------------------------------------------------------------------------------------------------------------------TGAATGTTGCCCAACTGGGTCTTCCCCGTTATAGTCAT

BE/11/2011 TTACTTAGTTACAATAAGGAACCATGTATCCACT------------------------------------------------------------------------------------------------------------------------------------------------------------------------------------------------------------------------------------------------------------------------------------------------------------------------------------------------------TGAATGTTGCCCAACTGGGTCTTCCCCGTTATAGTCAT

PAV32 TTACTTAGTTACAATAAGGAACCATGTATCCACT------------------------------------------------------------------------------------------------------------------------------------------------------------------------------------------------------------------------------------------------------------------------------------------------------------------------------------------------------TGAATGTTGCCCAACTGGGTCTTCCCCGTTATAGTCAT

HANRTR8 TTACTTAGTTACAATAAGGAACCATGTATCCACT------------------------------------------------------------------------------------------------------------------------------------------------------------------------------------------------------------------------------------------------------------------------------------------------------------------------------------------------------TGAATGTTGCCCAACTGGGTCTTCCCCGTTATAGTCAT

HANSCTR11B TTACTTAGTTACAATAAGGAACCATGTATCCACT------------------------------------------------------------------------------------------------------------------------------------------------------------------------------------------------------------------------------------------------------------------------------------------------------------------------------------------------------TGAATGTTGCCCAACTGGGTCTTCCCCGTTATAGTCAT

HANSCTR10 TTACTTAGTTACAATAAGGAACCATGTATCCACT------------------------------------------------------------------------------------------------------------------------------------------------------------------------------------------------------------------------------------------------------------------------------------------------------------------------------------------------------TGAATGTTGCCCAACTGGGTCTTCCCCGTTATAGTCAT

BE/14/2010 TTACTTAGTTACAATAAGGAACCATGTATCCACT------------------------------------------------------------------------------------------------------------------------------------------------------------------------------------------------------------------------------------------------------------------------------------------------------------------------------------------------------TGAATGTTGCCCAACTGGGTCTTCCCCGTTATAATCAT

UK/Lon1 G7 TTAAGATTGTGAAACGACGGGAAACGATTCTCCCGGTTCTTCATAACCACCATACTTTGTTTCAACGCTATAAACACCTTCTTGTATTGACGTCAT-----------------ATGTATGATTACATGCACTGATACAAACAGTAATGTTTGACGCTACAACACCAGTACCATTAAACCGTACAGTACATAACACCGTATCGCCACTGTCGTCACTAGAACCACCTCCGATCTTTTTCCAGGTAGAATACACGTCAGGATTCCACGTTACTGTTCCAGTACTGTTCAGACGTATCGTAACTTCCGTAAATCCATAAATACCGTGGTTCCACGTCAAAATTATGCACGCTATAAGTACCCCCCCCCCC----ATAATGGAATGTTGCCAAACCTGTTCTTTCCCGTTATAGCCAT

BE/33/2010 TTAAGATTGTGAAACAACGGGAAACGATTCTCCCGGTTCTTCATAACCACCATACTTTGTTTCAACGCTATAAACACCTTCTTGTATTGACGTCAT-----------------ATGTATGATTACATGCACTGATACAAACAGTAATGTTTGACGCTACAACACCAGTACCATTAAACCGTACAGTACATAACACCGTATCGCCACTGTCGTCACTAGAACCACCTCCGATCTTTTTCCAGGTAGAATACACGTCAGGATTCCACGTTACTGTTCCAGTACTGTTCAGACGTATCTTAACTTCCGTAAATCCATAAATACCGTGGTTCCACGTCAAAATTATGCACGCTATAAGTACCCCCCC--------ATAATGGAATGTTGCCAAACCGGTTCTTTCCCGTTATAGCCAT

HAN38 TTAAGATTGTGAAACAACGGGAAACGATTCTCCCGGTTCTTCATAACCACCATACTTTGTTTTAACGCTATAAACACCTTCTTGTATTGACGTCAT-----------------ATGTATGATTACATGCACTGATACAAACAGTAATGTTTGACGCTACAACACCAATATCATTAAACCGTACAGTACATAACACCGTATCGCCACTGTCGTCACTAGAACCACCTCCGATCTTTTTCCAGGTAGAATACACGTCAGGATTCCACGTTACTGTTCCAGTACTGTTCAGACGTATCTTAACTTCCGTAAATCCATAAATACCGTGGTTCCACGTCAAAATTATGCACGCTATAAGTACCCCCCC--------ATAATGGAATGTTGCCAAACCGGTTCTTTCCCGTTATAGCCAT

BE/46/2011 G4 TTAAGATTGTAAAACAACAGGAAACGATTCTCCCGGTCCCTCGTAACCACCATACTCTGTTTCAACGCTATAAACACCTTCTTGCGTTGCCGTCATATTGCATATGGTTAACGATGTATGATTGCATGCACTGATACAAATAGTAATATTTGCTGCCACCACACCAGTCTCATTAAACCGTACTGTACATAACACCGTATCCACACTGTTTCCGTTAGAATCTCCCCCGACTTTTTTCCAAGTAGAATGTACGTCAGGGCTCCACGTTACAGTTCCATTTCCATTAACAGGAATTTTAACCTGCGTAAATCCATAAATACCGTGGTTCCACGTCAAAATTATGCACGCTATAAGTACCCCCCCCCCC----ACAATGGAATGCTGCCAAACTGGTTCTTTCCCGTTATAGCCAT

BE/3/2011 TTAAGATTGTAAAACAACAGGAAACGATTCTCCCGGTCCCTCGTAACCACCATACTCTGTTTCAACGCTATAAACACCTTCTTGCGTTGCCGTCATATTGCATATGGTTAACGATGTATGATTGCATGCACTGATACAAATAGTAATATTTGCTGCCACCACACCAGTCTCATTAAACCGTACTGTACATAACACCGTATCCACACTGTTTCCGTTAGAATCTCCCCCGACTTTTTTCCAAGTAGAATGTACGTCAGGGCTCCACGTTACAGTTCCATTTCCATTAACAGGAATTTTAACCTGCGTAAATCCATAAATACCGTGGTTCCACGTCAAAATTATGCACGCTATAAGTACCCCCCCCCCC----ACAATGGAATGCTGCCAAACTGGTTCTTTCCCGTTATAGCCAT

BE/30/2010 TTAAGATTGTAAAACAACGGGAAACGATTCTCCCGGTCCCTCGTAACCACCATACTCTGTTTCGACGCTATAAACACCTTCTTGCGTTGCCGTCATATTGCATATGGTTAACGATGTATGATTGCATGCACTGATACAAATAGTAATATTTGCTGCCACCACACCAGTCTCATTAAACCGTACTGTACATAACACCGTATCCACACTGTTTCCGTTAGAATCTCCCCCGACTTTTTTCCAAGTAGAATGTACGTCAGGGCTCCACGTTACAGTTCCATTTCCATTAACAGGAATTTTAACCTGCGTAAATCCATAAATACCGTGGTTCCACGTCAAAATTATGCACGCTATAAGTACCCCCCCCCCC----ACAATGGAATGCTGCCAAACTGGTTCTTTCCCGTTATAGCCAT

JER5550 TTAAGATTGTAGAACAACGGGAAACGATTCTCCCGGTCCCTCGTAACCACCATACTCTGTTTCGACGCTATAAACACCTTCTTGCGTTGCCGTCATATTGCATATGGTTAACGATGTATGATTGCATGCACTGATACAAATAGTAATATTTGCTGCCACCACACCAGTCTCATTAAACCGTACTGTACATAACACCGTATCCACACTGTTTCCGTTAGAATCTCCCCCGACTTTTTTCCAAGTAGAATGTACGTCAGGGCTCCACGTT--AGTTCCATTTCCATTAACAGGAATTTTAACCTGCGTAAATCCATAAATACCGTGGTTCCACGTCAAAATTATGCACGCTATAAGTACCCCCCCCC------ACAATGGAATGCTGCCAAACTGGTTCTTTCCCGTTATAGCCAT

HAN39 TTAAGATTGTAGAACAACGGGAAACGATTCTCCCGGTCCCTCGTAACCACCATACTCTGTTTCGACGCTATAAACACCTTCTTGCGTTGCCGTCATATTGCATATGGTTAACGATGTATGATTGCATGCACTGATACAAATAGTAATATTTGCTGCCACCACACCAGTCTCATTAAACCGTACTGTACATAACACCGTATCCACACTGTTTCCGTTAGAATCTCCCCCGACTTTTTTCCAAGTAGAATGTACGTCAGGGCTCCACGTTACAGTTCCATTTCCATTAACAGGAATTTTAACCTGCGTAAATCCATAAATACCGTGGTTCCACGTCAAAATTATGCACGCTATAAGTACCCCCCCCC------ACAATGAAATGCTGCCAAACTGGTTCTTTCCCGTTATAGCCAT

HAN22 TTAAGATTGTAAAACAACAGGAAACGATTCTCCCGGTCCCTCGTAACCACCATACTCTGTTTCAACGCTATAAACACCTTCTTGCGTTGCCGTCATATTGCATATGGTTAACGATGTATGATTGCATGCACTGATACAAATAGTAATATTTGCTGCCACCACACCAGTCTCATTAAACCGTACTGTACATAACACCGTATCCACACTGTTTCCGTTAGAATCTCCCCCGACTTTTTTCCAAGTAGAATGTACGTCAGGGCTCCACGTTACAGTTCCATTTCCATTAACAGGAATTTTAACCTGCGTAAATCCATAAATACCGTGGTTCCACGTCAAAATTATGCACGCTATAAGTACCCCCCCCC------ACAATGGAATGCTGCCAAACTGGTTCTTTCCCGTTATAGCCAT

HAN23 TTAAGATTGTAAAACAACAGGAAACGATTCTCCCGGTCCCTCGTAACCACCATACTCTGTTTCAACGCTATAAACACCTTCTTGCGTTGCCGTCATATTGCATATGGTTAACGATGTATGATTGCATGCACTGATACAAATAGTAATATTTGCTGCCACCACACCAGTCTCATTAAACCGTACTGTACATAACACCGTATCCACACTGTTTCCGTTAGAATCTCCCCCGACTTTTTTCCAAGTAGAATGTACGTCAGGGCTCCACGTTACAGTTCCATTTCCATTAACAGGAATTTTAACCTGCGTAAATCCATAAATACCGTGGTTCCACGTCAAAATTATGCACGCTATAAGTACCCCCCCCC------ACAATGGAATGCTGCCAAACTGGTTCTTTCCCGTTATAGCCAT

BE/2/2011 TTAAGATTGTAAAACAACAGGAAACGATTCTCCCGGTCCCTCGTAACCACCATACTCTGTTTCAACGCTATAAACACCTTCTTGCGTTGCCGTCATATTGCATATGGTTAACGATGTATGATTGCATGCACTGATACAAATAGTAATATTTGCTGCCACCACACCAGTCTCATTAAACCGTACTGTACATAACACCGTATCCACACTGTTTCCGTTAGAATCTCCCCCGACTTTTTTCCAAGTAGAATGTACGTCAGGGCTCCACGTTACAGTTCCATTTCCATTAACAGGAATTTTAACCTGCGTAAATCCATAAATACCGTGGTTCCACGTCAAAATTATGCACGCTATAAGTACCCCCCCCC------ACAATGGAATGCTGCCAAACTGGTTCTTTCCCGTTATAGCCAT

BE/7/2011 TTAAGATTGTAAAACAACAGGAAACGATTCTCCCGGTCCCTCGTAACCACCATACTCTGTTTCAACGCTATAAACACCTTCTTGCGTTGCCGTCATATTGCATATGGTTAACGATGTATGATTGCATGCACTGATACAAATAGTAATATTTGCTGCCACCACACCAGTCTCATTAAACCGTACTGTACATAACACCGTATCCACACTGTTTCCGTTAGAATCTCCCCCGACTTTTTTCCAAGTAGAATGTACGTCAGGGCTCCACGTTACAGTTCCATTTCCATTAACAGGAATTTTAACCTGCGTAAATCCATAAATACCGTGGTTCCACGTCAAAATTATGCACGCTATAAGTACCCCCCCCC------ACAATGGAATGCTGCCAAACTGGTTCTTTCCCGTTATAGCCAT

BE/31/2011 TTAAGATTGTAAAACAACAGGAAACGATTCTCCCGGTCCCTCGTAACCACCATACTCTGTTTCAACGCTATAAACACCTTCTTGCGTTGCCGTCATATTGCATATGGTTAACGATGTATGATTGCATGCACTGATACAAATAGTAATATTTGCTGCCACCACACCAGTCTCATTAAACCGTACTGTACATAACACCGTATCCACACTGTTTCCGTTAGAATCTCCCCCGACTTTTTTCCAAGTAGAATGTACGTCAGGGCTCCACGTTACAGTTCCATTTCCATTAACAGGAATTTTAACCTGCGTAAATCCATAAATACCGTGGTTCCACGTCAAAATTATGCACGCTATAAGTACCCCCCCCC------ACAATGGAATGCTGCCAAACTGGTTCTTTCCCGTTATAGCCAT

BE/21/2011 TTAAGATTGTAAAACAACAGGAAACGATTCTCCCGGTCCCTCGTAACCACCATACTCTGTTTCAACGCTATAAACACCTTCTTGCGTTGCCGTCATATTGCATATGGTTAACGATGTATGATTGCATGCACTGATACAAATAGTAATATTTGCTGCCACCACACCAGTCTCATTAAACCGTACTGTACATAACACCGTATCCACACTGTTTCCGTTAGAATCTCCCCCGACTTTTTTCCAAGTAGAATGTACGTCAGGGCTCCACGTTACAGTTCCATTTCCATTAACAGGAATTTTAACCTGCGTAAATCCATAAATACCGTGGTTCCACGTCAAAATTATGCACGCTATAAGTACCCCCCCCC------ACAATGGAATGCTGCCAAACTGGTTCTTTCCCGTTATAGCCAT

BE/7/2012 TTAAGATTGTAAAACAACAGGAAACGATTCTCCCGGTCCCTCGTAACCACCATACTCTGTTTCAACGCTATAAACACCTTCTTGCGTTGCCGTCATATTGCATATGGTTAACGATGTATGATTGCATGCACTGATACAAATAGTAATATTTGCTGCCACCACACCAGTCTCATTAAACCGTACTGTACATAACACCGTATCCACACTGTTTCCGTTAGAATCTCCCCCGACTTTTTTCCAAGTAGAATGTACGTCAGGGCTCCACGTTACAGTTCCATTTCCATTAACAGGAATTTTAACCTGCGTAAATCCATAAATACCGTGGTTCCACGTCAAAATTATGCACGCTATAAGTACCCCCCCCC------ACAATGGAATGCTGCCAAACTGGTTCTTTCCCATTATAGCCAT

BE/40/2011 TTAAGATTGTAAAACAACGGGAAACGATTCTCCCGGTCCCTCGTAACCACCATACTCTGTTTCGACGCTATAAACACCTTCTTGCGTTGCCGTCATATTGCATATGGTTAACGATGTATGATTGCATGCACTGATACAAATAGTAATATTTGCTGCCACCACACCAGTCTCATTAAACCGTACTGTACATAACACCGTATCCACA--GTTTCCGTTATAATCTCCCCCGACTTTTTTCCAAGTAGAATGTACGTCAGGGCTCCACGTTACAGTTCCATTTCCATTAACAGGAATTTTAACCTGCGTAAATCCATAAATACCGTGGTTCCACGTCAAAATTATGCACGCTATAAGTACCCCCCCCCC-----ACAATGGAATGCTGCCAAACTGGTTCTTTCCCGTTATAGCCAT

PAV18 TTAAGATTGTAAAACAACAGGAAACGATTCTCCCGGTCCCTCGTAACCGCCATACTCTGTTTCAACGCTATAAACACCTTCTTGCGTTGCCGTCATATTGCATATGGTTAACGATGTATGATTGCATGCACTGATACAAATAGTAATATTTGCTGCCACCACACCAGTCTCATTAAACCGTACTGTACATAACACCGTATCCACACTGTTTCCGTTAGAATCTCCCCCGACTTTTTTCCAAGTAGAATGTACGTCAGGGCTCCACGTTACAGTTCCATTTCCATTAACAGGAATTTTAACCTGCGTAAATCCATAAATACCGTGGTTCCACGTCAAAATTATGCACGCTATAAGTACCCCCCC--------ACAATGGAATGCTGCCAAACTGGTTCTTTCCCGTTATAGCCAT

PAV25 TTAAGATTGTAAAACAACAGGAAACGATTCTCCCGGTCCCTCGTAACCACCATAC--TGTTTCAACGCTATAAACACCTTCTTGCGTTGCCGTCATATTGCATATGGTTAACGATGTATGATTGCATGCACTGATACAAATAGTAATATTTGCTGCCACCACACCAGTCTCATTAAACCGTACTGTACATAACACCGTATCCACACTGTTTCCGTTAGAATCTCCCCCGACTTTTTTCCAAGTAGAATGTACGTCAGGGCTCCACGTTACAGTTCCATTTCCATTAACAGGAATTTTAACCTGCGTAAATCCATAAATACCGTGGTTCCACGTCAAAATTATGCACGCTATAAGTACCCCCCCCCC-----ACAATGGAATGCTGCCAAACTGGTTCTTTCCCGTTATAGCCAT

BE/17/2011 TTAAGATTGTAAAACAACGGGAAACGATTCTCCCGGTCCCTCGTAACCACCATACTCTGTTTCAACGCTATAAACACCTTCTTGCGTTGCCGTCATATTGCATATGGTTAACGATGTATGATTGCATGCACTGATACAAATAGTAATATTTGCTGCCACCACACCAGTCTCATTAAACCGTACTGTACATAACACCGTATCCACACTGTTTCCGTTAGAATCCCCCCCGACTTTTTTCCAAGTAGAATGTACGTCAGGGCTCCACGTTACAGTTCCATTTCCATTAACAGGAATTTTAACCTGCGTAAATCCATAAATACCGTGGTTCCACGTCAAAATTATGCACGCTATAAGTACCCCCCCCCC-----ACAATGGAATGCTGCCAAACCGGTTATTTCCCGTTATAGCCAT

HAN28 TTAAGATTGTAGAACAACGGGAAACGATTCTCCCGGTCCCTCGTAACCACCATACTCTGTTTCGACGCTATAAACACCTTCTTGCGTTGCCGTCATATTGCATATGGTTAACGATGTATGATTGCATGCACTGATACAAATAGTAATATTTGCTGCCACCACACCAGTCTCATTAAACCGTACTGTACATAACACCGTATCCACACTGTTTCCGTTAGAATCTCCCCCGACTTTTTTCCAAGTAGAATGTACGTCAGGGCTCCACGTTACAGTTCCATTTCCATTAACAGGAATTTTAACCTGCGTAAATCCATAAATACCGTGGTTCCACGTCAAAATTATGCACGCTATAAGTACCCCCCCCCC-----ACAATGGAATGCTGCCAAACTGGTTCTTTCCCGTTATAGCCAT

HAN21 TTAAGATTGTAAAACAACAGGAAACGATTCTCCCGGTCCCTCGTAACCGCCATACTCTGTTTCAACGCTATAAACACCTTCTTGCGTTGCCGTCATATTGCATATGGTTAACGATGTATGATTGCATGCACTGATACAAATAGTAATATTTGCTGCCACCACACCAGTCTCATTAAACCGTACTGTACATAACACCGTATCCACACTGTTTCCGTTAGAATCTCCCCCGACTTTTTTCCAAGTAGAATGTACGTCAGGGCTCCACGCTACAGTTCCATTTCCATTAACAGGAATTTTAACCTGCGTAAATCCATAAATACCGTGGTTCCACGTCAAAATTATGCACGCTGTAAGTACCCCCCCCCC-----ACAATGGAATGCTGCCAAACTGGTTCTTTCCCGTTATAGCCAT

HANSCTR1A TTAAGATTGTAAAACAACAGGAAACGATTCTCCCGGTCCCTCGTAACCGCCATACTCTGTTTCAACGCTATAAACACCTTCTTGCGTTGCCGTCATATTGCATATGGTTAACGATGTATGATTGCATGCACTGATACAAATAGTAATATTTGCTGCCACCACACCAGTCTCATTAAACCGTACTGTACATAACACCGTATCCACACTGTTTCCGTTAGAATCTCCCCCGACTTTTTTCCAAGTAGAATGTACGTCAGGGCTCCACGTTACAGTTCCATTTCCATTAACAGGAATTTTAACCTGCGTAAATCCATAAATACCGTGGTTCCACGTCAAAATTATGCACGCTATAAGTACCCCCCCCCC-----ACAATGGAATGCTGCCAAACTGGTTCTTTCCCGTTATAGCCAT

HAN31 TTAAGATTGTAAAACAACAGGAAACGATTCTCCCGGTCCCTCGTAACCACCATACTCTGTTTCAACGCTATAAACACCTTCTTGCGTTGCCGTCATATTGCATATGGTTAACGATGTATGATTGCATGCACTGATACAAATAGTAATATTTGCTGCCACCACACCAGTCTCATTAAACCGTACTGTACATAACACCGTATCCACACTGTTTCCGTTAGAATCTCCCCCGACTTTTTTCCAAGTAGAATGTACGTCAGGGCTCCACGTTACAGTTCCATTTCCATTAACAGGAATTTTAACCTGCGTAAATCCATAAATACCGTGGTTCCACGTCAAAATTATGCACGCTATAAGTACCCCCCCCCC-----ACAATGGAATGCTGCCAAACTGGTTCTTTCCCATTATAGCCAT

BE/16/2012 TTAAGATTGTAAAACAACAGGAAACGATTCTCCCGGTCCCTCGTAACCACCATACTCTGTTTCAACGCTATAAACACCTTCTTGCGTTGCCGTCATATTGCATATGGTTAACGATGTATGATTGCATGCACTGATACAAATAGTAATATTTGCTGCCACCACACCAGTCTCATTAAACCGTACTGTACATAACACCGTATCCACACTGTTTCCGTTAGAATCTCCCCCGACTTTTTTCCAAGTAGAATGTACGTCAGGGCTCCACGTTACAGTTCCATTTCCATTAACAGGAATTTTAACCTGCGTAAATCCATAAATACCGTGGTTCCACGTCAAAATTATGCACGCTATAAGTACCCCCCCCCC-----ACAATGGAATGCTGCCAAACTGGTTCTTTCCCATTATAGCCAT

BE/30/2011 TTAAGATTGTAAAACAACAGGAAACGATTCTCCCGGTCCCTCGTAACCACCATACTCTGTTTCAACGCTATAAACACCTTCTTGCGTTGCCGTCATATTGCATATGGTTAACGATGTATGATTGCATGCACTGATACAAATAGTAATATTTGCTGCCACCACACCAGTCTCATTAAACCGTACTGTACATAACACCGTATCCACACTGTTTCCGTTAGAATCTCCCCCGACTTTTTTCCAAGTAGAATGTACGTCAGGGCTCCACGTTACAGTTCCATTTCCATTAACAGGAATTTTAACCTGCGTAAATCCATAAATACCGTGGTTCCACGTCAAAATTATGCACGCTATAAGTACCCCCCCCCC-----ACAATGGAATGCTGCCAAACTGGTTCTTTCCCATTATAGCCAT

BE/15/2012 TTAAGATTGTAAAACAACAGGAAACGATTCTCCCGGTCCCTCGTAACCACCATACTCTGTTTCAACGCTATAAACACCTTCTTGCGTTGCCGTCATATTGCATATGGTTAACGATGTATGATTGCATGCACTGATACAAATAGTAATATTTGCTGCCACCACACCAGTCTCATTAAACCGTACTGTACATAACACCGTATCCACACTGTTTCCGTTAGAATCTCCCCCGACTTTTTTCCAAGTAGAATGTACGTCAGGGCTCCACGTTACAGTTCCATTTCCATTAACAGGAATTTTAACCTGCGTAAATCCATAAATACCGTGGTTCCACGTCAAAATTATGCACGCTATAAGTACCCCCCCCCC-----ACAATGGAATGCTGCCAAACTGGTTCTTTCCCATTATAGCCAT

BE/11/2012 TTAAGATTGTAAAACAACAGGAAACGATTCTCCCGGTCCCTCGTAACCACCATACTCTGTTTCAACGCTATAAACACCTTCTTGCGTTGCCGTCATATTGCATATGGTTAACGATGTATGATTGCATGCACTGATACAAATAGTAATATTTGCTGCCACCACACCAGTCTCATTAAACCGTACTGTACATAACACCGTATCCACACTGTTTCCGTTAGAATCTCCCCCGACTTTTTTCCAAGTAGAATGTACGTCAGGGCTCCACGTTACAGTTCCATTTCCATTAACAGGAATTTTAACCTGCGTAAATCCATAAATACCGTGGTTCCACGTCAAAATTATGCACGCTATAAGTACCCCCCCCCC-----ACAATGGAATGCTGCCAAACTGGTTCTTTCCCATTATAGCCAT

BE/26/2010 TTAAGATTGTAAAACAACAGGAAACGATTCTCCCGGTCCCTCGTAACCACCATACTCTGTTTCAACGCTATAAACACCTTCTTGCGTTGCCGTCATATTGCATATGGTTAACGATGTATGATTGCATGCACTGATACAAATAGTAATATTTGCTGCCACCACACCAGTCTCATTAAACCGTACTGTACATAACACCGTATCCACACTGTTTCCGTTAGAATCTCCCCCGACTTTTTTCCAAGTAGAATGTACGTCAGGGCTCCACGTTACAGTTCCATTTCCATTAACAGGAATTTTAACCTGCGTAAATCCATAAATACCGTGGTTCCACGTCAAAATTATGCACGCTATAAGTACCCCCCCCCC-----ACAATGGAATGCTGCCAAACTGGTTCTTTCCCATTATAGCCAT

BE/3/2012 TTAAGATTGTAAAACAACAGGAAACGATTCTCCCGGTCCCTCGTAACCACCATACTCTGTTTCAACGCTATAAACACCTTCTTGCGTTGCCGTCATATTGCATATGGTTAACGATGTATGATTGCATGCACTGATACAAATAGTAATATTTGCTGCCACCACACCAGTCTCATTAAACCGTACTGTACATAACACCGTATCCACACTGTTTCCGTTAGAATCTCCCCCGACTTTTTTCCAAGTAGAATGTACGTCAGGGCTCCACGTTACAGTTCCATTTCCATTAACAGGAATTTTAACCTGCGTAAATCCATAAATACCGTGGTTCCACGTCAAAATTATGCACGCTATAAGTACCCCCCCCCC-----ACAATGGAATGCTGCCAAACTGGTTCTTTCCCATTATAGCCAT

BE/31/2010 TTAAGATTGTAAAACAACAGGAAACGATTCTCCCGGTCCCTCGTAACCACCATACTCTGTTTCAACGCTATAAACACCTTCTTGCGTTGCCGTCATATTGCATATGGTTAACGATGTATGATTGCATGCACTGATACAAATAGTAATATTTGCTGCCACCACACCAGTCTCATTAAACCGTACTGTACATAACACCGTATCCACACTGTTTCCGTTAGAATCTCCCCCGACTTTTTTCCAAGTAGAATGTACGTCAGGGCTCCACGTTACAGTTCCATTTCCATTAACAGGAATTTTAACCTGCGTAAATCCATAAATACCGTGGTTCCACGTCAAAATTATGCACGCTATAAGTACCCCCCCCCC-----ACAATGGAATGCTGCCAAACTGGTTCTTTCCCGTTATAGCCAT

BE/32/2011 TTAAGATTGTAAAACAACAGGAAACGATTCTCCCGGTCCCTCGTAACCACCATACTCTGTTTCAACGCTATAAACACCTTCTTGCGTTGCCGTCATATTGCATATGGTTAACGATGTATGATTGCATGCACTGATACAAATAGTAATATTTGCTGCCACCACACCAGTCTCATTAAACCGTACTGTACATAACACCGTATCCACACTGTTTCCGTTAGAATCTCCCCCGACTTTTTTCCAAGTAGAATGTACGTCAGGGCTCCACGTTACAGTTCCATTTCCATTAACAGGAATTTTAACCTGCGTAAATCCATAAATACCGTGGTTCCACGTCAAAATTATGCACGCTATAAGTACCCCCCCCCC-----ACAATGGAATGCTGCCAAACTGGTTCTTTCCCGTTATAGCCAT

BE/34/2011 TTAAGATTGTAAAACAACAGGAAACGATTCTCCCGGTCCCTCGTAACCACCATACTCTGTTTCAACGCTATAAACACCTTCTTGCGTTGCCGTCATATTGCATATGGTTAACGATGTATGATTGCATGCACTGATACAAATAGTAATATTTGCTGCCACCACACCAGTCTCATTAAACCGTACTGTACATAACACCGTATCCACACTGTTTCCGTTAGAATCTCCCCCGACTTTTTTCCAAGTAGAATGTACGTCAGGGCTCCACGTTACAGTTCCATTTCCATTAACAGGAATTTTAACCTGCGTAAATCCATAAATACCGTGGTTCCACGTCAAAATTATGCACGCTATAAGTACCCCCCCCCC-----ACAATGGAATGCTGCCAAACTGGTTCTTTCCCGTTATAGCCAT

U8 TTAAGATTGTAAAACAACAGGAAACGATTCTCCCGGTCCCTCGTAACCACCATACTCTGTTTCAACGCTATAAACACCTTCTTGCGTTGCCGTCATATTGCATATGGTTAACGATGTATGATTGCATGCACTGATACAAATAGTAATATTTGCTGCCACCACACCAGTCTCATTAAACCGTACTGTACATAACACCGTATCCACACTGTTTCCGTTAGAATCTCCCCCGACTTTTTTCCAAGTAGAATGTACGTCAGGGCTCCACGTTACAGTTCCATTTCCATTAACAGGAATTTTAACCTGCGTAAATCCATAAATACCGTGGTTCCACGTCAAAATTATGCACGCTATAAGTACCCCCCCCCC-----ACAATGGAATGCTGCCAAACTGGTTCTTTCCCGTTATAGCCAT

HANRTR2 TTAAGATTGTAAAACAACAGGAAACGATTCTCCCGGTCCCTCGTAACCACCATACTCTGTTTCAACGCTATAAACACCTTCTTGCGTTGCCGTCATATTGCATATGGTTAACGATGTATGATTGCATGCACTGATACAAATAGTAATATTTGCTGCCACCACACCAGTCTCATTAAACCGTACTGTACATAACACCGTATCCACACTGTTTCCGTTAGAATCTCCCCCGACTTTTTTCCAAGTAGAATGTACGTCAGGGCTCCACGTTACAGTTCCATTTCCATTAACAGGAATTTTAACCTGCGTAAATCCATAAATACCGTGGTTCCACGTCAAAATTATGCACGCTATAAGTACCCCCCCCCC-----ACAATGGAATGCTGCCAAACTGGTTCTTTCCCGTTATAGCCAT

**RL12**

Initiation and termination codons are highlighted in yellow. Sequences used for motif-matching are indicated in blue font (identifying genotypes) or red font (identifying a common recombinant, with the region of recombination highlighted in grey).

Cyan highlighting indicates strains in which this gene is mutated and the mutations responsible: substitutions that introduce in-frame stop codons (underlined) or insertions or deletions that cause frameshifts.

NL/Rot4 G1B ATGCGTACTCAACATCGACGGCGAAGCAAGTCATCATATACGCAAATAATATGCATGTTTATTATATTTTGGATTCTGCAGAAAGGCAAGTGTAACAACACGAC---TGCTAATACTTCCACGTCTACAGCACTTACAAGCTTG---------ATACCTACTACACAACTGTCATCTACTTTAGAAACCACCGGAAT---GTCCACCACTACATTTACAT------CTTCCAC---------CAAT------------ACATCCACAGGATTCACCACAAATTCTGTAAAAAGCACAGATGGGAGCTCTACTATTTCTACCAGACCTATTCAAACATCTACAACCAACGCAACTGTAATGACAACCTCACCAAACAGCGA---CATTAATTCATCGACGCAACATGTAACCCATAGTACTGTGACTTTGCAAACGACATCACTAT---CAACCAACAC------TAC------TATCAT---GGTA------AATGTAACT------GAAAACGTCAGCACACCNNNNNNNNNNNNNNNNNNNN---NNNNNNNNNNNNNNNNNNNNNNNNNNNNNNNNNNNNNNNNN------------NNNNNNNNNNNNNNNNNNNNNNNNNNNNNNNNNNNNNNNNNNNNNNNNNNNNNNNNNNNNNNNNNNNNNNNNNNNNNNNNNNNNNNNNNNNNNNNNNNNNNNNNNAAACAAGCTTGGCTTAGACATTTTAATGTATCCACACACGGAAAATACACCCATCCCGGTATAAGAAATGGAAA---ATATCATAACCATTCATTGAAAATCCTGCATTCGCGTGTACTATGTGAGTGGCAAACAAATTATCTAAAGCATCACTATGATTTATGTTTTACATGCGATCAGAATTTATCTTTATCTTTGTACGGTCTTAACTTTACTCATTCTGGTAAATATAGCTTTCGATGTTACATAAATGGGTATCCCTCTGAACAAA------------ATCAAAACTTTAATTTACAAGTACATCCTAGAAACAACACAAACGGGACACACGTAAATCCCTGGATATGCGAAGAACCAAAGCACGAATGGGACACTTC------------TCATAAACCGACCAATTATGAAGACAATACAGCCACATCATCTATAGATCATTTATACCGCTATAACAATCATTCTAACACATCACACGGCAGACGCACTACGTGGACGTTAGCATTAATTTGTGTAGCCTGCATTCTCCTATTTTTCGTCCGACGAGCTCTAAATAAAAAATATCATCCATTAAGTGACGATATAAGTGAATCAGAATTCATAGTTCGATACAATCCTGAGCACGAGGACTAA

BE/39/2011 ATGCGTACTCAACATCGACGGCGAAGCAAGTCATCATATACGCAAATAATATGCATGTTTATTATATTTTGGATTCTGCAGAAAGGCAAGTGTAACAACACGAC---TGCTAATACTTCCACGTCTACAGCACTTACAAGCTTG---------ATACCTACTACACAACTGTCATCTACTTTAGAAACCACCGGAAT---GTCCACCACTACATTTACAT------CTTCCAC---------CAAT------------ACATCCACAGGATTCACCACAAATTCTGTAAAAAGCACAGATGGGAGCTCTACTATTTCTACCAGACCTATTCAAACATCTACAACCAACGCAACTGTAATGACAACCTCACCAAACAGCGA---CATTAATTCATCGACGCAACATGTAACCCATAGTACTGTGACTTTGCAAACGACATCACTAT---CAACCAACAC------TAC------TATCAT---GGTA------AATGTAACT------GAAAACGTCAGCACACCGCTTCCAATTTGCTCGTCAT---CTAATAACACATTCAACGATACATCAAATAACCATACTTGT------------TATCATGAAAATAATACAATATCAAAAGAACCTGAAACATTATTGTATGCGGTACAAGGAGACAATATTACTATAACACACAATTTAACCACCACATCGTGCTACAAACAAGCTTGGCTTAGACATTTTAATGTATCCACACACGGAAAATACACCCATCCCGGTATAAGAAATGGAAA---ATATCATAACCATTCATTGAAAATCCTGCATTCGCGTGTACTATGTGAGTGGCAAACAAATTATCTAAAGCATCACTATGATTTATGTTTTACATGCGATCAGAATTTATCTTTATCTTTGTACGGTCTTAACTTTACTCATTCTGGTAAATATAGCTTTCGATGTTACATAAATGGGTATCCCTCTGAACAAA------------ATCAAAACTTTAATTTACAAGTACATCCTAGAAACAACACAAACGGGACACACGTAAATCCCTGGATATGCGAAGAACCAAAGCACGAATGGGACACTTC------------TCATAAACCGACCAATTATGAAGACAATACAGCCACATCATCTATAGATCATTTATACCGCTATAACAATCATTCTAACACATCACACGGCAGACGCACTACGTGGACGTTAGCATTAATTTGTGTAGCCTGCATTCTCCTATTTTTCGTCCGACGAGCTCTAAATAAAAAATATCATCCATTAAGTGACGATATAAGTGAATCAGAATTCATAGTTCGATACAATCCTGAGCACGAGGACTAA

BE/48/2011 ATGCGTACTCAACATCGACGGCGAAGCAAGTCATCATATACGCAAATAATATGCATGTTTATTATATTTTGGATTCTGCAGAAAGGCAAGTGTAACAACACGAC---TGCTAATACTTCCACGTCTACAGCACTTACAAGCTTG---------ATACCTACTACACAACTGTCATCTACTTTAGAAACCACCGGAAT---GTCCACCACTACATTTACAT------CTTCCAC---------CAAT------------ACATCCACAGGATTCACCACAAATTCTGTAAAAAGCACAGATGGGAGCTCTACTATTTCTACCAGACCTATTCAAACATCTACAACCAACGCAACTGTAATGACAACCTCACCAAACAGCGA---CATTAATTCATCGACGCAACATGTAACCCATAGTACTGTGACTTTGCAAACGACATCACTAT---CAACCAACAC------TAC------TATCAT---GGTA------AATGTAACT------GAAAACGTCAGCACACCGCTTCCAATTTGCTCGTCAT---CTAATAACACATTCAACGATACATCAAATAACCATACTTGT------------TATCATGAAAATAATACAATATCAAAAGAACCTGAAACATTATTGTATGCGGTACAAGGAGACAATATTACTATAACACACAATTTAACCACCACATCGTGCTACAAACAAGCTTGGCTTAGACATTTTAATGTATCCACACACGGAAAATACACCCATCCCGGTATAAGAAATGGAAA---ATATCATAACCATTCATTGAAAATCCTGCATTCGCGTGTACTATGTGAGTGGCAAACAAATTATCTAAAGCATCACTATGATTTATGTTTTACATGCGATCAGAATTTATCTTTATCTTTGTACGGTCTTAACTTTACTCATTCTGGTAAATATAGCTTTCGATGTTACATAAATGGGTATCCCTCTGAACAAA------------ATCAAAACTTTAATTTACAAGTACATCCTAGAAACAACACAAACGGGACACACGTAAATCCCTGGATATGCGAAGAACCAAAGCACGAATGGGACACTTC------------TCATAAACCGACCAATTATGAAGACAATACAGCCACATCATCTATAGATCATTTATACCGCTATAACAATCATTCTAACACATCACACGGCAGACGCACTACGTGGACGTTAGCATTAATTTGTGTAGCCTGCATTCTCCTATTTTTCGTCCGACGAGCTCTAAATAAAAAATATCATCCATTAAGTGACGATATAAGTGAATCAGAATTCATAGTTCGATACAATCCTGAGCACGAGGACTAA

HAN ATGTGTACTCAACATCGACGGCGAAGCAAGTCATCATATACGCAAATAATATGCATGTTTATTATATTTTGGATTCTGCAGAAAGGCAAGTGTAACAACACGAC---TGCTAATACTTCCATGTCTACAGCACTTACAAGCTTG---------ATACCCACTACACAACTGTCATCTACTTTAGAAACCACCGGAAT---GTCTACCACTACATTTACAT------CTTCCAC---------CAAT------------GCATCCACAGGATTCACCACAAATTCTGTAAAAAGCACAGATGGGAGCTCTACTATTTCTACCAGACCTATTCAAACATCTACAACCAACGCAACTGTAATGACAACCTCACCAAACAGCGA---CATTAATTCATCGACGCAACATGTAACCCATAGTACTGTGACTTTGCAAACGACATCACTAT---CAACCAACAC------TAC------TATCAT---GGTA------AATGTAACT------GAAAACGTCAGCACACCGCTTCCAATTTGCTCGTCAT---CTAATAACACACTCAACGATACATCAAATAACCATACTTGT------------TATCATGAAAATAATACAATATCAAAAGAACCTGAAATATTATTGTATGCGGTACAAGGAGACAATATTACTATAACACACAATTTAACCACCACATCGTGCTACAAACAAGCTTGGCTTAGACATTTTAATGTATCCACACACGGAAAATACACCCATCCCGGTATAAGAAATGGAAA---ATATCATAACCATTCATTGAAAATCCTGCATTCGCGTGTACTATGTGAGTGGCAAACAAATTATCTAAAACATCACTATGATTTATGTTTTACATGCGATCAGAATTTATCTTTATCTTTGTACGGTCTTAACTTTACTCATTCTGGTAAATATAGCTTTCGATGTTACATAAATGGGCATCCCTCTGAACAAA------------ATCAAAACTTTAATTTACAAGTACATCCTAGAAACAACACAAACGGGACACACGTAAATCCCTGGATATGCGAAGAACCAAAGCACGAATGGGACACTTC------------TCATAAACCGACCAGTCACAAAGATGATACAACC---ACATCTACCAATCATTTATACCGCTACAACAATCATTCTAACACATCGCACGGCAGACACACTACGTGGACGTTAGCGTTAATTTGTGTAGCCTGCATTCTCCTATTTTTCGTCCGACGAGCTCTAAATAAAAAATATCATCCATTAAGTGACGATATAAGTGAATCAGAATTCATAGTTCGATACAATCCTGAGCACGAAGACTAA

BE/30/2010 ATGCGTACTCAACATCGACAGCGAAGCAAGTCATCATATACGCAGATAATATGCATGTTTATTATACTTTGGATTCTGCAGAAAGGCAAGTGTAACAACACGAC---TGCTAATACTTCCATGTCTACAGCACTTACAAGCTTG---------ATACCTACTACACAACTGTCATCTACTTTAGAAACCACCGGAAT---GTCTACCACTACATTTACAT------CTTCCAC---------CAAT------------ACATCCACAGGATTCACCACAAATTCTGTAAAAAGCACAGATGGGAGCTCTACTATTTCTACCAGACCTATTCAAACATCTACAACCAACGCAACTGTAATAACAACCTCACCAAACAGCGA---CATTAATTCATCGACGCAACATGTAACCCATAGTACTGTGACTTTGCAAACGACATCACTAT---CAACCAACAC------TAC------TATCAT---GGTA------AATGTAACT------GAAAACGTCAGCACACCGCTTCCAATTTGCTCGTCAT---CTAATAACACATTCAACGATACATCAAATAACCATACTTGT------------TATCATGAAAATAATACAATATCAAAAGAACCTGAAACATTATTGTATGCGGTACAAGGAGACAATATTACTATAACACACAATTTAACCACCACATCGTGCTACAAACAAGCTTGGCTTAGACATTTTAATGTATCCACACACGGAAAATACACCCATCCCGGTATAAGAAATGGAAA---ATATCATAACCATTCATTGAAAATCCTGCATTCGCGTGTACTATGTGAGTGGCAAACAAATTATCTAAAACATCACTATGATTTATGTTTTACATGCGATCAGAATTTATCTTTATCTTTGTACGGTCTTAACTTTACTCATTCTGGTAAATATAGCTTTCGATGTTACATAAATGGGTATCCCTCTGAACAAA------------ATCAAAACTTTAATTTACAAGTACATCCTAGAAACAACACAAACGGGACACACGTAAATCCCTGGATATGCGAAGAACCAAAGCACGAATGGGACACTTC------------TCATAAACCGACCAGTCACAAAGATGATACAACC---ACATCTACCAATCATTTATACCGCTATAACAATCATTCTAACACATCACACGGCAGACGCACTACGTGGACGTTAGCGTTAATTTGTGTAGCCTGCATTCTCCTATTTTTCGTCCGACGAGCTCTAAATAAAAAATATCATCCATTAAGTGACGATATAAGTGAATCAGAATTCATAGTTCGATACAATCCTGAGCACGAGGACTAA

BE/35/2011 ATGCGTACTCAACATCGACGGCGAAGCAAGTCATCATATACGCAAATAATATGCATGTTTATTATATTTTGGATTCTGCAGAAAGGCAAGTGTAACAACACGAC---TGCTAATACTTCCATGTCTACAGCACTTACAAGCTTG---------ATACCTACTACACAACTGTCATCTACTTTAGGAACCACCGGAAT---GTCCACCACTACATTTACAT------CTTCCAC---------CAAT------------ACATCCACAGGATTCACCACAAATTCTGTAAAAAGCACAGATGGGAGCTCTACTATTTCTACCAGACCTATTCAAACATCTACAACCAACGCAACTGTAATGACAACCTCACCAAACAGCGA---CATTAATTTATCGACGCAACATGTAACCCATAGTACTGTGACTTTGCAAACGACATCACTAT---CAACCAACAC------TAC------TATCAT---GGTA------AATGTAACT------GAAAACGTCAGCACACCGCTTCCAATTTGCTCGTCAT---CTAATAACACATTCAACGATACATCAAATAACCATACTTGT------------TATCATGAAAATAATACAATATCAAAAGAACCTGAAACATTATTGTATGCGGTACAAGGAGACAATATTACTATAACACACAATTTAACCACCACATCGTGCTACAAACAAGCTTGGCTTAGACATTTTAATGTATCCACACACGGAAAATACACCCATCCCGGTATAAGAAATGGAAA---ATATCATAACCATTCATTGAAAATCCTGCATTCGCGTGTACTATGTGAGTGGCAAACAAATTATCTAAAGCATCACTATGATTTATGTTTTACATGCGATCAGAATTTATCTTTATCTTTGTACGGTCTTAACTTTACTCATTCTGGTAAATATAGCTTTCGATGTTACATAAATGGGTATCCCTCTGAACAAA------------ATCAAAACTTTAATTTACAAGTACATCCTAGAAACAACACAAACGGGACACACGTAAATCCCTGGATATGCGAAGAACCAAAGCACGAATGGGACACTTC------------TCATAGACCGACCAGTCACAAAGATGATGCAATC---ACATCTACCAATCATTTATACCGCTATAACAATCATTCTAACACATCACACGGCAGACGCACTACGTGGACGTTAGCATTAATTTGTGTAGCCTGCATTCTCCTATTTTTCGTCCGACGAGCTCTAAATAAAAAATATCATCCATTAAGTGACGATATAAGTGAATCAGAATTCATAGTTCGATACACTCCTGAGCACGAGGACTAA

JER2002 ATGCGTACTCAACATCGACGGCGAAGCAAGTCATCATATACGCAAATAATATGCATGTTTATTATATTTTGGATTCTGCAGAAAGGCAAGTGTAACAACACGAC---TGCTAATACTTCCATGTCTACAGCACTTACAAGCTTG---------ATACCTACTACACAACTGTCATCTACTTTAGAAACCACCGGAAT---GTCCACCACTACATTTACAT------CTTCCAC---------CAAT------------ACATCCACAGGATTCACCACAAATTCTGTAAAAAGCACAGATGGGAGCTCTACTATTTCTACCAGACCTATTCAAACATCTACAACCAACGCAACTGTAATGACAACCTCACCAAACAGCGA---CATTAATTCATCGACGCAGCATGTAACCCATAGTACTGTGACTTTGCAAACGACATCACTAT---CAACCAACAC------TAC------TATCAT---GGTA------AATGTAACT------GAAAACGTCAGCACACCGCTTCCAATTTGCTCGTCAT---CTAATAACACATTCAACGATACATCAAATAACCATACTTGT------------TATCATGAAAATAATACAATATCAAAAGAACCTGAAACATTATTGTATGCGGTACAAGGAGACAATATTACTATAACACACAATTTAACCACCACATCGTGCTACAAACAAGCTTGGCTTAGACATTTTAATGTATCCACACACGGAAAATACACCCATCCCGGTATAAGAAATGGAAA---ATATCATAACCATTCATTGAAAATCCTGCATTCGCGTGTACTATGTGAGTGGCAAACAAATTATCTAAAGCATCACTATGATTTATGTTTTACATGCGATCAGAATTTATCTTTATCTTTGTACGGTCTTAACTTTACTCATTCTGGTAAATATAGCTTTCGATGTTACATAAATGGGTATCCCTCTGAACAAA------------ATCAAAACTTTAATTTACAAGTACATCCTAGAAACAACACAAACGGGACACACGTAAATCCCTGGATATGCGAAGAACCAAAGCACGAATGGGACACTTC------------TCATAGACCGACCAGTCACAAAGATGATGCAATC---ACATCTACCAATCATTTATACCGCTATAACAATCATTCTAACACATCACACGGCAGACACACTACGTGGACGTTAGCATTAATTTGTGTAGCCTGCATTCTCCTATTTTTCGTCCGACGAGCTCTAAATAAAAAATATCATCCATTAAGTGACGATATAAGTGAATCAGAATTCATAGTTCGATACACTCCTGAGCACGAGGACTAA

PAV18 ATGCGTACTCAACATCGACGGCGAAGCAAGTCATCATATACGCAAATAATATGCATGTTTATTATATTTTGGATTCTGCAGAAAGGCAAGTGTAACAACACGAC---TGCTAATACTTCCATGTCTACAGCACTTACAAGCTTG---------ATACCTACTACACAACTGTCATCTACTTTAGAAACCACCGGAAT---GTCCACCACTACATTTACAT------CTTCCAC---------CAAT------------ACATCCACAGGATTCACCACAAATTCTGTAAAAAGCACAGATGGGAGCTCTACTATTTCTACCAGACCTATTCAAACATCTACAACCAACGCAACTGTAATGACAACCTCACCAAACAGCGA---CATTAATTCATCGACGCAACATGTAACCCATAGTACTGTGACTTTGCAAACGACATCACTAT---CAACCAACAC------TAC------TATCAT---GGTA------AATGTAACT------GAAAACGTCAGCACACCGCTTCCAATTTGCTCGTCAT---CTAATAACACATTCAACGATACATCAAATAACCATACTTGT------------TATCATGAAAATAATACAATATCAAAAGAACCTGAAACATTATTGTATGCGGTACAAGGAGACAATATTACTATAACACACAATTTAACCACCACATCGTGCTACAAACAAGCTTGGCTTAGACATTTTAATGTATCCACACACGGAAAATACACCCATCCCGGTATAAGAAATGGAAA---ATATCATAACCATTCATTGAAAATCCTGCATTCGCGTGTACTATGTGAGTGGCAAACAAATTATCTAAAGCATCACTATGATTTATGTTTTACATGCGATCAGAATTTATCTTTATCTTTGTACGGTCTTAACTTTACTCATTCTGGTAAATATAGCTTTCGATGTTACATAAATGGGTATCCCTCTGAACAAA------------ATCAAAACTTTAATTTACAAGTACATCCTAGAAACAACACAAACGGGACACACGTAAATCCCTGGATATGCGAAGAACCAAAGCACGAATGGGACACTTC------------TCATAGACCGACCAGTCACAAAGATGATGCAATC---ACATCTACCAATCATTTATACCGCTATAACAATCATTCTAACACATCACACGGCAGACACACTACGTGGACGTTAGCATTAATTTGTGTAGCCTGCATTCTCCTATTTTTCGTCCGACGAGCTCTAAATAAAAAATATCATCCATTAAGTGACGATATAAGTGAATCAGAATTCATAGTTCGATACACTCCTGAGCACGAGGACTAA

PAV1 ATGCGTACTCAACATCGACGGCGAAGCAAGTCATCATATACGCAAATAATATGCATGTTTATTATATTTTGGATTCTGCAGAAAGGCAAGTGTAACAACACGAC---TGCTAATACTTCCATGTCTACAGCACTTACAAGCTTG---------ATACCTACTACACAACTGTCATCTACTTTAGAAACCACCGGAAT---GTCCACCACTACATTTACAT------CTTCCAC---------CAAT------------ACATCCACAGGATTCACCACAAATTCTGTAAAAAGCACAGATGGGAGCTCTACTATTTCTACCAGACCTATTCAAACATCTACAACCAACGCAACTGTAATGACAACCTCACCAAACAGCGA---CATTAATTCATCGACGCAACATGTAACCCATAGTACTGTGACTTTGCAAACGACATCACTAT---CAACCAACAC------TAC------TATCAT---GGTA------AATGTAACT------GAAAACGTCAGCACACCGCTTCCAATTTGCTCGTCAT---CTAATAACACATTCAACGATACATCAAATAACCATACTTGT------------TATCATGAAAATAATACAATATCAAAAGAACCTGAAACATTATTGTATGCGGTACAAGGAGACAATATTACTATAACACACAATTTAACCACCACATCGTGCTACAAACAAGCTTGGCTTAGACATTTTAATGTATCCACACACGGAAAATACACCCATCCCGGTATAAGAAATGGAAA---ATATCATAACCATTCATTGAAAATCCTGCATTCGCGTGTACTATGTGAGTGGCAAACAAATTATCTAAAGCATCACTATGATTTATGTTTTACATGCGATCAGAATTTATCTTTATCTTTGTACGGTCTTAACTTTACTCATTCTGGTAAATATAGCTTTCGATGTTACATAAATGGGTATCCCTCTGAACAAA------------ATCAAAACTTTAATTTACAAGTACATCCTAGAAACAACACAAACGGGACACACGTAAATCCCTGGATATGCGAAGAACCAAAGCACGAATGGGACACTTC------------TCATAGACCGACCAGTCACAAAGATGATGCAATC---ACATCTACCAATCATTTATACCGCTATAACAATCATTCTAACACATCACACGGCAGACACACTACGTGGACGTTAGCATTAATTTGTGTAGCCTGCATTCTCCTATTTTTCGTCCGACGAGCTCTAAATAAAAAATATCATCCATTAAGTGACGATATAAGTGAATCAGAATTCATAGTTCGATACACTCCTGAGCACGAGGACTAA

PAV21 ATGCGTACTCAACATCGACGGCGAAGCAAGTCATCATATACGCAAATAATATGCATGTTTATTATATTTTGGATTCTGCAGAAAGGCAAGTGTAACAACACGAC---TGCTAATACTTCCATGTCTACAGCACTTACAAGCTTG---------ATACCTACTACACAACTGTCATCTACTTTAGAAACCACCGGAAT---GTCCACCACTACATTTACAT------CTTCCAC---------CAAT------------ACATCCACAGGATTCACCACAAATTCTGTAAAAAGCACAGATGGGAGCTCTACTATTTCTACCAGACCTATTCAAACATCTACAACCAACGCAACTGTAATGACAACCTCACCAAACAGCGA---CATTAATTCATCGACGCAACATGTAACCCATAGTACTGTGACTTTGCAAACGACATCACTAT---CAACCAACAC------TAC------TATCAT---GGTA------AATGTAACT------GAAAACGTCAGCACACCGCTTCCAATTTGCTCGTCAT---CTAATAACACATTCAACGATACATCAAATAACCATACTTGT------------TATCATGAAAATAATACAATATCAAAAGAACCTGAAACATTATTGTATGCGGTACAAGGAGACAATATTACTATAACACACAATTTAACCACCACATCGTGCTACAAACAAGCTTGGCTTAGACATTTTAATGTATCCACACACGGAAAATACACCCATCCCGGTATAAGAAATGGAAA---ATATCATAACCATTCATTGAAAATCCTGCATTCGCGTGTACTATGTGAGTGGCAAACAAATTATCTAAAGCATCACTATGATTTATGTTTTACATGCGATCAGAATTTATCTTTATCTTTGTACGGTCTTAACTTTACTCATTCTGGTAAATATAGCTTTCGATGTTACATAAATGGGTATCCCTCTGAACAAA------------ATCAAAACTTTAATTTACAAGTACATCCTAGAAACAACACAAACGGGACACACGTAAATCCCTGGATATGCGAAGAACCAAAGCACGAATGGGACACTTC------------TCATAGACCGACCAGTCACAAAGATGATGCAATC---ACATCTACCAATCATTTATACCGCTATAACAATCATTCTAACACATCACACGGCAGACACACTACGTGGACGTTAGCATTAATTTGTGTAGCCTGCATTCTCCTATTTTTCGTCCGACGAGCTCTAAATAAAAAATATCATCCATTAAGTGACGATATAAGTGAATCAGAATTCATAGTTCGATACACTCCTGAGCACGAGGACTAA

HAN21 ATGCGTACTCAACATCGACGGCGAAGCAAGTCATCATATACGCAAATAATATGCATGTTTATTATATTTTGGATTCTGCAGAAAGGCAAGTGTAACAACACGAC---TGCTAATACTTCCATGTCTACAGCACTTACAAGCTTG---------ATACCTACTACACAACTGTCATCTACTTTAGAAACCACCGGAAT---GTCCACCACTACATTTACAT------CTTCCAC---------CAAT------------ACATCCACAGGATTCACCACAAATTCTGTAAAAAGCACAGATGGGAGCTCTACTATTTCTACCAGACCTATTCAAACATCTACAACCAACGCAACTGTAATGACAACCTCACCAAACAGCGA---CATTAATTCATCGACGCAACATGTAACCCATAGTACTGTGACTTTGCAAACGACATCACTAT---CAACCAACAC------TAC------TATCAT---GGTA------AATGTAACT------GAAAACGTCAGCACACCGCTTCCAATTTGCTCGTCAT---CTAATAACACATTCAACGATACATCAAATAACCATACTTGT------------TATCATGAAAATAATACAATATCAAAAGAACCTGAAACATTATTGTATGCGGTACAAGGAGACAATATTACTATAACACACAATTTAACCACCACATCGTGCTACAAACAAGCTTGGCTTAGACATTTTAATGTATCCACACACGGAAAATACACCCATCCCGGTATAAGAAATGGAAA---ATATCATAACCATTCATTGAAAATCCTGCATTCGCGTGTACTATGTGAGTGGCAAACAAATTATCTAAAGCATCACTATGATTTATGTTTTACATGCGATCAGAATTTATCTTTATCTTTGTACGGTCTTAACTTTACTCATTCTGGTAAATATAGCTTTCGATGTTACATAAATGGGTATCCCTCTGAACAAA------------ATCAAAACTTTAATTTACAAGTACATCCTAGAAACAACACAAACGGGACACACGTAAATCCCTGGATATGCGAAGAACCAAAGCACGAATGGGACACTTC------------TCATAGACCGACCAGTCACAAAGATAATGCAATC---ACATCTACCAATCATTTATACCGCTATAACAATCATTCTAACACATCACACGGCAGACACACTACGTGGACGTTAGCATTAATTTGTGTAGCCTGCATTCTCCTATTTTTCGTCCGACGAGCTCTAAATAAAAAATATCATCCATTAAGTGACGATATAAGTGAATCAGAATTCATAGTTCGATACACTCCTGAGCACGAGGACTAA

BE/19/2010 ATGCGTACTCAACATCGACGGCGAAGCAAGTCATCATATACGCAAATAATATGCATGTTTATTATATTTTGGATTCTGCAGAAAGGCAAGTGTAACAACACGAC---TGCTAATACTTCCATGTCTACAGCACTTACAAGCTTG---------ATACCTACTACACAACTGTCATCTACTTTAGAAACCACCGGAAT---GTCTACCACTACATTTACAT------CTTCCAC---------CAAT------------ACATCCACAGGATTCACCACAAATTCTGTAAAAAGCACAGATGGGAGCTCTACTATTTCTACCAGACCTATTCAAACATCTACAACCAACGCAACTGTAATGACAACCTCACCAAACAGCGA---CATTAATTCATCGACGCAACATGTAACCCATAGTACTGTGACTTTGCAAACGACATCACTAT---CAACCAACAC------TAC------TATCAT---GGTA------AATGTAACT------GAAAACGTCAGCACACCGCTTCCAATTTGCTCGTCAT---CTAATAACACATTCAACGATACATCAAATAACCATACTTGT------------TATCATGAAAATAATACAATATCAAAAGAACCTGAAACATTATTGTATGCGGTACAAGGAGACAATATTACTATAACACACAATTTAACCACCACATCGTGCTACAAACAAGCTTGGCTTAGACATTTTAATGTATCTACACACGGAAAATACACCCATCCCGGTATAAGAAATGGAAA---ATA---TAACCATTCATTGAAAATCCTGCATTCGCGTGTACTATGTGAGTGGCAAACAAATTATCTAAAGCATCACTATGATTTATGTTTTACATGCGATCAGAATTTATCTTTATCTTTGTACGGTCTTAACTTTACTCATTCTGGTAAATATAGCTTTCGATGTTACATAAATGGGTATCCCTCTGAACAAA------------ATCAAAACTTTAATTTACAAGTACATCCTAGAAACAACACAAACGGGACACACGTAAATCCCTGGATATGCGAAGAACCAAAGCACGAATGGGACACTTC------------TCATAGACCGACCAGTCACAAAGATGATGCAATC---ACATCTACCAATCATTTATACCGCTATAACAATCATTCTAACACATCACACGGCAGACGCACTACGTGGACGTTAGCATTAATTTGTGTAGCCTGCATTCTCCTATTTTTCGTCCGACGAGCTCTAAATAAAAAATATCATCCATTAAGTGACGATATAAGTGAATCAGAATTCATAGTTCGATACACTCCTGAGCACGAGGACTAA

JER3855 ATGCGTACTCAACATCGACGGCGAAGCAAGTCATCATATACGCAAATAATATGCATGTTTATTATATTTTGGATTCTGCAGAAAGGCAAGTGTAACAACACGAC---TGCTAATACTTCCATGTCTACAGCACTTACAAGCTTG---------ATACCTACTACACAACTGTCATCTACTTTAGAAACCACCGGAAT---GTCTACCACTACATTTACAT------CTTCCAC---------CAAT------------ACATCCACAGGATTCACCACAAATTCTGTAAAAAGCACAGATGGGAGCTCTACTATTTCTACCAGACCTATTCAAACATCTACAACCAACGCAACTGTAATGACAACCTCACCAAACAGCGA---CATTAATTCATCGACGCAACATGTAATCCATAGTACTGTGACTTTGCAAACGACATCACTAT---CAACCAACAC------TAC------TATCAT---GGTA------AATGTAACT------GAAAACGTCAGCACACCGCTTCCAATTTGCTCGTCAT---CTAATAACACATTCAACGATACATCAAATAACCATACTTGT------------TATCATGAAAATAATACAATATCAAAAGAACCTGAAACATTATTGTATGCGGTACAAGGAGACAATATTACTATAACACACAATTTAACCACCACATCGTGCTACAAACAAGCTTGGCTTAGACATTTTAATGTATCTACACACGGAAAATACACCCATCCCGGTATAAGAAATGGAAA---ATATCATAACCATTCATTGAAAATCCTGCATTCGCGTGTACTATGTGAGTGGCAAACAAATTATCTAAAGCATCACTATGATTTATGTTTTACATGCGATCAGAATTTATCTTTATCTTTGTACGGTCTTAACTTTACTCATTCTGGCAAATATAGCTTTCGATGTTACATAAATGGGTATCCCTCTGAACAAA------------ATCAAAACTTTAATTTACAAGTACATCCTAGAAACAACACAAACGGGACACACGTAAATCCCTGGATATGCGAAGAACCAAAGCACGAATGGGACACTTC------------TCATAGACCGACCAGTCACAAAGATGATGCAATC---ACATCTACCAATCATTTATACCGCTATAACAATCATTCTAACACATCACACGGCGGACGCACTACGTGGACGTTAGCATTAATTTGTGTAGCCTGCATTCTCCTATTTTTCGTCCGACGAGCTCTAAATAAAAAATATCATCCATTAAGTGACGATATAAGTGAATCAGAATTCATAGTTCGATACACTCCTGAGCACGAGGACTAA

BE/31/2011 ATGCGTACTCAACATCGACGGCGAAGCAAGTCATCATATACGCAAATAATATGCATGTTTATTATATTTTGGATTCTGCAGAAAGGCAAGTGTAACAACACGAC---TGCTAATACTTCCATGTCTACAGCACTTACAAGCTTG---------ATACCTACTACACAACTGTCATCTACTTTAGAAACCACCGGAAT---GTCTACCACTACATTTACAT------CTTCCAC---------CAAT------------ACATCCACAGGATTCACCACAAATTCTGTAAAAAGCACAGATGGGAGCTCTACTATTTCTACCAGACCTATTCAAACATCTACAACCAACGCAACTGTAATGACAACCTCACCAAACAGCGA---CATTAATTCATCGACGCAACATGTAACCCATAGTACTGTGACTTTGCAAACGACATCACTAT---CAACCAACAC------TAC------TATCAT---GGTA------AATGTAACT------GAAAACGTCAGCACACCGCTTCCAATTTGCTCGTCAT---CTAATAACACATTCAACGATACATCAAATAACCATACTTGT------------TATCATGAAAATAATACAATATCAAAAGAACCTGAAACATTATTGTATGCGGTACAAGGAGACAATATTACTATAACACACAATTTAACCACCACATCGTGCTACAAACAAGCTTGGCTTAGACATTTTAATGTATCTACACACGGAAAATACACCCATCCCGGTATAAGAAATGGAAA---ATATCATAACCATTCATTGAAAATCCTGCATTCGCGTGTACTATGTGAGTGGCAAACAAATTATCTAAAGCATCACTATGATTTATGTTTTACATGCGATCAGAATTTATCTTTATCTTTGTACGGTCTTAACTTTACTCATTCTGGTAAATATAGCTTTCGATGTTACATAAATGGGTATCCCTCTGAACAAA------------ATCAAAACTTTAATTTACAAGTACATCCTAGAAACAACACAAACGGGACACACGTAAATCCCTGGATATGCGAAGAACCAAAGCACGAATGGGACACTTC------------TCATAGACCGACCAGTCACAAAGATGATGCAATC---ACATCTACCAATCATTTATACCGCTATAACAATCATTCTAACACATCACACGGCAGACGCACTACGTGGACGTTAGCATTAATTTGTGTAGCCTGCATTCTCCTATTTTTCGTCCGACGAGCTCTAAATAAAAAATATCATCCATTAAGTGACGATATAAGTGAATCAGAATTCATAGTTCGATACACTCCTGAGCACGAGGACTAA

BE/3/2011 ATGCGTACTCAACATCGACGGCGAAGCAAGTCATCATATACGCAAATAATATGCATGTTTATTATATTTTGGATTCTGCAGAAAGGCAAGTGTAACAACACGAC---TGCTAATACTTCCATGTCTACAGCACTTACAAGCTTG---------ATACCTACTACACAACTGTCATCTACTTTAGAAACCACCGGAAT---GTCTACCACTACATTTACAT------CTTCCAC---------CAAT------------ACATCCACAGGATTCACCACAAATTCTGTAAAAAGCACAGATGGGAGCTCTACTATTTCTACCAGACCTATTCAAACATCTACAACCAACGCAACTGTAATGACAACCTCACCAAACAGCGA---CATTAATTCATCGACGCAACATGTAACCCATAGTACTGTGACTTTGCAAACGACATCACTAT---CAACCAACAC------TAC------TATCAT---GGTA------AATGTAACT------GAAAACGTCAGCACACCGCTTCCAATTTGCTCGTCAT---CTAATAACACATTCAACGATACATCAAATAACCATACTTGT------------TATCATGAAAATAATACAATATCAAAAGAACCTGAAACATTATTGTATGCGGTACAAGGAGACAATATTACTATAACACACAATTTAACCACCACATCGTGCTACAAACAAGCTTGGCTTAGACATTTTAATGTATCTACACACGGAAAATACACCCATCCCGGTATAAGAAATGGAAA---ATATCATAACCATTCATTGAAAATCCTGCATTCGCGTGTACTATGTGAGTGGCAAACAAATTATCTAAAGCATCACTATGATTTATGTTTTACATGCGATCAGAATTTATCTTTATCTTTGTACGGTCTTAACTTTACTCATTCTGGTAAATATAGCTTTCGATGTTACATAAATGGGTATCCCTCTGAACAAA------------ATCAAAACTTTAATTTACAAGTACATCCTAGAAACAACACAAACGGGACACACGTAAATCCCTGGATATGCGAAGAACCAAAGCACGAATGGGACACTTC------------TCATAGACCGACCAGTCACAAAGATGATGCAATC---ACATCTACCAATCATTTATACCGCTATAACAATCATTCTAACACATCACACGGCAGACGCACTACGTGGACGTTAGCATTAATTTGTGTAGCCTGCATTCTCCTATTTTTCGTCCGACGAGCTCTAAATAAAAAATATCATCCATTAAGTGACGATATAAGTGAATCAGAATTCATAGTTCGATACACTCCTGAGCACGAGGACTAA

BE/21/2011 ATGCGTACTCAACATCGACGGCGAAGCAAGTCATCATATACGCAAATAATATGCATGTTTATTATATTTTGGATTCTGCAGAAAGGCAAGTGTAACAACACGAC---TGCTAATACTTCCATGTCTACAGCACTTACAAGCTTG---------ATACCTACTACACAACTGTCATCTACTTTAGAAACCACCGGAAT---GTCTACCACTACATTTACAT------CTTCCAC---------CAAT------------ACATCCACAGGATTCACCACAAATTCTGTAAAAAGCACAGATGGGAGCTCTACTATTTCTACCAGACCTATTCAAACATCTACAACCAACGCAACTGTAATGACAACCTCACCAAACAGCGA---CATTAATTCATCGACGCAACATGTAACCCATAGTACTGTGACTTTGCAAACGACATCACTAT---CAACCAACAC------TAC------TATCAT---GGTA------AATGTAACT------GAAAACGTCAGCACACCGCTTCCAATTTGCTCGTCAT---CTAATAACACATTCAACGATACATCAAATAACCATACTTGT------------TATCATGAAAATAATACAATATCAAAAGAACCTGAAACATTATTGTATGCGGTACAAGGAGACAATATTACTATAACACACAATTTAACCACCACATCGTGCTACAAACAAGCTTGGCTTAGACATTTTAATGTATCTACACACGGAAAATACACCCATCCCGGTATAAGAAATGGAAA---ATATCATAACCATTCATTGAAAATCCTGCATTCGCGTGTACTATGTGAGTGGCAAACAAATTATCTAAAGCATCACTATGATTTATGTTTTACATGCGATCAGAATTTATCTTTATCTTTGTACGGTCTTAACTTTACTCATTCTGGTAAATATAGCTTTCGATGTTACATAAATGGGTATCCCTCTGAACAAA------------ATCAAAACTTTAATTTACAAGTACATCCTAGAAACAACACAAACGGGACACACGTAAATCCCTGGATATGCGAAGAACCAAAGCACGAATGGGACACTTC------------TCATAGACCGACCAGTCACAAAGATGATGCAATC---ACATCTACCAATCATTTATACCGCTATAACAATCATTCTAACACATCACACGGCAGACGCACTACGTGGACGTTAGCATTAATTTGTGTAGCCTGCATTCTCCTATTTTTCGTCCGACGAGCTCTAAATAAAAAATATCATCCATTAAGTGACGATATAAGTGAATCAGAATTCATAGTTCGATACACTCCTGAGCACGAGGACTAA

NL/Rot3 G3 ATGCGTACACAACATCGACGGTGGAACAAGTCATCATATACGCAAATAATATGCATGTTTATTATTTTTTGGATTCTGCAGAAAAGCAAGTGTAACAACACCACTATCGCTAATACTTCCACGCCGATTACGCCCACAAGCTTG---------ATATCTACTACACAGTTAGCATCTACCAACA---------------------------CATTTACAT------CCTCCAACGTTACCGAAAAT------------ACATCCACAGAAGCCATTACAAGTTCCGTAAAAAGCACAGATGTAAG---TTCTATTTCCACCACACCTACCACAACATCGATGACTAATGCAACTGTAATGACAATCTCACCAAACGGCGG---CATAAATTCATCGATACAACATGTAACTAACAATACCGTGACTTTGCAAACAACTATATCAA---CCAACACTAC------TAT------CATAAT------------------AAATACAACTG---AAAACAGTACGCCGTTCTCAAATTGCTCGTCAC---CTAAT------------------------------------------------AG---CACATTGAGTACGATGTTACAAGAATCTGAAACACTATTGAACGCGGCACAAGGAGAGAATATCACTATAAAACACAACCTAACCATAACATCGTGCTACAAAACAGCTTGGCTTAGACATTTTAATATATNNNNNNNNNNNNNNNNNNNNNNNNNNNNNNNNNNNNNNNNNNN---NNATTATAACTATTCATTAAAAATTCTTCATTCGCGTACACTATGTGAGTCTCAAACACACTATTTAAAACATCATTATGATTTATGTTTTACATGTGATCACAATTTTTCTTTGTCTCTGTACGGTCTCAATTTTACTCATTCTGGGAAATATAGCTTTCGATGTTACAAGTATGATCATCCCTCTGAACAAA------------ATCAAAACTTTAATCTACAAGTACATCCTAGAAATGACACAAACGGGACAGATGTGAATCCATGGATATGCGAAGAACCAAAGCACGAATGGAAGACTTTGGCTGCCACGTCTCGTAAACCAACCAGTCATAAAAACTATACAACCACATCATCTACAGATCATCTGTACCGTCATAACAACCATTCTAACACATCACACAGCAGACGCACTACATGGACTTTAGTATTAATCTGTATAGCCTGCATTCTCCTGTTTTTCGTCCGAAGAGCTTTAAACAAAAAATATCATCCACTGAGGGACGATATAAGTGAATCAGAATTTATAGTTCGCTACAATCCAGAGCACGAGGACTAA

CZ/2/2013 ATGCGTACACAACATCGACGGTGGAACAAGTCATCATATACGCAAATAATATGCATGTTTATTATTTTTTGGATTCTGCAGAAAAGCAAGTGTAACAACACCACTATCGCTAATACTTCCACGCCGATTACGCCCACAAGCTTG---------ATATCTACTACACAGTTAGCATCTACCAACA---------------------------CATTTACAT------CCTCCAACGTTACCGAAAAT------------ACATCCACAGAAGCCATTACAAGTTCCGTAAAAAGCACAGATGTGAG---TTCTATTTCCACCACACCTACCACAACATCGATGACTAATGCAACTGTAATGACAATCTCACCAAACGGCGG---CATAAATTCATCGATACAACATGTAACTAACAATACCGTGACTTTGCAAACAAATATATCAA---CCAACACTAC------TAT------CATAAT------------------AAATACAACTG---AAAACAGTACGCCGTTCTCAAATTGCTCGTCAC---CTAAT------------------------------------------------AG---CACATTGAGTACGATGTTACAAGAATCTGAAACACTATTGAACGCGGCACAAGGAGAGAATATCACTATAAAACACAACCTAACCATAACATCGTGCTACAAAACAGCTTGGCTTAGACATTTTAATATATCCACACATGGAAAATACACCCATTCAAAAATAAAAAATGGAAG---ATATTATAACTATTCATTAAAAATTCTTCATTCGCGTACACTATGTGAGTCTCAAACACACTATTTAAAACATCATTATGATTTATGTTTTACATGTGATCACAATTTTTCTTTGTCTCTGTACGGTCTCAATTTTACTCATTCTGGGAAATATAGCTTTCGATGTTACAAGTATGATCATCCCTCTGAACAAA------------ATCAAAACTTTAATCTACAAGTACATCCTAGAAATGACACAAACGGGACAGATGTGAATCCATGGATATGCGAAGAACCAAAGCACGAATGGAAGACTTTGGCTGCCACGTCTCGTAAACCAACCAGTCATAAAAACTATACAACCACATCATCTACAGATCATCTGTACCGTCATAACAACCATTCTAACACATCACACAGCAGACGCACTACATGGACTTTAGTATTAATCTGTATAGCCTGCATTCTCCTGTTTTTCGTCCGAAGAGCTTTAAACAAAAAATATCATCCACTGAGGGACGATATAAGTGAATCAGAATTTATAGTTCGCTACAATCCAGAGCACGAGGACTAA

BE/11/2011 ATGCGTACACAACATCGACGGTGGAACAAGTCATCATATACGCAAATAATATGCATGTTTATTATTTTTTGGATTCTGCAGAAAAGCAAGTGTAACAACACCACTATCGCTAATACTTCCACGCCGATTACGCCCACAAGCTTG---------ATATCTACTACACAGTTAGCATCTACCAACA---------------------------CATTTACAT------CCTCCAACGTTACCGAAAAT------------ACATCCACAGAAGCCATTACAAGTTCCGTAAAAAGCACAGATGTGAG---TTCTATTTCCACCACACCTACCACAACATCGATGACTAATGCAACTGTAATGACAATCTCACCAAACGGCGG---CATAAATTCATCGATACAACATGTAACTAACAATACCGTGACTTTGCAAACAAATATATCAA---CCAACACTAC------TAT------CATAAT------------------AAATACAACTG---AAAACAGTACGCCGTTCTCAAATTGCTCGTCAC---CTAAT------------------------------------------------AG---CACATTGAGTACGATGTTACAAGAATCTGAAACACTATTGAACGCGGCACAAGGAGAGAATATCACTATAAAACACAACCTAACCATAACATCGTGCTACAAAACAGCTTGGCTTAGACATTTTAATATATCCACACATGGAAAATACACCCATTCAAAAATAAAAAATGGAAG---ATATTATAACTATTCATTAAAAATTCTTCATTCGCGTACACTATGTGAGTCTCAAACACACTATTTAAAACATCATTATGATTTATGTTTTACATGTGATCACAATTTTTCTTTGTCTCTGTACGGTCTCAATTTTACTCATTCTGGGAAATATAGCTTTCGATGTTACAAGTATGATCATCCCTCTGAACAAA------------ATCAAAACTTTAATCTACAAGTACATCCTAGAAATGACACAAACGGGACAGATGTGAATCCATGGATATGCGAAGAACCAAAGCACGAATGGAAGACTTTGGCTGCCACGTCTCGTAAACCAACCAGTCATAAAAACTATACAACCACATCATCTACAGATCATCTGTACCGTCATAACAACCATTCTAACACATCACACAGCAGACGCACTACATGGACTTTAGTATTAATCTGTATAGCCTGCATTCTCCTGTTTTTCGTCCGAAGAGCTTTAAACAAAAAATATCATCCACTGAGGGACGATATAAGTGAATCAGAATTTATAGTTCGCTACAATCCAGAGCACGAGGACTAA

HANSCTR10 ATGCGTACACAACATCGACGGTGGAACAAGTCATCATATACGCAAATAATATGCATGTTTATTATTTTTTGGATTCTGCAGAAAAGCAAGTGTAACAACACCACTATCGCTAATACTTCCACGCCGATTACGCCCACAAGCTTG---------ATATCTACTACACAGTTAGCATCTACCAACA---------------------------CATTTACAT------CCTCCAACGTTACCGAAAAT------------ACATCCACAGAAGCCATTACAAGTTCCGTAAAAAGCACAGATGTGAG---TTCTATTTCCACCACACCTACCACAACATCGATGACTAATGCAACTGTAATGACAATCTCACCAAACGGCGG---CATAAATTCATCGATACAACATGTAACTAACAATACCGTGACTTTGCAAACAAATATATCAA---CCAACACTAC------TAT------CATAAT------------------AAATACAACTG---AAAACAGTACGCCGTTCTCAAATTGCTCGTCAC---CTAAT------------------------------------------------AG---CACATTGAGTACGATGTTACAAGAATCTGAAACACTATTGAACGCGGCACAAGGAGAGAATATCACTATAAAACACAACCTAACCATAACATCGTGCTACAAAACAGCTTGGCTTAGACATTTTAATATATCCACACATGGAAAATACACCCATTCAAAAATAAAAAATGGAAG---ATATTATAACTATTCATTAAAAATTCTTCATTCGCGTACACTATGTGAGTCTCAAACACACTATTTAAAACATCATTATGATTTATGTTTTACATGTGATCACAATTTTTCTTTGTCTCTGTACGGTCTCAATTTTACTCATTCTGGGAAATATAGCTTTCGATGTTACAAGTATGATCATCCCTCTGAACAAA------------ATCAAAACTTTAATCTACAAGTACATCCTAGAAATGACACAAACGGGACAGATGTGAATCCATGGATATGCGAAGAACCAAAGCACGAATGGAAGACTTTGGCTGCCACGTCTCGTAAACCAACCAGTCATAAAAACTATACAACCACATCATCTACAGATCATCTGTACCGTCATAACAACCATTCTAACACATCACACAGCAGACGCACTACATGGACTTTAGTATTAATCTGTATAGCCTGCATTCTCCTGTTTTTCGTCCGAAGAGCTTTAAACAAAAAATATCATCCACTGAGGGACGATATAAGTGAATCAGAATTTATAGTTCGCTACAATCCAGAGCACGAGGACTAA

PAV32 ATGCGTACACAACATCGACGGTGGAACAAGTCATCATATACGCAAATAATATGCATGTTTATTATTTTTTGGATTCTGCAGAAAAGCAAGTGTAACAACACCACTATCGCTAATACTTCCACGCCGATTACGCCCACAAGCTTG---------ATATCTACTACACAGTTAGCATCTACCAACA---------------------------CATTTACAT------CCTCCAACGTTACCGAAAAT------------ACATCCACAGAAGCCATTACAAGTTCCGTAAAAAGCACAGATGTGAG---TTCTATTTCCACCACACCTACCACAACATCGATGACTAATGCAACTGTAATGACAATCTCACCAAACGGCGG---CATAAATTCATCGATACAACATGTAACTAACAATACCGTGACTTTGCAAACAAATATATCAA---CCAACACTAC------TAT------CATAAT------------------AAATACAACTG---AAAACAGTACGCCGTTCTCAAATTGCTCGTCAC---CTAAT------------------------------------------------AG---CACATTGAGTACGATGTTACAAGAATCTGAAACACTATTGAACGCGGCACAAGGAGAGAATATCACTATAAAACACAACCTAACCATAACATCGTGCTACAAAACAGCTTGGCTTAGACATTTTAATATATCCACACATGGAAAATACACCCATTCAAAAATAAAAAATGGAAG---ATATTATAACTATTCATTAAAAATTCTTCATTCGCGTACACTATGTGAGTCTCAAACACACTATTTAAAACATCATTATGATTTATGTTTTACATGTGATTACAATTTTTCTTTGTCTCTGTACGGTCTCAATTTTACTCATTCTGGGAAATATAGCTTTCGATGTTACAAGTATGATCATCCCTCTGAACAAA------------ATCAAAACTTTAATCTACAAGTACATCCTAGAAATGACACAAACGGGACAGATGTGAATCCATGGATATGCGAAGAACCAAAGCACGAATGGAAGACTTTGGCTGCCACGTCTCGTAAACCAACCAGTCATAAAAACTATACAACCACATCATCTACAGATCATCTGTACCGTCATAACAACCATTCTAACACATCACACAGCAGACGCACTACATGGACTTTAGTATTAATCTGTATAGCCTGCATTCTCCTGTTTTTCGTCCGAAGAGCTTTAAACAAAAAATATCATCCACTGAGGGACGATATAAGTGAATCAGAATTTATAGTTCGCTACAATCCAGAGCACGAGGACTAA

BE/2/2013 ATGCGTACACAACATCGACGGTGGAACAAGTCATCATATACGCAAATAATATGCATGTTTATTATTTTTTGGATTCTGCAGAAAAGCAAGTGTAACAACACCACTATCGCTAATACTTCCACGCCGATTACGCCCACAAGCTTG---------ATATCTACTACACAGTTAGCATCTACCAACA---------------------------CATTTACAT------CCTCCAACGTTACCGAAAAT------------ACATCCACAGAAGCCATTACAAGTTCCGTAAAAAGCACAGATGTAAG---TTCTATTTCCACCACACCTACCACAACATTGATGACTAATGCAACTGTAATGACAATCTCACCAAACGGCGG---CATAAATTCATCGATACAACATGTAACTAACAATACCGTGACTTTGCAAACAACTATATCAA---CCAACACTAC------TAT------CATAAT------------------AAATACAACTG---AAAACAGTACGCCGTTCTCAAATTGCTCGTCAC---CTAAT------------------------------------------------AG---CACATTGAGTACGATGTTACAAGAATCTGAAACACTATTGAACGCGGTACAAGGAGAGAATATCACTATAAAACACAACCTAACCATAACATCGTGCTACAAAACAGCTTGGCTTAGACATTTTAATATATCCACACATGGAAAATACACCCATTCAAAAATAAAAAATGGAAG---ATATTATAACTATTCATTAAAAATTCTTCATTCGCGTACACTATGTGAGTCTCAAACACACTATTTAAAACATCATTATGATTTATGTTTTACATGTGATCACAATTTTTCTTTGTCTCTGTACGGTCTCAATTTTACTCATTCTGGGAAATATAGCTTTCGATGTTACAAGTATGATCATCCCTCTGAACAAA------------ATCAAAACTTTAATCTACAAGTACATCCTAGAAATGACACAAACGGGACAGATGTGAATCCATGGATATGCGAAGAACCAAAGCACGAATGGAAGACTTTGGCTGCCACGTCTCGTAAACCAACCAGTCATAAAAACTATACAACCACATCATCTACAGATCATCTGTACCGTCATAACAACCATTCTAACACATCACACAGCAGACGCACTACATGGACTTTAGTATTAATCTGTATAGCCTGCATTCTCCTGTTTTTCGTCCGAAGAGCTTTAAACAAAAAATATCATCCACTGAGGGACGATATAAGTGAATCAGAATTTATAGTTCGCTACAATCCAGAGCACGAGGACTAA

BE/25/2010 ATGCGTACACAACATCGACGGTGGAACAAGTCATCATATACGCAAATAATATGCATGTTTATTATTTTTTGGATTCTGCAGAAAAGCAAGTGTAACAACACCACTATCGCTAATACTTCCACGCCGATTACGCCCACAAGCTTG---------ATATCTACTACACAGTTAGCATCTACCAACA---------------------------CATTTACAT------CCTCCAACGTTACCGAAAAT------------ACATCCACAGAAGCCATTACAAGTTCCGTAAAAAGCACAGATGTAAG---TTCTATTTCCACCACACCTACCACAACATCGATGACTAATGCAACTGTAATGACAATCTCACCAAACGGCGG---CATAAATTCATCGATACAACATGTAACTAACAATACCGTGACTTTGCAAACAACTATATCAA---CCAACACTAC------TAT------CATAAT------------------AAATACAACTG---AAAACAGTACGCCGTTCTCAAATTGCTCGTCAC---CTAAT------------------------------------------------AG---CACATTGAGTACGATGTTACAAGAATCTGAAACACTATTGAACGCGGCACAAGGAGAGAATATCACTATAAAACACAACCTAACCATAACATCGTGCTACAAAACAGCTTGGCTTAGACATTTTAATATATCCACACATGGAAAATACACCCATTCAAAAATAAAAAATGGAAG---ATATTATAACTATTCATTAAAAATTCTTCATTCGCGTACACTATGTGAGTCTCAAACACACTATTTAAAACATCATTATGATTTATGTTTTACATGTGATCACAATTTTTCTTTGTCTCTGTACGGTCTCAATTTTACTCATTCTGGGAAATATAGCTTTCGATGTTACAAGTATGATCATCCCTCTGAACAAA------------ATCAAAACTTTAATCTACAAGTACATCCTAGAAATGACACAAACGGGACAGATGTGAATCCATGGATATGCGAAGAACCAAAGCACGAATGGAAGACTTTGGCTGCCACGTCTCGTAAACCAACCAGTCATAAAAACTATACAACCACATCATCTACAGATCATCTGTACCGTCATAACAACCATTCTAACACATCACACAGCAGACGCACTACATGGACTTTAGTATTAATCTGTATAGCCTGCATTCTCCTGTTTTTCGTCCGAAGAGCTTTAAACAAAAAATATCATCCACTGAGGGACGATATAAGTGAATCAGAATTTATAGTTCGCTACAATCCAGAGCACGAGGACTAA

JER5409 ATGCGTACACAACATCGACGGTGGAACAAGTCATCATATACGCAAATAATATGCATGTTTATTATTTTTTGGATTCTGCAGAAAAGCAAGTGTAACAACACCACTATCGCTAATACTTCCACGCCGATTACGCCCACAAGCTTG---------ATATCTACTACACAGTTAGCATCTACCAACA---------------------------CATTTACAT------CCTCCAACGTTACCGAAAAT------------ACATCCACAGAAGCCATTACAAGTTCCGTAAAAAGCACAGATGTAAG---TTCTATTTCCACCACACCTACCACAACATCGATGACTAATGCAACTGTAATGACAATCTCACCAAACGGCGG---CATAAATTCATCGATACAACATGTAACTAACAATACCGTGACTTTGCAAACAACTATATCAA---CCAACACTAC------TAT------CATAAT------------------AAATACAACTG---AAAACAGTACGCCGTTCTCAAATTGCTCGTCAC---CTAAT------------------------------------------------AG---CACATTGAGTACGATGTTACAAGAATCTGAAACACTATTGAACGCGGCACAAGGAGAGAATATCACTATAAAACACAACCTAACCATAACATCGTGCTACAAAACAGCTTGGCTTAGACATTTTAATATATCCACACATGGAAAATACACCCATTCAAAAATAAAAAATGGAAG---ATATTATAACTATTCATTAAAAATTCTTCATTCGCGTACACTATGTGAGTCTCAAACACACTATTTAAAACATCATTATGATTTATGTTTTACATGTGATCACAATTTTTCTTTGTCTCTGTACGGTCTCAATTTTACTCATTCTGGGAAATATAGCTTTCGATGTTACAAGTATGATCATCCCTCTGAACAAA------------ATCAAAACTTTAATCTACAAGTACATCCTAGAAATGACACAAACGGGACAGATGTGAATCCATGGATATGCGAAGAACCAAAGCACGAATGGAAGACTTTGGCTGCCACGTCTCGTAAACCAACCAGTCATAAAAACTATACAACCACATCATCTACAGATCATCTGTACCGTCATAACAACCATTCTAACACATCACACAGCAGACGCACTACATGGACTTTAGTATTAATCTGTATAGCCTGCATTCTCCTGTTTTTCGTCCGAAGAGCTTTAAACAAAAAATATCATCCACTGAGGGACGATATAAGTGAATCAGAATTTATAGTTCGCTACAATCCAGAGCACGAGGACTAA

JER4041 ATGCGTACACAACATCGACGGTGGAACAAGTCATCATATACGCAAATAATATGCATGTTTATTATTTTTTGGATTCTGCAGAAAAGCAAGTGTAACAACACCACTATCGCTAATACTTCCACGCCGATTACGCCCACAAGCTTG---------ATATCTACTACACAGTTAGCATCTACCAACA---------------------------CATTTACAT------CCTCCAACGTTACCGAAAAT------------ACATCCACAGAAGCCATTACAAGTTCCGTAAAAAGCACAGATGTAAG---TTCTATTTCCACCACACCTACCACAACATCGATGACTAATGCAACTGTAATGACAATCTCACCAAACGGCGG---CATAAATTCATCGATACAACATGTAACTAACAATACCGTGACTTTGCAAACAACTATATCAA---CCAACACTAC------TAT------CATAAT------------------AAATACAACTG---AAAACAGTACGCCGTTCTCAAATTGCTCGTCAC---CTAAT------------------------------------------------AG---CACATTGAGTACGATGTTACAAGAATCTGAAACACTATTGAACGCGGCACAAGGAGAGAATATCACTATAAAACACAACCTAACCATAACATCGTGCTACAAAACAGCTTGGCTTAGACATTTTAATATATCCACACATGGAAAATACACCCATTCAAAAATAAAAAATGGAAG---ATATTATAACTATTCATTAAAAATTCTTCATTCGCGTACACTATGTGAGTCTCAAACACACTATTTAAAACATCATTATGATTTATGTTTTACATGTGATCACAATTTTTCTTTGTCTCTGTACGGTCTCAATTTTACTCATTCTGGGAAATATAGCTTTCGATGTTACAAGTATGATCATCCCTCTGAACAAA------------ATCAAAACTTTAATCTACAAGTACATCCTAGAAATGACACAAACGGGACAGATGTGAATCCATGGATATGCGAAGAACCAAAGCACGAATGGAAGACTTTGGCTGCCACGTCTCGTAAACCAACCAGTCATAAAAACTATACAACCACATCATCTACAGATCATCTGTACCGTCATAACAACCATTCTAACACATCACACAGCAGACGCACTACATGGACTTTAGTATTAATCTGTATAGCCTGCATTCTCCTGTTTTTCGTCCGAAGAGCTTTAAACAAAAAATATCATCCACTGAGGGACGATATAAGTGAATCAGAATTTATAGTTCGCTACAATCCAGAGCACGAGGACTAA

JER851 ATGCGTACACAACATCGACGGTGGAACAAGTCATCATATACGCAAATAATATGCATGTTTATTATTTTTTGGATTCTGCAGAAAAGCAAGTGTAACAACACCACTATCGCTAATACTTCCACGCCGATTACGCCCACAAGCTTG---------ATATCTACTACACAGTTAGCATCTACCAACA---------------------------CATTTACAT------CCTCCAACGTTACCGAAAAT------------ACATCCACAGAAGCCATTACAAGTTCCGTAAAAAGCACAGATGTAAG---TTCTATTTCCACCACACCTACCACAACATCGATGACTAATGCAACTGTAATGACAATCTCACCAAACGGCGG---CATAAATTCATCGATACAACATGTAACTAACAATACCGTGACTTTGCAAACAACTATATCAA---CCAACACTAC------TAT------CATAAT------------------AAATACAACTG---AAAACAGTACGCCGTTCTCAAATTGCTCGTCAC---CTAAT------------------------------------------------AG---CACATTGAGTACGATGTTACAAGAATCTGAAACACTATTGAACGCGGCACAAGGAGAGAATATCACTATAAAACACAACCTAACCATAACATCGTGCTACAAAACAGCTTGGCTTAGACATTTTAATATATCCACACATGGAAAATACACCCATTCAAAAATAAAAAATGGAAG---ATATTATAACTATTCATTAAAAATTCTTCATTCGCGTACACTATGTGAGTCTCAAACACACTATTTAAAACATCATTATGATTTATGTTTTACATGTGATCACAATTTTTCTTTGTCTCTGTACGGTCTCAATTTTACTCATTCTGGGAAATATAGCTTTCGATGTTACAAGTATGATCATCCCTCTGAACAAA------------ATCAAAACTTTAATCTACAAGTACATCCTAGAAATGACACAAACGGGACAGATGTGAATCCATGGATATGCGAAGAACCAAAGCACGAATGGAAGACTTTGGCTGCCACGTCTCGTAAACCAACCAGTCATAAAAACTATACAACCACATCATCTACAGATCATCTGTACCGTCATAACAACCATTCTAACACATCACACAGCAGACGCACTACATGGACTTTAGTATTAATCTGTATAGCCTGCATTCTCCTGTTTTTCGTCCGAAGAGCTTTAAACAAAAAATATCATCCACTGAGGGACGATATAAGTGAATCAGAATTTATAGTTCGCTACAATCCAGAGCACGAGGACTAA

HAN1 ATGCGTACACAACATCGACGGTGGAACAAGTCATCATATACGCAAATAATATGCATGTTTATTATTTTTTGGATTCTGCAGAAAAGCAAGTGTAACAACACCACTATCGCTAATACTTCCACGCCGATTACGCCCACAAGCTTG---------ATATCTACTACACAGTTAGCATCTACCAACA---------------------------CATTTACAT------CCTCCAACGTTACCGAAAAT------------ACATCCACAGAAGCCATTACAAGTTCCGTAAAAAGCACAGATGTAAG---TTCTATTTCCACCACACCTACCACAACGTCGATGACTAATGCAACTGTAATGACAATCTCACCAAACGGCGG---CATAAATTCATCGATACAACATGTAACTAACAATACCGTGACTTTGCAAACAACTATATCAA---CCAACACTAC------TAT------CATAAT------------------AAATACAACTG---AAAACAGTACGCCGTTCTCAAATTGCTCGTCAC---CTAAT------------------------------------------------AG---CACATTGAGTACGATGTTACAAGAATCTGAAACACTATTGAACGCGGCACAAGGAGAGAATATCACTATAAAACACAACCTAACCATAACATCGTGCTACAAAACAGCTTGGCTTAGACATTTTAATATATCCACACATGGAAAATACACCCATTCAAAAATAAAAAATGGAAG---ATATTATAACTATTCATTAAAAATTCTTCATTCGCGTACACTATGTGAGTCTCAAACACACTATTTAAAACATCATTATGATTTATGTTTTACATGTGATCACAATTTTTCTTTGTCTCTGTACGGTCTCAATTTTACTCATTCTGGGAAATATAGCTTTCGATGTTACAAGTATGATCATCCCTCTGAACAAA------------ATCAAAACTTTAATCTACAAGTACATCCTAGAAATGACACAAACGGGACAGATGTGAATCCATGGATATGCGAAGAACCAAAGCACGAATGGAAGACTTTGGCTGCCACGTCTCGTAAACCAACCAGTCATAAAAACTATACAACCACATCATCTACAGATCATCTGTACCGTCATAACAACCATTCTAACACATCACACAGCAGACGCACTACATGGACTTTAGTATTAATCTGTATAGCCTGCATTCTCCTGTTTTTCGTCCGAAGAGCTTTAAACAAAAAATATCATCCACTGAGGGACGATATAAGTGAATCAGAATTTATAGTTCGCTACAATCCAGAGCACGAGGACTAA

HAN36 G2 ATGCGTACACAACATCGACGGCGAAACAAGTCATCGTACACGCAAATAACATGCATGTTTATCATTTTTTGGATTCTGCAGAAAAGCAAGTGTAACAACACCACTATCGCTAATACTTCCACGTCAATTACACTCACAAGCTTG---------ATATCTACTGCACAACTAACATCTACTTTACAAACCACCGGAAT---GTCTACCACTACATTCACAT------CCTCCGATGTCAACGCCAAC------------ACATCCACAGGATTCACTGCAAGCTCTGCAAAAAGCACAGACGTGATCTCAACTATTTCCACCATACCCACTCAAACATCTACAATTAACGCGACTGTAATGACAACCTCACCAAACGGAGG---CATGAATTTATCGACACAACATATAATCAGCAGTACCGCGACTTCGCAAGCAACTACATCAT---TACCAATCAA------TAC------TAGTAC---AATG------GTAACAAATACAACTCAAAACATCAGTACACCACTCCCAACTTGCTCATCAT---CTAATAGCACATTCAATGATACATCAAACAACCGTACTTGT------------CA---TGAAAACAGTACAATATCACAAGAATCTGAAACATTGTTGAAGGCAATACAAGGAGACAATATCACTATAATACACAACCTAACCACCACATCGTGCTACAAGACAGCTTGGCTTAGACATTTTAATATATCCACACACAGAAAATACACCCATCCCAACATAAAGAGTGGAAA---ATTTAGTAACCATTCATTAAAGATCCTCCATTCGCGTGTACTGTGTGAGTGGCAGACACATTACCTAAAACATCACTACGATTTATGTTTTACATGCGATCAGAATTTATCTTTGTCTCTGTACGGTCTTAATTTTACTCACTCTGGTAAATATAGCTTTCGATGTTACAAAAGTGGCCATCCCTCTGAACAAA------------ATCAAAATTTTAATCTACAAGTACATCCTAGAAACAACACGAACGAGACACATGTGAACCCCTGGATATGCGAAGAACCAAAGCACGAATGGGATACTTTGGCTGCTACATCTGATAAACCGACCAGTCATAAAGACGATACAACCACATCATCTACAGATCATCTATACCGCTATAATAATCATTCCAACACATCACACGGCAGACACACTACGTGGACTTTAGTGTTAATTTGTATAGCCTGCATTCTCCTATTTTTCGTCCGACGAGCTCTAAACAAAAAATACCATCCATTAAGGGACGATATCAGTGAATCAGAATTCATAGTTCGATACAATCCTGAGCATGAGGATTAA

TB40/E ATGCGTACACAACATCGACGGCGAAACAAGTCATCGTACACGCAAATAACATGCATGTTTATCATTTTTTGGATTCTGCAGAAAAGCAAGTGTAACAACACCACTATCGCTAATACTTCCACGTCAATTACACTCACAAGCTTG---------ATATCTACTGCACAACTAACATCTACTTTACAAACCACCGGAAT---GTCTACCACTACATTCACAT------CCTCCGATGTCAACGCCAAC------------ACATCCACAGGATTCACTGCAAGCTCTGCAAAAAGCACAGACGTGATCTCAACTATTTCCACCATACCCACTCAAACATCTACAATTAACGCGACTGTAATGACAACCTCACCAAACGGAGG---CATGAATTTATCGACACAACATATAATCAGCAGTACCGCGACTTCGCAAGCAACTACATCAT---TACCAATCAA------TAC------TAGTAC---AATG------GTAACAAATACAACTCAAAACATCAGTACACCACTCCCAACTTGCTCATCAT---CTAATAGCACATTCAATGATACATCAAACAACCGTACTTGT------------CA---TGAAAACAGTACAATATCACAAGAATCTGAAACATTGTTGAAGGCAATACAAGGAGACAATATCACTATAATACACAACCTAACCACCACATCGTGCTACAAGACAGCTTGGCTTAGACATTTTAATATATCCACACACAGAAAATACACCCATCCCAACATAAAGAGTGGAAA---ATTTAGTAACCATTCATTAAAGATCCTCCATTCGCGTGTACTGTGTGAGTGGCAGACACATTACCTAAAACATCACTACGATTTATGTTTTACATGCGATCAGAATTTATCTTTGTCTCTGTACGGTCTTAATTTTACTCACTCTGGTAAATATAGCTTTCGATGTTACAAAAGTGGCCATCCCTCTGAACAAA------------ATCAAAATTTTAATCTACAAGTACATCCTAGAAACAACACGAACGAGACACATGTGAACCCCTGGATATGCGAAGAACCAAAGCACGAATGGGATACTTTGGCTGCTACATCTGATAAACCGACCAGTCATAAAGACGATACAACCACATCATCTACAGATCATCTATACCGCTATAATAATCATTCCAACACATCACACGGCAGACACACTACGTGGACTTTAGTGTTAATTTGTATAGCCTGCATTCTCCTATTTTTCGTCCGACGAGCTCTAAACAAAAAATACCATCCATTAAGGGACGATATCAGTGAATCAGAATTCATAGTTCGATACAATCCTGAGCATGAGGATTAA

HAN25 ATGCGTACACAACATCGACGGCGAAACAAGTCATCGTACACGCAAATAACATGCATGTTTATCATTTTTTGGATTCTGCAGAAAAGCAAGTGTAACAACACCACTATCGCTAATACTTCCACGTCAATTACACTCACAAGCTTG---------ATATCTACTGCACAACTAACATCTACTTTACAAACCACCGGAAT---GTCTACCACTACATTCACAT------CCTCCGATGTCAACGCCAAC------------ACATCCACAGGATTCACTGCAAGCTCTGCAAAAAGCACAGACGTGATCTCAACTATTTCCACCATACCCACTCAAACATCTACAATTAACGCGACTGTAATGACAACCTCACCAAACGGAGG---CATGAATTTATCGACACAACATATAATCAGCAGTACCGCGACTTCGCAAGCAACTACATCAT---TACCAATCAA------TAC------TAGTAC---AATG------GTAACAAATACAACTCAAAACATCAGTACACCACTCCCAACTTGCTCATCAT---CTAATAGCACATTCAATGATACATCAAACAACCGTACTTGT------------CA---TGAAAACAGTACAATATCACAAGAATCTGAAACATTGTTGAAGGCAATACAAGGAGACAATATCACTATAATACACAACCTAACCACCACATCGTGCTACAAGACAGCTTGGCTTAGACATTTTAATATATCCACACACAGAAAATACACCCATCCCAACATAAAGAGTGGAAA---ATTTAGTAACCATTCATTAAAGATCCTCCATTCGCGTGTACTGTGTGAGTGGCAGACACATTACCTAAAACATCACTACGATTTATGTTTTACATGCGATCAGAATTTATCTTTGTCTCTGTACGGTCTTAATTTTACTCACTCTGGTAAATATAGCTTTCGATGTTACAAAAGTGGCCATCCCTCTGAACAAA------------ATCAAAATTTTAATCTACAAGTACATCCTAGAAACAACACGAACGAGACACATGTGAACCCCTGGATATGCGAAGAACCAAAGCACGAATGGGATACTTTGGCTGCTACATCTGATAAACCGACCAGTCATAAAGACGATACAACCACATCATCTACAGATCATCTATACCGCTATAATAATCATTCCAACACATCACACGGCAGACACACTACGTGGACTTTAGTGTTAATTTGTATAGCCTGCATTCTCCTATTTTTCGTCCGACGAGCTCTAAACAAAAAATACCATCCATTAAGGGACGATATCAGTGAATCAGAATTCATAGTTCGATACAATCCTGAGCATGAGGATTAA

AF1 ATGCGTACACAACATCGACGGCGAAACAAGTCATCGTACACGCAAATAACATGCATGTTTATCATTTTTTGGATTCTGCAGAAAAGCAAGTGTAACAACACCACTATCGCTAATACTTCCACGTCAATTACACTCACAAGCTTG---------ATATCTACTGCACAACTAACATCTACTTTACAAACCACCGGAAT---GTCTACCACTACATTCACAT------CCTCCGATGTCAACGCCAAC------------ACATCCACAGGATTCACTGCAAGCTCTGCAAAAAGCACAGACGTGATCTCAACTATTTCCACCATACCCACTCAAACATCTACAATTAACGCGACTGTAATGACAACCTCACCAAACGGAGG---CATGAATTTATCGACACAACATATAATCAGCAGTACCGCGACTTCGCAAGCAACTACATCAT---TACCAATCAA------TAC------TACTAC---AATG------GTAACAAATACAACTCAAAACATCAGTACACCACTCCCAACTTGCTCATCAT---CTAATAGCACATTCAATGATACATCAAACAACCGTACTTGT------------CA---TGAAAACAGTACAATATCACAAGAATCTGAAACATTGTTGAAGGCAATACAAGGAGACAATATCACTATAATACACAACCTAACCACCACATCGTGCTACAAGACAGCTTGGCTTAGACATTTTAATATATCCACACACAGAAAATACACCCATCCCAACATAAAGAGTGGAAA---ATTTAGTAACCATTCATTAAAGATCCTCCATTCGCGTGTACTGTGTGAGTGGCAGACACATTACCTAAAACATCACTACGATTTATGTTTTACATGCGATCAGAATTTATCTTTGTCTCTGTACGGTCTTAATTTTACTCACTCTGGTAAATATAGCTTTCGATGTTACAAAAGTGGCCATCCCTCTGAACAAA------------ATCAAAATTTTAATCTACAAGTACATCCTAGAAACAACACGAACGAGACACATGTGAACCCCTGGATATGCGAAGAACCAAAGCACGAATGGGATACTTTGGCTGCTACATCTGATAAACCGACCAGTCATAAAGACGATACAACCACATCATCTACAGATCATCTATACCGCTATAATAATCATTCCAACACATCACACGGCAGACGCACTACGTGGACTTTAGTGTTAATTTGTATAGCCTGCATTCTCCTATTTTTCGTCCGACGAGCTCTAAACAAAAAATACCATCCATTAAGGGACGATATCAGTGAATCAGAATTCATAGTTCGATACAATCCTGAGCATGAGGATTAA

UK/Lon2 G1A ATGCGCACACAACATCGACGGTGGAACAAGTCATCATATACGCAAATAATATGCATGTTTATTATTTTTTGGATTCTGCAGAAAAGCAAGTGTAACAACACCACTATCGCCAATACTTCCACGTCGATTACACCCACAAGCTTA---------ATATCTACTACACAACTGACATCTACGTTACAAACCACCGAAAT---GTCTACCACTATGTTCACAT------CCTCCAATGGCAACGTCAAC------------ACATCCACAGGATTCACTGCAAGCTCTGTAAAAGGCACAGACGTGACCTCAACTATTTCCACCATATCTACCCAAACATCTACAACTAACGTAACTGTAATAACAACTTCACCAAACGGCGA---CACGAATTCATCGACACAGCATGTAACCGATANNNNNNNNNNNNNNNNNNNNNNNNNNNNNN---NNNNNNNNNN------NNN------NNNNNN---NNN------------NNNNNNNNNNNNNNNNNNNNNNNNNNNNNNNNNNNNNNNNNNNNNNN---NNNNNNN---------------------------------------------------NNNNNNNNNNNNNNNNNNNNNNNNNNNNNNNNNNNNNNNNNNNNNNNNNNNNNNNNNNNNNNNNNNNNNNNNNNNNNNNNNNNNNNNNNNNNNGTGCTACAAAACAGCCTGGCTTAGACATTTTAATATATCCACACACGGAAAATACACCCATCCCAACATAAGAAATGGAAA---ATATCATAACCATTCATTGAAAATCCTCCATTCGCGTATACTATGTGAGTGGCACACAAATTATCTAAAACATCACTATGATTTATGTTTTACATGCGATCGTAATTTATCTTTATCTCTGTACGGTCTTAATTTTACTCATTCTGGTAAATATAGCTTTCGATGTTATAAAACTGGGCATCCCTCCGAACAAA------------ATCAAAACTTTAATCTGCAAATACATCCTAGAAACAACACAAACGGGACACACGTGAATCCCTGGGTATGTGAAGAACCAAAGCACGAATGGGACACTTCTCA---------TAAACCGACC---AATTATGAAGACAATACAGCCACATCATCTATAGATCATTTATACCGCTATAACAATCATTCTAACACATCACACGGCAGACGCACTACGTGGACGTTAGCATTAATTTGTGTAGCCTGCATTCTCCTATTTTTCGTCCGACGAGCTCTAAATAAAAAATATCATCCATTAAGTGACGATATAAGTGAATCAGAATTCATAGTTCGATACAATCCTGAGCACGAGGACTAA

UK/Lon9 ATGCGTACACAACATCGACGGTGGAACAAGTCATCATATACGCAAATAATATGCATGTTTATTATTTTTTGGATTCTGCAGAAAAGCAAGTGTAACAACACCACTATCGTTAATACTTCCACGTCGATTACACCCACAAGCTTA---------ATATCTACTACACAACTGACATCTAAGTTACAAACCACCGAAAT---GTCTACCACTATGTTCACAT------CCTCCAATGGCAACGTCAAC------------ACATCCACAGGATTCACTGCAAGCTCTGTAAAAGGCACAGACGTGACCTCGACTAGTTCCACCATATCTACCCAAACATCTACAACTAACGTAAATGTAATAACAACTTCACCAAACGGCGA---CACGAATTCATCGACACAGCATGTAACCGATAGTACTGTGACTTTGCAAACTATATCATTAT---CAACCAACAC------TAC------TNNNNN---NNN------------NNNNNNNNNNNNNNNNNNNNNNNNNNNNNNNNNNNNTTGCTCATCGC---CTAACAG---------------------------------------------------CACAAATAATACGATATCAAAAGAATCTGAAACATTATTGGAGGCGGCACAAGGAGACAATATTACTATAACACACAACCTAACCATCACATCGTGCTACAAAACAGCCTGGCTTAGACATTTTAATATATCCACACACGGAAAATACACCCATCCCAACATAAGAAATGGAAA---ATATCATAACCATTCATTGAAAATCCTCCATTCGCGTATACTATGTGAGTGGCACACAAATTATCTAAAACATCACTATGATTTATGTTTTACATGCGATCGTAATTTATCTTTATCTCTGTACGGTCTTAATTTTACTCATTCTGGTAAATATAGCTTTCGATGTTATAAAACTGGGCATCCCTCCGAACAAA------------ATCAAAACTTTAATCTGCAAGTACATCCTAGAAACAACACAAACGGGACACACGTGAATCCTTGGGTATGTGAAGAACCAAAACACGAATGGGACACTTCTCA---------TAAACCGACC---AATTATAAAGACAATACAGCCACATCATCTATAGATCATTTATACCGCTATAACAATCATTCTAACACATCACACGGCAGACGCACTACGTGGACGTTAGCATTAATTTGTGTAGCCTGCATTCTCCTATTTTTCGTCCGACGAGCTCTAAATAAAAAATATCATCCATTAAGTGACGATATAAGTGAATCAGAATTCATAGTTCGATACAATCCTGAGCACGAGGACTAA

HAN11 ATGCGCACACAACATCGACGGTGGAACAAGTCATCATATACGCAAATAATATGCATGTTTATTATTTTTTGGATTCTGCAGAAAAGCAAGTGTAACAACACCACTATCGCTAATACTTCCACGTCGATTACACCCACAAGCTTG---------ATATCTACTACACAACTGACATCTACGTTACAAACCACCGAAAT---GTCTACCACTATGTTCACAT------CCTCCAATGGCAACGTCAAC------------ACATCCACAGGATTCACTGCAAGCTCTGTAAAAGGCACAGACGTGACCTCAACTATTTCCACCATATCTACCCAAACATCTACAACTAACGTAACTGTAATAACAACTTCACCAAACGGCGA---CACGAATTCATCGACACAGCATGTAACCGATAGTACTGTGACTTTGCAAACTATATCATTAT---CAACCAACAC------TAC------TACTAT---GGA------------AAATGCAAATGAAAACGTCACTACACCGCTTCCAACTTGCTCATCGC---CTAACAG---------------------------------------------------CACAAATAATACGATATCAAAAGAATCTGAAACATTATTGGAGGCGGCACAAGGAGACAATATTACTATAACACACAACCTAACCATCACATCGTGCTACAAAACAGCCTGGCTTAGACATTTTAATATATCCACACACGGAAAATACACCCATCCCAACATAAGAAATGGAAA---ATATCATAACCATTCATTGAAAATCCTCCATTCGCGTATACTATGTGAGTGGCACACAAATTATCTAAAACATCACTATGATTTATGTTTTACATGCGATCGTAATTTATCTTTATCTCTGTACGGTCTTAATTTTACTCATTCTGGTAAATATAGCTTTCGATGTTATAAAACTGGGCATCCCTCCGAACAAA------------ATCAAAACTTTAATTTACAAGTACATCCTAGAAACAACACAAACGGGACACACGTAAATCCCTGGATATGCGAAGAACCAAAGCACGAATGGGACACTTCTCA---------TAAACCGACC---AGTCACA---AAGATGATACAACCACATCTACCAATCATTTATACCGCTATAACAATCATTCTAACACATCACACGGCAGACGCACTACGTGGACGTTAGCGTTAATTTGTGTAGCCTGCATTCTCCTATTTTTCGTCCGACGAGCTCTAAATAAAAAATATCATCCATTAAGTGACGATATAAGTGAATCAGAATTCATAGTTCGATACAATCCTGAGCACGAGGACTAA

BE/7/2011 ATGCGCACACAACATCGACGGTGGAACAAGTCATCATATACGCAAATAATATGCATGTTTATTATTTTTTGGATTCTGCAGAAAAGCAAGTGTAACAACACCACTATCGCTAATACTTCCACGTCGATTACACCCACAAGCTTA---------ATATCTACTACACAACTGACATCCACGTTACAAACCACCGAAAT---GTCTACCACTATGTTCACAT------CCTCCAATGGCAACGTCAAC------------ACATCCACAGGATTCACTGCAAGCTCTGTAAAAGGCACAGACGTGACCTCAACTATTTCCGCCATATCTACCCAAACATCTACAACTAACGTAACT-----AATAACTTCACCAAACGGCGA---CACGAATTCATCGACACAGCATGTAACCGATAGTACTGTGACTTTGCAAACTATATCATTAT---CAACCAACAC------TAC------TACTAT---GGT------------AAATGCAAATGAAAACGTCACTACACCGCTTCCAACTTGCTCATCGC---CTAACAG---------------------------------------------------CACAAATAATACTATATCAAAAGAACCTGAAACATTATTGGAGGCGGCACAAGGAGACAATATTACTATAACACACAACCTAACCATCACATCGTGCTACAAAACAGCCTGGCTTAGACATTTTAATATATCCACACACGGAAAATACACTCATCCCAACATAAGAAATGGAAA---ATATTATAACCATTCATTGAAAATCCTCCATTCGCGTATACTATGTGAGTGGCACACAAATTATCTAAAACATCACTATGATTTATGTTTTACATGCGATCGTAATTTATCTTTATCTCTGTACGGTCTTAATTTTACTCATTCTGGTAAATATAGCTTTCGATGTTATAAAACTGGCCATCCCTCCGAACAAA------------ATCAAAACTTTAATCTGCAAATACATCCTAGAAACAACACAAACGGGACACACGTGGATCCCTGGGTATGTGAAGAACCAAAGCACGAATGGGACACTTCTCC---------TCATAAACCGACCAATTATGAAGACAATACAGCCACATCATCTATAGATCATTTATACCGCTATAACAATCATTCTAACACATCACACGGCAGACGTACTACGTGGACGTTAGCATTAATTTGTGTAGCCTGCATTCTCCTATTTTTCGTCCGACGAGCTCTAAATAAAAAATATCATCCATTAAGTGACGATATAAGTGAATCAGAATTCATAGTTCGATACAATCCTGAGCACGAGGACTAA

HANRTR2 ATGCGCACACAACATCGACGGTGGAACAAGTCATCATATACGCAAATAATATGCATGTTTATTATTTTTTGGATTCTGCAGAAAAGCAAGTGTAACAACACCACTATCGCTAATACTTCCACGTCGATTACACCCACAAGCTTA---------ATATCTACTACACAACTGACATCCACGTTACAAACCACCGAAAT---GTCTACCACTATGTTCACAT------CCTCCAATGGCAACGTCAAC------------ACATCCACAGGATTCACTGCAAGCTCTGTAAAAGGCACAGACGTGACCTCAACTATTTCCGCCATATCTACCCAAACATCTACAACTAACGTAACTGTAATA---ACTTCACCAAACGGCGA---CACGAATTCATCGACACAGCATGTAACCGATAGTACTGTGACTTTGCAAACTATATCATTAT---CAACCAACAC------TAC------TACTAT---GGT------------AAATGCAAATGAAAACGTCACTACACCGCTTCCAACTTGCTCATCGC---CTAACAG---------------------------------------------------CACAAATAATACTATATCAAAAGAACCTGAAACATTATTGGAGGCGGCACAAGGAGACAATATTACTATAACACACAACCTAACCATCACATCGTGCTACAAAACAGCCTGGCTTAGACATTTTAATATATCCACACACGGAAAATACACTCATCCCAACATAAGAAATGGAAA---ATATTATAACCATTCATTGAAAATCCTCCATTCGCGTATACTATGTGAGTGGCACACAAATTATCTAAAACATCACTATGATTTATGTTTTACATGCGATCGTAATTTATCTTTATCTCTGTACGGTCTTAATTTTACTCATTCTGGTAAATATAGCTTTCGATGTTATAAAACTGGCCATCCCTCCGAACAAA------------ATCAAAACTTTAATCTGCAAATACATCCTAGAAACAACACAAACGGGACACACGTGGATCCCTGGGTATGTGAAGAACCAAAGCACGAATGGGACACTTCTCC---------TCATAAACCGACCAATTATGAAGACAATACAGCCACATCATCTATAGATCATTTATACCGCTATAACAATCATTCTAACACATCACACGGCAGATGTACTACGTGGACGTTAGCATTAATTTGTGTAGCCTGCATTCTCCTATTTTTCGTCCGACGAGCTCTAAATAAAAAATATCATCCATTAAGTGACGATATAAGTGAATCAGAATTCATAGTTCGATACAATCCTGAGCACGAGGACTAA

UK/Lon8 ATGCGCACACAACATCGACGGTGGAACAAGTCATCATATACGCAAATAATATGCATGTTTATTATTTTTTGGATTCTGCAGAAAAGCAAGTGTAACAACACCACTATCGCTAATACTTCCACGTCGATTACACCCACAAGCTTA---------ATATCTACTACACAACTGACATCCACGTTACAAACCACCGAAAT---GTCTACCACTATGTTCACAT------CCTCCAATGGCAACGTCAAC------------ACATCCACAGGATTCACTGCAAGCTCTGTAAAAGGCACAGACGTGACCTCAACTATTTCCGCCATATCTACCCAAACATCTACAACTAACGTAACTGTAATAACAACTTCACCAAACGGCGA---CACGAATTCATCGACACAGCATGTAACCGATAGTACTGTGACTTTGCAAACTATATCATTAT---CAACCAACAC------TAC------TACTAT---GGT------------AAATGCAAATGAAAACGTCACTACACCGCTTCCAACTTGCTCATCGC---CTAACAG---------------------------------------------------CACAAATAATACTATATCAAAAGAACCTGAAACATTATTGGAGGCGGCACAAGGAGACAATATTACTATAACACACAACCTAACCATCACATCGTGCTACAAAACAGCCTGGCTTAGACATTTTAATATATCCACACACGGAAAATACACTCATCCCAACATAAGAAATGGAAA---ATATTATAACCATTCATTGAAAATCCTCCATTCGCGTATACTATGTGAGTGGCACACAAATTATCTAAAACATCACTATGATTTATGTTTTACATGCGATCGTAATTTATCTTTATCTCTGTACGGTCTTAATTTTACTCATTCTGGTAAATATAGCTTTCGATGTTATAAAACTGGGCATCCCTCCGAACAAA------------ATCAAAACTTTAATCTGCAAATACATCCTAGAAACAACACAAACGGGACACACGTGGATCCCTGGGTATGTGAAGAACCAAAGCACGAATGGGACACTTCTCA------------TAAACCGACCAATTATGAAGACAATACAGCCACATCATCTATAGATCATTTATACCGCTATAACAATCATTCTAACACATCACACGGCAGACGTACTACGTGGACGTTAGCATTAATTTGTGTAGCCTGCATTCTCCTATTTTTCGTCCGACGAGCTCTAAATAAAAAATATCATCCATTAAGTGACGATATAAGTGAATCAGAATTCATAGTTCGATACAATCCTGAGCACGAGGACTAA

Pat_C ATGCGCACACAACATCGACGGTGGAACAAGTCATCATATACGCAAATAATATGCATGTTTATTATTTTTTGGATTCTGTAGAAAAGCAAGTGTAACAACACCACTATCGCTAATACTTCCACGTCGATTACACCCACAAGCTTA---------ATATCTATTACACAACTGACATCCACGTTACAAACCACCGAAAT---GTCTACCACTATGTTCACAT------CCTCCAATGGCAACGTCAAC------------ACATCCACAGGATTCACTGCAAGCTCTGTAAAAGGCACAGACGTGACCTCAACTATTTCCGCCATATCTACCCAAACATCTACAACTAACGTAACTGTAATAACAACTTCACCAAACGGCGA---CACGAATTCATCGACACAGCATGTAACCGATAGTACTGTGACTTTGCAAACTATATCATTAT---CAACCAACAC------TAC------TACTAT---GGT------------AAATGCAAATGAAAACGTCACTACACCGCTTCCAACTTGCTCATCGC---CTAACAG---------------------------------------------------CACAAATAATACTATATCAAAAGAACCTGAAACATTATTGGAGGCGGCACAAGGAGACAATATTACTATAACACACAACCTAACCATCACATCGTGCTACAAAACAGCCTGGCTTAGACATTTTAATATATCCACACACGGAAAATACACTCATCCCAACATAAGAAATGGAAA---ATATTATAACCATTCATTGAAAATCCTCCATTCGCGTATACTATGTGAGTGGCACACAAATTATCTAAAACATCACTATGATTTATGTTTTACATGCGATCGTAATTTATCTTTATCTCTGTACGGTCTTAATTTTACTCATTCTGGTAAATATAGCTTTCGATGTTATAAAACTGGGCATCCCTCCGAACAAA------------ATCAAAACTTTAATCTGCAAATACATCCTAGAAACAACACAAACGGGACACACGTGGATCCCTGGGTATGTGAAGAACCAAAGCACGAATGGGACACTTCTCC---------TCATAAACCGACCAATTATGAAGACAATACAGCCACATCATCTATAGATCATTTATACCGCTATAACAATCATTCTAACACATCACACGGCAGACGTACTACGTGGACGTTAGCATTAATTTGTGTAGCCTGCATTCTCCTATTTTTCGTCCGACGAGCTCTAAATAAAAAATATCATCCATTAAGTGACGATATAAGTGAATCAGAATTCATAGTTCGATACAATCCTGAGCACGAGGACTAA

NAN4LA ATGCGCACACAACATCGACGGTGGAACAAGTCATCATATACGCAAATAATATGCATGTTTATTATTTTTTGGATTCTGCAGAAAAGCAAGTGTAACAACACCACTATCGCTAATACTTCCACGTCGATTACACCCACAAGCTTA---------ATATCTACTACACAACTGACATCCACGTTACAAACCACCGAAAT---GTCTACCACTATGTTCACAT------CCTCCAATGGCAACGTCAAC------------ACATCCACAGGATTCACTGCAAGCTCTGTAAAAGGCACAGACGTGACCTCAACTATTTCCGCCATATCTACCCAAACATCTACAACTAACGTAACTGTAATAACAACTTCACCAAACGGCGA---CACGAATTCATCGACACAGCATGTAACCGATAGTACTGTGACTTTGCAAACTATATCATTAT---CAACCAACAC------TAC------TACTAT---GGT------------AAATGCAAATGAAAACGTCACTACACCGCTTCCAACTTGCTCATCGC---CTAACAG---------------------------------------------------CACAAATAATACTATATCAAAAGAACCTGAAACATTATTGGAGGCGGCACAAGGAGACAATATTACTATAACACACAACCTAACCATCACATCGTGCTACAAAACAGCCTGGCTTAGACATTTTAATATATCCACACACGGAAAATACACTCATCCCAACATAAGAAATGGAAA---ATATTATAACCATTCATTGAAAATCCTCCATTCGCGTATACTATGTGAGTGGCACACAAATTATCTAAAACATCACTATGATTTATGTTTTACATGCGATCGTAATTTATCTTTATCTCTGTACGGTCTTAATTTTACTCATTCTGGTAAATATAGCTTTCGATGTTATAAAACTGGGCATCCCTCCGAACAAA------------ATCAAAACTTTAATCTGCAAATACATCCTAGAAACAACACAAACGGGACACACGTGGATCCCTGGGTATGTGAAGAACCAAAGCACGAATGGGACACTTCTCC---------TCATAAACCGACCAATTATGAAGACAATACAGCCACATCATCTATAGATCATTTATACCGCTATAACAATCATTCTAACACATCACACGGCAGACGTACTACGTGGACGTTAGCATTAATTTGTGTAGCCTGCATTCTCCTATTTTTCGTCCGACGAGCTCTAAATAAAAAATATCATCCATTAAGTGACGATATAAGTGAATCAGAATTCATAGTTCGATACAATCCTGAGCACGAGGACTAA

BE/26/2010 ATGCGCACACAACATCGACGGTGGAACAAGTCATCATATACGCAAATAATATGCATGTTTATTATTTTTTGGATTCTGCAGAAAAGCAAGTGTAACAACACCACTATCGCTAATACTTCCACGTCGATTACACCCACAAGCTTA---------ATATCTACCACACAACTGACATCCACGTTACAAACCACCGAAAT---GTCTACCACTATGTTCACAT------CCTCCAATGGCAACGTCAAC------------ACATCCACAGGATTCACTGCAAGCTCTGTAAAAGGCACAGACGTGACCTCAACTATTTCCGCCATATCTACCCAAACATCTACAACTAACGTAACTGTAATAACAACTTCACCAAACGGCGA---CACGAATTCATCGACACAGCATGTAACCGATAGTACTGTGACTTTGAAAACTATATCATTAT---CAACCAACAC------TAC------TACTAT---GGT------------AAATGCAAATGAAAACGTCACTACACCGCTTCCAACTTGCTCATCGC---CTAACAG---------------------------------------------------CACAAATAATACTATATCAAAAGAACCTGAAACATTATTGGAGGCGGCACAAGGAGACAATATTACTATAACACACAACCTAACCATCACATCGTGCTACAAAACAGCCTGGCTTAGACATTTTAATATATCCACACACGGAAAATACACTCATCCCAACATAAGAAATGGAAA---ATATTATAACCATTCATTGAAAATCCTCCATTCGCGTATACTATGTGAGTGGCACACAAATTATCTAAAACATCACTATGATTTATGTTTTACATGCGATCGTAATTTATCTTTATCTCTGTACGGTCTTAATTTTACTCATTCTGGTAAATATAGCTTTCGATGTTATAAAACTGGCCATCCCTCCGAACAAA------------ATCAAAACTTTAATCTGCAAATACATCCTAGAAACAACACAAACGGGACACACGTGGATCCCTGGGTATGTGAAGAACCAAAGCACGAATGGGACACTTCTCC---------TCATAAACCGACCAATTATGAAGACAATACAGCCACATCATCTATAGATCATTTATACCGCTATAACAATCATTCTAACACATCACACGGCAGACGTACTACGTGGACGTTAGCATTAATTTGTGTAGCCTGCATTCTCCTATTTTTCGTCCGACGAGCTCTAAATAAAAAATATCATCCATTAAGTGACGATATAAGTGAATCAGAATTCATAGTTCGATACAATCCTGAGCACGAGGACTAA

BE/11/2012 ATGCGCACACAACATCGACGGTGGAACAAGTCATCATATACGCAAATAATATGCATGTTTATTATTTTTTGGATTCTGCAGAAAAGCAAGTGTAACAACACCACTATCGCTAATACTTCCACGTCGATTACACCCACAAGCTTA---------ATATCTACTACACAACTGACATCCACGTTACAAACCACCGAAAT---GTCTACCACTATGTTCACAT------CCTCCAATGGCAACGTCAAC------------ACATCCACAGGATTCACTGCAAGCTCTGTAAAAGGCACAGACGTGACCTCAACTATTTCCGCCATATCTACCCAAACATCTACAACTAACGTAACTGTAATAACAACTTCACCAAACGGCGA---CACGAATTCATCGACACAGCATGTAACCGATAGTACTGTGACTTTGAAAACTATATCATTAT---CAACCAACAC------TAC------TACTAT---GGT------------AAATGCAAATGAAAACGTCACTACACCGCTTCCAACTTGCTCATCGC---CTAACAG---------------------------------------------------CACAAATAATACTATATCAAAAGAACCTGAAACATTATTGGAGGCGGCACAAGGAGACAATATTACTATAACACACAACCTAACCATCACATCGTGCTACAAAACAGCCTGGCTTAGACATTTTAATATATCCACACACGGAAAATACACTCATCCCAACATAAGAAATGGAAA---ATATTATAACCATTCATTGAAAATCCTCCATTCGCGTATACTATGTGAGTGGCACACAAATTATCTAAAACATCACTATGATTTATGTTTTACATGCGATCGTAATTTATCTTTATCTCTGTACGGTCTTAATTTTACTCATTCTGGTAAATATAGCTTTCGATGTTATAAAACTGGCCATCCCTCCGAACAAA------------ATCAAAACTTTAATCTGCAAATACATCCTAGAAACAACACAAACGGGACACACGTGGATCCCTGGGTATGTGAAGAACCAAAGCACGAATGGGACACTTCTCC---------TCATAAACCGACCAATTATGAAGACAATACAGCCACATCATCTATAGATCATTTATACCGCTATAACAATCATTCTAACACATCACACGGCAGACGTACTACGTGGACGTTAGCATTAATTTGTGTAGCCTGCATTCTCCTATTTTTCGTCCGACGAGCTCTAAATAAAAAATATCATCCATTAAGTGACGATATAAGTGAATCAGAATTCATAGTTCGACACAATCCTGAGCACGAGGACTAA

BE/30/2011 ATGCGCACACAACATCGACGGTGGAACAAGTCATCATATACGCAAATAATATGCATGTTTATTATTTTTTGGATTCTGCAGAAAAGCAAGTGTAACAACACCACTATCGCTAATACTTCCACGTCGATTACACCCACAAGCTTA---------ATATCTACTACACAACTGACATCCACGTTACAAACCACCGAAAT---GTCTACCACTATGTTCACAT------CCTCCAATGGCAACGTCAAC------------ACATCCACAGGATTCACTGCAAGCTCTGTAAAAGGCACAGACGTGACCTCAACTATTTCCGCCATATCTACCCAAACATCTACAACTAACGTAACTGTAATAACAACTTCACCAAACGGCGA---CACGAATTCATCGACACAGCATGTAACCGATAGTACTGTGACTTTGAAAACTATATCATTAT---CAACCAACAC------TAC------TACTAT---GGT------------AAATGCAAATGAAAACGTCACTACACCGCTTCCAACTTGCTCATCGC---CTAACAG---------------------------------------------------CACAAATAATACTATATCAAAAGAACCTGAAACATTATTGGAGGCGGCACAAGGAGACAATATTACTATAACACACAACCTAACCATCACATCGTGCTACAAAACAGCCTGGCTTAGACATTTTAATATATCCACACACGGAAAATACACTCATCCCAACATAAGAAATGGAAA---ATATTATAACCATTCATTGAAAATCCTCCATTCGCGTATACTATGTGAGTGGCACACAAATTATCTAAAACATCACTATGATTTATGTTTTACATGCGATCGTAATTTATCTTTATCTCTGTACGGTCTTAATTTTACTCATTCTGGTAAATATAGCTTTCGATGTTATAAAACTGGCCATCCCTCCGAACAAA------------ATCAAAACTTTAATCTGCAAATACATCCTAGAAACAACACAAACGGGACACACGTGGATCCCTGGGTATGTGAAGAACCAAAGCACGAATGGGACACTTCTCC---------TCATAAACCGACCAATTATGAAGACAATACAGCCACATCATCTATAGATCATTTATACTGCTATAACAATCATTCTAACACATCACACGGCAGACGTACTACGTGGACGTTAGCATTAATTTGTGTAGCCTGCATTCTCCTATTTTTCGTCCGACGAGCTCTAAATAAAAAATATCATCCATTAAGTGACGATATAAGTGAATCAGAATTCATAGTTCGATACAATCCTGAGCACGAGGACTAA

BE/16/2012 ATGCGCACACAACATCGACGGTGGAACAAGTCATCATATACGCAAATAATATGCATGTTTATTATTTTTTGGATTCTGCAGAAAAGCAAGTGTAACAACACCACTATCGCTAATACTTCCACGTCGATTACACCCACAAGCTTA---------ATATCTACTACACAACTGACATCCACGTTACAAACCACCGAAAT---GTCTACCACTATGTTCACAT------CCTCCAATGGCAACGTCAAC------------ACATCCACAGGATTCACTGCAAGCTCTGTAAAAGGCACAGACGTGACCTCAACTATTTCCGCCATATCTACCCAAACATCTACAACTAACGTAACTGTAATAACAACTTCACCAAACGGCGA---CACGAATTCATCGACACAGCATGTAACCGATAGTACTGTGACTTTGAAAACTATATCATTAT---CAACCAACAC------TAC------TACTAT---GGT------------AAATGCAAATGAAAACGTCACTACACCGCTTCCAACTTGCTCATCGC---CTAACAG---------------------------------------------------CACAAATAATACTATATCAAAAGAACCTGAAACATTATTGGAGGCGGCACAAGGAGACAATATTACTATAACACACAACCTAACCATCACATCGTGCTACAAAACAGCCTGGCTTAGACATTTTAATATATCCACACACGGAAAATACACTCATCCCAACATAAGAAATGGAAA---ATATTATAACCATTCATTGAAAATCCTCCATTCGCGTATACTATGTGAGTGGCACACAAATTATCTAAAACATCACTATGATTTATGTTTTACATGCGATCGTAATTTATCTTTATCTCTGTACGGTCTTAATTTTACTCATTCTGGTAAATATAGCTTTCGATGTTATAAAACTGGCCATCCCTCCGAACAAA------------ATCAAAACTTTAATCTGCAAATACATCCTAGAAACAACACAAACGGGACACACGTGGATCCCTGGGTATGTGAAGAACCAAAGCACGAATGGGACACTTCTCC---------TCATAAACCGACCAATTATGAAGACAATACAGCCACATCATCTATAGATCATTTATACCGCTATAACAATCATTCTAACACATCACACGGCAGACGTACTACGTGGACGTTAGCATTAATTTGTGTAGCCTGCATTCTCCTATTTTTCGTCCGACGAGCTCTAAATAAAAAATATCATCCATTAAGTGACGATATAAGTGAATCAGAATTCATAGTTCGATACAATCCTGAGCACGAGGACTAA

BE/7/2012 ATGCGCACACAACATCGACGGTGGAACAAGTCATCATATACGCAAATAATATGCATGTTTATTATTTTTTGGATTCTGCAGAAAAGCAAGTGTAACAACACCACTATCGCTAATACTTCCACGTCGATTACACCCACAAGCTTA---------ATATCTACTACACAACTGACATCCACGTTACAAACCACCGAAAT---GTCTACCACTATGTTCACAT------CCTCCAATGGCAACGTCAAC------------ACATCCACAGGATTCACTGCAAGCTCTGTAAAAGGCACAGACGTGACCTCAACTATTTCCGCCATATCTACCCAAACATCTACAACTAACGTAACTGTAATAACAACTTCACCAAACGGCGA---CACGAATTCATCGACACAGCATGTAACCGATAGTACTGTGACTTTGAAAACTATATCATTAT---CAACCAACAC------TAC------TACTAT---GGT------------AAATGCAAATGAAAACGTCACTACACCGCTTCCAACTTGCTCATCGC---CTAACAG---------------------------------------------------CACAAATAATACTATATCAAAAGAACCTGAAACATTATTGGAGGCGGCACAAGGAGACAATATTACTATAACACACAACCTAACCATCACATCGTGCTACAAAACAGCCTGGCTTAGACATTTTAATATATCCACACACGGAAAATACACTCATCCCAACATAAGAAATGGAAA---ATATTATAACCATTCATTGAAAATCCTCCATTCGCGTATACTATGTGAGTGGCACACAAATTATCTAAAACATCACTATGATTTATGTTTTACATGCGATCGTAATTTATCTTTATCTCTGTACGGTCTTAATTTTACTCATTCTGGTAAATATAGCTTTCGATGTTATAAAACTGGCCATCCCTCCGAACAAA------------ATCAAAACTTTAATCTGCAAATACATCCTAGAAACAACACAAACGGGACACACGTGGATCCCTGGGTATGTGAAGAACCAAAGCACGAATGGGACACTTCTCC---------TCATAAACCGACCAATTATGAAGACAATACAGCCACATCATCTATAGATCATTTATACCGCTATAACAATCATTCTAACACATCACACGGCAGACGTACTACGTGGACGTTAGCATTAATTTGTGTAGCCTGCATTCTCCTATTTTTCGTCCGACGAGCTCTAAATAAAAAATATCATCCATTAAGTGACGATATAAGTGAATCAGAATTCATAGTTCGATACAATCCTGAGCACGAGGACTAA

BE/15/2012 ATGCGCACACAACATCGACGGTGGAACAAGTCATCATATACGCAAATAATATGCATGTTTATTATTTTTTGGATTCTGCAGAAAAGCAAGTGTAACAACACCACTATCGCTAATACTTCCACGTCGATTACACCCACAAGCTTA---------ATATCTACTACACAACTGACATCCACGTTACAAACCACCGAAAT---GTCTACCACTATGTTCACAT------CCTCCAATGGCAACGTCAAC------------ACATCCACAGGATTCACTGCAAGCTCTGTAAAAGGCACAGACGTGACCTCAACTATTTCCGCCATATCTACCCAAACATCTACAACTAACGTAACTGTAATAACAACTTCACCAAACGGCGA---CACGAATTCATCGACACAGCATGTAACCGATAGTACTGTGACTTTGAAAACTATATCATTAT---CAACCAACAC------TAC------TACTAT---GGT------------AAATGCAAATGAAAACGTCACTACACCGCTTCCAACTTGCTCATCGC---CTAACAG---------------------------------------------------CACAAATAATACTATATCAAAAGAACCTGAAACATTATTGGAGGCGGCACAAGGAGACAATATTACTATAACACACAACCTAACCATCACATCGTGCTACAAAACAGCCTGGCTTAGACATTTTAATATATCCACACACGGAAAATACACTCATCCCAACATAAGAAATGGAAA---ATATTATAACCATTCATTGAAAATCCTCCATTCGCGTATACTATGTGAGTGGCACACAAATTATCTAAAACATCACTATGATTTATGTTTTACATGCGATCGTAATTTATCTTTATCTCTGTACGGTCTTAATTTTACTCATTCTGGTAAATATAGCTTTCGATGTTATAAAACTGGCCATCCCTCCGAACAAA------------ATCAAAACTTTAATCTGCAAATACATCCTAGAAACAACACAAACGGGACACACGTGGATCCCTGGGTATGTGAAGAACCAAAGCACGAATGGGACACTTCTCC---------TCATAAACCGACCAATTATGAAGACAATACAGCCACATCATCTATAGATCATTTATACCGCTATAACAATCATTCTAACACATCACACGGCAGACGTACTACGTGGACGTTAGCATTAATTTGTGTAGCCTGCATTCTCCTATTTTTCGTCCGACGAGCTCTAAATAAAAAATATCATCCATTAAGTGACGATATAAGTGAATCAGAATTCATAGTTCGATACAATCCTGAGCACGAGGACTAA

BE/3/2012 ATGCGCACACAACATCGACGGTGGAACAAGTCATCATATACGCAAATAATATGCATGTTTATTATTTTTTGGATTCTGCAGAAAAGCAAGTGTAACAACACCACTATCGCTAATACTTCCACGTCGATTACACCCACAAGCTTA---------ATATCTACTACACAACTGACATCCACGTTACAAACCACCGAAAT---GTCTACCACTATGTTCACAT------CCTCCAATGGCAACGTCAAC------------ACATCCACAGGATTCACTGCAAGCTCTGTAAAAGGCACAGACGTGACCTCAACTATTTCCGCCATATCTACCCAAACATCTACAACTAACGTAACTGTAATAACAACTTCACCAAACGGCGA---CACGAATTCATCGACACAGCATGTAACCGATAGTACTGTGACTTTGAAAACTATATCATTAT---CAACCAACAC------TAC------TACTAT---GGT------------AAATGCAAATGAAAACGTCACTACACCGCTTCCAACTTGCTCATCGC---CTAACAG---------------------------------------------------CACAAATAATACTATATCAAAAGAACCTGAAACATTATTGGAGGCGGCACAAGGAGACAATATTACTATAACACACAACCTAACCATCACATCGTGCTACAAAACAGCCTGGCTTAGACATTTTAATATATCCACACACGGAAAATACACTCATCCCAACATAAGAAATGGAAA---ATATTATAACCATTCATTGAAAATCCTCCATTCGCGTATACTATGTGAGTGGCACACAAATTATCTAAAACATCACTATGATTTATGTTTTACATGCGATCGTAATTTATCTTTATCTCTGTACGGTCTTAATTTTACTCATTCTGGTAAATATAGCTTTCGATGTTATAAAACTGGCCATCCCTCCGAACAAA------------ATCAAAACTTTAATCTGCAAATACATCCTAGAAACAACACAAACGGGACACACGTGGATCCCTGGGTATGTGAAGAACCAAAGCACGAATGGGACACTTCTCC---------TCATAAACCGACCAATTATGAAGACAATACAGCCACATCATCTATAGATCATTTATACCGCTATAACAATCATTCTAACACATCACACGGCAGACGTACTACGTGGACGTTAGCATTAATTTGTGTAGCCTGCATTCTCCTATTTTTCGTCCGACGAGCTCTAAATAAAAAATATCATCCATTAAGTGACGATATAAGTGAATCAGAATTCATAGTTCGATACAATCCTGAGCACGAGGACTAA

PAV25 ATGCGCACACAACATCGACGGTGGAACAAGTCATCATATACGCAAATAATATGCATGTTTATTATTTTTTGGATTCTGCAGAAAAGCAAGTGTAACAACACCACTATCGCTAATACTTCCACGTCGATTACACCCACAAGCTTA---------ATATCTACTACACAACTGACATCCACGTTACAAACCACCGAAAT---GTCTACCACTATGTTCACAT------CCTCCAATGGCAACGTCAAC------------ACATCCACAGGATTCACTGCAAGCTCTGTAAAAGGCACAGACGTGACCTCAACTATTTCCGCCATATCTACCCAAACATCTACAACTAACGTAACTGTAATAACAACTTCACCAAACGGCGA---CACGAATTCATCGACACAGCATGTAACCGATAGTACTGTGACTTTGCAAACTATATCATTAT---CAACCAACAC------TAC------TACTAT---GGT------------AAATGCAAATGAAAACGTCACTACACCGCTTCCAACTTGCTCATCGC---CTAACAG---------------------------------------------------CACAAATAATACTATATCAAAAGAACCTGAAACATTATTGGAGGCGGCACAAGGAGACAATATTACTATAACACACAACCTAACCATCACATCGTGCTACAAAACAGCCTGGCTTAGACATTTTAATATATCCACACACGGAAAATACACTCATCCCAACATAAGAAATGGAAA---ATATTATAACCATTCATTGAAAATCCTCCATTCGCGTATACTATGTGAGTGGCACACAAATTATCTAAAACATCACTATGATTTATGTTTTACATGCGATCGTAATTTATCTTTATCTCTGTACGGTCTTAATTTTACTCATTCTGGTAAATATAGCTTTCGATGTTATAAAACTGGCCATCCCTCCGAACAAA------------ATCAAAACTTTAATCTGCAAATACATCCTAGAAACAACACAAACGGGACACACGTGGATCCCTGGGTATGTGAAGAACCAAAGCACGAATGGGACACTTCTCC---------TCATAAACCGACCAATTATGAAGACAATACAGCCACATCATCTATAGATCATTTATACCGCTATAACAATCATTCTAACACATCACACGGCAGACGTACTACGTGGACGTTAGCATTAATTTGTGTAGCCTGCATTCTCCTATTTTTCGTCCGACGAGCTCTAAATAAAAAATATCATCCATTAAGTGACGATATAAGTGAATCAGAATTCATAGTTCGATACAATCCTGAGCACGAGGACTAA

BE/31/2010 ATGCGCACACAACATCGACGGTGGAACAAGTCATCATATACGCAAATAATATGCATGTTTATTATTTTTTGGATTCTGCAGAAAAGCAAGTGTAACAACACCACTATCGCTAATACTTCCACGTCGATTACACCCACAAGCTTA---------ATATCTACTACACAACTGACATCCACGTTACAAACCACCGAAAT---GTCTACCACTATGTTCACAT------CCTCCAATGGCAACGTCAAC------------ACATCCACAGGATTCACTGCAAGCTCTGTAAAAGGCACAGACGTGACCTCAACTATTTCCGCCATATCTACCCAAACATCTACAACTAACGTAACTGTAATAACAACTTCACCAAACGGCGA---CACGAATTCATCGACACAGCATGTAACCGATAGTACTGTGACTTTGCAAACTATATCATTAT---CAACCAACAC------TAC------TACTAT---GGT------------AAATGCAAATGAAAACGTCACTACACCGCTTCCAACTTGCTCATCGC---CTAACAG---------------------------------------------------CACAAATAATACTATATCAAAAGAACCTGAAACATTATTGGAGGCGGCACAAGGAGACAATATTACTATAACACACAACCTAACCATCACATCGTGCTACAAAACAGCCTGGCTTAGACATTTTAATATATCCACACACGGAAAATACACTCATCCCAACATAAGAAATGGAAA---ATATTATAACCATTCATTGAAAATCCTCCATTCGCGTATACTATGTGAGTGGCACACAAATTATCTAAAACATCACTATGATTTATGTTTTACATGCGATCGTAATTTATCTTTATCTCTGTACGGTCTTAATTTTACTCATTCTGGTAAATATAGCTTTCGATGTTATAAAACTGGCCATCCCTCCGAACAAA------------ATCAAAACTTTAATCTGCAAATACATCCTAGAAACAACACAAACGGGACACACGTGGATCCCTGGGTATGTGAAGAACCAAAGCACGAATGGGACACTTCTCC---------TCATAAACCGACCAATTATGAAGACAATACAGCCACATCATCTATAGATCATTTATACCGCTATAACAATCATTCTAACACATCACACGGCAGACGTACTACGTGGACGTTAGCATTAATTTGTGTAGCCTGCATTCTCCTATTTTTCGTCCGACGAGCTCTAAATAAAAAATATCATCCATTAAGTGACGATATAAGTGAATCAGAATTCATAGTTCGATACAATCCTGAGCACGAGGACTAA

HAN22 ATGCGCACACAACATCGACGGTGGAACAAGTCATCATATACGCAAATAATATGCATGTTTATTATTTTTTGGATTCTGCAGAAAAGCAAGTGTAACAACACCACTATCGCTAATACTTCCACGTCGATTACACCCACAAGCTTA---------ATATCTACTACACAACTGACATCCACGTTACAAACCACCGAAAT---GTCTACCACTATGTTCACAT------CCTCCAATGGCAACGTCAAC------------ACATCCACAGGATTCACTGCAAGCTCTGTAAAAGGCACAGACGTGACCTCAACTATTTCCGCCATATCTACCCAAACATCTACAACTAACGTAACTGTAATAACAACTTCACCAAACGGCGA---CACGAATTCATCGACACAGCATGTAACCGATAGTACTGTGACTTTGCAAACTATATCATTAT---CAACCAACAC------TAC------TACTAT---GGT------------AAATGCAAATGAAAACGTCACTACACCGCTTCCAACTTGCTCATCGC---CTAACAG---------------------------------------------------CACAAATAATACTATATCAAAAGAACCTGAAACATTATTGGAGGCGGCACAAGGAGACAATATTACTATAACACACAACCTAACCATCACATCGTGCTACAAAACAGCCTGGCTTAGACATTTTAATATATCCACACACGGAAAATACACTCATCCCAACATAAGAAATGGAAA---ATATTATAACCATTCATTGAAAATCCTCCATTCGCGTATACTATGTGAGTGGCACACAAATTATCTAAAACATCACTATGATTTATGTTTTACATGCGATCGTAATTTATCTTTATCTCTGTACGGTCTTAATTTTACTCATTCTGGTAAATATAGCTTTCGATGTTATAAAACTGGCCATCCCTCCGAACAAA------------ATCAAAACTTTAATCTGCAAATACATCCTAGAAACAACACAAACGGGACACACGTGGATCCCTGGGTATGTGAAGAACCAAAGCACGAATGGGACACTTCTCC---------TCATAAACCGACCAATTATGAAGACAATACAGCCACATCATCTATAGATCATTTATACCGCTATAACAATCATTCTAACACATCACACGGCAGACGTACTACGTGGACGTTAGCATTAATTTGTGTAGCCTGCATTCTCCTATTTTTCGTCCGACGAGCTCTAAATAAAAAATATCATCCATTAAGTGACGATATAAGTGAATCAGAATTCATAGTTCGATACAATCCTGAGCACGAGGACTAA

HAN23 ATGCGCACACAACATCGACGGTGGAACAAGTCATCATATACGCAAATAATATGCATGTTTATTATTTTTTGGATTCTGCAGAAAAGCAAGTGTAACAACACCACTATCGCTAATACTTCCACGTCGATTACACCCACAAGCTTA---------ATATCTACTACACAACTGACATCCACGTTACAAACCACCGAAAT---GTCTACCACTATGTTCACAT------CCTCCAATGGCAACGTCAAC------------ACATCCACAGGATTCACTGCAAGCTCTGTAAAAGGCACAGACGTGACCTCAACTATTTCCGCCATATCTACCCAAACATCTACAACTAACGTAACTGTAATAACAACTTCACCAAACGGCGA---CACGAATTCATCGACACAGCATGTAACCGATAGTACTGTGACTTTGCAAACTATATCATTAT---CAACCAACAC------TAC------TACTAT---GGT------------AAATGCAAATGAAAACGTCACTACACCGCTTCCAACTTGCTCATCGC---CTAACAG---------------------------------------------------CACAAATAATACTATATCAAAAGAACCTGAAACATTATTGGAGGCGGCACAAGGAGACAATATTACTATAACACACAACCTAACCATCACATCGTGCTACAAAACAGCCTGGCTTAGACATTTTAATATATCCACACACGGAAAATACACTCATCCCAACATAAGAAATGGAAA---ATATTATAACCATTCATTGAAAATCCTCCATTCGCGTATACTATGTGAGTGGCACACAAATTATCTAAAACATCACTATGATTTATGTTTTACATGCGATCGTAATTTATCTTTATCTCTGTACGGTCTTAATTTTACTCATTCTGGTAAATATAGCTTTCGATGTTATAAAACTGGCCATCCCTCCGAACAAA------------ATCAAAACTTTAATCTGCAAATACATCCTAGAAACAACACAAACGGGACACACGTGGATCCCTGGGTATGTGAAGAACCAAAGCACGAATGGGACACTTCTCC---------TCATAAACCGACCAATTATGAAGACAATACAGCCACATCATCTATAGATCATTTATACCGCTATAACAATCATTCTAACACATCACACGGCAGACGTACTACGTGGACGTTAGCATTAATTTGTGTAGCCTGCATTCTCCTATTTTTCGTCCGACGAGCTCTAAATAAAAAATATCATCCATTAAGTGACGATATAAGTGAATCAGAATTCATAGTTCGATACAATCCTGAGCACGAGGACTAA

BE/32/2011 ATGCGCACACAACATCGACGGTGGAACAAGTCATCATATACGCAAATAATATGCATGTTTATTATTTTTTGGATTCTGCAGAAAAGCAAGTGTAACAACACCACTATCGCTAATACTTCCACGTCGATTACACCCACAAGCTTA---------ATATCTACTACACAACTGACATCCACGTTACAAACCACCGAAAT---GTCTACCACTATGTTCACAT------CCTCCAATGGCAACGTCAAC------------ACATCCACAGGATTCACTGCAAGCTCTGTAAAAGGCACAGACGTGACCTCAACTATTTCCGCCATATCTACCCAAACATCTACAACTAACGTAACTGTAATAACAACTTCACCAAACGGCGA---CACGAATTCATCGACACAGCATGTAACCGATAGTACTGTGACTTTGCAAACTATATCATTAT---CAACCAACAC------TAC------TACTAT---GGT------------AAATGCAAATGAAAACGTCACTACACCGCTTCCAACTTGCTCATCGC---CTAACAG---------------------------------------------------CACAAATAATACTATATCAAAAGAACCTGAAACATTATTGGAGGCGGCACAAGGAGACAATATTACTATAACACACAACCTAACCATCACATCGTGCTACAAAACAGCCTGGCTTAGACATTTTAATATATCCACACACGGAAAATACACTCATCCCAACATAAGAAATGGAAA---ATATTATAACCATTCATTGAAAATCCTCCATTCGCGTATACTATGTGAGTGGCACACAAATTATCTAAAACATCACTATGATTTATGTTTTACATGCGATCGTAATTTATCTTTATCTCTGTACGGTCTTAATTTTACTCATTCTGGTAAATATAGCTTTCGATGTTATAAAACTGGCCATCCCTCCGAACAAA------------ATCAAAACTTTAATCTGCAAATACATCCTAGAAACAACACAAACGGGACACACGTGGATCCCTGGGTATGTGAAGAACCAAAGCACGAATGGGACACTTCTCC---------TCATAAACCGACCAATTATGAAGACAATACAGCCACATCATCTATAGATCATTTATACCGCTATAACAATCATTCTAACACATCACACGGCAGACGTACTACGTGGACGTTAGCATTAATTTGTGTAGCCTGCATTCTCCTATTTTTCGTCCGACGAGCTCTAAATAAAAAATATCATCCATTAAGTGACGATATAAGTGAATCAGAATTCATAGTTCGATACAATCCTGAGCACGAGGACTAA

BE/2/2011 ATGCGCACACAACATCGACGGTGGAACAAGTCATCATATACGCAAATAATATGCATGTTTATTATTTTTTGGATTCTGCAGAAAAGCAAGTGTAACAACACCACTATCGCTAATACTTCCACGTCGATTACACCCACAAGCTTA---------ATATCTACTACACAACTGACATCCACGTTACAAACCACCGAAAT---GTCTACCACTATGTTCACAT------CCTCCAATGGCAACGTCAAC------------ACATCCACAGGATTCACTGCAAGCTCTGTAAAAGGCACAGACGTGACCTCAACTATTTCCGCCATATCTACCCAAACATCTACAACTAACGTAACTGTAATAACAACTTCACCAAACGGCGA---CACGAATTCATCGACACAGCATGTAACCGATAGTACTGTGACTTTGCAAACTATATCATTAT---CAACCAACAC------TAC------TACTAT---GGT------------AAATGCAAATGAAAACGTCACTACACCGCTTCCAACTTGCTCATCGC---CTAACAG---------------------------------------------------CACAAATAATACTATATCAAAAGAACCTGAAACATTATTGGAGGCGGCACAAGGAGACAATATTACTATAACACACAACCTAACCATCACATCGTGCTACAAAACAGCCTGGCTTAGACATTTTAATATATCCACACACGGAAAATACACTCATCCCAACATAAGAAATGGAAA---ATATTATAACCATTCATTGAAAATCCTCCATTCGCGTATACTATGTGAGTGGCACACAAATTATCTAAAACATCACTATGATTTATGTTTTACATGCGATCGTAATTTATCTTTATCTCTGTACGGTCTTAATTTTACTCATTCTGGTAAATATAGCTTTCGATGTTATAAAACTGGCCATCCCTCCGAACAAA------------ATCAAAACTTTAATCTGCAAATACATCCTAGAAACAACACAAACGGGACACACGTGGATCCCTGGGTATGTGAAGAACCAAAGCACGAATGGGACACTTCTCC---------TCATAAACCGACCAATTATGAAGACAATACAGCCACATCATCTATAGATCATTTATACCGCTATAACAATCATTCTAACACATCACACGGCAGACGTACTACGTGGACGTTAGCATTAATTTGTGTAGCCTGCATTCTCCTATTTTTCGTCCGACGAGCTCTAAATAAAAAATATCATCCATTAAGTGACGATATAAGTGAATCAGAATTCATAGTTCGATACAATCCTGAGCACGAGGACTAA

BE/34/2011 ATGCGCACACAACATCGACGGTGGAACAAGTCATCATATACGCAAATAATATGCATGTTTATTATTTTTTGGATTCTGCAGAAAAGCAAGTGTAACAACACCACTATCGCTAATACTTCCACGTCGATTACACCCACAAGCTTA---------ATATCTACTACACAACTGACATCCACGTTACAAACCACCGAAAT---GTCTACCACTATGTTCACAT------CCTCCAATGGCAACGTCAAC------------ACATCCACAGGATTCACTGCAAGCTCTGTAAAAGGCACAGACGTGACCTCAACTATTTCCGCCATATCTACCCAAACATCTACAACTAACGTAACTGTAATAACAACTTCACCAAACGGCGA---CACGAATTCATCGACACAGCATGTAACCGATAGTACTGTGACTTTGCAAACTATATCATTAT---CAACCAACAC------TAC------TACTAT---GGT------------AAATGCAAATGAAAACGTCACTACACCGCTTCCAACTTGCTCATCGC---CTAACAG---------------------------------------------------CACAAATAATACTATATCAAAAGAACCTGAAACATTATTGGAGGCGGCACAAGGAGACAATATTACTATAACACACAACCTAACCATCACATCGTGCTACAAAACAGCCTGGCTTAGACATTTTAATATATCCACACACGGAAAATACACTCATCCCAACATAAGAAATGGAAA---ATATTATAACCATTCATTGAAAATCCTCCATTCGCGTATACTATGTGAGTGGCACACAAATTATCTAAAACATCACTATGATTTATGTTTTACATGCGATCGTAATTTATCTTTATCTCTGTACGGTCTTAATTTTACTCATTCTGGTAAATATAGCTTTCGATGTTATAAAACTGGCCATCCCTCCGAACAAA------------ATCAAAACTTTAATCTGCAAATACATCCTAGAAACAACACAAACGGGACACACGTGGATCCCTGGGTATGTGAAGAACCAAAGCACGAATGGGACACTTCTCC---------TCATAAACCGACCAATTATGAAGACAATACAGCCACATCATCTATAGATCATTTATACCGCTATAACAATCATTCTAACACATCACACGGCAGACGTACTACGTGGACGTTAGCATTAATTTGTGTAGCCTGCATTCTCCTATTTTTCGTCCGACGAGCTCTAAATAAAAAATATCATCCATTAAGTGACGATATAAGTGAATCAGAATTCATAGTTCGATACAATCCTGAGCACGAGGACTAA

U8 ATGCGCACACAACATCGACGGTGGAACAAGTCATCATATACGCAAATAATATGCATGTTTATTATTTTTTGGATTCTGCAGAAAAGCAAGTGTAACAACACCACTATCGCTAATACTTCCACGTCGATTACACCCACAAGCTTA---------ATATCTACTACACAACTGACATCCACGTTACAAACCACCGAAAT---GTCTACCACTATGTTCACAT------CCTCCAATGGCAACGTCAAC------------ACATCCACAGGATTCACTGCAAGCTCTGTAAAAGGCACAGACGTGACCTCAACTATTTCCGCCATATCTACCCAAACATCTACAACTAACGTAACTGTAATAACAACTTCACCAAACGGCGA---CACGAATTCATCGACACAGCATGTAACCGATAGTACTGTGACTTTGCAAACTATATCATTAT---CAACCAACAC------TAC------TACTAT---GGT------------AAATGCAAATGAAAACGTCACTACACCGCTTCCAACTTGCTCATCGC---CTAACAG---------------------------------------------------CACAAATAATACTATATCAAAAGAACCTGAAACATTATTGGAGGCGGCACAAGGAGACAATATTACTATAACACACAACCTAACCATCACATCGTGCTACAAAACAGCCTGGCTTAGACATTTTAATATATCCACACACGGAAAATACACTCATCCCAACATAAGAAATGGAAA---ATATTATAACCATTCATTGAAAATCCTCCATTCGCGTATACTATGTGAGTGGCACACAAATTATCTAAAACATCACTATGATTTATGTTTTACATGCGATCGTAATTTATCTTTATCTCTGTACGGTCTTAATTTTACTCATTCTGGTAAATATAGCTTTCGATGTTATAAAACTGGCCATCCCTCCGAACAAA------------ATCAAAACTTTAATCTGCAAATACATCCTAGAAACAACACAAACGGGACACACGTGGATCCCTGGGTATGTGAAGAACCAAAGCACGAATGGGACACTTCTCC---------TCATAAACCGACCAATTATGAAGACAATACAGCCACATCATCTATAGATCATTTATACCGCTATAACAATCATTCTAACACATCACACGGCAGACGTACTACGTGGACGTTAGCATTAATTTGTGTAGCCTGCATTCTCCTATTTTTCGTCCGACGAGCTCTAAATAAAAAATATCATCCATTAAGTGACGATATAAGTGAATCAGAATTCATAGTTCGATACAATCCTGAGCACGAGGACTAA

JER847 ATGCGCACACAACATCGACGGTGGAACAAGTCATCATATACGCAAATAATATGCATGTTTATTATTTTTTGGATTCTGCAGAAAAGCAAGTGTAACAACACCACTATCGCTAATACTTCCACGTCGATTACACCCACAAGCTTA---------ATATCTACTACACAACTGACATCCACGTTACAAACCACCGAAAT---GTCTACCACTATGTTCACAT------CCTCCAATGGCAACGTCAAC------------ACATCCACAGGATTCACTGCAAGCTCTGTAAAAGGCACAGACGTGACCTCAACTATTTCCGCCATATCTACCCAAACATCTACAACTAACGTAACTGTAATAACAACTTCACCAAACGGCGA---CACGAATTCATCGACACAGCATGTAACCGATAGTACTGTGACTTTGCAAACTATATCATTAT---CAACCAACAC------TAC------TACTAT---GGT------------AAATGCAAATGAAAACGTCACTACACCGCTTCCAACTTGCTCATCGC---CTAACAG---------------------------------------------------CACAAATAATACTATATCAAAAGAACCTGAAACATTATTGGAGGCGGCACAAGGAGACAATATTACTATAACACACAACCTAACCATCACATCGTGCTACAAAACAGCCTGGCTTAGACATTTTAATATATCCACACACGGAAAATACACTCATCCCAACATAAGAAATGGAAA---ATATTATAACCATTCATTGAAAATCCTCCATTCGCGTATACTATGTGAGTGGCACACAAATTATCTAAAACATCACTATGATTTATGTTTTACATGCGATCGTAATTTATCTTTATCTCTGTACGGTCTTAATTTTACTCATTCTGGTAAATATAGCTTTCGATGTTATAAAACTGGCCATCCCTCCGAACAAA------------ATCAAAACTTTAATCTGCAAATACATCCTAGAAACAACACAAACGGGACACACGTGGATCCCTGGGTATGTGAAGAACCAAAGCACGAATGGGACACTTCTCC---------TCATAAACCGACCAATTATGAAGACAATACAGCCACATCATCTATAGATCATTTATACCGCTATAACAATCATTCTAACACATCACACGGCAGACGTACTACGTGGACGTTAGCATTAATTTGTGTAGCCTGCATTCTCCTATTTTTCGTCCGACGAGCTCTAAATAAAAAATATCATCCATTAAGTGACGATATAAGTGAATCAGAATTCATAGTTCGATACAATCCTGAGCACGAGGACTAA

BE/46/2011 ATGCGCACACAACATCGACGGTGGAACAAGTCATCATATACGCAAATAATATGCATGTTTATTATTTTTTGGATTCTGCAGAAAAGCAAGTGTAACAACACCACTATCGCTAATACTTCCACGTCGATTACACCCACAAGCTTA---------ATATCTACTACACAACTGACATCCACGTTACAAACCACCGAAAT---GTCTACCACTATGTTCACAT------CCTCCAATGGCAACGTCAAC------------ACATCCACAGGATTCACTGCAAGCTCTGTAAAAGGCACAGACGTGACCTCAACTATTTCCGCCATATCTACCCAAACATCTACAACTAACGTAACTGTAATAACAACTTCACCAAACGGCGA---CACGAATTCATCGACACAGCATGTAACCGATAGTACTGTGACTTTGCAAACTATATCATTAT---CAACCAACAC------TAC------TACTAT---GGT------------AAATGCAAATGAAAACGTCACTACACCGCTTCCAACTTGCTCATCGC---CTAACAG---------------------------------------------------CACAAATAATACTATATCAAAAGAACCTGAAACATTATTGGAGGCGGCACAAGGAGACAATATTACTATAACACACAACCTAACCATCACATCGTGCTACAAAACAGCCTGGCTTAGACATTTTAATATATCCACACACGGAAAATACACTCATCCCAACATAAGAAATGGAAA---ATATTATAACCATTCATTGAAAATCCTCCATTCGCGTATACTATGTGAGTGGCACACAAATTATCTAAAACATCACTATGATTTATGTTTTACATGCGATCGTAATTTATCTTTATCTCTGTACGGTCTTAATTTTACTCATTCTGGTAAATATAGCTTTCGATGTTATAAAACTGGCCATCCCTCCGAACAAA------------ATCAAAACTTTAATCTGCAAATACATCCTAGAAACAACACAAACGGGACACACGTGGATCCCTGGGTATGTGAAGAACCAAAGCACGAATGGGACACTTCTCC---------TCATAAACCGACCAATTATGAAGACAATACAGCCACATCATCTATAGATCATTTATACCGCTATAACAATCATTCTAACACATCACACGGCAGACGTACTACGTGGACGTTAGCATTAATTTGTGTAGCCTGCATTATCCTATTTTTCGTCCGACGAGCTCTAAATAAAAAATATCATCCATTAAGTGACGATATAAGTGAATCAGAATTCATAGTTCGATACAATCCTGAGCACGAGGACTAA

PAV4 ATGCGCACACAACATCGACGGTGGAACAAGTCATCATATACGCAAATAATATGCATGTTTATTATTTTTTGGATTCTGCAGAAAAGCAAGTGTAACAACACCACTATCGCCAATACTTCCACGTCGATTACACCCACAAGCTTA---------ATATCTACTACACAACTGACATCTACGTTACAAACCACCGAAAT---GTCTACCACTATGTTCACAT------CCTCCAATGGCAACGTCAAC------------ACATCCACAGGATTCACTGCAAGCTCTGTAAAAGGCACAGACGTGACCTCAACTATTTCCACCATATCTACCCAAACATCTACAACTAACGTAACTGTAATAACAACTTCACCAAACGGCGA---CACGAATTCATCGACACAGCATGTAACCGATAGTACTGTGACTTTGCAAACTATATCATTAT---CAACCAACAC------TAC------TACTAT---GAT------------AAATGCAAATGAAAACGTCACTACACCGCTTCCAACTTGCTCATCGC---CTAACAG---------------------------------------------------TACAAATAATACGATAGCAAAAGAATCTGAAACATTATTGGAGGCGGCACAAGGAGACAATATTACTATAACACACAACCTAACCATCACATCGTGCTACAAAACAGCCTGGCTTAGACATTTTAATATATCCACACACGGAAAATACACCCATCCCAACATAAGAAATGGAAA---ATATCATAACCATTCATTGAAAATCCTCCATTCGCGTATACTATGTGAGTGGCACACAAATTATCTAAAACATCACTATGATTTATGTTTTACATGCGATCGTAATTTATCTTTATCTCTGTACGGTCTTAATTTTACTCATTCTGGTAAATATAGCTTTCGATGTTATAAAACTGGGCATCCCTCCGAACAAA------------ATCAAAACTTTAATCTGCAAATACATCCTAGAAACAACACAAACGGGACACACGTGAATCCCTGGGTATGTGAAGAACCAAAGCACGAATGGGACACTTC------------TCATAAACCGACCAATTATGAAGACAATACAGCCACATCATCTATAGATCATTTATACCGCTATAACAATCATTCTAACACATCACACGGCAGACGCACTACGTGGACGTTAGCATTAATTTGTGTAGCCTGCATTCTCCTATTTTTCGTCCGACGAGCTCTAAATAAAAAATATCATCCATTAAGTGACGATATAAGTGAATCAGAATTCATAGTTCGATACAATCCTGAGCACGAGGACTAA

BE/22/2010 ATGCGCACACAACATCGACGGTGGAACAAGTCATCATATACGCAAATAATATGCATGTTTATTATTTTTTGGATTCTGCAGAAAAGCAAGTGTAACAACACCACTATCGCCAATACTTCCACGTCGATTACACCCACAAGCTTA---------ATATCTACTACACAACTGACATCTACGTTACAAACCACCGAAAT---GTCTACCACTATGTTCACAT------CCTCCAATGGCAACGTCAAC------------ACATCCACAGGATTCACTGCAAGCTCTGTAAAAGGCACAGACGTGACCTCAACTATTTCCACCATATCTACCCAAACATCTACAACTAACGTAACTGTAATAACAACTTCACCAAACGGCGA---CACGAATTCATCGACACAGCATGTAACCGATAGTACTGTGACTTTGCAAACTATATCATTAT---CAACCAACAC------TAC------TACTAT---GAT------------AAATGCAAATGAAAACGTCACTACACCGCTTCCAACTTGCTCATCGC---CTAACAG---------------------------------------------------TACAAATAATACGATAGCAAAAGAATCTGAAACATTATTGGAGGCGGCACAAGGAGACAATATTACTATAACACACAACCTAACCATCACATCGTGCTACAAAACAGCCTGGCTTAGACATTTTAATATATCCACACACGGAAAATACACCCATCCCAACATAAGAAATGGAAA---ATATCATAACCATTCATTGAAAATCCTCCATTCGCGTATACTATGTGAGTGGCACACAAATTATCTAAAACATCACTATGATTTATGTTTTACATGCGATCGTAATTTATCTTTATCTCTGTACGGTCTTAATTTTACTCATTCTGGTAAATATAGCTTTCGATGTTATAAAACTGGGCATCCCTCCGAACAAA------------ATCAAAACTTTAATCTGCAAATACATCCTAGAAACAACACAAACGGGACACACGTGAATCCCTGGGTATGTGAAGAACCAAAGCACGAATGGGACACTTC------------TCATAAACCGACCAATTATGAAGACAATACAGCCACATCATCTATAGATCATTTATACCGCTATAACAATCATTCTAACACATCACACGGCAGACGCACTACGTGGACGTTAGCATTAATTTGTGTAGCCTGCATTCTCCTATTTTTCGTCCGACGAGCTCTAAATAAAAAATATCATCCATTAAGTGACGATATAAGTGAATCAGAATTCATAGTTCGATACAATCCTGAGCACGAGGACTAA

PAV7 ATGCGCACACAACATCGACGGTGGAACAAGTCATCATATACGCAAATAATATGCATGTTTATTATTTTTTGGATTCTGCAGAAAAGCAAGTGTAACAACACCACTATCGCCAATACTTCCACGTCGATTACACCCACAAGCTTA---------ATATCTACTACACAACTGACATCTACGTTACAAACCACCGAAAT---GTCTACCACTATGTTCACAT------CCTCCAATGGCAACGTCAAC------------ACATCCACAGGATTCACTGCAAGCTCTGTAAAAGGCACAGACGTGACCTCAACTATTTCCACCATATCTACCCAAACATCTACAACTAACGTAACTGTAATAACAACTTCACCAAACGGCGA---CACGAATTCATCGACACAGCATGTAACCGATAGTACTGTGACTTTGCAAACTATATCATTAT---CAACCAACAC------TAC------TACTAT---GAT------------AAATGCAAATGAAAACGTCACTACACCGCTTCCAACTTGCTCATCGC---CTAACAG---------------------------------------------------TACAAATAATACGATAGCAAAAGAATCTGAAACATTATTGGAGGCGGCACAAGGAGACAATATTACTATAACACACAACCTAACCATCACATCGTGCTACAAAACAGCCTGGCTTAGACATTTTAATATATCCACACACGGAAAATACACCCATCCCAACATAAGAAATGGAAA---ATATCATAACCATTCATTGAAAATCCTCCATTCGCGTATACTATGTGAGTGGCACACAAATTATCTAAAACATCACTATGATTTATGTTTTACATGCGATCGTAATTTATCTTTATCTCTGTACGGTCTTAATTTTACTCATTCTGGTAAATATAGCTTTCGATGTTATAAAACTGGGCATCCCTCCGAACAAA------------ATCAAAACTTTAATCTGCAAATACATCCTAGAAACAACACAAACGGGACACACGTGAATCCCTGGGTATGTGAAGAACCAAAGCACGAATGGGACACTTC------------TCATAAACCGACCAATTATGAAGACAATACAGCCACATCATCTATAGATCATTTATACCGCTATAACAATCATTCTAACACATCACACGGCAGACGCACTACGTGGACGTTAGCATTAATTTGTGTAGCCTGCATTCTCCTATTTTTCGTCCGACGAGCTCTAAATAAAAAATATCATCCATTAAGTGACGATATAAGTGAATCAGAATTCATAGTTCGATACAATCCTGAGCACGAGGACTAA

Pat_E ATGCGCACACAACATCGACGGTGGAACAAGTCATCATATACGCAAATAATATGCATGTTTATTATTTTTTGGATTCTGCAGAAAAGCAAGTGTAACAACACCACTATCGCCAATACTTCCACGTCGATTACACCCACAAGCTTA---------ATATCTACTACACAACTGACATCTACGTTACAAACCACCGAAAT---GTCTACCACTATGTTCACAT------CCTCCAATGGCAACGTCAAC------------ACATCCACAGGATTCACTGCAAGCTCTGTAAAAGGCACAGACGTGACCTCAACTATTTCCACCATATCTACCCAAACATCTACAACTAACGTAACTGTAATAACAACTTCACCAAACGGCGA---CACGAATTCATCGACACAGCATGTAACCGATAGTACTGTGACTTTGCAAACTATATCATTAT---CAACCAACAC------TAC------TACTAT---GAT------------AAATGCAAATGAAAACGTCACTACACCGCTTCCAACTTGCTCATCGC---CTAACAG---------------------------------------------------TACAAATAATACGATAGCAAAAGAATCTGAAACATTATTGGAGGCGGCACAAGGAGACAATATTACTATAACACACAACCTAACCATCACATCGTGCTACAAAACAGCCTGGCTTAGACATTTTAATATATCCACACACGGAAAATACACCCATCCCAACATAAGAAATGGAAA---ATATCATAACCATTCATTGAAAATCCTCCATTCGCGTATACTATGTGAGTGGCACACAAATTATCTAAAACATCACTATGATTTATGTTTTACATGCGATCGTAATTTATCTTTATCTCTGTACGGTCTTAATTTTACTCATTCTGGTAAATATAGCTTTCGATGTTATAAAACTGGGCATCCCTCCGAACAAA------------ATCAAAACTTTAATCTGCAAATACATCCTAGAAACAACACAAACGGGACACACGTGAATCCCTGGGTATGTGAAGAACCAAAGCACGAATGGGACACTTC------------TCATAAACCGACCAATTATGAAGACAATACAGCCACATCATCTATAGATCATTTATACCGCTATAACAATCATTCTAACACATCACACGGCAGACGCACTACGTGGACGTTAGCATTAATTTGTGTAGCCTGCATTCTCCTATTTTTCGTCCGACGAGCTCTAAATAAAAAATATCATCCATTAAGTGACGATATAAGTGAATCAGAATTCATAGTTCGATACAATCCTGAGCACGAGGACTAA

PAV11 ATGCGCACACAACATCGACGGTGGAACAAGTCATCATATACGCAAATAATATGCATGTTTATTATTTTTTGGATTCTGCAGAAAAGCAAGTGTAACAACACCACTATCGCCAATACTTCCACGTCGATTACACCCACAAGCTTA---------ATATCTACTACACAACTGACATCTACGTTACAAACCACCGAAAT---GTCTACCACTATGTTCACAT------CCTCCAATGGCAACGTCAAC------------ACATCCACAGGATTCACTGCAAGCTCTGTAAAAGGCACAGACGTGACCTCAACTATTTCCACCATATCTACCCAAACATCTACAACTAACGTAACTGTAATAACAACTTCACCAAACGGCGA---CACGAATTCATCGACACAGCATGTAACCGATAGTACTGTGACTTTGCAAACTATATCATTAT---CAACCAACAC------TAC------TACTAT---GAT------------AAATGCAAATGAAAACGTCACTACACCGCTTCCAACTTGCTCATCGC---CTAACAG---------------------------------------------------TACAAATAATACGATATCAAAAGAATCTGAAACATTATTGGAGGCGGCACAAGGAGACAATATTACTATAACACACAACCTAACCATCACATCGTGCTACAAAACAGCCTGGCTTAGACATTTTAATATATCCACACACGGAAAATACACCCATCCCAACATAAGAAATGGAAA---ATATCATAACCATTCATTGAAAATCCTCCATTCGCGTATACTATGTGAGTGGCACACAAATTATCTAAAACATCACTATGATTTATGTTTTACATGCGATCGTAATTTATCTTTATCTCTGTACGGTCTTAATTTTACTCATTCTGGTAAATATAGCTTTCGATGTTATAAAACTGGGCATCCCTCCGAACAAA------------ATCAAAACTTTAATCTGCAAATACATCCTAGAAACAACACAAACGGGACACACGTGAATCCCTGGGTATGTGAAGAACCAAAGCACGAATGGGACACTTC------------TCATAAACCGACCAATTATGAAGACAATACAGCCACATCATCTATAGATCATTTATACCGCTATAACAATCATTCTAACACATCACACGGCAGACGCACTACGTGGACGTTAGCATTAATTTGTGTAGCCTGCATTCTCCTATTTTTCGTCCGACGAGCTCTAAATAAAAAATATCATCCATTAAGTGACGATATAAGTGAATCAGAATTCATAGTTCGATACAATCCTGAGCACGAGGACTAA

BE/19/2011 ATGCGCACACAACATCGACGGTGGAACAAGTCATCATATACGCAAATAATATGCATGTTTATTATTTTTTGGATTCTGCAGAAAAGCAAGTGTAACAACACCACTATCGCCAATACTTCCACGTCGATTACACCCACAAGCTTA---------ATATCTACTACACAACTGACATCTACGTTACAAACCACCGAAAT---GTCTACCACTATGTTCACAT------CCTCCAATGGCAACGTCAAC------------ACATCCACAGGATTCACTGCAAGCTCTGTAAAAGGCACAGACGTGACCTCAACTATTTCCACCATATCTACCCAAACATCTACAACTAACGTAACTGTAATAACAACTTCACCAAACGGCGA---CACGAATTCATCGACACAGCATGTAACCGATAGTACTGTGACTTTGCAAACTATATCATTAT---CAACCAACAC------TAC------TACTAT---GAT------------AAATGCAAATGAAAACGTCACTACACCGCTTCCAACTTGCTCATCGC---CTAACAG---------------------------------------------------TACAAATAATACGATATCAAAAGAATCTGAAACATTATTGGAGGCGGCACAAGGAGACAATATTACTATAACACACAACCTAACCATCACATCGTGCTACAAAACAGCCTGGCTTAGACATTTTAATATATCCACACACGGAAAATACACCCATCCCAACATAAGAAATGGAAA---ATATCATAACCATTCATTGAAAATCCTCCATTCGCGTATACTATGTGAGTGGCACACAAATTATCTAAAACATCACTATGATTTATGTTTTACATGCGATCGTAATTTATCTTTATCTCTGTACGGTCTTAATTTTACTCATTCTGGTAAATATAGCTTTCGATGTTATAAAACTGGGCATCCCTCCGAACAAA------------ATCAAAACTTTAATCTGCAAATACATCCTAGAAACAACACAAACGGGACACACGTGAATCCCTGGGTATGTGAAGAACCAAAGCACGAATGGGACACTTC------------TCATAAACCGACCAATTATGAAGACAATACAGCCACATCATCTATAGATCATTTATACCGCTATAACAATCATTCTAACACATCACACGGCAGACGCACTACGTGGACGTTAGCATTAATTTGTGTAGCCTGCATTCTCCTATTTTTCGTCCGACGAGCTCTAAATAAAAAATATCATCCATTAAGTGACGATATAAGTGAATCAGAATTCATAGTTCGATACAATCCTGAGCACGAGGACTAA

HAN17 ATGCGCACACAACATCGACGGTGGAACAAGTCATCATATACGCAAATAATATGCATGTTTATTATTTTTTGGATTCTGCAGAAAAGCAAGTGTAACAACACCACTATCGCCAATACTTCCACGTCGATTACACCCACAAGCTTA---------ATATCTACTACACAACTGACATCTACGTTACAAACCACCGAAAT---GTCTACCACTATGTTCACAT------CCTCCAATGGCAACGTCAAC------------ACATCCACAGGATTCACTGCAAGCTCTGTAAAAGGCACAGACGTGACCTCAACTATTTCCACCATATCTACCCAAACATCTACAACTAACGTAACTGTAATAACAACTTCACCAAACGGCGA---CACGAATTCATCGACACAGCATGTAACCGATAGTACTGTGACTTTGCAAACTATATCATTAT---CAACCAACAC------TAC------TACTAT---GAT------------AAATGCAAATGAAAACGTCACTACACCGCTTCCAACTTGCTCATCGC---CTAACAG---------------------------------------------------TACAAATAATACGATATCAAAAGAATCTGAAACATTATTGGAGGCGGCACAAGGAGACAATATTACTATAACACACAACCTAACCATCACATCGTGCTACAAAACAGCCTGGCTTAGACATTTTAATATATCCACACACGGAAAATACACCCATCCCAACATAAGAAATGGAAA---ATATCATAACCATTCATTGAAAATCCTCCATTCGCGTATACTATGTGAGTGGCACACAAATTATCTAAAACATCACTATGATTTATGTTTTACATGCGATCGTAATTTATCTTTATCTCTGTACGGTCTTAATTTTACTCATTCTGGTAAATATAGCTTTCGATGTTATAAAACTGGGCATCCCTCCGAACAAA------------ATCAAAACTTTAATCTGCAAATACATCCTAGAAACAACACAAACGGGACACACGTGAATCCCTGGGTATGTGAAGAACCAAAGCACGAATGGGACACTTC------------TCATAAACCGACCAATTATGAAGACAATACAGCCACATCATCTATAGATCATTTATACCGCTATAACAATCATTCTAACACATCACACGGCAGACGCACTACGTGGACGTTAGCATTAATTTGTGTAGCCTGCATTCTCCTATTTTTCGTCCGACGAGCTCTAAATAAAAAATATCATCCATTAAGTGACGATATAAGTGAATCAGAATTCATAGTTCGATACAATCCTGAGCACGAGGACTAA

BE/14/2010 ATGCGCACACAACATCGACGGTGGAACAAGTCATCATATACGCAAATAATATGCATGTTTATTATTTTTTGGATTCTGCAGAAAAGCAAGTGTAACAACACCACTATCGCCAATACTTCCACGTCGATTACACCCACAAGCTTA---------ATATCTACTACACAACTGACATCTACGTTACAAACCACCGAAAT---GTCTACCACTATGTTCACAT------CCTCCAATGGCAACGTCAAC------------ACATCCACAGGATTCACTGCAAGCTCTGTAAAAGGCACAGACGTGACCTCAACTATTTCCACCATATCTACCCAAACATCTACAACTAACGTAACTGTAATAACAACTTCACCAAACGGCGA---CACGAATTCATCGACACAGCATGTAACCGATAGTACTGTGACTTTGCAAACTATATCATTAT---CAACCAACAC------TAC------TACTAT---GAT------------AAATGCAAATGAAAACGTCACTACACCGCTTCCAACTTGCTCATCGC---CTAACAG---------------------------------------------------TACAAATAATACGATATCAAAAGAATCTGAAACATTATTGGAGGCGGCACAAGGAGACAATATTACTATAACACACAACCTAACCATCACATCGTGCTACAAAACAGCCTGGCTTAGACATTTTAATATATCCACACACGGAAAATACACCCATCCCAACATAAGAAATGGAAA---ATATCATAACCATTCATTGAAAATCCTCCATTCGCGTATACTATGTGAGTGGCACACAAATTATCTAAAACATCACTATGATTTATGTTTTACATGCGATCGTAATTTATCTTTATCTCTGTACGGTCTTAATTTTACTCATTCTGGTAAATATAGCTTTCGATGTTATAAAACTGGGCATCCCTCCGAACAAA------------ATCAAAACTTTAATCTGCAAATACATCCTAGAAACAACACAAACGGGACACACGTGAATCCCTGGGTATGTGAAGAACCAAAGCACGAATGGGACACTTC------------TCATAAACCGACCAATTATGAAGACAATACAGCCACATCATCTATAGATCATTTATACCGCTATAACAATCATTCTAACACATCACACGGCAGACGCACTACGTGGACGTTAGCATTAATTTGTGTAGCCTGCATTCTCCTATTTTTCGTCCGACGAGCTCTAAATAAAAAATATCATCCATTAAGTGACGATATAAGTGAATCAGAATTCATAGTTCGATACAATCCTGAGCACGAGGACTAA

VR1814 ATGCGCACACAACATCGACGGTGGAACAAGTCATCATATACGCAAATAATATGCATGTTTATTATTTTTTGGATTCTGCAGAAAAGCAAGTGTAACAACACCACTATCGCCAATACTTCCACGTCGATTACACCCACAAGCTTA---------ATATCTACTACACAACTGACATCTACGTTACAAACCACCGAAAT---GTCTACCACTATGTTCACAT------CCTCCAATGGCAACGTCAAC------------ACATCCACAGGATTCACTGCAAGCTCTGTAAAAGGCACAGACGTGACCTCAACTATTTCCACCATATCTACCCAAACATCTACAACTAACGTAACTGTAATAACAACTTCACCAAACGGCGA---CACGAATTCATCGACACAGCATGTAACCGATAGTACTGTGACTTTGCAAACTATATCATTAT---CAACCAACAC------TAC------TACTAT---GAT------------AAATGCAAATGAAAACGTCACTACACCGCTTCCAACTTGCTCATCGC---CTAACAG---------------------------------------------------TACAAATAATACGATATCAAAAGAATCTGAAACATTATTGGAGGCGGCACAAGGAGACAATATTACTATAACACACAACCTAACCATCACATCGTGCTACAAAACAGCCTGGCTTAGACATTTTAATATATCCACACACGGAAAATACACCCATCCCAACATAAGAAATGGAAA---ATATCATAACCATTCATTGAAAATCCTCCATTCGCGTATACTATGTGAGTGGCACACAAATTATCTAAAACATCACTATGATTTATGTTTTACATGCGATCGTAATTTATCTTTATCTCTGTACGGTCTTAATTTTACTCATTCTGGTAAATATAGCTTTCGATGTTATAAAACTGGGCATCCCTCCGAACAAA------------ATCAAAACTTTAATCTGCAAATACATCCTAGAAACAACACAAACGGGACACACGTGAATCCCTGGGTATGTGAAGAACCAAAGCACGAATGGGACACTTC------------TCATAAACCGACCAATTATGAAGACAATACAGCCACATCATCTATAGATCATTTATACCGCTATAACAATCATTCTAACACATCACACGGCAGACGCACTACGTGGACGTTAGCATTAATTTGTGTAGCCTGCATTCTCCTATTTTTCGTCCGACGAGCTCTAAATAAAAAATATCATCCATTAAGTGACGATATAAGTGAATCAGAATTCATAGTTCGATACAATCCTGAGCACGAGGACTAA

BE/9/2011 ATGCGCACACAACATCGACGGTGGAACAAGTCATCATATACGCAAATAATATGCATGTTTATTATTTTTTGGATTCTGCAGAAAAGCAAGTGTAACAACACCACTATCGCCAATACTTCCACGTCGATTACACCCACAAGCTTA---------ATATCTACTACACAACTGACATCTACGTTACAAACCACCGAAAT---GTCTACCACTATGTTCACAT------CCTCCAATGGCAACGTCAAC------------ACATCCACAGGATTCACTGCAAGCTCTGTAAAAGGCACAGACGTGACCTCAACTATTTCCACCATATCTACCCAAACATCTACAACTAACGTAACTGTAATAACAACTTCACCAAACGGCGA---CACGAATTCATCGACACAGCATGTAACCGATAGTACTGTGACTTTGCAAACTATATCATTAT---CAACCAACAC------TAC------TACTAT---GAT------------AAATGCAAATGAAAACGTCACTACACCGCTTCCAACTTGCTCATCGC---CTAACAG---------------------------------------------------TACAAATAATACGATATCAAAAGAATCTGAAACATTATTGGAGGCGGCACAAGGAGACAATATTACTATAACACACAACCTAACCATCACATCGTGCTACAAAACAGCCTGGCTTAGACATTTTAATATATCCACACACGGAAAATACACCCATCCCAACATAAGAAATGGAAA---ATATCATAACCATTCATTGAAAATCCTCCATTCGCGTATACTATGTGAGTGGCACACAAATTATCTAAAACATCACTATGATTTATGTTTTACATGCGATCGTAATTTATCTTTATCTCTGTACGGTCTTAATTTTACTCATTCTGGTAAATATAGCTTTCGATGTTATAAAACTGGGCATCCCTCCGAACAAA------------ATCAAAACTTTAATCTGCAAATACATCCTAGAAACAACACAAACGGGACACACGTGAATCCCTGGGTATGTGAAGAACCAAAGCACGAATGGGACACTTC------------TCATAAACCGACCAATTATGAAGACAATACAGCCACATCATCTATAGATCATTTATACCGCTATAACAATCATTCTAACACATCACACGGCAGACGCACTACGTGGACGTTAGCATTAATTTGTGTAGCCTGCATTCTCCTATTTTTCGTCCGACGAGCTCTAAATAAAAAATATCATCCATTAAGTGACGATATAAGTGAATCAGAATTCATAGTTCGATACAATCCTGAGCACGAGGACTAA

Towne ATGCGCACACAACATCGACGGTGGAACAAGTCATCATATACGCAAATAATATGCATGTTTATTATTTTTTGGATTCTGCAGAAAAGCAAGTGTAACAACACCACTATCGCCAATACTTCCACGTCGATTACACCCACAAGCTTA---------ATATCTACTACACAACTGACATCTACGTTACAAACCACCGAAAT---GTCTACCACTATGTTCACAT------CCTCCAATGGCAACGTCAAC------------ACATCCACAGGATTCACTGCAAGCTCTGTAAAAGGCACAGACGTGACCTCAACTATTTCCACCATATCTACCCAAACATCTACAACTAACGTAACTGTAATAACAACTTCACCAAACGGCGA---CACGAATTCATCGACACAGCATGTAACCGATAGTACTGTGACTTTGCAAACTATATCATTAT---CAACCAACAC------TAC------TACTAT---GAT------------AAATGCAAATGAAAACGTCACTACACCGCTTCCAACTTGCTCATCGC---CTAACAG---------------------------------------------------TACAAATAATACGATATCAAAAGAATCTGAAACATTATTGGAGGCGGCACAAGGAGACAATATTACTATAACACACAACCTAACCATCACATCGTGCTACAAAACAGCCTGGCTTAGACATTTTAATATATCCACACACGGAAAATACACCCATCCCAACATAAGAAATGGAAA---ATATCATAACCATTCATTGAAAATCCTCCATTCGCGTATACTATGTGAGTGGCACACAAATTATCTAAAACATCACTATGATTTATGTTTTACATGCGATCGTAATTTATCTTTATCTCTGTACGGTCTTAATTTTACTCATTCTGGTAAATATAGCTTTCGATGTTATAAAACTGGGCATCCCTCCGAACAAA------------ATCAAAACTTTAATCTGCAAATACATCCTAGAAACAACACAAACGGGACACACGTGAATCCCTGGGTATGTGAAGAACCAAAGCACGAATGGGACACTTC------------TCATAAACCGACCAATTATGAAGACAATACAGCCACATCATCTATAGATCATTTATACCGCTATAACAATCATTCTAACACATCACACGGCAGACGCACTACGTGGACGTTAGCATTAATTTGTGTAGCCTGCATTCTCCTATTTTTCGTCCGACGAGCTCTAAATAAAAAATATCATCCATTAAGTGACGATATAAGTGAATCAGAATTCATAGTTCGATACAATCCTGAGCACGAGGACTAA

6397 ATGCGCACACAACATCGACGGTGGAACAAGTCATCATATACGCAAATAATATGCATGTTTATTATTTTTTGGATTCTGCAGAAAAGCAAGTGTAACAACACCACTATCGCCAATACTTCCACGTCGATTACACCCACAAGCTTA---------ATATCTACTACACAACTGACATCTACGTTACAAACCACCGAAAT---GTCTACCACTATGTTCACAT------CCTCCAATGGCAACGTCAAC------------ACATCCACAGGATTCACTGCAAGCTCTGTAAAAGGCACAGACGTGACCTCAACTATTTCCACCATATCTACCCAAACATCTACAACTAACGTAACTGTAATAACAACTTCACCAAACGGCGA---CACGAATTCATCGACACAGCATGTAACCGATAGTACTGTGACTTTGCAAACTATATCATTAT---CAACCAACAC------TAC------TACTAT---GAT------------AAATGCAAATGAAAACGTCACTACACCGCTTCCAACTTGCTCATCGC---CTAACAG---------------------------------------------------TACAAATAATACGATATCAAAAGAATCTGAAACATTATTGGAGGCGGCACAAGGAGACAATATTACTATAACACACAACCTAACCATCACATCGTGCTACAAAACAGCCTGGCTTAGACATTTTAATATATCCACACACGGAAAATACACCCATCCCAACATAAGAAATGGAAA---ATATCATAACCATTCATTGAAAATCCTCCATTCGCGTATACTATGTGAGTGGCACACAAATTATCTAAAACATCACTATGATTTATGTTTTACATGCGATCGTAATTTATCTTTATCTCTGTACGGTCTTAATTTTACTCATTCTGGTAAATATAGCTTTCGATGTTATAAAACTGGGCATCCCTCCGAACAAA------------ATCAAAACTTTAATCTGCAAATACATCCTAGAAACAACACAAACGGGACACACGTGAATCCCTGGGTATGTGAAGAACCAAAGCACGAATGGGACACTTC------------TCATAAACCGACCAATTATGAAGACAATACAGCCACATCATCTATAGATCATTTATACCGCTATAACAATCATTCTAACACATCACACGGCAGACGCACTACGTGGACGTTAGCATTAATTTGTGTAGCCTGCATTCTCCTATTTTTCGTCCGACGAGCTCTAAATAAAAAATATCATCCATTAAGTGACGATATAAGTGAATCAGAATTCATAGTTCGATACAATCCTGAGCACGAGGACTAA

U11 ATGCGCACACAACATCGACGGTGGAACAAGTCATCATATACGCAAATAATATGCATGTTTATTATTTTTTGGATTCTGCAGAAAAGCAAGTGTAACAACACCACTATCGCCAATACTTCCACGTCGATTACACCCACAAGCTTA---------ATATCTACTACACAACTGACATCTACGTTACAAACCACCGAAAT---GTCTACCACTATGTTCACAT------CCTCCAATGGCAACGTCAAC------------ACATCCACAGGATTCACTGCAAGCTCTGTAAAAGGCACAGACGTGACCTCAACTATTTCCACCATATCTACCCAAACATCTACAACTAACGTAACTGTAATAACAACTTCACCAAACGGCGA---CACGAATTCATCGACACAGCATGTAACCGATAGTACTGTGACTTTGCAAACTATATCATTAT---CAACCAACAC------TAC------TACTAT---GAT------------AAATGCAAATGAAAACGTCACTACACCGCTTCCAACTTGCTCATCGC---CTAACAG---------------------------------------------------TACAAATAATACGATATCAAAAGAATCTGAAACATTATTGGAGGCGGCACAAGGAGACAATATTACTATAACACACAACCTAACCATCACATCGTGCTACAAAACAGCCTGGCTTAGACATTTTAATATATCCACACACGGAAAATACACCCATCCCAACATAAGAAATGGAAA---ATATCATAACCATTCATTGAAAATCCTCCATTCGCGTATACTATGTGAGTGGCACACAAATTATCTAAAACATCACTATGATTTATGTTTTACATGCGATCGTAATTTATCTTTATCTCTGTACGGTCTTAATTTTACTCATTCTGGTAAATATAGCTTTCGATGTTATAAAACTGGGCATCCCTCCGAACAAA------------ATCAAAACTTTAATCTGCAAATACATCCTAGAAACAACACAAACGGGACACACGTGAATCCCTGGGTATGTGAAGAACCAAAGCACGAATGGGACACTTC------------TCATAAACCGACCAATTATGAAGACAATACAGCCACATCATCTATAGATCATTTATACCGCTATAACAATCATTCTAACACATCACACGGCAGACGCACTACGTGGACGTTAGCATTAATTTGTGTAGCCTGCATTCTCCTATTTTTCGTCCGACGAGCTCTAAATAAAAAATATCATCCATTAAGTGACGATATAAGTGAATCAGAATTCATAGTTCGATACAATCCTGAGCACGAGGACTAA

JER1289 ATGCGCACACAACATCGACGGTGGAACAAGTCATCATATACGCAAATAATATGCATGTTTATTATTTTTTGGATTCTGCAGAAAAGCAAGTGTAACAACACCACTATCGCCAATACTTCCACGTCGATTACACCCACAAGCTTA---------ATATCTACTACACAACTGACATCTACGTTACAAACCACCGAAAT---GTCTACCACTATGTTCACAT------CCTCCAATGGCAACGTCAAC------------ACATCCACAGGATTCACTGCAAGCTCTGTAAAAGGCACAGACGTGACCTCAACTATTTCCACCATATCTACCCAAACATCTACAACTAACGTAACTGTAATAACAACTTCACCAAACGGCGA---CACGAATTCATCGACACAGCATGTAACCGATAGTACTGTGACTTTGCAAACTATATCATTAT---CAACCAACAC------TAC------TACTAT---GAT------------AAATGCAAATGAAAACGTCACTACACCGCTTCCAACTTGCTCATCGC---CTAACAG---------------------------------------------------TACAAATAATACGATATCAAAAGAATCTGAAACATTATTGGAGGCGGCACAAGGAGACAATATTACTATAACACACAACCTAACCATCACATCGTGCTACAAAACAGCCTGGCTTAGACATTTTAATATATCCACACACGGAAAATACACCCATCCCAACATAAGAAATGGAAA---ATATCATAACCATTCATTGAAAATCCTCCATTCGCGTATACTATGTGAGTGGCACACAAATTATCTAAAACATCACTATGATTTATGTTTTACATGCGATCGTAATTTATCTTTATCTCTGTACGGTCTTAATTTTACTCATTCTGGTAAATATAGCTTTCGATGTTATAAAACTGGGCATCCCTCCGAACAAA------------ATCAAAACTTTAATCTGCAAATACATCCTAGAAACAACACAAACGGGACACACGTGAATCCCTGGGTATGTGAAGAACCAAAGCACGAATGGGACACTTC------------TCATAAACCGACCAATTATGAAGACAATACAGCCACATCATCTATAGATCATTTATACCGCTATAACAATCATTCTAACACATCACACGGCAGACGCACTACGTGGACGTTAGCATTAATTTGTGTAGCCTGCATTCTCCTATTTTTCGTCCGACGAGCTCTAAATAAAAAATATCATCCATTAAGTGACGATATAAGTGAATCAGAATTCATAGTTCGATACAATCCTGAGCACGAGGACTAA

HANSCTR11B ATGCGCACACAACATCGACGGTGGAACAAGTCATCATATACGCAAATAATATGCATGTTTATTATTTTTTGGATTCTGCAGAAAAGCAAGTGTAACAACACCACTATCGCCAATACTTCCACGTCGATTACACCCACAAGCTTA---------ATATCTACTACACAACTGACATCTACGTTACAAACCACCGAAAT---GTCTACCACTATGTTCACAT------CCTCCAATGGCAACGTCAAC------------ACATCCACAGGATTCACTGCAAGCTCTGTAAAAGGCACAGACGTGACCTCAACTATTTCCACCATATCTACCCAAACATCTACAACTAACGTAACTGTAATAACAACTTCACCAAACGGCGA---CACGAATTCATCGACACAGCATGTAACCGATAGTACTGTGACTTTGCAAACTATATCATTAT---CAACCAACAC------TAC------TACTAT---GAT------------AAATGCAAATGAAAACGTCACTACACCGCTTCCAACTTGCTCATCGC---CTAACAG---------------------------------------------------TACAAATAATACGATATCAAAAGAATCTGAAACATTATTGGAGGCGGCACAAGGAGACAATATTACTATAACACACAACCTAACCATCACATCGTGCTACAAAACAGCCTGGCTTAGACATTTTAATATATCCACACACGGAAAATACACCCATCCCAACATAAGAAATGGAAA---ATATCATAACCATTCATTGAAAATCCTCCATTCGCGTATACTATGTGAGTGGCACACAAATTATCTAAAACATCACTATGATTTATGTTTTACATGCGATCGTAATTTATCTTTATCTCTGTACGGTCTTAATTTTACTCATTCTGGTAAATATAGCTTTCGATGTTATAAAACTGGGCATCCCTCCGAACAAA------------ATCAAAACTTTAATCTGCAAATACATCCTAGAAACAACACAAACGGGACACACGTGAATCCCTGGGTATGTGAAGAACCAAAGCACGAATGGGACACTTC------------TCATAAACCGACCAATTATGAAGACAATACAGCCACATCATCTATAGATCATTTATACCGCTATAACAATCATTCTAACACATCACACGGCAGACGCACTACGTGGACGTTAGCATTAATTTGTGTAGCCTGCATTCTCCTATTTTTCGTCCGACGAGCTCTAAATAAAAAATATCATCCATTAAGTGACGATATAAGTGAATCAGAATTCATAGTTCGATACAATCCTGAGCACGAGGACTAA

HANSCTR1A ATGCGCACACAACATCGACGGTGGAACAAGTCATCATATACGCAAATAATATGCATGTTTATTATTTTTTGGATTCTGCAGAAAAGCAAGTGTAACAACACCACTATCGCCAATACTTCCACGTCGATTACACCCACAAGCTTA---------ATATCTACTACACAACTGACATCTACGTTACAAACCACCGAAAT---GTCTACCACTATGTTCACAT------CCTCCAATGGCAACGTCAAC------------ACATCCACAGGATTCACTGCAAGCTCTGTAAAAGGCACAGACGTGACCTCAACTATTTCCACCATATCTACCCAAACATCTACAACTAACGTAACTGTAATAACAACTTCACCAAACGGCGA---CACGAATTCATCGACACAGCATGTAACCGATAGTACTGTGACTTTGCAAACTATATCATTAT---CAACCAACAC------TAC------TACTAT---GAT------------AAATGCAAATGAAAACGTCACTACACCGCTTCCAACTTGCTCATCGC---CTAACAG---------------------------------------------------TACAAATAATACGATATCAAAAGAATCTGAAACATTATTGGAGGCGGCACAAGGAGACAATATTACTATAACACACAACCTAACCATCACATCGTGCTACAAAACAGCCTGGCTTAGACATTTTAATATATCCACACACGGAAAATACACCCATCCCAACATAAGAAATGGAAA---ATATCATAACCATTCATTGAAAATCCTCCATTCGCGTATACTATGTGAGTGGCACACAAATTATCTAAAACATCACTATGATTTATGTTTTACATGCGATCGTAATTTATCTTTATCTCTGTACGGTCTTAATTTTACTCATTCTGGTAAATATAGCTTTCGATGTTATAAAACTGGGCATCCCTCCGAACAAA------------ATCAAAACTTTAATCTGCAAATACATCCTAGAAACAACACAAACGGGACACACGTGAATCCCTGGGTATGTGAAGAACCAAAGCACGAATGGGACACTTC------------TCATAAACCGACCAATTATGAAGACAATACAGCCACATCATCTATAGATCATTTATACCGCTATAACAATCATTCTAACACATCACACGGCAGACGCACTACGTGGACGTTAGCATTAATTTGTGTAGCCTGCATTCTCCTATTTTTCGTCCGACGAGCTCTAAATAAAAAATATCATCCATTAAGTGACGATATAAGTGAATCAGAATTCATAGTTCGATACAATCCTGAGCACGAGGACTAA

BE/26/2011 ATGCGTACACAACATCGACGGTGGAACAAGTCATCATATACGCAAATAATATGCATGTTTATTATTTTTTGGATTCTGCAGAAAAGCAAGTGTAACAACACCACTATCGTTAATACTTCCACGTCGATTACACCCACAAGCTTA---------ATATCTACTACACAACTGACATCTAAGTTACAAACCACCGAAAT---GTCTACCACTATGTTCACAT------CCTCCAATGGCAACGTCAAC------------ACATCCACAGGATTCACTGCAAGCTCTGTAAAAGGCACAGACGTGACCTCAACTAGTTCCACCATATCTACCCAAACATCTACAACTAACGTAAATGTAATAACAACTTCACCAAACGGCGA---CACGAATTCATCGACACAGCATGTAACCGATAGTACTGTGACTTTGCAAACTATATCATTAT---CAACCAACAC------TAC------TACTAT---GGT------------AAATGCAAATGAAAACGTCACTACACCGCTTCCAACTTGCTCATCGC---CTAACAG---------------------------------------------------CACAAATAATACGATATCAAAAGAATCTGAAACATTATTGGAGGCGGCACAAGGAGACAATATTACTATAACACACAACCTAACCATCACATCGTGCTACAAAACAGCCTGGCTTAGACATTTTAATATATCCACACACGGAAAATACACCCATCCCAACATAAGAAATGGAAA---ATATCATAACCATTCATTGAAAATCCTCCATTCGCGTATACTATGTGAGTGGCACACAAATTATCTAAAACATCACTATGATTTATGTTTTACATGCGATCGTAATTTATCTTTATCTCTGTACGGTCTTAATTTTACTCATTCTGGTAAATATAGCTTTCGATGTTATAAAACTGGGCATCCCTCCGAACAAA------------ATCAAAACTTTAATCTGCAAGTACATCCTAGAAACAACACAAACGGGACACACGTGAATCCTTGGGTATGTGAAGAACCAAAACACGAATGGGACACTTC------------TCATAAACCGACCAATTATAAAGACAATACAGCCACATCATCTATAGATCATTTATACCGCTATAACAATCATTTTAACACATCACACGGCAGACGCACTACGTGGACGTTAGCATTAATTTGTGTAGCCTGCATTCTCCTATTTTTCGTCCGACGAACTCTAAATAAAAAATATCATCCATTAAGTGACGATATAAGTGAATCAGAATTCATAGTTCGATACAATCCTGAGCACGAGGACTAA

BE/10/2011 ATGCGTACACAACATCGACGGTGGAACAAGTCATCATATACGCAAATAATATGCATGTTTATTATTTTTTGGATTCTGCAGAAAAGCAAGTGTAACAACACCACTATCGTTAATACTTCCACGTCGATTACACCCACAAGCTTA---------ATATCTACTACACAACTGACATCTAAGTTACAAACCACCGAAAT---GTCTACCACTATGTTCACAT------CCTCCAATGGCAACGTCAAC------------ACATCCACAGGATTCACTGCAAGCTCTGTAAAAGGCACAGACGTGACCTCAACTAGTTCCACCATATCTACCCAAACATCTACAACTAACGTAAATGTAATAACAACTTCACCAAACGGCGA---CACGAATTCATCGACACAGCATGTAACCGATAGTACTGTGACTTTGCAAACTATATCATTAT---CAACCAACAC------TAC------TACTAT---GGT------------AAATGCAAATGAAAACGTCACTACACCGCTTCCAACTTGCTCATCGC---CTAACAG---------------------------------------------------CACAAATAATACGATATCAAAAGAATCTGAAACATTATTGGAGGCGGCACAAGGAGACAATATTACTATAACACACAACCTAACCATCACATCGTGCTACAAAACAGCCTGGCTTAGACATTTTAATATATCCACACACGGAAAATACACCCATCCCAACATAAGAAATGGAAA---ATATCATAACCATTCATTGAAAATCCTCCATTCGCGTATACTATGTGAGTGGCACACAAATTATCTAAAACATCACTATGATTTATGTTTTACATGCGATCGTAATTTATCTTTATCTCTGTACGGTCTTAATTTTACTCATTCTGGTAAATATAGCTTTCGATGTTATAAAACTGGGCATCCCTCCGAACAAA------------ATCAAAACTTTAATCTGCAAGTACATCCTAGAAACAACACAAACGGGACACACGTGAATCCTTGGGTATGTGAAGAACCAAAACACGAATGGGACACTTC------------TCATAAACCGACCAATTATAAAGACAATACAGCCACATCATCTATAGATCATTTATACCGCTATAACAATCATTCTAACACATCACACGGCAGACGCACTACGTGGACGTTAGCATTAATTTGTGTAGCCTGCATTCTCCTATTTTTCGTCCGACGAGCTCTAAATAAAAAATATCATCCATTAAGTGACGATATAAGTGAATCAGAATTCATAGTTCGATACAATCCTGAGCACGAGGACTAA

BE/20/2011 ATGCGTACACAACATCGACGGTGGAACAAGTCATCATATACGCAAATAATATGCATGTTTATTATTTTTTGGATTCTGCAGAAAAGCAAGTGTAACAACACCACTATCGTTAATACTTCCACGTCGATTACACCCACAAGCTTA---------ATATCTACTACACAACTGACATCTAAGTTACAAACCACCGAAAT---GTCTACCACTATGTTCACAT------CCTCCAATGGCAACGTCAAC------------ACATCCACAGGATTCACTGCAAGCTCTGTAAAAGGCACAGACGTGACCTCAACTAGTTCCACCATATCTACCCAAACATCTACAACTAACGTAAATGTAATAACAACTTCACCAAACGGCGA---CACGAATTCATCGACACAGCATGTAACCGATAGTACTGTGACTTTGCAAACTATATCATTAT---CAACCAACAC------TAC------TACTAT---GGT------------AAATGCAAATGAAAACGTCACTACACCGCTTCCAACTTGCTCATCGC---CTAACAG---------------------------------------------------CACAAATAATACGATATCAAAAGAATCTGAAACATTATTGGAGGCGGCACAAGGAGACAATATTACTATAACACACAACCTAACCATCACATCGTGCTACAAAACAGCCTGGCTTAGACATTTTAATATATCCACACACGGAAAATACACCCATCCCAACATAAGAAATGGAAA---ATATCATAACCATTCATTGAAAATCCTCCATTCGCGTATACTATGTGAGTGGCACACAAATTATCTAAAACATCACTATGATTTATGTTTTACATGCGATCGTAATTTATCTTTATCTCTGTACGGTCTTAATTTTACTCATTCTGGTAAATATAGCTTTCGATGTTATAAAACTGGGCATCCCTCCGAACAAA------------ATCAAAACTTTAATCTGCAAGTACATCCTAGAAACAACACAAACGGGACACACGTGAATCCTTGGGTATGTGAAGAACCAAAACACGAATGGGACACTTC------------TCATAAACCGACCAATTATAAAGACAATACAGCCACATCATCTATAGATCATTTATACCGCTATAACAATCATTCTAACACATCACACGGCAGACGCACTACGTGGACGTTAGCATTAATTTGTGTAGCCTGCATTCTCCTATTTTTCGTCCGACGAGCTCTAAATAAAAAATATCATCCATTAAGTGACGATATAAGTGAATCAGAATTCATAGTTCGATACAATCCTGAGCACGAGGACTAA

BE/9/2010 ATGCGTACACAACATCGACGGTGGAACAAGTCATCATATACGCAAATAATATGCATGTTTATTATTTTTTGGATTCTGCAGAAAAGCAAGTGTAACAACACCACTATCGTTAATACTTCCACGTCGATTACACCCACAAGCTTA---------ATATCTACTACACAACTGACATCTAAGTTACAAACCACCGAAAT---GTCTACCACTATGTTCACAT------CCTCCAATGGCAACGTCAAC------------ACATCCACAGGATTCACTGCAAGCTCTGTAAAAGGCACAGACGTGACCTCAACTAGTTCCACCATATCTACCCAAACATCTACAACTAACGTAAATGTAATAACAACTTCACCAAACGGCGA---CACGAATTCATCGACACAGCATGTAACCGATAGTACTGTGACTTTGCAAACTATATCATTAT---CAACCAACAC------TAC------TACTAT---GGT------------AAATGCAAATGAAAACGTCACTACACCGCTTCCAACTTGCTCATCGC---CTAACAG---------------------------------------------------CACAAATAATACGATATCAAAAGAATCTGAAACATTATTGGAGGCGGCACAAGGAGACAATATTACTATAACACACAACCTAACCATCACATCGTGCTACAAAACAGCCTGGCTTAGACATTTTAATATATCCACACACGGAAAATACACCCATCCCAACATAAGAAATGGAAA---ATATCATAACCATTCATTGAAAATCCTCCATTCGCGTATACTATGTGAGTGGCACACAAATTATCTAAAACATCACTATGATTTATGTTTTACATGCGATCGTAATTTATCTTTATCTCTGTACGGTCTTAATTTTACTCATTCTGGTAAATATAGCTTTCGATGTTATAAAACTGGGCATCCCTCCGAACAAA------------ATCAAAACTTTAATCTGCAAGTACATCCTAGAAACAACACAAACGGGACACACGTGAATCCTTGGGTATGTGAAGAACCAAAACACGAATGGGACACTTC------------TCATAAACCGACCAATTATAAAGACAATACAGCCACATCATCTATAGATCATTTATACCGCTATAACAATCATTCTAACACATCACACGGCAGACGCACTACGTGGACGTTAGCATTAATTTGTGTAGCCTGCATTCTCCTATTTTTCGTCCGACGAGCTCTAAATAAAAAATATCATCCATTAAGTGACGATATAAGTGAATCAGAATTCATAGTTCGATACAATCCTGAGCACGAGGACTAA

BE/10/2012 ATGCGTACACAACATCGACGGTGGAACAAGTCATCATATACGCAAATAATATGCATGTTTATTATTTTTTGGATTCTGCAGAAAAGCAAGTGTAACAACACCACTATCGTTAATACTTCCACGTCGATTACACCCACAAGCTTA---------ATATCTACTACACAACTGACATCTAAGTTACAAACCACCGAAAT---GTCTACCACTATGTTCACAT------CCTCCAATGGCAACGTCAAC------------ACATCCACAGGATTCACTGCAAGCTCTGTAAAAGGCACAGACGTGACCTCAACTAGTTCCACCATATCTACCCAAACATCTACAACTAACGTAAATGTAATAACAACTTCACCAAACGGCGA---CACGAATTCATCGACACAGCATGTAACCGATAGTACTGTGACTTTGCAAACTATATCATTAT---CAACCAACAC------TAC------TACTAT---GGT------------AAATGCAAATGAAAACGTCACTACACCGCTTCCAACTTGCTCATCGC---CTAACAG---------------------------------------------------CACAAATAATACGATATCAAAAGAATCTGAAACATTATTGGAGGCGGCACAAGGAGACAATATTACTATAACACACAACCTAACCATCACATCGTGCTACAAAACAGCCTGGCTTAGACATTTTAATATATCCACACACGGAAAATACACCCATCCCAACATAAGAAATGGAAA---ATATCATAACCATTCATTGAAAATCCTCCATTCGCGTATACTATGTGAGTGGCACACAAATTATCTAAAACATCACTATGATTTATGTTTTACATGCGATCGTAATTTATCTTTATCTCTGTACGGTCTTAATTTTACTCATTCTGGTAAATATAGCTTTCGATGTTATAAAACTGGGCATCCCTCCGAACAAA------------ATCAAAACTTTAATCTGCAAGTACATCCTAGAAACAACACAAACGGGACACACGTGAATCCTTGGGTATGTGAAGAACCAAAACACGAATGGGACACTTC------------TCATAAACCGACCAATTATAAAGACAATACAGCCACATCATCTATAGATCATTTATACCGCTATAACAATCATTCTAACACATCACACGGCAGACGCACTACGTGGACGTTAGCATTAATTTGTGTAGCCTGCATTCTCCTATTTTTCGTCCGACGAACTCTAAATAAAAAATATCATCCATTAAGTGACGATATAAGTGAATCAGAATTCATAGTTCGATACAATCCTGAGCACGAGGACTAA

BE/1/2010 ATGCGTACACAACATCGACGGTGGAACAAGTCATCATATACGCAAATAATATGCATGTTTATTATTTTTTGGATTCTGCAGAAAAGCAAGTGTAACAACACCACTATCGTTAATACTTCCACGTCGATTACACCCACAAGCTTA---------ATATCTACTACACAACTGACATCTAAGTTACAAACCACCGAAAT---GTCTACCACTATGTTCACAT------CCTCCAATGGCAACGTCAAC------------ACATCCACAGGATTCACTGCAAGCTCTGTAAAAGGCACAGACGTGACCTCAACTAGTTCCACCATATCTACCCAAACATCTACAACTAACGTAACTGTAATAACAACTTCACCAAACGGCGA---CACGAATTCATCGACACAGCATGTAACCGATAGTACTGTGACTTTGCAAACTATATCATTAT---CAACCAACAC------TAC------TACTAT---GGT------------AAATGCAAATGAAAACGTCACTACACCGCTTCCAACTTGCTCATCGC---CTAACAG---------------------------------------------------CACAAATAATACGATATCAAAAGAATCTGAAACATTATTGGAGGCGGCACAAGGAGACAATATTACTATAACACACAACCTAACCATCACATCGTGCTACAAAACAGCCTGGCTTAGACATTTTAATATATCCACACACGGAAAATACACCCATCCCAACATAAGAAATGGAAA---ATATCATAACCATTCATTGAAAATCCTCCATTCGCGTATACTATGTGAGTGGCACACAAATTATCTAAAACATCACTATGATTTATGTTTTACATGCGATCGTAATTTATCTTTATCTCTGTACGGTCTTAATTTTACTCATTCTGGTAAATATAGCTTTCGATGTTATAAAACTGGGCATCCCTCCGAACAAA------------ATCAAAACTTTAATCTGCAAGTACATCCTAGAAACAACACAAACGGGACACACGTGAATCCCTGGGTATGTGAGGAACCAAAACACGAATGGGACACTTC------------TCATAAACCGACCAATTATAAAGACAATACAGCCACATCATCTATAGATCATTTATACCGCTATAACAATCATTCTAACACATCACACGGCAGACGCACTACGTGGACGTTAGCATTAATTTGTGTAGCCTGCATTCTCCTATTTTTCGTCCGACGAGCTCTAAATAAAAAATATCATCCATTAAGGGACGATATAAGTGAATCAGAATTCATAGTTCGATACAATCCTGAGCACGAGGACTAA

CINCY ATGCGTACACAACATCGACGGTGGAACAAGTCATCATATACGCAAATAATATGCATGTTTATTATTTTTTGGATTCTGCAGAAAAGCAAGTGTAACAACACCACTATCGTTAATACTTCCACGTCGATTACACCCACAAGCTTA---------ATATCTACTACACAACTGACATCTAAGTTACAAACCACCGAAAT---GTCTACCACTATGTTCACAT------CCTCCAATGGCAACGTCAAC------------ACATCCACAGGATTCACTGCAAGCTCTGTAAAAGGCACAGACGTGACCTCAACTAGTTCCACCATATCTACCCAAACATCTACAACTAACGTAACTGTAATAACAACTTCACCAAACGGCGA---CACGAATTCATCGACACAGCATGTAACCGATAGTACTGTGACTTTGCAAACTATATCATTAT---CAACCAACAC------TAC------TACTAT---GGT------------AAATGCAAATGAAAACGTCACTACACCGCTTCCAACTTGCTCATCGC---CTAACAG---------------------------------------------------CACAAATAATACGATATCAAAAGAATCTGAAACATTATTGGAGGCGGCACAAGGAGACAATATTACTATAACACACAACCTAACCATCACATCGTGCTACAAAACAGCCTGGCTTAGACATTTTAATATATCCACACACGGAAAATACACCCATCCCAACATAAGAAATGGAAA---ATATCATAACCATTCATTGAAAATCCTCCATTCGCGTATACTATGTGAGTGGCACACAAATTATCTAAAACATCACTATGATTTATGTTTTACATGCGATCGTAATTTATCTTTATCTCTGTACGGTCTTAATTTTACTCATTCTGGTAAATATAGCTTTCGATGTTATAAAACTGGGCATCCCTCCGAACAAA------------ATCAAAACTTTAATCTGCAAGTACATCCTAGAAACAACACAAACGGGACACACGTGAATCCCTGGGTATGTGAAGAACCAAAACACGAATGGGACACTTC------------TCATAAACCGACCAATTATAAAGACAATACAGCCACATCATCTATAGATCATTTATACCGCTATAACAATCATTCTAATACATCACACGGCAGACGCACTACGTGGACGTTAGCATTAATTTGTGTAGCCTGCATTCTCCTATTTTTCGTCCGACGAGCTCTAAATAAAAAATATCATCCATTAAGTGACGATATAAGTGAATCAGAATTCATAGTTCGATACAATCCTGAGCACGAGGACTAA

Pat_H ATGCGTACACAACATCGACGGTGGAACAAGTCATCATATACGCAAATAATATGCATGTTTATTATTTTTTGGATTCTGCAGAAAAGCAAGTGTAACAACACCACTATCGTTAATACTTCCACGTCGATTACACCCACAAGCTTA---------ATATCTACTACACAACTGACATCTAAGTTACAAACCACCGAAAT---GTCTACCACTATGTTCACAT------CCTCCAATGGCAACGTCAAC------------ACATCCACAGGATTCACTGCAAGCTCTGTAAAAGGCACAGACGTGACCTCAACTAGTTCCACCATATCTACCCAAACATCTACAACTAACGTAACTGTAATAACAACTTCACCAAACGGCGA---CACGAATTCATCGACACAGCATGTAACCGATAGTACTGTGACTTTGCAAACTATATCATTAT---CAACCAACAC------TAC------TACTAT---GGT------------AAATGCAAATGAAAACGTCACTACACCGCTTCCAACTTGCTCATCGC---CTAACAG---------------------------------------------------CACAAATAATACGATATCAAAAGAATCTGAAACATTATTGGAGGCGGCACAAGGAGACAATATTACTATAACACACAACCTAACCATCACATCGTGCTACAAAACAGCCTGGCTTAGACATTTTAATATATCCACACACGGAAAATACACCCATCCCAACATAAGAAATGGAAA---ATATCATAACCATTCATTGAAAATCCTCCATTCGCGTATACTATGTGAGTGGCACACAAATTATCTAAAACATCACTATGATTTATGTTTTACATGCGATCGTAATTTATCTTTATCTCTGTACGGTCTTAATTTTACTCATTCTGGTAAATATAGCTTTCGATGTTATAAAACTGGGCATCCCTCCGAACAAA------------ATCAAAACTTTAATCTGCAAGTACATCCTAGAAACAACACAAACGGGACACACGTGAATCCCTGGGTATGTGAAGAACCAAAACACGAATGGGACACTTC------------TCATAAACCGACCAATTATAAAGACAATACAGCCACATCATCTATAGATCATTTATACCGCTATAACAATCATTCTAACACATCACACGGCAGACGCACTACGTGGACGTTAGCATTAATTTGTGTAGCCTGCATTCTCCTATTTTTCGTCCGACGAGCTCTAAATAAAAAATATCATCCATTAAGTGACGATATAAGTGAATCAGAATTCATAGTTCGATACAATCCTGAGCACGAGGACTAA

BE/13/2011 G10 ATGCGTGGCATTTGGAAGCCAGGAAGTAACCTAATATACAGA---TTGATATGCGTACTTGCTATCTGGACTCCTTTGTACAGAAGTAAATGTGAGAACACTACTAATGC---------------TTCATCCAACGCTGAATCTACAAGCGTTATCACTA---CACTAACTACAGATATTTCATCTACATACCAATCATTACCTGCATCAACTTCAACAT------------CTATCTCTAATACAG------CTATCACAAGCACTCGCATGGCTTCCAGTCCAAATACA---TCGTATGTTTCCACCTCTTACGATAACACATCGA------------------------CGTCAGTGATGATTGCAAGCACATCCACGGAAACTAACGTTAGTTCTACGGAAATAACCGTTACTTCTAT---------AACTACTTCAGCCGCAACCAACTCCACAGTGAATGTGACAGACATGACCGCAACAATTATTA---CATCTCCAGCCGCAACAACAAACGCAACCATTAGTAACACATCATTCG---TAAATTGCGAAGTGTGGAA---CAC---TACGCTTAAAAATAATACAAAAGACCGTG---ACGAGTGCCAACCAATAAAAGTAAATAAAACAGATATAATAGCAGAAGAGTGGACCAATGTTACTATACAAAGCAATTTCACAGTACCGCACTGTAACAAGGTAATTTGGATACGACACTATAATCGAACCACACACGGAAACTACTTCCCAATACAATATAGACGTCC----------TTTTATTAGAAGTGCTTTGTACTCACTT--GAAATTTGCGGCCAAACGTACACCCACAGTCTTCTACACTTATATGATTTGTGCATCTCGT---GTGACAACGGAACACTACATCTTTACGGTGTAAACACAACTCATTCAGGTAGATATACTGCACGTTGCCATACTTATGAATATCATAGCAACCACGGTAAACATGAGGACAAAAACTTTAATTTGATTATAAATCCTAGAAATAATACTAATTATACCAACGACATCTGGATATGTC-----------CACAAC----------CTACGAAAGACGAAACCCAAGCAGACAACCAAAGTAAAGAAAAAC---ATTTAACAACGACGGACAATTCACCTTCTCATAAACCAAACCATTACTCAAGAACCTCACACCGCAGTGCCTGGACTGTCACGTTGCTTTGTATTGCCTGTATACTCCTATTTTTTTTCCGACGTTTTTTTAACAAAAAATACCGTACGCTGGATGATACTGTCAGCGAATCCGAATTTATTGTACGGTATAACCCAGAGCATGAAGATTGA

BE/27/2010 ATGCGTGGCATTTGGAAGCCAGGAAGTAACCTAATATACAGA---TTGATATGCGTACTTGCTATCTGGACTCCTTTGTACAGAAGTAAATGTGAGAACACTACTAATGC---------------TTCATCCAACGCTGAATCTACAAGCGTTATCACTA---CACTAACTACAGATATTTCATCTACATACCAATCATTACCTGCATCAACTTCAACAT------------CTATCTCTAATACAG------CTATCACAAGCACTCGCATGGCTTCCAGTCCAAATACA---TCGTATGTTTCCACCTCTTACGATAACACATCGA------------------------CGTCAGTGATGATTGCAAGCACATCCACGGAAACTAACGTTAGTTCTACGGAAATAACCGTTACTTCTAT---------AACTACTTCAGCCGCAACCAACTCCACAGTGAATGTGACAGACATGACCGCAACAATTATTA---CATCTCCAGCCGCAACAACAAACGCAACCATTAGTAACACATCATTCG---TAAATTGCGAAGTGTGGAA---CAC---TACGCTTAAAAATAATACAAAAGACCGTG---ACGAGTGCCAACCAATAAAAGTAAATAAAACAGATATAATAGCAGAAGAGTGGACCAATGTTACTATACAAAGCAATTTCACAGTACCGCACTGTAACAAGGTAATTTGGATACGACACTATAATCGAACCACACACGGAAACTACTTCCCAATACAATATAGACGTCC----------TTTTATTAGAAGTGCTTTGTACTCACTT--GAAATTTGCGGCCAAACGTACACCCACAGTCTTCTACACTTATATGATTTGTGCATCTCGT---GTGACAACGGAACACTACATCTTTACGGTGTAAACACAACTCATTCAGGTAGATATACTGCACGTTGCCATACTTATGAATATCATAGCAACCACGGTAAACATGAGGACAAAAACTTTAATTTGATTATAAATCCTAGAAATAATACTAATTATACCAACGACATCTGGATATGTC-----------CACAAC----------CTACGAAAGACGAAACCCAAGCAGACAACCAAAGTAAAGAAAAAC---ATTTAACAACGACGGACAATTCACCTTCTCATAAACCAAACCATTACTCAAGAACCTCACACCGCAGTGCCTGGACTGTCACGTTGCTTTGTATTGCCTGTATACTCCTATTTTTTTTCCGACGTTTTTTTAACAAAAAATACCGTACGCTGGATGATACTGTCAGCGAATCCGAATTTATTGTACGGTATAACCCAGAGCATGAAGATTGA

NL/Rot2 ATGCGTGGCATTTGGAAGCCAGGAAGTAACCTAATATACAGA---TTGATATGCGTACTTACTATCTGGACTCCTTTGTACAGAAGTAAATGTGAGAACACTACTAATGC---------------TTCATCCAACGCTGAATCTACAAGCGTTATCACTA---CACTAACTACAGATATTTCATCTACATACCAATCATTACCTGCATCAACTTCAACAT------------CTATTTCTAATACAG------CTATTACAAGCACTCGCATGGCTTCCAGTCCAAATACA---TCGTATGTTTCCACCTCTTACGATAACACATCGA------------------------CGTCAGTGATGATCGCAAGCACATCCACGGAAACTAACGTTAGTTCTACGGAAATAACCGTTACTTCTAT---------AACTACTTCAGCCGCAACCAACTCCACAGTGAATGTGACAGACATGACCGCAACAATTATTA---CATCTCCAGCCGCAACAACAAACGCAACAATTAGTAACACATCATTCG---TAAATTGCGAAGTGTGGAA---CAC---CACGCTTAAAAATAATACAAAAGACCGTG---ACGAGTGCCAACCAATAAAAGTAAATAAAACAGATATAATAGCAGAAGAGTGGACCAATGTTACTATACAAAGCAATTTCACGATACCGCACTGTAACAAGGTAATTTGGATACGACACTATAATCGAACCACACACGGAAACTACTTCCCAATACAATATAGACGTCC----------TTTTATTAGAAGTGCTTTGTACTCACTT--GAAATTTGCGGCCAAACGTACACCCACAGTCTTCTACACTTATATGATTTGTGCATCTCGT---GTGACAACGGAACACTACATCTTTACGGTGTAAACACAACTCATTCAGGTAGATATACTGCACGTTGCCATACTTATGAATATCATAGCAACCACGGTAAACATGAGGACAAAAACTTTAATTTGATTATAAATCCTAGAAATAATACTAATTATACCAACAACATCTGGATATGTC-----------CACAAC----------CTACGAAAGACGAAACCCAAGCAGACAACCAAAGTAAAGAAAAAC---ATTTAACAACGACGGACAATTCACTTTCTCATAAACCAAACCATTACTCAAGAACCTCACACCGCAGTGCCTGGACTGTCACGTTGCTTTGTATTGCCTGTATACTCCTATTTTTTTTCCGACGTTTTTTTAACAAAAAATACCGTACGCTGGATGATACTGTCAGCGAATCCGAATTTATTGTACGGTATAACCCAGAGCATGAAGATTGA

Pat_K ATGCGTGGCATTTGGAAGCCAGGAAGTAACCTAATATACAGA---TTGATATGCGTACTTACTATCTGGACTCCTTTGTACAGAAGTAAATGTGAGAACACTACTAATGC---------------TTCATCCAACGCTGAATCTACAAGCGTTATCACTA---CACTAACTGCAGATACCTCATCTACATACCAATCATTACCTGCATCAACTTCAACAT------------CTATCTCTAATACAG------CTATCACAAGCACTCGCATGGCTTCCAGTCCAAATACA---TCGTATGTTTCCACCTCTTACGATAACACATCGA------------------------CGTCAGTGATGATTGCAAGCACATCCACGGAAACTAACGTTAGTTCTACGGAAATAACCGTTACATCTAT---------AACTACTTCAGCCACAACCAACTCCACAGTGACTGTGACAGACATGACCGCAACAATTATTA---CATCTTCAGCCGCAACAACAAACGCAACCATTAGTAACACATCATCCG---TAAATTGCGAAATGTGGAA---CAC---TACGCTTAAAAATAATACAAAAGACCGTG---ACGAGTGCCAACCAATAAAAGTAAATAAAACAGATATAATAGCAGAAGAGTGGACCAATGTTACTATACAAAGCAATTTCACGATACCGCACTGTAACAAGGTAATTTGGATACGACACTATAATCGAACCACACACGGAAACTACTTCCCAATACAATATAGACGTCC----------TTTTATTAGAAGTGCTTTGTACTCACTT--GAAATTTGCGGCCAAACGTACACCCACAGTCTTCTACACTTATATGATTTGTGCATCTCGT---GTGACAACGGAACACTACATCTTTACGGTGTAAACACAACTCATTCAGGTAGATATACTGCACGTTGTCATACTTATGAATATCATAGCAACCACGGTAAACATGAAGACAAAAACTTTAATTTGATTATAAATCCTAGAAATAATACTAATTATACCAACGACATCTGGATATGTC-----------CACAAC----------CTACGAAAGACGAAACCCAAGCAGACAACCAAAGTAAAGAAAAAC---ATTTAACAACGACGGACAATTCACTTTCTCATAAACCAAACCATTACTCAAGAACCTCACACCGCAGTGCCTGGACTGTCACGTTGCTTTGTGTTGCCTGTATACTCCTATTTTTTTTCCGACGTTTTTTTAACAAAAAATACCGTACGCTAGATGATACTGTCAGCGAATCCGAATTTATTGTACGGTATAACCCAGAGCATGAAGATTGA

PRA5 ATGCGTGGCATTTGGAAGCCAAGAAGTAACCTAATATACAGA---TTGATATACGTACTTACTATCTGGACTCCTTTGTACAGAAGTAAATGTGAGAACACTACTAATGC---------------TTCATCCAACGCTGAATCTACAAGCGTTATCACTA---CACTAACTGCAGATACCTCATCTACATACCAATCATTACCTGCATCAACTTCAACAT------------CTATCTCTAATACAG------CTATCACAAGCACTCGCATGGCTTCCAGTCCAAATACA---TCGTATGTTTCCACCTCTTACGATAACACATCGA------------------------CGTCAGTGATGATTACAAGCACATCCACGGAAACTAACGTTAGTTCTACGGAAATAACCGTTACATCTAT---------AACTACTTCAGCCACAACCAACTCCACAGTGAATGTGACAGACATGACCGCAACAATTATTA---CATCTCCAGCCGCAACAACAAACGCAACCATTAGTAACACATCATTCG---TAAATTGCGAAATGTGGAA---CAC---TACGCTTAAAAATAATACAAAAGACCGTG---ACGAGTGCCAACCAATAAAAGTAAATAAAACAGATATAATAGCAGAAGAGTGGACCAATGTTACTATACAAAGCAATTTCACGATACCGCACTGTAACAAGGTAATTTGGATACGACACTATAATCGAACCACACACGGAAACTACTTCCCAATACAATATAGACGTCC----------TTTTATTAGAAGTGCTTTGTACTCACTT--GAAATTTGCGGCCAAACGTACACCCACAGTCTTCTACACTTATATGATTTGTGCATCTCGT---GTGACAACGGAACACTACATCTTTACGGTGTAAACACAACTCATTCAGGTAGATATACTGCACGTTGCCATACTTATGAATATCATAGCAACCACGGTAAACATGAGGACAAAAACTTTAATTTGATTATAAATCCTAGAAATAATACTAATTATACCAACGACATCTGGATATGTC-----------CACAAC----------CTACGAAAGACGAAACCCAAGCAGACAACCAAAGTAAAGAAAAAC---ACTTAACAACGACGGACAATTCACTTTCTCATAAACCAAACCATTACTCAAGAACCTCACACCGCAGTGCCTGGACTGTCGCGTTGCTTTGTATTGCCTGTATACTCCTATTTTTTTTCCGACGTTTT-TTAACAAAAAATACCGTACGCTGGATGATACTGTCAGCGAATCCGAATTTATTGTACGGTATAACCCAGAGCATGAAGATTGA

BE/11/2010 ATGCGTGGCATTTGGAAGCCAAGAAGTAACCTAATATACAGA---TTGATATGCGTACTTACTATCTGGACTCCTTTGTACAGAAGTAAATGTGAGAACACTACTAATGC---------------TTCATCCAACGCTGAATCTACAAGCGTTATCACTA---CACTAACTGCAGATACCTCATCTACATACCAATCATTACCTGCATCAACTTCAACAT------------CTATCTCTAATACAG------CTATCACAAGCACTCGCATGGCTTCCAGTCCAAATACA---TCGTATGTTTCCACCTCTTACGATAACACATCGA------------------------CGTCAGTGATGATTACAAGCACATCCACGGAAACTAACGTTAGTTCTACGGAAATAACCGTTACATCTAT---------AACTACTTCAGCCACAACCAACTCCACAGTGAATGTGACAGACATGACCGCAACAATTATTA---CATCTCCAGCCGCAACAACAAACGCAACCATTAGTAACACATCATTCG---TAAATTGCGAAATGTGGAA---CAC---TACGCTTAAAAATAATACAAAAGACCGTG---ACGAGTGCCAACCAATAAAAGTAAATAAAACAGATATAATAGCAGAAGAGTGGACCAATGTTACTATACAAAGCAATTTCACGATACCGCACTGTAACAAGGTAATTTGGATACGACACTATAATCGAACCACACACGGAAACTACTTCCCAATACAATATAGACGTCC----------TTTTATTAGAAGTGCTTTGTACTCACTT--GAAATTTGCGGCCAAACGTACACCCACAGTCTTCTACACTTATATGATTTGTGCATCTCGT---GTGACAACGGAACACTACATCTTTACGGTGTAAACACAACTCATTCAGGTAGATATACTGCACGTTGCCATACTTATGAATATCATAGCAACCACGGTAAACATGAGGACAAAAACTTTAATTTGATTATAAATCCTAGAAATAATACTAATTATACCAACGACATCTGGATATGTC-----------CACAAC----------CTACGAAAGACGAAACCCAAGCAGACAACCAAAGTAAAGAAAAAC---ATTTAACAACGACGGACAATTCACTTTCTCATAAACCAAACCATTACTCAAGAACCTCACACCGCAGTGCCTGGACTGTCGCGTTGCTTTGTATTGCCTGTATACTCCTATTTTTTTTCCGACGTTTTTTTAACAAAAAATACCGTACGCTGGATGATACTGTCAGCGAATCCGAATTTATTGTACGGTATAACCCAGAGCATGAAGATTGA

JER893 ATGCGTGGCATTTGGAAGCCAAGAAGTAACCTAATATACAGA---TTGATATGCGTACTTACTATCTGGACTCCTTTGTACAGAAGTAAATGTGAGAACACTACTAATGC---------------TTCATCCAACGCTGAATCTACAAGCGTTATCACTA---CACTAACTGCAGATACCTCATCTACATACCAATCATTACCTGCATCAACTTCAACAT------------CTATCTCTAATACAG------CTATCACAAGCACTCGCATGGCTTCCAGTCCAAATACA---TCGTATGTTTCCACCTCTTACGATAACACATCGA------------------------CGTCAGTGATGATTACAAGCACATCCACGGAAACTAACGTTAGTTCTACGGAAATAACCGTTACATCTAT---------AACTACTTCAGCCACAACCAACTCCACAGTGAATGTGACAGACATGACCGCAACAATTATTA---CATCTCCAGCCGCAACAACAAACGCAACCATTAGTAACACATCATTCG---TAAATTGCGAAATGTGGAA---CAC---TACGCTTAAAAATAATACAAAAGACCGTG---ACGAGTGCCAACCAATAAAAGTAAATAAAACAGATATAATAGCAGAAGAGTGGACCAATGTTACTATACAAAGCAATTTCACGATACCGCACTGTAACAAGGTAATTTGGATACGACACTATAATCGAACCACACACGGAAACTACTTCCCAATACAATATAGACGTCC----------TTTTATTAGAAGTGCTTTGTACTCACTT--GAAATTTGCGGCCAAACGTACACCCACAGTCTTCTACACTTATATGATTTGTGCATCTCGT---GTGACAACGGAACACTACATCTTTACGGTGTAAACACAACTCATTCAGGTAGATATACTGCACGTTGCCATACTTATGAATATCATAGCAACCACGGTAAACATGAGGACAAAAACTTTAATTTGATTATAAATCCTAGAAATAATACTAATTATACCAACGACATCTGGATATGTC-----------CACAAC----------CTACGAAAGACGAAACCCAAGCAGACAACCAAAGTAAAGAAAAAC---ATTTAACAACGACGGACAATTCACTTTCTCATAAACCAAACCATTACTCAAGAACCTCACACCGCAGTGCCTGGACTGTCGCGTTGCTTTGTATTGCCTGTATACTCCTATTTTTTTTCCGACGTTTTTTTAACAAAAAATACCGTACGCTGGATGATACTGTCAGCGAATCCGAATTTATTGTACGGTATAACCCAGAGCATGAAGATTGA

2CEN2 ATGCGTGGCATTTGGAAGCCAAGAAGTAACCTAATATACAGA---TTGATATGCGTACTTACTATCTGGACTCCTTTGTACAGAAGTAAATGTGAGAACACTACTAATGC---------------TTCATCCAACGCTGAATCTACAAGCGTTATCACTA---CACTAACTGCAGATACCTCATCTACATACCAATCATTACCTGCATCAACTTCAACAT------------CTATCTCTAATACAG------CTATCACAAGCACTCGCATGGCTTCCAGTCCAAATACA---TCGTATGTTTCCACCTCTTACGATAACACATCGA------------------------CGTCAGTGATGATTACAAGCACATCCACGGAAACTAACGTTAGTTCTACGGAAATAACCGTTACATCTAT---------AACTACTTCAGCCACAACCAACTCCACAGTGAATGTGACAGACATGACCGCAACAATTATTA---CATCTCCAGCCGCAACAACAAACGCAACCATTAGTAACACATCATTCG---TAAATTGCGAAATGTGGAA---CAC---TACGCTTAAAAATAATACAAAAGACCGTG---ACGAGTGCCAACCAATAAAAGTAAATAAAACAGATATAATAGCAGAAGAGTGGACCAATGTTACTATACAAAGCAATTTCACGATACCGCACTGTAACAAGGTAATTTGGATACGACACTATAATCGAACCACACACGGAAACTACTTCCCAATACAATATAGACGTCC----------TTTTATTAGAAGTGCTTTGTACTCACTT--GAAATTTGCGGCCAAACGTACACCCACAGTCTTCTACACTTATATGATTTGTGCATCTCGT---GTGACAACGGAACACTACATCTTTACGGTGTAAACACAACTCATTCAGGTAGATATACTGCACGTTGCCATACTTATGAATATCATAGCAACCACGGTAAACATGAGGACAAAAACTTTAATTTGATTATAAATCCTAGAAATAATACTAATTATACCAACGACATCTGGATATGTC-----------CACAAC----------CTACGAAAGACGAAACCCAAGCAGACAACCAAAGTAAAGAAAAAC---ACTTAACAACGACGGACAATTCACTTTCTCATAAACCAAACCATTACTCAAGAACCTCACACCGCAGTGCCTGGACTGTCGCGTTGCTTTGTATTGCCTGTATACTCCTATTTTTTTTCCGACGTTTTTTTAACAAAAAATACCGTACGCTGGATGATACTGTCAGCGAATCCGAATTTATTGTACGGTATAACCCAGAGCATGAAGATTGA

BE/16/2010 ATGCGTGGCATTTGGAAGCCAAGAAGTAACCTAATATACAGA---TTGATATGCGTACTTACTATCTGGACTCCTTTGTACAGAAGTAAATGTGAGAACACTACTAATGC---------------TTCATCCAACGCTGAATCTACAAGCGTTATCACTA---CACTAACTGCAGATACCTCATCTACATACCAATCATTACCTGCATCAACTTCAACAT------------CTATCTCTAATACAG------CTATCACAAGCACTCGCATGGCTTCCAGTCCAAATACA---TCGTATGTTTCCACCTCTTACGATAACACATCGA------------------------CGTCAGTGATGATTACAAGCACATCCACGGAAACTAACGTTAGTTCTACGGAAATAACCGTTACATCTAT---------AACTACTTCAGCCACAACCAACTCCACAGTGAATGTGACAGACATGACCGCAACAATTATTA---CATCTCCAGCCGCAACAACAAACGCAACCATTAGTAACACATCATTCG---TAAATTGCGAAATGTGGAA---CAC---TACGCTTAAAAATAATACAAAAGACCGTG---ACGAGTGCCAACCAATAAAAGTAAATAAAACAGATATAATAGCAGAAGAGTGGACCAATGTTACTATACAAAGCAATTTCACGATACCGCACTGTAACAAGGTAATTTGGATACGACACTATAATCGAACCACACACGGAAACTACTTCCCAATACAATATAGACGTCC----------TTTTATTAGAAGTGCTTTGTACTCACTT--GAAATTTGCGGCCAAACGTACACCCACAGTCTTCTACACTTATATGATTTGTGCATCTCGT---GTGACAACGGAACACTACATCTTTACGGTGTAAACACAACTCATTCAGGTAGATATACTGCACGTTGCCATACTTATGAATATCATAGCAACCACGGTAAACATGAGGACAAAAACTTTAATTTGATTATAAATCCTAGAAATAATACTAATTATACCAACGACATCTGGATATGTC-----------CACAAC----------CTACGAAAGACGAAACCCAAGCAGACAACCAAAGTAAAGAAAAAC---ACTTAACAACGACGGACAATTCACTTTCTCATAAACCAAACCATTACTCAAGAACCTCACACCGCAGTGCCTGGACTGTCGCGTTGCTTTGTATTGCCTGTATACTCCTATTTTTTTTCCGACGTTTTTTTAACAAAAAATACCGTACGCTGGATGATACTGTCAGCGAATCCGAATTTATTGTACGGTATAACCCAGAGCATGAAGATTGA

HAN31 ATGCGTGGCATTTGGAAGCCAAGAAGTAACCTAATATACAGA---TTGATATGCGTACTTACTATCTGGACTCCTTTGTACAGAAGTAAATGTGAGAACACTACTAATGC---------------TTCATCCAACGCTGAATCTACAAGCGTTATCACTA---CACTAACTGCAGATACCTCATCTACATACCAATCATTACCTGCATCAACTTCAACAT------------CTATCTCTAATACAG------CTATCACAAGCACTCGCATGGCTTCCAGTCCAAATACA---TCGTATGTTTCCACCTCTTACGATAACACATCGA------------------------CGTCAGTGATGATTACAAGCACATCCACGGAAACTAACGTTAGTTCTACGGAAATAACCGTTACATCTAT---------AACTACTTCAGCCACAACCAACTCCACAGTGAATGTGACAGACATGACCGCAACAATTATTA---CATCTCCAGCCGCAACAACAAACGCAACCATTAGTAACACATCATTCG---TAAATTGCGAAATGTGGAA---CAC---TACGCTTAAAAATAATACAAAAGACCGTG---ACGAGTGCCAACCAATAAAAGTAAATAAAACAGATATAATAGCAGAAGAGTGGACCAATGTTACTATACAAAGCAATTTCACGATACCGCACTGTAACAAGGTAATTTGGATACGACACTATAATCGAACCACACACGGAAACTACTTCCCAATACAATATAGACGTCC----------TTTTATTAGAAGTGCTTTGTACTCACTT--GAAATTTGCGGCCAAACGTACACCCACAGTCTTCTACACTTATATGATTTGTGCATCTCGT---GTGACAACGGAACACTACATCTTTACGGTGTAAACACAACTCATTCAGGTAGATATACTGCACGTTGCCATACTTATGAATATCATAGCAACCACGGTAAACATGAGGACAAAAACTTTAATTTGATTATAAATCCTAGAAATAATACTAATTATACCAACGACATCTGGATATGTC-----------CACAAC----------CTACGAAAGACGAAACCCAAGCAGACAACCAAAGTAAAGAAAAAC---ACTTAACAACGACGGACAATTCACTTTCTCATAAACCAAACCATTACTCAAGAACCTCACACCGCAGTGCCTGGACTGTCGCGTTGCTTTGTATTGCCTGTATACTCCTATTTTTTTTCCGACGTTTTTTTAACAAAAAATACCGTACGCTGGATGATACTGTCAGCGAATCCGAATTTATTGTACGGTATAACCCAGAGCATGAAGATTGA

PRA3 ATGCGTGGCATTTGGAAGCCAAGAAGTAACCTAATATACAGA---TTGATATGCGTACTTACTATCTGGACTCCTTTGTACAGAAGTAAATGTGAGAACACTACTAATGC---------------TTCATCCAACGCTGAATCTACAAGCGTTATCACTA---CACTAACTGCAGATACCTCATCTACATACCAATCATTACCTGCATCAACTTCAACAT------------CTATCTCTAATACAG------CTATCACAAGCACTCGCATGGCTTCCAGTCCAAATACA---TCGTATGTTTCCACCTCTTACGATAACACATCGA------------------------CGTCAGTGATGATTACAAGCACATCCACGGAAACTAACGTTAGTTCTACGGAAATAACCGTTACATCTAT---------AACTACTTCAGCCACAACCAACTCCACAGTGAATGTGACAGACATGACCGCAACAATTATTA---CATCTCCAGCCGCAACAACAAACGCAACCATTAGTAACACATCATTCG---TAAATTGCGAAATGTGGAA---CAC---TACGCTTAAAAATAATACAAAAGACCGTG---ACGAGTGCCAACCAATAAAAGTAAATAAAACAGATATAATAGCAGAAGAGTGGACCAATGTTACTATACAAAGCAATTTCACGATACCGCACTGTAACAAGGTAATTTGGATACGACACTATAATCGAACCACACACGGAAACTACTTCCCAATACAATATAGACGTCC----------TTTTATTAGAAGTGCTTTGTACTCACTT--GAAATTTGCGGCCAAACGTACACCCACAGTCTTCTACACTTATATGATTTGTGCATCTCGT---GTGACAACGGAACACTACATCTTTACGGTGTAAACACAACTCATTCAGGTAGATATACTGCACGTTGCCATACTTATGAATATCATAGCAACCACGGTAAACATGAGGACAAAAACTTTAATTTGATTATAAATCCTAGAAATAATACTAATTATACCAACGACATCTGGATATGTC-----------CACAAC----------CTACGAAAGACGAAACCCAAGCAGACAACCAAAGTAAAGAAAAAC---ACTTAACAACGACGGACAATTCACTTTCTCATAAACCAAACCATTACTCAAGAACCTCACACCGCAGTGCCTGGACTGTCGCGTTGCTTTGTATTGCCTGTATACTCCTATTTTTTTTCCGACGTTTTTTTAACAAAAAATACCGTACGCTGGATGATACTGTCAGCGAATCCGAATTTATTGTACGGTATAACCCAGAGCATGAAGATTGA

HANRTR10 ATGCGTGGCATTTGGAAGCCAAGAAGTAACCTAATATACAGA---TTGATATGCGTACTTACTATCTGGACTCCTTTGTACAGAAGTAAATGTGAGAACACTACTAATGC---------------TTCATCCAACGCTGAATCTACAAGCGTTATCACTA---CACTAACTGCAGATACCTCATCTACATACCAATCATTACCTGCATCAACTTCAACAT------------CTATCTCTAATACAG------CTATCACAAGCACTCGCATGGCTTCCAGTCCAAATACA---TCGTATGTTTCCACCTCTTACGATAACACATCGA------------------------CGTCAGTGATGATTACAAGCACATCCACGGAAACTAACGTTAGTTCTACGGAAATAACCGTTACATCTAT---------AACTACTTCAGCCACAACCAACTCCACAGTGAATGTGACAGACATGACCGCAACAATTATTA---CATCTCCAGCCGCAACAACAAACGCAACCATTAGTAACACATCATTCG---TAAATTGCGAAATGTGGAA---CAC---TACGCTTAAAAATAATACAAAAGACCGTG---ACGAGTGCCAACCAATAAAAGTAAATAAAACAGATATAATAGCAGAAGAGTGGACCAATGTTACTATACAAAGCAATTTCACGATACCGCACTGTAACAAGGTAATTTGGATACGACACTATAATCGAACCACACACGGAAACTACTTCCCAATACAATATAGACGTCC----------TTTTATTAGAAGTGCTTTGTACTCACTT--GAAATTTGCGGCCAAACGTACACCCACAGTCTTCTACACTTATATGATTTGTGCATCTCGT---GTGACAACGGAACACTACATCTTTACGGTGTAAACACAACTCATTCAGGTAGATATACTGCACGTTGCCATACTTATGAATATCATAGCAACCACGGTAAACATGAGGACAAAAACTTTAATTTGATTATAAATCCTAGAAATAATACTAATTATACCAACGACATCTGGATATGTC-----------CACAAC----------CTACGAAAGACGAAACCCAAGCAGACAACCAAAGTAAAGAAAAAC---ACTTAACAACGACGGACAATTCACTTTCTCATAAACCAAACCATTACTCAAGAACCTCACACCGCAGTGCCTGGACTGTCGCGTTGCTTTGTATTGCCTGTATACTCCTATTTTTTTTCCGACGTTTTTTTAACAAAAAATACCGTACGCTGGATGATACTGTCAGCGAATCCGAATTTATTGTACGGTATAACCCAGAGCATGAAGATTGA

HANRTR4 ATGCGTGGCATTTGGAAGCCAAGAAGTAACCTAATATACAGA---TTGATATGCGTACTTACTATCTGGACTCCTTTGTACAGAAGTAAATGTGAGAACACTACTAATGC---------------TTCATCCAACGCTGAATCTACAAGCGTTATCACTA---CACTAACTGCAGATACCTCATCTACATACCAATCATTACCTGCATCAACTTCAACAT------------CTATCTCTAATACAG------CTATCACAAGCACTCGCATGGCTTCCAGTCCAAATACA---TCGTATGTTTCCACCTCTTACGATAACACATCGA------------------------CGTCAGTGATGATTACAAGCACATCCACGGAAACTAACGTTAGTTCTACGGAAATAACCGTTACATCTAT---------AACTACTTCAGCCACAACCAACTCCACAGTGAATGTGACAGACATGACCGCAACAATTATTA---CATCTCCAGCCGCAACAACAAACGCAACCATTAGTAACACATCATTCG---TAAATTGCGAAATGTGGAA---CAC---TACGCTTAAAAATAATACAAAAGACCGTG---ACGAGTGCCAACCAATAAAAGTAAATAAAACAGATATAATAGCAGAAGAGTGGACCAATGTTACTATACAAAGCAATTTCACGATACCGCACTGTAACAAGGTAATTTGGATACGACACTATAATCGAACCACACACGGAAACTACTTCCCAATACAATATAGACGTCC----------TTTTATTAGAAGTGCTTTGTACTCACTT--GAAATTTGCGGCCAAACGTACACCCACAGTCTTCTACACTTATATGATTTGTGCATCTCGT---GTGACAACGGAACACTACATCTTTACGGTGTAAACACAACTCATTCAGGTAGATATACTGCACGTTGCCATACTTATGAATATCATAGCAACCACGGTAAACATGAGGACAAAAACTTTAATTTGATTATAAATCCTAGAAATAATACTAATTATACCAACGACATCTGGATATGTC-----------CACAAC----------CTACGAAAGACGAAACCCAAGCAGACAACCAAAGTAAAGAAAAAC---ACTTAACAACGACGGACAATTCACTTTCTCATAAACCAAACCATTACTCAAGAACCTCACACCGCAGTGCCTGGACTGTCGCGTTGCTTTGTATTGCCTGTATACTCCTATTTTTTTTCCGACGTTTTTTTAACAAAAAATACCGTACGCTGGATGATACTGTCAGCGAATCCGAATTTATTGTACGGTATAACCCAGAGCATGAAGATTGA

HANSCTR8 ATGCGTGGCATTTGGAAGCCAAGAAGTAACCTAATATACAGA---TTGATATGCGTACTTACTATCTGGACTCCTTTGTACAGAAGTAAATGTGAGAACACTACTAATGC---------------TTCATCCAACGCTGAATCTACAAGCGTTATCACTA---CACTAACTGCAGATACCTCATCTACATACCAATCATTACCTGCATCAACTTCAACAT------------CTATCTCTAATACAG------CTATCACAAGCACTCGCATGGCTTCCAGTCCAAATACA---TCGTATGTTTCCACCTCTTACGATAACACATCGA------------------------CGTCAGTGATGATTACAAGCACATCCACGGAAACTAACGTTAGTTCTACGGAAATAACCGTTACATCTAT---------AACTACTTCAGCCACAACCAACTCCACAGTGAATGTGACAGACATGACCGCAACAATTATTA---CATCTCCAGCCGCAACAACAAACGCAACCATTAGTAACACATCATTCG---TAAATTGCGAAATGTGGAA---CAC---TACGCTTAAAAATAATACAAAAGACCGTG---ACGAGTGCCAACCAATAAAAGTAAATAAAACAGATATAATAGCAGAAGAGTGGACCAATGTTACTATACAAAGCAATTTCACGATACCGCACTGTAACAAGGTAATTTGGATACGACACTATAATCGAACCACACACGGAAACTACTTCCCAATACAATATAGACGTCC----------TTTTATTAGAAGTGCTTTGTACTCACTT--GAAATTTGCGGCCAAACGTACACCCACAGTCTTCTACACTTATATGATTTGTGCATCTCGT---GTGACAACGGAACACTACATCTTTACGGTGTAAACACAACTCATTCAGGTAGATATACTGCACGTTGCCATACTTATGAATATCATAGCAACCACGGTAAACATGAGGACAAAAACTTTAATTTGATTATAAATCCTAGAAATAATACTAATTATACCAACGACATCTGGATATGTC-----------CACAAC----------CTACGAAAGACGAAACCCAAGCAGACAACCAAAGTAAAGAAAAAC---ACTTAACAACGACGGACAATTCACTTTCTCATAAACCAAACCATTACTCAAGAACCTCACACCGCAGTGCCTGGACTGTCGCGTTGCTTTGTATTGCCTGTATACTCCTATTTTTTTTCCGACGTTTTTTTAACAAAAAATACCGTACGCTGGATGATACTGTCAGCGAATCCGAATTTATTGTACGGTATAACCCAGAGCATGAAGATTGA

HANRTR9 ATGCGTGGCATTTGGAAGCCAAGAAGTAACCTAATATACAGA---TTGATATGCGTACTTACTATCTGGACTCCTTTGTACAGAAGTAAATGTGAGAACACTACTAATGC---------------TTCATCCAACGCTGAATCTACAAGCGTTATCACTA---CACTAACTGCAGATACCTCATCTACATACCAATCATTACCTGCATCAACTTCAACAT------------CTATCTCTAATACAG------CTATCACAAGCACTCGCATGGCTTCCAGTCCAAATACA---TCGTATGTTTCCACCTCTTACGATAACACATCGA------------------------CGTCAGTGATGATTACAAGCACATCCACGGAAACTAACGTTAGTTCTACGGAAATAACCGTTACATCTAT---------AACTACTTCAGCCACAACCAACTCCACAGTGAATGTGACAGACATGACCGCAACAATTATTA---CATCTCCAGCCGCAACAACAAACGCAACCATTAGTAACACATCATTCG---TAAATTGCGAAATGTGGAA---CAC---TACGCTTAAAAATAATACAAAAGACCGTG---ACGAGTGCCAACCAATAAAAGTAAATAAAACAGATATAATAGCAGAAGAGTGGACCAATGTTACTATACAAAGCAATTTCACGATACCGCACTGTAACAAGGTAATTTGGATACGACACTATAATCGAACCACACACGGAAACTACTTCCCAATACAATATAGACGTCC----------TTTTATTAGAAGTGCTTTGTACTCACTT--GAAATTTGCGGCCAAACGTACACCCACAGTCTTCTACACTTATATGATTTGTGCATCTCGT---GTGACAACGGAACACTACATCTTTACGGTGTAAACACAACTCATTCAGGTAGATATACTGCACGTTGCCATACTTATGAATATCATAGCAACCACGGTAAACATGAGGACAAAAACTTTAATTTGATTATAAATCCTAGAAATAATACTAATTATACCAACGACATCTGGATATGTC-----------CACAAC----------CTACGAAAGACGAAACCCAAGCAGACAACCAAAGTAAAGAAAAAC---ACTTAACAACGACGGACAATTCACTTTCTCATAAACCAAACCATTACTCAAGAACCTCACACCGCAGTGCCTGGACTGTCGCGTTGCTTTGTATTGCCTGTATACTCCTATTTTTTTTCCGACGTTTTTTTAACAAAAAATACCGTACGCTGGATGATACTGTCAGCGAATCCGAATTTATTGTACGGTATAACCCAGAGCATGAAGATTGA

HAN2 ATGCGTGGCATTTGGAAGCCAAGAAGTAACCTAATATACAGA---TTGATATACGTACTTACTATCTGGACTCCTTTGTACAGAAGTAAATGTGAGAACACTACTAATGC---------------TTCATCCAACGCTGAATCTACAAGCGTTATCACTA---CACTAACTGCAGATACCTCATCTACATACCAATCATTACCTGCATCAACTTCAACAT------------CTATCTCTAATACAG------CTATCACAAGCACTCGCATGGCTTCCAGTCCAAATACA---TCGTATGTTTCCACCTCTTACGATAACACATCGA------------------------CGTCAGTGATGATTACAAGCACATCCACGGAAACTAACGTTAGTTCTACGGAAATAACCGTTACATCTAT---------AACTACTTCAGCCACAACCAACTCCACAGTGAATGTGACAGACATGACCGCAACAATTATTA---CATCTCCAGCCGCAACAACAAACGCAACCATTAGTAACACATCATTCG---TAAATTGCGAAATGTGGAA---CAC---TACGCTTAAAAATAATACAAAAGACCGTG---ACGAGTGCCAACCAATAAAAGTAAATAAAACAGATATAATAGCAGAAGAGTGGACCAATGTTACTATACAAAGCAATTTCACGATACCGCACTGTAACAAGGTAATTTGGATACGACACTATAATCGAACCACACACGGAAACTACTTCCCAATACAATATAGACGTCC----------TTTTATTAGAAGTGCTTTGTACTCACTT--GAAATTTGCGGCCAAACGTACACCCACAGTCTTCTACACTTATATGATTTGTGCATCTCGT---GTGACAACGGAACACTACATCTTTACGGTGTAAACACAACTCATTCAGGTAGATATACTGCACGTTGCCATACTTATGAATATCATAGCAACCACGGTAAACATGAGGACAAAAACTTTAATTTGATTATAAATCCTAGAAATAATACTAATTATACCAACGACATCTGGATATGTC-----------CACAAC----------CTACGAAAGACGAAACCCAAGCAGACAACCAAAGTAAAGAAAAAC---ACTTAACAACGACGGACAATTCACTTTCTCATAAACCAAACCATTACTCAAGAACCTCACACCGCAGTGCCTGGACTGTCGCGTTGCTTTGTATTGCCTGTATACTCCTATTTTTTTTCCGACGTTTTTTTAACAAAAAATACCGTACGCTGGATGATACTGTCAGCGAATCCGAATTTATTGTACGGTATAACCCAGAGCATGAAGATTGA

BE/13/2010 G6 ATGCGTGGCATTTGGAAGCCAGGAAGTAACCTAATATACAGA---TTGATATACGTACTTACTATTTGGACTCCTTTGTATAGAAGTAAATGTGAGAACACTACTAATTC---------------TTCATCCACCGCTGAATCTACAAGCATTATCACTACTACATTGACTACAGATCTTTCATCTACATATCAAATATCATCTGCACCAACTTCAACAC------------CTATTTCTAACACAG------CTATTACAAGTACTCTCATCACTTCCAATCCAAATACA---TCGTATGTTTTCACCTCTTACAACACAT------------------------------------CGACAATTTTAAGCACATCAACGGAAACTAACGTTGGTTCTACGGAAATAAACGTTACTTCCAT---------GAATACTTCGGCAACAAGCAACGTCACATCAAATGTCACAGAGATAAAAGTAACAACAGTCACCATAATTCCAACCGTAATAACAAATACAACTATCAGTAACA---CATCTG---TAAGTTGTGAAATGTTCAA---TA------CGACACTTAATAATACACAAGACCGTG---ATGAATGCAAACCAATAAAAGTAAATAAAACAGATATCAAAGCAGAAGAATGGACCAATGTTACTATACAAAGCAATTTCACAATACCGCACTGTCACAAGGTGGTTTGGATACGACAATATAATCTAACCACACATGGAGATTACTTCCCAACACGATATAAACGTCC----------TTTTGTTAAAGGTGCCTTGTATTCACGT--GAAATATGCGGCCATACGTACACACACAATTTTCTACACTCATATGATCTATGCATCTCGT---GCGACAACGGAACACTACATCTCTACGGTGTAAACACAACGCATTCAGGTAGATATACTGCACGGTGTCATATCTATGAACATCATAATAATCACGGCAATTACAATGATAAAAATTTTAATCTGATTATATATCCTAGAAATAATACTAACAATACCAACGGCATCTGGATATGTC-----------CACGAC----------CTACAAAAGACGAAGCCCAAGAAAACAATCAGGGTGAAGAAAAAC---ATCTAACAACGACGGACAACTCCGTTTCCCATAAACGAAACCATTACCCAAGAACCTCACACCGCAGTGCCTGGACTGTTACGTTGCTTTGTGTCGCCTGTATACTCCTATTTTTTTGTCGACGTCTTTTTAACAAAAAATACCGTATATTGGATGACACCGTCAGCGAATCGGAATTTATTGTACGGTATAACCCAGAGCATGAAGATTGA

HAN24 ATGCATGGCATTTGGAAACCAGGAAGCAACTTAATATACAGA---TGGATATACGTACTTACTATTTGGACTCCTTTGTATAGAAGTAAATGTGAGAACACTGCTAATTC---------------TTCATCCACCGCTGAATCTACAAGCATTATCACTACTACATTGACTACAGATCTTTCATCTACATATCAAATATCATCTGCATCAACTTCAACAC------------CTATTTCTAACACAG------CTACTACAAGCACTCTCATCACTTCTAATCCAAATACA---TCGTATGTTTTCACCTCTTACAATACAT------------------------------------CGACAATTTTAAGCACATCAACGGAAACTAACGTTGGTTCTACGGAAATAAACGTTACTTCCAT---------GAATACTTCGGCAACAAGCAACGTCACATCAAATGTCACAGAGATAAAAGTAACAACAGTCACCATAATTCCAACCGTAATAACAAATACAACTATCAGTAACA---CATCTG---TAAGTTGTGAAATGTTCAA---TA------CGACAATTAATAATACACAAGACCGTG---ATGAATGTAAACCAATAAAAGTAAATAAAACAGATATCAAAGCAGAAGAATGGACCAATGTTACTATACAAAGCAATTTCACAATACCGCACTGTCACAAGGTGGTTTGGATACGACAATATAATCTAACCACACATGGAGATTACTTCCCAACACGATATAAACGTCC----------TTTTGTTAAAGGTGCCTTGTATTCACGT--GAAATATGCGGCCATACATACACACACAATCTTCTACACTCATATGACTTATGCATCTCGT---GTGACAACGGAACACTACATCTCTACGGTGTAAACACAACTCATTCAGGTAGATATACTGCACGTTGTCATATTTATGAACATAA------TCACGGCACACATGAGGACAAAAATTTTAATCTGATTATATATCCTAGAAATAATACTAACAATACCAACGGTATCTGGATATGTC-----------CACGAC----------CTACAAAAGACGAAGCCCAAGAAAACAACCAGAGTGAAGAAAAAC---ATCTAACAACGACGGACAACTCTGTTTCCCATAAACGAAACCATTACCCAAGAACCTCACACCGCAGTGCCTGGACTGTTACGTTGCTTTGTGTCGCCTGTATACTCCTATTTTTTTTTCGACGTCTTTTTAACAAAAAATACCGTATGTTGGATGACACCGTCAGCGAATCGGAATTTATTGTACGGTATAACCCAGAGCATGAAGATTGA

HAN39 ATGCCATGCATTTGGAAACCAGGAAGCAACTTAATATACAGA---TGGATATACGTACTTACTATTTGGACTCCTTTGTATAGAAGTAAATGTGAGAACACTGCTAATTC---------------TTCATCCACCGCTGAATCTACAAGCATTATCACTACTACATTGACTACAGATCTTTCATCTACATATCAAATATCATCTGCATCAACTTCAACAC------------CTATTTCTAACACAG------CTACTACAAGCACTCTCATCACTTCTAATCCAAATACA---TCGTATGTTTTCACCTCTTACAATACAT------------------------------------CGACAATTTTAAGCACATCAACGGAAACTAACGTTGGTTCTACGGAAATAAACGTTACTTCCAT---------GAATACTTCGGCAACAAGCAACGTCACATCAAATGTCACAGAGATAAAAGTAACAACAGTCACCATAATTCCAACCGTAATAACAAATACAACTATCAGTAACA---CATCTG---TAAGTTGTGAAATGTTCAA---TA------CGACAATTAATAATACACAAGACCGTG---ATGAATGTAAACCAATAAAAGTAAATAAAACAGATATCAAAGCAGAAGAATGGACCAATGTTACTATACAAAGCAATTTCACAATACCGCACTGTCACAAGGTGGTTTGGATACGGCAATATAATCTAACCACACATGGAGATTACTTCCCAACACGATATAAACGTCC----------TTTTGTTAAAGGTGCCTTGTATTCACGT--GAAATATGCGGCCATACATACACACACAATCTTCTACACTCATATGACTTATGCATCTCGT---GCGACAACGGAACACTACATCTCTACGGTGTAAACACAACTCATTCAGGTAGATATACTGCACGTTGTCATATTTATGAACATAATAA---TCACGGCACACATGAGGACAAAAATTTTAATCTGATTATATATCCTAGAAATAATACTAACAATACCAACGGTATCTGGATATGTC-----------CACGAC----------CTACAAAAGACGAAGCCCAAGAAAACAACCAGAGTGAAGAAAAAC---ATCTAACAACGACGGACAACTCTGTTTCCCATAAACGAAACCATTACCCAAGAACCTCACACCGCAGTGCCTGGACTGTTACGTTGCTTTGTGTCGCCTGTATACTCCTATTTTTTTTTCGACGTCTTTTTAACAAAAAATACCGTATGTTGGATGACACCGTCAGCGAATCGGAATTTATTGTACGGTATAACCCAGAGCATGAAGATTGA

HAN28 ATGCATGGCATTTGGAAACCAGGAAGCAACTTAATATACAGA---TGGATATACGTACTTACTATTTGGACTCCTTTGTATAGAAGTAAATGTGAGAACACTGCTAATTC---------------TTCATCCACCGCTGAATCTACAAGCATTATCACTACTACATTGACTACAGATCTTTCATCTACATATCAAATATCATCTGCATCAACTTCAACAC------------CTATTTCTAACACAG------CTACTACAAGCACTCTCATCACTTCTAATCCAAATACA---TCGTATGTTTTCACCTCCTACAATACAT------------------------------------CGACAATTTTAAGCACATCAACGGAAACTAACGTTGGTTCTACGGAAATAAACGTTACTTCCAT---------GAATACTTCGGTAACAAGCAACGTCACATCAAATGTCACAGAGATAAAAGTAACAACAGTCACCATAATTCCAACCGTAATAACAAATACAACTATCAGTAACA---CATCTG---TAAGTTGTGAAATGTTCAA---TA------CGACAATTAATAATACACAAGACCGTG---ATGAATGTAAACCAATAAAAGTAAATAAAACAGATATCAAAGCAGAAGAATGGACCAATGTTACTATACAAAGCAATTTCACAATACCGCACTGTCACAAGGTGGTTTGGATACGACAATATAATCTAACCACACATGGAGATTACTTCCCAACACGATATAAACGTCC----------TTTTGTTAAAGGTGCCTTGTATTCACGT--GAAATATGCGGCCATACATACACACACAATCTTCTACACTCATATGACTTATGCATCTCGT---GCGACAACGGAACACTACATCTCTACGGTGTAAACACAACTCATTCAGGTAGATATACTGCACGTTGTCATATTTATGAACATAATAA---TCACGGCACACATGAGGACAAAAATTTTAATCTGATTATATATCCTAGAAATAATACTAACAATACCAACGGTATCTGGATATGTC-----------CACGAC----------CTACAAAAGACGAAGCCCAAGAAAACAACCAGGGTGAAGAAAAAC---ATCTAACAACGACGGACAACTCTGTTTCCCATAAACGAAACCATTACCCAAGAACCTCACACCGCAGTGCCTGGACTGTTACGTTGCTTTGTGTCGCCTGTATACTCCTATTTTTTTTTCGACGTCTTTTTAACAAAAAATACCGTATGTTGGATGACACCGTCAGCGAATCGGAATTTATTGTACGGTATAACCCAGAGCATGAAGATTGA

HANRTR6 ATGCATGGCATTTGGAAACCAGGAAGCAACTTAATATACAGA---TGGATATACGTACTTACTATTTGGACTCCTTTGTATAGAAGTAAATGTGAGAACACTGCTAATTC---------------TTCATCCACCGCTGAATCTACAAGCATTATCACTACTACATTGACTACAGATCTTTCATCTACATATCAAATATCATCTGCATCAACTTCAACAC------------CTATTTCTAACACAG------CTACTACAAGCACTCTCATCACTTCTAATCCAAATACA---TCGTATGTTTTCACCTCTTACAATACAT------------------------------------CGACAATTTTAAGCACATCAACGGAAACTAACGTTGGTTCTACGGAAATAAACGTTACTTCCAT---------GAATACTTCGGCAACAAGCAACGTCACATCAAATGTCACAGAGATAAAAGTAACAACAGTCACCATAATTCCAACCGTAATAACAAATACAACTATCAGTAATA---CATCTG---TAAGTTGTGAAATGTTCAA---TA------CGACAATTAATAATACACAAGACCGTG---ATGAATGTAAACCAATAAAAGTAAATAAAACAGATATCAAAGCAGAAGAATGGACCAATGTTACTATACAAAGCAATTTCACAATACCGCACTGTCACAAGGTGGTTTGGATACGACAATATAATCTAACCACACATGGAGATTACTTCCCAACACGATATAAACGTCC----------TTTTGTTAAAGGTGCCTTGTATTCACGT--GAAATATGCGGCCATACATACACACACAATCTTCTACACTCATATGACTTATGCATCTCGT---GTGACAACGGAACACTACATCTCTACGGTGTAAACACAACTCATTCAGGTAGATATACTGCACGTTGTCATATTTATGAACATAATAA---TCACGGCACACATGAGGACAAAAATTTTAATCTGATTATATATCCTAGAAATAATACTAACAATACCAACGGTATCTGGATATGTC-----------CACGAC----------CTACAAAAGACGAAGCCCAAGAAAACAACCAGAGTGAAGAAAAAC---ATCTAACAACGACGGACAACTCTGTTTCCCATAAACGAAACCATTACCCAAGAACCTCACACCGCAGTGCCTGGACTGTTACGTTGCTTTGTGTCGCCTGTATACTCCTATTTTTTTTTCGACGTCTTTTTAACAAAAAATACCGTATGTTGGATGACACCGTCAGCGAATCGGAATTTATTGTACGGTATAACCCAGAGCATGAAGATTGA

HANRTR8 ATGCATGGCATTTGGAAACCAGGAAGCAATTTAATATACAGA---TGGATATACGTACTTACTATTTGGACTCCTTTGTATAGAAGTAAATGTGAGAACACTGCTAATTC---------------TTCATCCACCGCTGAATCTACAAGCATTATCACTACTACATTGACTACAGATCTTTCATCTACATATCAAATATCATCTGCATCAACTTCAACAC------------CTATTTCTAACACAG------CTACTACAAGCACTCTCATCACTTCTAATCCAAATACA---TCGTATGTTTTCACCTCTTACAATACAT------------------------------------CGACAATTTTAAGCACATCAACGGAAACTAACGTTGGTTCTACGGAAATAAACGTTACTTCCAT---------GAATACTTCGGCAACAAGCAACGTCACATCAAATGTCACAGAGATAAAAGTAACAACAGTCACCATAATTCCAACCGTAATAACAAATACAACTATCAGTAACA---CATCTG---TAAGTTGTGAAATGTTCAA---TA------CGACAATTAATAATACACAAGACCGTG---ATGAATGTAAACCAATAAAAGTAAATAAAACAGATATCAAAGCAGAAGAATGGACCAATGTTACTATACAAAGCAATTTCACAATACCGCACTGTCACAAGGTGGTTTGGATACGACAATATAATCTAACCACACATGGAGATTACTTCCCAACACGATATAAACGTCC----------TTTTGTTAAAGGTGCCTTGTATTCACGT--GAAATATGCGGCCATACATACACACACAATCTTCTACACTCATATGACTTATGCATCTCGT---GCGACAACGGAACACTACATCTCTACGGTGTAAACACAACTCATTCAGGTAGATATACTGCACGTTGTCATATTTATGAACATAATAA---TCACGGCACACATGAGGACAAAAATTTTAATCTGATTATATATCCTAGAAATAATACTAACAATACCAACGGTATCTGGATATGTC-----------CACGAC----------CTACAAAAGACGAAGCCCAAGAAAACAACCAGAGTGAAGAAAAAC---ATCTAACAACGACGGACAACTCTGTTTCCCATAAACGAAACCATTACCCAAGAACCTCACACCGCAGTGCCTGGACTGTTACGTTGCTTTGTGTCGCCTGTATACTCCTATTTTTTTTTCGACGTCTTTTTAACAAAAAATACCGTATGTTGGATGACACCGTCAGCGAATCGGAATTTATTGTACGGTATAACCCAGAGCATGAAGATTGA

3301 ATGCATGGCATTTGGAAACCAGGAAGCAACTTAATATACAGA---TGGATATACGTACTTACTATTTGGACTCCTTTGTATAGAAGTAAATGTGAGAACACTGCTAATTC---------------TTCATCCACCGCTGAATCTACAAGCATTATCACTACTACATTGACTACAGATCTTTCATCTACATATCAAATATCATCTGCATCAACTTCAACAC------------CTATTTCTAACACAG------CTACTACAAGCACTCTCATCACTTCTAATCCAAATACA---TCGTATGTTTTCACCTCTTACAATACAT------------------------------------CGACAATTTTAAGCACATCAACGGAAACTAACGTTGGTTCTACGGAAATAAACGTTACTTCCAT---------GAATACTTCGGCAACAAGCAACGTCACATCAAATGTCACAGAGATAAAAGTAACAACAGTCACCATAATTCCAACCGTAATAACAAATACAACTATCAGTAACA---CATCTG---TAAGTTGTGAAATGTTCAA---TA------CGACAATTAATAATACACAAGACCGTG---ATGAATGTAAACCAATAAAAGTAAATAAAACAGATATCAAAGCAGAAGAATGGACCAATGTTACTATACAAAGCAATTTCACAATACCGCACTGTCACAAGGTGGTTTGGATACGACAATATAATCTAACCACACATGGAGATTACTTCCCAACACGATATAAACGTCC----------TTTTGTTAAAGGTGCCTTGTATTCACGT--GAAATATGCGGCCATACATACACACACAATCTTCTACACTCATATGACTTATGCATCTCGT---GCGACAACGGAACACTACATCTCTACGGTGTAAACACAACTCATTCAGGTAGATATACTGCACGTTGTCATATTTATGAACATAATAA---TCACGGCACACATGAGGACAAAAATTTTAATCTGATTATATATCCTAGAAATAATACTAACAATACCAACGGTATCTGGATATGTC-----------CACGAC----------CTACAAAAGACGAAGCCCAAGAAAACAACCAGAGTGAAGAAAATC---ATCTAACAACGACGGACAACTCTGTTTCCCATAAACGAAACCATTACCCAAGAACCTCACACCGCAGTGCCTGGACTGTTACGTTGCTTTGTGTCGCCTGTATACTCCTATTTTTTTTTCGACGTCTTTTTAACAAAAAATACCGTATGTTGGATGACACCGTCAGCGAATCGGAATTTATTGTACGGTATAACCCAGAGCATGAAGATTGA

PAV12 ATGCATGGCATTTGGAAACCAGGAAGCAACTTAATATACAGA---TGGATATACGTACTTACTATTTGGACTCCTTTGTATAGAAGTAAATGTGAGAACACTGCTAATTC---------------TTCATCCACCGCTGAATCTACAAGCATTATCACTACTACATTGACTACAGATCTTTCATCTACATATCAAATATCATCTGCATCAACTTCAACAC------------CTATTTCTAACACAG------CTACTACAAGCACTCTCATCACTTCTAATCCAAATACA---TCGTATGTTTTCACCTCTTACAATACAT------------------------------------CGACAATTTTAAGCACATCAACGGAAACTAACGTTGGTTCTACGGAAATAAACGTTACTTCCAT---------GAATACTTCGGCAACAAGCAACGTCACATCAAATGTCACAGAGATAAAAGTAACAACAGTCACCATAATTCCAACCGTAATAACAAATACAACTATCAGTAACA---CATCTG---TAAGTTGTGAAATGTTCAA---TA------CGACAATTAATAATACACAAGACCGTG---ATGAATGTAAACCAATAAAAGTAAATAAAACAGATATCAAAGCAGAAGAATGGACCAATGTTACTATACAAAGCAATTTCACAATACCGCACTGTCACAAGGTGGTTTGGATACGACAATATAATCTAACCACACATGGAGATTACTTCCCAACACGATATAAACGTCC----------TTTTGTTAAAGGTGCCTTGTATTCACGT--GAAATATGCGGCCATACATACACACACAATCTTCTACACTCATATGACTTATGCATCTCGT---GCGACAACGGAACACTACATCTCTACGGTGTAAACACAACTCATTCAGGTAGATATACTGCACGTTGTCATATTTATGAACATAATAA---TCACGGCACACATGAGGACAAAAATTTTAATCTGATTATATATCCTAGAAATAATACTAACAATACCAACGGTATCTGGATATGTC-----------CACGAC----------CTACAAAAGACGAAGCCCAAGAAAACAACCAGAGTGAAGAAAAAC---ATCTAACAACGACGGACAACTCTGTTTCCCATAAACGAAACCATTACCCAAGAACCTCACACCGCAGTGCCTGGACTGTTACGTTGCTTTGTGTCGCCTGTATACTCCTATTTTTTTTTCGACGTCTTTTTAACAAAAAATACCGTATGTTGGATGACACCGTCAGCGAATCGGAATTTATTGTACGGTATAACCCAGAGCATGAAGATTGA

BE/45/2011 ATGCATGGCATTTGGAAACCAGGAAGCAACTTAATATACAGA---TGGATATACGTACTTACTATTTGGACTCCTTTGTATAGAAGTAAATGTGAGAACACTGCTAATTC---------------TTCATCCACCGCTGAATCTACAAGCATTATCACTACTACATTGACTACAGATCTTTCATCTACATATCAAATATCATCTGCATCAACTTCAACAC------------CTATTTCTAACACAG------CTACTACAAGCACTCTCATCACTTCTAATCCAAATACA---TCGTATGTTTTCACCTCTTACAATACAT------------------------------------CGACAATTTTAAGCACATCAACGGAAACTAACGTTGGTTCTACGGAAATAAACGTTACTTCCAT---------GAATACTTCGGCAACAAGCAACGTCACATCAAATGTCACAGAGATAAAAGTAACAACAGTCACCATAATTCCAACCGTAATAACAAATACAACTATCAGTAACA---CATCTG---TAAGTTGTGAAATGTTCAA---TA------CGACAATTAATAATACACAAGACCGTG---ATGAATGTAAACCAATAAAAGTAAATAAAACAGATATCAAAGCAGAAGAATGGACCAATGTTACTATACAAAGCAATTTCACAATACCGCACTGTCACAAGGTGGTTTGGATACGACAATATAATCTAACCACACATGGAGATTACTTCCCAACACGATATAAACGTCC----------TTTTGTTAAAGGTGCCTTGTATTCACGT--GAAATATGCGGCCATACATACACACACAATCTTCTACACTCATATGACTTATGCATCTCGT---GCGACAACGGAACACTACATCTCTACGGTGTAAACACAACTCATTCAGGTAGATATACTGCACGTTGTCATATTTATGAACATAATAA---TCACGGCACACATGAGGACAAAAATTTTAATCTGATTATATATCCTAGAAATAATACTAACAATACCAACGGTATCTGGATATGTC-----------CACGAC----------CTACAAAAGACGAAGCCCAAGAAAACAACCAGAGTGAAGAAAAAC---ATCTAACAACGACGGACAACTCTGTTTCCCATAAACGAAACCATTACCCAAGAACCTCACACCGCAGTGCCTGGACTGTTACGTTGCTTTGTGTCGCCTGTATACTCCTATTTTTTTTTCGACGTCTTTTTAACAAAAAATACCGTATGTTGGATGACACCGTCAGCGAATCGGAATTTATTGTACGGTATAACCCAGAGCATGAAGATTGA

CZ/3/2012 ATGCATGGCATTTGGAAACCAGGAAGCAACTTAATATACAGA---TGGATATACGTACTTACTATTTGGACTCCTTTGTATAGAAGTAAATGTGAGAACACTGCTAATTC---------------TTCATCCACCGCTGAATCTACAAGCATTATCACTACTACATTGACTACAGATCTTTCATCTACATATCAAATATCATCTGCATCAACTTCAACAC------------CTATTTCTAACACAG------CTACTACAAGCACTCTCATCACTTCTAATCCAAATACA---TCGTATGTTTTCACCTCTTACAATACAT------------------------------------CGACAATTTTAAGCACATCAACGGAAACTAACGTTGGTTCTACGGAAATAAACGTTACTTCCAT---------GAATACTTCGGCAACAAGCAACGTCACATCAAATGTCACAGAGATAAAAGTAACAACAGTCACCATAATTCCAACCGTAATAACAAATACAACTATCAGTAACA---CATCTG---TAAGTTGTGAAATGTTCAA---TA------CGACAATTAATAATACACAAGACCGTG---ATGAATGTAAACCAATAAAAGTAAATAAAACAGATATCAAAGCAGAAGAATGGACCAATGTTACTATACAAAGCAATTTCACAATACCGCACTGTCACAAGGTGGTTTGGATACGACAATATAATCTAACCACACATGGAGATTACTTCCCAACACGATATAAACGTCC----------TTTTGTTAAAGGTGCCTTGTATTCACGT--GAAATATGCGGCCATACATACACACACAATCTTCTACACTCATATGACTTATGCATCTCGT---GCGACAACGGAACACTACATCTCTACGGTGTAAACACAACTCATTCAGGTAGATATACTGCACGTTGTCATATTTATGAACATAATAA---TCACGGCACACATGAGGACAAAAATTTTAATCTGATTATATATCCTAGAAATAATACTAACAATACCAACGGTATCTGGATATGTC-----------CACGAC----------CTACAAAAGACGAAGCCCAAGAAAACAACCAGAGTGAAGAAAAAC---ATCTAACAACGACGGACAACTCTGTTTCCCATAAACGAAACCATTACCCAAGAACCTCACACCGCAGTGCCTGGACTGTTACGTTGCTTTGTGTCGCCTGTATACTCCTATTTTTTTTTCGACGTCTTTTTAACAAAAAATACCGTATGTTGGATGACACCGTCAGCGAATCGGAATTTATTGTACGGTATAACCCAGAGCATGAAGATTGA

BE/13/2012 ATGCATGGCATTTGGAAACCAGGAAGCAACTTAATATACAGA---TGGATATACGTACTTACTATTTGGACTCCTTTGTATAGAAGTAAATGTGAGAACACTGCTAATTC---------------TTCATCCACCGCTGAATCTACAAGCATTATCACTACTACATTGACTACAGATCTTTCATCTACATATCAAATATCATCTGCATCAACTTCAACAC------------CTATTTCTAACACAG------CTACTACAAGCACTCTCATCACTTCTAATCCAAATACA---TCGTATGTTTTCACCTCTTACAATACAT------------------------------------CGACAATTTTAAGCACATCAACGGAAACTAACGTTGGTTCTACGGAAATAAACGTTACTTCCAT---------GAATACTTCGGCAACAAGCAACGTCACATCAAATGTCACAGAGATAAAAGTAACAACAGTCACCATAATTCCAACCGTAATAACAAATACAACTATCAGTAACA---CATCTG---TAAGTTGTGAAATGTTCAA---TA------CGACAATTAATAATACACAAGACCGTG---ATGAATGTAAACCAATAAAAGTAAATAAAACAGATATCAAAGCAGAAGAATGGACCAATGTTACTATACAAAGCAATTTCACAATACCGCACTGTCACAAGGTGGTTTGGATACGACAATATAATCTAACCACACATGGAGATTACTTCCCAACACGATATAAACGTCC----------TTTTGTTAAAGGTGCCTTGTATTCACGT--GAAATATGCGGCCATACATACACACACAATCTTCTACACTCATATGACTTATGCATCTCGT---GCGACAACGGAACACTACATCTCTACGGTGTAAACACAACTCATTCAGGTAGATATACTGCACGTTGTCATATTTATGAACATAATAA---TCACGGCACACATGAGGACAAAAATTTTAATCTGATTATATATCCTAGAAATAATACTAACAATACCAACGGTATCTGGATATGTC-----------CACGAC----------CTACAAAAGACGAAGCCCAAGAAAACAACCAGAGTGAAGAAAAAC---ATCTAACAACGACGGACAACTCTGTTTCCCATAAACGAAACCATTACCCAAGAACCTCACACCGCAGTGCCTGGACTGTTACGTTGCTTTGTGTCGCCTGTATACTCCTATTTTTTTTTCGACGTCTTTTTAACAAAAAATACCGTATGTTGGATGACACCGTCAGCGAATCGGAATTTATTGTACGGTATAACCCAGAGCATGAAGATTGA

JER3230 ATGCATGGCATTTGGAAACCAGGAAGCAACTTAATATACAGA---TGGATATACGTACTTACTATTTGGACTCCTTTGTATAGAAGTAAATGTGAGAACACTGCTAATTC---------------TTCATCCACCGCTGAATCTACAAGCATTATCACTACTACATTGACTACAGATCTTTCATCTACATATCAAATATCATCTGCATCAACTTCAACAC------------CTATTTCTAACACAG------CTACTACAAGCACTCTCATCACTTCTAATCCAAATACA---TCGTATGTTTTCACCTCTTACAATACAT------------------------------------CGACAATTTTAAGCACATCAACGGAAACTAACGTTGGTTCTACGGAAATAAACGTTACTTCCAT---------GAATACTTCGGCAACAAGCAACGTCACATCAAATGTCACAGAGATAAAAGTAACAACAGTCACCATAATTCCAACCGTAATAACAAATACAACTATCAGTAACA---CATCTG---TAAGTTGTGAAATGTTCAA---TA------CGACAATTAATAATACACAAGACCGTG---ATGAATGTAAACCAATAAAAGTAAATAAAACAGATATCAAAGCAGAAGAATGGACCAATGTTACTATACAAAGCAATTTCACAATACCGCACTGTCACAAGGTGGTTTGGATACGACAATATAATCTAACCACACATGGAGATTACTTCCCAACACGATATAAACGTCC----------TTTTGTTAAAGGTGCCTTGTATTCACGT--GAAATATGCGGCCATACATACACACACAATCTTCTACACTCATATGACTTATGCATCTCGT---GCGACAACGGAACACTACATCTCTACGGTGTAAACACAACTCATTCAGGTAGATATACTGCACGTTGTCATATTTATGAACATAATAA---TCACGGCACACATGAGGACAAAAATTTTAATCTGATTATATATCCTAGAAATAATACTAACAATACCAACGGTATCTGGATATGTC-----------CACGAC----------CTACAAAAGACGAAGCCCAAGAAAACAACCAGAGTGAAGAAAAAC---ATCTAACAACGACGGACAACTCTGTTTCCCATAAACGAAACCATTACCCAAGAACCTCACACCGCAGTGCCTGGACTGTTACGTTGCTTTGTGTCGCCTGTATACTCCTATTTTTTTTTCGACGTCTTTTTAACAAAAAATACCGTATGTTGGATGACACCGTCAGCGAATCGGAATTTATTGTACGGTATAACCCAGAGCATGAAGATTGA

PRA8 ATGCATGGCATTTGGAAACCAGGAAGCAACTTAATATACAGA---TGGATATACGTACTTACTATTTGGACTCCTTTGTATAGAAGTAAATGTGAGAACACTGCTAATTC---------------TTCATCCACCGCTGAATCTACAAGCATTATCACTACTACATTGACTACAGATCTTTCATCTACATATCAAATATCATCTGCATCAACTTCAACAC------------CTATTTCTAACACAG------CTACTACAAGCACTCTCATCACTTCTAATCCAAATACA---TCGTATGTTTTCACCTCTTACAATACAT------------------------------------CGACAATTTTAAGCACATCAACGGAAACTAACGTTGGTTCTACGGAAATAAACGTTACTTCCAT---------GAATACTTCGGCAACAAGCAACGTCACATCAAATGTCACAGAGATAAAAGTAACAACAGTCACCATAATTCCAACCGTAATAACAAATACAACTATCAGTAACA---CATCTG---TAAGTTGTGAAATGTTCAA---TA------CGACAATTAATAATACACAAGACCGTG---ATGAATGTAAACCAATAAAAGTAAATAAAACAGATATCAAAGCAGAAGAATGGACCAATGTTACTATACAAAGCAATTTCACAATACCGCACTGTCACAAGGTGGTTTGGATACGACAATATAATCTAACCACACATGGAGATTACTTCCCAACACGATATAAACGTCC----------TTTTGTTAAAGGTGCCTTGTATTCACGT--GAAATATGCGGCCATACATACACACACAATCTTCTACACTCATATGACTTATGCATCTCGT---GCGACAACGGAACACTACATCTCTACGGTGTAAACACAACTCATTCAGGTAGATATACTGCACGTTGTCATATTTATGAACATAATAA---TCACGGCACACATGAGGACAAAAATTTTAATCTGATTATATATCCTAGAAATAATACTAACAATACCAACGGTATCTGGATATGTC-----------CACGAC----------CTACAAAAGACGAAGCCCAAGAAAACAACCAGAGTGAAGAAAAAC---ATCTAACAACGACGGACAACTCTGTTTCCCATAAACGAAACCATTACCCAAGAACCTCACACCGCAGTGCCTGGACTGTTACGTTGCTTTGTGTCGCCTGTATACTCCTATTTTTTTTTCGACGTCTTTTTAACAAAAAATACCGTATGTTGGATGACACCGTCAGCGAATCGGAATTTATTGTACGGTATAACCCAGAGCATGAAGATTGA

BE/15/2011 ATGCATGGCATTTGGAAACCAGGAAGCAACTTAATATACAGA---TGGATATACGTACTTACTATTTGGACTCCTTTGTATAGAAGTAAATGTGAGAACACTGCTAATTC---------------TTCATCCACCGCTGAATCTACAAGCATTATCACTACTACATTGACTACAGATCTTTCATCTACATATCAAATATCATCTGCATCAACTTCAACAC------------CTATTTCTAACACAG------CTACTACAAGCACTCTCATCACTTCTAATCCAAATACA---TCGTATGTTTTCACCTCTTACAATACAT------------------------------------CGACAATTTTAAGCACATCAACGGAAACTAACGTTGGTTCTACGGAAATAAACGTTACTTCCAT---------GAATACTTCGGCAACAAGCAACGTCACATCAAATGTCACAGAGATAAAAGTAACAACAGTCACCATAATTCCAACCGTAATAACAAATACAACTATCAGTAACA---CATCTG---TAAGTTGTGAAATGTTCAA---TA------CGACAATTAATAATACACAAGACCGTG---ATGAATGTAAACCAATAAAAGTAAATAAAACAGATATCAAAGCAGAAGAATGGACCAATGTTACTATACAAAGCAATTTCACAATACCGCACTGTCACAAGGTGGTTTGGATACGACAATATAATCTAACCACACATGGAGATTACTTCCCAACACGATATAAACGTCC----------TTTTGTTAAAGGTGCCTTGTATTCACGT--GAAATATGCGGCCATACATACACACACAATCTTCTACACTCATATGACTTATGCATCTCGT---GTGACAACGGAACACTACATCTCTACGGTGTAAACACAACTCATTCAGGTAGATATACTGCACGTTGTCATATTTATGAACATAATAA---TCACGGCACACATGAGGACAAAAATTTTAATCTGATTATATATCCTAGAAATAATACTAACAATACCAACGGTATCTGGATATGTC-----------CACGAC----------CTACAAAAGACGAAGCCCAAGAAAACAACCAGAGTGAAGAAAAAC---ATCTAACAACGACGGACAACTCTGTTTCCCATAAACGAAACCATTACCCAAGAACCTCACACCGCAGTGCCTGGACTGTTACGTTGCTTTGTGTCGCCTGTATACTCCTATTTTTTTTTCGACGTCTTTTTAACAAAAAATACCGTATGTTGGATGACACCGTCAGCGAATCGGAATTTATTGTACGGTATAACCCAGAGCATGAAGATTGA

BE/4/2011 ATGCATGGCATTTGGAAACCAGGAAGCAACTTAATATACAGA---TGGATATACGTACTTACTATTTGGACTCCTTTGTATAGAAGTAAATGTGAGAACACTGCTAATTC---------------TTCATCCACCGCTGAATCTACAAGCATTATCACTACTACATTGACTACAGATCTTTCATCTACATATCAAATATCATCTGCATCAACTTCAACAC------------CTATTTCTAACACAG------CTACTACAAGCACTCTCATCACTTCTAATCCAAATACA---TCGTATGTTTTCACCTCTTACAATACAT------------------------------------CGACAATTTTAAGCACATCAACGGAAACTAACGTTGGTTCTACGGAAATAAACGTTACTTCCAT---------GAATACTTCGGCAACAAGCAACGTCACATCAAATGTCACAGAGATAAAAGTAACAACAGTCACCATAATTCCAACCGTAATAACAAATACAACTATCAGTAACA---CATCTG---TAAGTTGTGAAATGTTCAA---TA------CGACAATTAATAATACACAAGACCGTG---ATGAATGTAAACCAATAAAAGTAAATAAAACAGATATCAAAGCAGAAGAATGGACCAATGTTACTATACAAAGCAATTTCACAATACCGCACTGTCACAAGGTGGTTTGGATACGACAATATAATCTAACCACACATGGAGATTACTTCCCAACACGATATAAACGTCC----------TTTTGTTAAAGGTGCCTTGTATTCACGT--GAAATATGCGGCCATACATACACACACAATCTTCTACACTCATATGACTTATGCATCTCGT---GTGACAACGGAACACTACATCTCTACGGTGTAAACACAACTCATTCAGGTAGATATACTGCACGTTGTCATATTTATGAACATAATAA---TCACGGCACACATGAGGACAAAAATTTTAATCTGATTATATATCCTAGAAATAATACTAACAATACCAACGGTATCTGGATATGTC-----------CACGAC----------CTACAAAAGACGAAGCCCAAGAAAACAACCAGAGTGAAGAAAAAC---ATCTAACAACGACGGACAACTCTGTTTCCCATAAACGAAACCATTACCCAAGAACCTCACACCGCAGTGCCTGGACTGTTACGTTGCTTTGTGTCGCCTGTATACTCCTATTTTTTTTTCGACGTCTTTTTAACAAAAAATACCGTATGTTGGATGACACCGTCAGCGAATCGGAATTTATTGTACGGTATAACCCAGAGCATGAAGATTGA

PRA6 ATGCATGGCATTTGGAAACCAGGAAGCAACTTAATATACAGA---TGGATATACGTACTTACTATTTGGACTCCTTTGTATAGAAGTAAATGTGAGAACACTGCTAATTC---------------TTCATCCACCGCTGAATCTACAAGCATTATCACTACTACATTGACTACAGATCTTTCATCTACATATCAAATATCATCTGCATCAACTTCAACAC------------CTATTTCTAACACAG------CTACTACAAGCACTCTCATCACTTCTAATCCAAATACA---TCGTATGTTTTCACCTCTTACAATACAT------------------------------------CGACAATTTTAAGCACATCAACGGAAACTAACGTTGGTTCTACGGAAATAAACGTTACTTCCAT---------GAATACTTCGGCAACAAGCAACGTCACATCAAATGTCACAGAGATAAAAGTAACAACAGTCACCATAATTCCAACCGTAATAACAAATACAACTATCAGTAACA---CATCTG---TAAGTTGTGAAATGTTCAA---TA------CGACAATTAATAATACACAAGACCGTG---ATGAATGTAAACCAATAAAAGTAAATAAAACAGATATCAAAGCAGAAGAATGGACCAATGTTACTATACAAAGCAATTTCACAATACCGCACTGTCACAAGGTGGTTTGGATACGACAATATAATCTAACCACACATGGAGATTACTTCCCAACACGATATAAACGTCC----------TTTTGTTAAAGGTGCCTTGTATTCACGT--GAAATATGCGGCCATACATACACACACAATCTTCTACACTCATATGACTTATGCATCTCGT---GTGACAACGGAACACTACATCTCTACGGTGTAAACACAACTCATTCAGGTAGATATACTGCACGTTGTCATATTTATGAACATAATAA---TCACGGCACACATGAGGACAAAAATTTTAATCTGATTATATATCCTAGAAATAATACTAACAATACCAACGGTATCTGGATATGTC-----------CACGAC----------CTACAAAAGACGAAGCCCAAGAAAACAACCAGAGTGAAGAAAAAC---ATCTAACAACGACGGACAACTCTGTTTCCCATAAACGAAACCATTACCCAAGAACCTCACACCGCAGTGCCTGGACTGTTACGTTGCTTTGTGTCGCCTGTATACTCCTATTTTTTTTTCGACGTCTTTTTAACAAAAAATACCGTATGTTGGATGACACCGTCAGCGAATCGGAATTTATTGTACGGTATAACCCAGAGCATGAAGATTGA

HANSCTR2 ATGCATGGCATTTGGAAACCAGGAAGCAACTTAATATACAGA---TGGATATACGTACTTACTATTTGGACTCCTTTGTATAGAAGTAAATGTGAGAACACTGCTAATTC---------------TTCATCCACCGCTGAATCTACAAGCATTATCACTACTACATTGACTACAGATCTTTCATCTACATATCAAATATCATCTGCATCAACTTCAACAC------------CTATTTCTAACACAG------CTACTACAAGCACTCTCATCACTTCTAATCCAAATACA---TCGTATGTTTTCACCTCTTACAATACAT------------------------------------CGACAATTTTAAGCACATCAACGGAAACTAACGTTGGTTCTACGGAAATAAACGTTACTTCCAT---------GAATACTTCGGCAACAAGCAACGTCACATCAAATGTCACAGAGATAAAAGTAACAACAGTCACCATAATTCCAACCGTAATAACAAATACAACTATCAGTAACA---CATCTG---TAAGTTGTGAAATGTTCAA---TA------CGACAATTAATAATACACAAGACCGTG---ATGAATGTAAACCAATAAAAGTAAATAAAACAGATATCAAAGCAGAAGAATGGACCAATGTTACTATACAAAGCAATTTCACAATACCGCACTGTCACAAGGTGGTTTGGATACGACAATATAATCTAACCACACATGGAGATTACTTCCCAACACGATATAAACGTCC----------TTTTGTTAAAGGTGCCTTGTATTCACGT--GAAATATGCGGCCATACATACACACACAATCTTCTACACTCATATGACTTATGCATCTCGT---GTGACAACGGAACACTACATCTCTACGGTGTAAACACAACTCATTCAGGTAGATATACTGCACGTTGTCATATTTATGAACATAATAA---TCACGGCACACATGAGGACAAAAATTTTAATCTGATTATATATCCTAGAAATAATACTAACAATACCAACGGTATCTGGATATGTC-----------CACGAC----------CTACAAAAGACGAAGCCCAAGAAAACAACCAGAGTGAAGAAAAAC---ATCTAACAACGACGGACAACTCTGTTTCCCATAAACGAAACCATTACCCAAGAACCTCACACCGCAGTGCCTGGACTGTTACGTTGCTTTGTGTCGCCTGTATACTCCTATTTTTTTTTCGACGTCTTTTTAACAAAAAATACCGTATGTTGGATGACACCGTCAGCGAATCGGAATTTATTGTACGGTATAACCCAGAGCATGAAGATTGA

Pat_F ATGCGTGGCATTTGGAAGCCAGGAAGCAACTTAATATACAGA---TGGATATACGTACTTACTATTTGGACTCCTTTGTATAGAAGTAAATGTGAGAACACTACTAATTC---------------TTCATCCACCGCTGACTCTACAAGCATTATCACTACTACATTGACTACAGATCTTTCATCTACATATCAAATATCATCTGCATCAACTTCAACAC------------CTATTTCTAACACAG------CTACTACAAGCACTCTCATCACTTCTAATCCAAATACA---TCGTATGTTTTCACCTCTTACAATACAT------------------------------------CGACAATTTTAAGCACATCAACGGAAACTAACGTTGGTTCTACGGAAATAAACGTTACTTCCAT---------GAATACTTCGGCAACAAGCAACGTCACATCAAATGTCACAGAGATAAAAGTAACAACAGTCACCATAACTCCAACCGTAATAACAAATACAACTATCAGTAACA---CATCTG---TAAGTTGTGAAATGTTCAA---TA------CGACAATTAATAATACACAAGACCGTG---ATGAATGCAAACCAATAAAAGTAAATAAAACAGATATCAAAGCAGAAGAATGGACCAATGTTACTATACAAAGCAATTTCACAATATCGCACTGTCACAAGGTGGTTTGGATACGACAATATAATCTAACCACACATGGAGATTACTTCCCAACACGATATAAACGTCC----------TTTTGTTAAAGGTGCCTTGTATTCACGT--GAAATATGTGGCCATACATACACACACAATCTTCTACACTCATATGACTTATGCATCTCGT---GCGACAACGGAACACTACATCTCTACGGTGTAAACACAACTCATTCAGGTAGATATACTGCACGTTGTCATATTTATGAACATAATGA---TCACGGCACACATGAGGACAAAAATTTTAATCTGATTATATATCCTAGAAATAATACTAACAATACCAACGGTATCTGGATATGTC-----------CACGAC----------CTACAAAAGACGAAGCCCAAGAAAACAACCAGAGTGAAGAAAAAC---ATCTAACAACGACGGACAACTCTGTTTCCCATAAACGAAACCATTACCCAAGAACCTCACACCGCAGTGCCTGGACTGTTACGTTGCTTTGTGTCGCCTGTATACTCCTATTTTTTTTTCGACGTCTTTTTAACAAAAAATACCGTATGTTGGATGACACCGTCAGCGAATCGGAATTTATTGTACGGTATAACCCAGAGCATGAAGATTGA

JER4755 ATGCGTGGCATTTGGAAGCCAGGAAGCAACTTAATATACAGA---TGGATATACGTACTTACTATTTGGACTCCTTTGTATAGAAGTAAATGTGAGAACACTACTAATTC---------------TTCATCCACCGCTGAATCTACAAGCATTATCACTACTACATTAACTACAGATCTTTCATCTACATATCAAATATCATCTGCATCAACTTCAACAC------------CTATTTCTAACACAG------CTACTACAAGCACTCTCATCACTTCTAATCCAAATACA---TCGTATGTTTTCACCTCTTACAATACAT------------------------------------CGACAATTTTAAGCACATCAACGGAAACTAACGTTGGTTCTACGGAAATAAACGTTACTTCCAT---------GAATACTTCGGCAACAAGCAACGTCACATCAAATGTCACAGAGATAAAAGTAACAACAGTCACCATAATTCCAACCGTAATAACAAATACAACTATCAGTAACA---CATCTG---TAAGTTGTGAAATGTTCAA---TA------CGACAATTAATAATACACAAGACCGTG---ATGAATGCAAACCAATAAAAGTAAATAAAACAGATATCAAAGCAGAAGAATGGACCAATGTTACTATACAAAGCAATTTCACAATACCGCACTGTCACAAGGTGGTTTGGATACGACAATATAATCTAACCACACATGGAGATTACTTCCCAACACGATATAAACGTCC----------TTTTGTTAAAGGTGCCTTGTATTCACGT--GAAATATGCGGCCATACATACACATACAATCTTCTACACTCATATGACTTATGCATCTCGT---GCGACAACGGAACACTACATCTCTACGGTGTAAACACAACTCATTCAGGTAGATATACTGCACGTTGTCATATTTATGAACATAATAA---TCACGGCACACATGAGGACAAAAATTTTAATCTGATTATATATCCTAGAAATAATACTAACAATACCAACGGTATCTGGATATGTC-----------CACGAC----------CTACAAAAGACGAAGCCCAAGAAAACAACCAGAGTGAAGAAAAAC---ATCTAACAACGACGGACAACTCTGTTTCCCATAAACGAAACCATTACCCAAGAACCTCACACCGCAGTGCCTGGACTGTTACGTTGCTTTGTGTCGCCTGTATACTCCTATTTTTTTTTCGACGTCTTTTTAACAAAAAATACCGTATGTTGGATGACACCGTCAGCGAATCGGAATTTATTGTACGCTATAACCCAGAGCATGAAGATTGA

U4 ATGCGTGGCATTTGGAAGCCAGGAAGCAACTTAATATACAGA---TGGATATACGTACTTACTATTTGGACTCCTTTGTATAGAAGTAAATGTGAGAACACTACTAATTC---------------TTCATCCACCGCTGAATCTACAAGCATTATCACTACTACATTAACTACAGATCTTTCATCTACATATCAAATATCATCTGCATCAACTTCAACAC------------CTATTTCTAACACAG------CTACTACAAGCACTCTCATCACTTCTAATCCAAATACA---TCGTATGTTTTCACCTCTTACAATACAT------------------------------------CGACAATTTTAAGCACATCAACGGAAACTAACGTTGGTTCTACGGAAATAAACGTTACTTCCAT---------GAATACTTCGGCAACAAGCAACGTCACATCAAATGTCACAGAGATAAAAGTAACAACAGTCACCATAATTCCAACCGTAATAACAAATACAACTATCAGTAACA---CATCTG---TAAGTTGTGAAATGTTCAA---TA------CGACAATTAATAATACACAAGACCGTG---ATGAATGCAAACCAATAAAAGTAAATAAAACAGATATCAAAGCAGAAGAATGGACCAATGTTACTATACAAAGCAATTTCACAATACCGCACTGTCACAAGGTGGTTTGGATACGACAATATAATCTAACCACACATGGAGATTACTTCCCAACACGATATAAACGTCC----------TTTTGTTAAAGGTGCCTTGTATTCACGT--GAAATATGCGGCCATACATACACATACAATCTTCTACACTCATATGACTTATGCATCTCGT---GCGACAACGGAACACTACATCTCTACGGTGTAAACACAACTCATTCAGGTAGATATACTGCACGTTGTCATATTTATGAACATAATAA---TCACGGCACACATGAGGACAAAAATTTTAATCTGATTATATATCCTAGAAATAATACTAACAATACCAACGGTATCTGGATATGTC-----------CACGAC----------CTACAAAAGACGAAGCCCAAGAAAACAACCAGAGTGAAGAAAAAC---ATCTAACAACGACGGACAACTCTGTTTCCCATAAACGAAACCATTACCCAAGAACCTCACACCGCAGTGCCTGGACTGTTACGTTGCTTTGTGTCGCCTGTATACTCCTATTTTTTTTTCGACGTCTTTTTAACAAAAAATACCGTATGTTGGATGACACCGTCAGCGAATCGGAATTTATTGTACGCTATAACCCAGAGCATGAAGATTGA

HANChild2&3 ATGCGTGGCATTTGGAAGCCAGGAAGCAACTTAATATACAGA---TGGATATACGTACTTACTATTTGGACTCCTTTGTATAGAAGTAAATGTGAGAACACTACTAATTC---------------TTCATCCACCGCTGAATCTACAAGCATTATCACTACTACATTGACTACAGATCTTTCATCTACATATCAAATATCATCTGCATCAACTTCAACAC------------CTATTTCTAACACAG------CTACTACAAGCACTCTCATCACTTCTAATCCAAATACA---TCGTATGTTTTCACCTCTTACAATACAT------------------------------------CGACAATTTTAAGCACATCAACGGAAACTAACGTTGGTTCTACGGAAATAAACGTTACTTCCAT---------GAATACTTCGGCAACAAGCAACGTCACATCGAATGTCACAGAGATAAAAGTAACAACAGTCACCATAATTCCAACCGTAATAACAAATACAACTATCAGTAACA---CATCTG---TAAGTTGTGAAATGTTCAA---TA------CGACAATTAATAATACACAAGACCGTG---ATGAATGCAAACCAATAAAAGTAAATAAAACAGATATCAAAGCAGAAGAATGGACCAATGTTACTATACAAAGCAATTTCACAATACCGCACTGTCACAAGGTGGTTTGGATACGACAATATAATCTAACCACACATGGAGATTACTTCCCAACACGATATAAACGTCC----------TTTTGTTAAAGGTGCCTTGTATTCACGT--GAAATATGCGGCCATACATACACACACAATCTTCTACACTCATATGACTTATGCATCTCGT---GCGACAACGGAACACTACATCTCTACGGTGTAAACACAACTCATTCAGGTAGATATACTGCACGTTGTCATATTTATGAACATAATAA---TCACGGCACACATGAGGACAAAAATTTTAATCTGATTATATATCCTAGAAATAATACTAACAATACCAACGGTATCTGGATATGTC-----------CACGAC----------CTACAAAAGACGAAGCCCAAGAAAACAACCAGAGTGAAGAAAAAC---ATCTAACAACGACGGACAACTCTGTTTCCCATAAACGAAACCATTACCCAAGAACCTCACACCGCAGTGCCTGGACTGTTACGTTGCTTTGTGTCGCCTGTATACTCCTATTTTTTTTTCGACGTCTTTTTAACAAAAAATACCGTATGTTGGATGACACCGTCAGCGAATCGGAATTTATTGTACGCTATAACCCAGAGCATGAAGATTGA

BE/5/2010 ATGCGTGGCATTTGGAAGCCAGGAAGCAACTTAATATACAGA---TGGATATACGTACTTACTATTTGGACTCCTTTGTATAGAAGTAAATGTGAGAACACTACTAATTC---------------TTCATCCACCGCTGAATCTACAAGCATTATCACTACTACATTGACTACAGATCTTTCATCTACATATCAAATATCATCTGCATCAACTTCAACAC------------CTATTTCTAACACAG------CTACTACAAGCACTCTCATCACTTCTAATCCAAATACA---TCGTATGTTTTCACCTCTTACAATACAT------------------------------------CGACAATTTTAAGCACATCAACGGAAACTAACGTTGGTTCTACGGAAATAAACGTTACTTCCAT---------GAATACTTCGGCAACAAGCAACGTCACATCAAATGTCACAGAGATAAAAGTAACAACAGTCACCATAATTCCAACCGTAATAACAAATACAACTATCAGTAACA---CATCTG---TAAGTTGTGAAATGTTCAA---TA------CGACAATTAATAATACACAAGACCGTG---ATGAATGCAAACCAATAAAAGTAAATAAAACAGATATCAAAGCAGAAGAATGGACCAATGTTACTATACAAAGCAATTTCACAATACCGCACTGTCACAAGGTGGTTTGGATACGACAATATAATCTAACCACACATGGAGATTACTTCCCAACACGATATAAACGTCC----------TTTTGTTAAAGGTGCCTTGTATTCACGT--GAAATATGCGGCCATACATACACACACAATCTTCTACACTCATATGACTTATGCATCTCGT---GCGACAACGGAACACTACATCTCTACGGTGTAAACACAACTCATTCAGGTAGATATACTGCACGTTGTCATATTTATGAACATAATAA---TCACGGCACACATGAGGACAAAAATTTTAATCTGATTATATATCCTAGAAATAATACTAACAATACCAACGGTATCTGGATATGTC-----------CACGAC----------CTACAAAAGACGAAGCCCAAGAAAACAACCAGAGTGAAGAAAAAC---ATCTAACAACGACGGACAACTCTGTTTCCCATAAACGAAACCATTACCCAAGAACCTCACACCGCAGTGCCTGGACTGTTACGTTGCTTTGTGTCGCCTGTATACTCCTATTTTTTTTTCGACGTCTTTTTAACAAAAAATACCGTATGTTGGATGACACCGTCAGCGAATCGGAATTTATTGTACGCTATAACCCAGAGCATGAAGATTGA

HAN16 ATGCGTGGCATTTGGAAGCCAGGAAGCAACTTAATATACAGA---TGGATATACGTACTTACTATTTGGACTCCTTTGTATAGAAGTAAATGTGAGAACACTACTAATTC---------------TTCATCCACCGCTGAATCTACAAGCATTATCACTACTACATTGACTACAGATCTTTCATCTACATATCAAATATCATCTGCATCAACTTCAACAC------------CTATTTCTAACACAG------CTACTACAAGCACTCTCATCACTTCTAATCCAAATACA---TCGTATGTTTTCACCTCTTACAATACAT------------------------------------CGACAATTTTAAGCACATCAACGGAAACTAACGTTGGTTCTACGGAAATAAACGTTACTTCCAT---------GAATACTTCGGCAACAAGCAACGTCACATCAAATGTCACAGAGATAAAAGTAACAACAGTCACCATAATTCCAACCGTAATAACAAATACAACTATCAGTAACA---CATCTG---TAAGTTGTGAAATGTTCAA---TA------CGACAATTAATAATACACAAGACCGTG---ATGAATGCAAACCAATAAAAGTAAATAAAACAGATATCAAAGCAGAAGAATGGACCAATGTTACTATACAAAGCAATTTCACAATACCGCACTGTCACAAGGTGGTTTGGATACGACAATATAATCTAACCACACATGGAGATTACTTCCCAACACGATATAAACGTCC----------TTTTGTTAAAGGTGCCTTGTATTCACGT--GAAATATGCGGCCATACATACACACACAATCTTCTACACTCATATGACTTATGCATCTCGT---GCGACAACGGAACACTACATCTCTACGGTGTAAACACAACTCATTCAGGTAGATATACTGCACGTTGTCATATTTATGAACATAATAA---TCACGGCACACATGAGGACAAAAATTTTAATCTGATTATATATCCTAGAAATAATACTAACAATACCAACGGTATCTGGATATGTC-----------CACGAC----------CTACAAAAGACGAAGCCCAAGAAAACAACCAGAGTGAAGAAAAAC---ATCTAACAACGACGGACAACTCTGTTTCCCATAAACGAAACCATTACCCAAGAACCTCACACCGCAGTGCCTGGACTGTTACGTTGCTTTGTGTCGCCTGTATACTCCTATTTTTTTTTCGACGTCTTTTTAACAAAAAATACCGTATGTTGGATGACACCGTCAGCGAATCGGAATTTATTGTACGCTATAACCCAGAGCATGAAGATTGA

HANSCTR11A ATGCGTGGCATTTGGAAGCCAGGAAGCAACTTAATATACAGA---TGGATATACGTACTTACTATTTGGACTCCTTTGTATAGAAGTAAATGTGAGAACACTACTAATTC---------------TTCATCCACCGCTGAATCTACAAGCATTATCACTACTACATTGACTACAGATCTTTCATCTACATATCAAATATCATCTGCATCAACTTCAACAC------------CTATTTCTAACACAG------CTACTACAAGCACTCTCATCACTTCTAATCCAAATACA---TCGTATGTTTTCACCTCTTACAATACAT------------------------------------CGACAATTTTAAGCACATCAACGGAAACTAACGTTGGTTCTACGGAAATAAACGTTACTTCCAT---------GAATACTTCGGCAACAAGCAACGTCACATCAAATGTCACAGAGATAAAAGTAACAACAGTCACCATAATTCCAACCGTAATAACAAATACAACTATCAGTAACA---CATCTG---TAAGTTGTGAAATGTTCAA---TA------CGACAATTAATAATACACAAGACCGTG---ATGAATGCAAACCAATAAAAGTAAATAAAACAGATATCAAAGCAGAAGAATGGACCAATGTTACTATACAAAGCAATTTCACAATACCGCACTGTCACAAGGTGGTTTGGATACGACAATATAATCTAACCACACATGGAGATTACTTCCCAACACGATATAAACGTCC----------TTTTGTTAAAGGTGCCTTGTATTCACGT--GAAATATGCGGCCATACATACACACACAATCTTCTACACTCATATGACTTATGCATCTCGT---GCGACAACGGAACACTACATCTCTACGGTGTAAACACAACTCATTCAGGTAGATATACTGCACGTTGTCATATTTATGAACATAATAA---TCACGGCACACATGAGGACAAAAATTTTAATCTGATTATATATCCTAGAAATAATACTAACAATACCAACGGTATCTGGATATGTC-----------CACGAC----------CTACAAAAGACGAAGCCCAAGAAAACAACCAGAGTGAAGAAAAAC---ATCTAACAACGACGGACAACTCTGTTTCCCATAAACGAAACCATTACCCAAGAACCTCACACCGCAGTGCCTGGACTGTTACGTTGCTTTGTGTCGCCTGTATACTCCTATTTTTTTTTCGACGTCTTTTTAACAAAAAATACCGTATGTTGGATGACACCGTCAGCGAATCGGAATTTATTGTACGCTATAACCCAGAGCATGAAGATTGA

PAV20 ATGCGTGGCATTTGGAAGCCAGGAAGCAACTTAATATACAGA---TGGATATACGTACTTACTATTTGGACTCCTTTGTATAGAAGTAAATGTGAGAACACTACTAATTC---------------TTCATCCACCGCTGAATCTACAAGCATTATCACTACTACATTGACTACAGATCTTTCATCTACATATCAAATATCATCTGCATCAACTTCAACAC------------CTATTTCTAACACAG------CTACTACAAGCACTCTCATCACTTCTAATCCAAATACA---TCGTATGTTTTCACCTCTTACAATACAT------------------------------------CGACAATTTTAAGCACATCAACGGAAACTAACGTTGGTTCTACGGAAATAAACGTTACTTCCAT---------GAATACTTCGGCAACAAGCAACGTCACATCAAATGTCACAGAGATAAAAGTAACAACAGTCACCATAATTCCAACCGTAATAACAAATACAACTATCAGTAACA---CATCTG---TAAGTTGTGAAATGTTCAA---TA------CGACAATTAATAATACACAAGACCGTG---ATGAATGCAAACCAATAAAAGTAAATAAAACAGATATCAAAGCAGAAGAATGGACCAATGTTACTATACAAAGCAATTTCACAATACCGCACTGTCACAAGGTGGTTTGGATACGACAATATAATCTAACCACACATGGAGATTACTTCCCAACACGATATAAACGTCC----------TTTTGTTAAAGGTGCCTTGTATTCACGT--GAAATATGCGGCCATACATACACACACAATCTTCTACACTCATATGACTTATGCATCTCGT---GCGACAACGGAACACTACATCTCTACGGTGTAAACACAACTCATTCAGGTAGATATACTGCACGTTGTCATATTTATGAACATAATAA---TCACGGCACACATGAGGACAAAAATTTTAATTTGATTATATATCCTAGAAATAATACTAACAATACCAACGGTATCTGGATATGTC-----------CACGAC----------CTACAAAAGACGAAGCCCAAGAAAACAACCAGAGTGAAGAAAAAC---ATCTAACAACGACGGACAACTCTGTTTCCCATAAACGAAACCATTACCCAAGAACCTCACACCGCAGTGCCTGGACTGTTACGTTGCTTTGTGTCGCCTGTATACTCCTATTTTTTTTTCGACGTCTTTTTAACAAAAAATACCGTATGTTGGATGACACCGTCAGCGAATCGGAATTTATTGTACGCTATAACCCAGAGCATGAAGATTGA

HAN27 ATGCGTGGCATTTGGAAGCCAGGAAGTAACCTAATATACAGA---TTTATATGCGTACTTACTATCTGGACTCCTTTGTACAGAAGTAAATGTGAGAACACTACTAATGC---------------TTCATCCACCGCTGAATCTACAAGCATTATCACTACTACATTGACTACAGATCTTTCATCTACATATCAAATATCATCTGCATCAACTTCAACAC------------CTATTTCTAACACAG------CTACCATAAGCACTTTCATCACTTCCAATCCAAATACA---TCGTATGTTTCCACCTCTTACAATACAT------------------------------------CGATAATTTTAAGCACATCAACGGAAACTAACGTTGGCTCTACGGAAATAAACGTTACTTCCAT---------GAATACTTCAGCAACAAGCAACGTCACGTCAAATGT------------------------CACCATAACTCCAACCGTAGTAACAAGTACAACTATCAGTAACATATCATCTG---TAAGTTATGAAATGTTCAA---TA------CGACAATTAATAATACACAAGACTGTA---ATGAATGTCAACCAATAAAAGTAAATAAAACAGATATCGAAGCAGAAGAATGGACCAATGTTACCATACAAAGCAATTTCACGATACCCCATTGTCACAAGGTGGTTTGGATGCGACAATATAATCTAACCACACATGGAGATTATT--------------------------------------------------------------GAAATATGCGGCCATACATACACACACAATCTTCTACACTCATATGACTTATGCATCTCGT---GCGACAACGGAACACTACATCTCTACGGTGTAAACACAACTCATTCAGGTAGATATACTGCACGTTGTCATATCTATGAACATAATGA---TCACGGCACACATGAGGACAAAAATTTTAATCTGATTATATATCCTAGAAATAATACTAACAATACCAACGGTATCTGGATATGTC-----------CACGAC----------CTACAAAAGACGAAGCCCAAGAAAACAACCAGAGTGAAGAAAAGC---ATCTAACAACGACGGACAATTCTGTTTCCCATAAACGAAACCATTACCCAAGAACTTCCCACCGCAGTGCCTGGACTGTTACGTTGCTTTGTGTCGCCTGTATACTCCTATTTTTTTTTCGACGTCTTTTTAATAAAAAATACCGTATGTTGGATGACACCGTTAGCGAATCGGAATTTATTGTACGGTATAACCCAGAGCATGAAGATTGA

UK/Lon5 ATGCGTGGCATTTGGAAGCCAGGAAGTAACCTAATATACAGA---TTTATATGCGTACTTACTATCTGGACTCCTTTGTACAGAAGTAAATGTGAGAACACTACTAATGC---------------TTCATCCACCGCTGAATCTACAAGCATTATCACTACTACATTGACTACAGATCTTTCATCTACATATCAAATATCATCTGCATCAACTTCAACAC------------CTATTTCTAACACAG------CTACCATAAGCACTTTCATCACTTCCAATCCAAATACA---TCGTATGTTTCCACCTCTTACAATACAT------------------------------------CGATAATTTTAAGCACATCAACGGAAACTAACGTTGGCTCTACGGAAATAAACGTTACTTCCAT---------GAATACTTCAGCAACAAGCAACGTCACGTCAAATGT------------------------CACCATAACTCCAACCGTAGTAACAAGTACAACTATCAGTAACATATCATCTG---TAAGTTATGAAATGTTCAA---TA------CGACAATTAATAATACACAAGACTGTG---ATGAATGTCAACCAATAAAAGTAAATAAAACAGATATCGAAGCAGAAGAATGGACCAATGTTACCATACAAAGCAATTTCACAATACCCCATTGTCACAAGGTGGTTTGGATGCGACAATATAATCTAACCACACATGGAGATTATTTCCCAATACGATATAAACGTCC-------------TGTTAAAGGTGCCTTGTATTCACGT--GAAATATGCGGCCATACATACACACACAATCTTCTACACTCATATGACTTATGCATCTCGT---GCGACAACGGAACACTACATCTCTACGGTGTAAACACAACTCATTCAGGTAGATATACTGCACGTTGTCATATCTATGAACATAATGA---TCACGGCACACATGAGGACAAAAATTTTAATCTGATTATATATCCTAGAAATAATACTAACAATCCCAACGGTATCTGGATATGTC-----------CACGAC----------CTACAAAAGACGAAGCCCAAGAAAACAACCAGAGTGAAGAAAAGC---ATCTAACAACGACGGACAATTCTGTTTCCCATAAACGAAACCATTACCCAAGAACTTCCCACCGCAGTGCCTGGACTGTTACGTTGCTTTGTGTCGCCTGTATACTCCTATTTTTTTTTCGACGTCTTTTTAATAAAAAATATCGTATGTTGGATGACACCGTTAGCGAATCGGAATTTATTGTACGGTATAACCCAGAGCATGAAGATTGA

HANSCTR4 ATGCGTGGCATTTGGAAGCCAGGAAGTAACCTAATATACAGA---TTTATATGCGTACTTACTATCTGGACTCCTTTGTACAGAAGTAAATGTGAGAACACTACTAATGC---------------TTCATCCACCGCTGAATCTACAAGCATTATCACTACTACATTGACTACAGATCTTTCATCTACATATCAAATATCATCTGCATCAACTTCAACAC------------CTATTTCTAACACAG------CTACCATAAGCACTTTCATCACTTCCAATCCAAATACA---TCGTATGTTTCCACCTCTTACAATACAT------------------------------------CGATAATTTTAAGCACATCAACGGAAACTAACGTTGGCTCTACGGAAATAAACGTTACTTCCAT---------GAATACTTCAGCAACAAGCAACGTCACATCAAATGT------------------------CACCATAACTCCAACCGTAGTAACAAGTACAACTATCAGTAACATATCATCTG---TAAGTTATGAAATGTTCAA---TA------CGACAATTAATAATACACAAGACTGTG---ATGAATGTCAACCAATAAAAGTAAATAAAACAGATATCGAAGCAGAAGAATGGACCAATGTTACCATACAAAGCAATTTCACGATACCCCATTGTCACAAGGTGGTTTGGATGCGACAATATAATCTAACCACACATGGAGATTATTTCCCAATACGATATAAACGTCC-------------TGTTAAAGGTGCCTTGTATTCACGT--GAAATATGCGGCCATACATACACACACAATCTTCTACACTCATATGACTTATGCATCTCGT---GCGACAACGGAACACTACATCTCTACGGTGTAAACACAACTCATTCAGGTAGATATACTGCACGTTGTCATATCTATGAACATAATGA---TCACGGCACACATGAGGACAAAAATTTTAATCTGATTATATATCCTAGAAATAATACTAACAATACCAACGGTATCTGGATATGTC-----------CACGAC----------CTACAAAAGACGAAGCCCAAGAAAACAACCAGAGTGAAGAAAAGC---ATCTAACAACGACGGACAATTCTGTTTCCCATAAACGAAACCATTACCCAAGAACTTCCCACCGCAGTGCCTGGACTGTTACGTTGCTTTGTGTCGCCTGTATACTCCTATTTTTTTTTCGACGTCTTTTTAATAAAAAATACCGTATGTTGGATGACACCGTTAGCGAATCGGAATTTATTGTACGGTATAACCCAGAGCATGAAGATTGA

PAV5 ATGCGTGGCATTTGGAAGCCAGGAAGTAACCTAATATACAGA---TTTATATGCGTACTTACTATCTGGACTCCTTTGTACAGAAGTAAATGTGAGAACACTACTAATGC---------------TTCATCCACCGCTGAATCTACAAGCATTATCACTACTACATTGACTACAGATCTTTCATCTACATATCAAATATCATCTGCATCAACTTCAACAC------------CTATTTCTAACACAG------CTACCATAAGCACTTTCATCACTTCCAATCCAAATACA---TCGTATGTTTCCACCTCTTACAATACAT------------------------------------CGATAATTTTAAGCACATCAACGGAAACTAACGTTGGCTCTACGGAAATAAACGTTACTTCCAT---------GAATACTTCAGCAACAAGCAACGTCACGTCAAATGT------------------------CACCATAACTCCAACCGTAGTAACAAGTACAACTATCAGTAACATATCATCTG---TAAGTTATGAAATGTTCAA---TA------CGACAATTAATAATACACAAGACTGTA---ATGAATGTCAACCAATAAAAGTAAATAAAACAGATATCGAAGCAGAAGAATGGACCAATGTTACCATACAAAGCAATTTCACGATACCCCATTGTCACAAGGTGGTTTGGATGCGACAATATAATCTAACCACACATGGAGATTATTTCCCAATACGATATAAACGTCC-------------TGTTAAAGGTGCCTTGTATTCACGT--GAAATATGCGGCCATACATACACACACAATCTTCTACACTCATATGACTTATGCATCTCGT---GCGACAACGGAACACTACATCTCTACGGTGTAAACACAACTCATTCAGGTAGATATACTGCACGTTGTCATATCTATGAACATAATGA---TCACGGCACACATGAGGACAAAAATTTTAATCTGATTATATATCCTAGAAATAATACTAACAATACCAACGGTATCTGGATATGTC-----------CACGAC----------CTACAAAAGACGAAGCCCAAGAAAACAACCAGAGTGAAGAAAAGC---ATCTAACAACGACGGACAATTCTGTTTCCCATAAACGAAACCATTACCCAAGAACTTCCCACCGCAGTGCCTGGACTGTTACGTTGCTTTGTGTCGCCTGTATACTCCTATTTTTTTTTCGACGTCTTTTTAATAAAAAATACCGTATGTTGGATGACACCGTTAGCGAATCGGAATTTATTGTACGGTATAACCCAGAGCATGAAGATTGA

W ATGCGTGGCATTTGGAAGCCAGGAAGTAACCTAATATACAGA---TTTATATGCGTACTTACTATCTGGACTCCTTTGTACAGAAGTAAATGTGAGAACACTACTAATGC---------------TTCATCCACCGCTGAATCTACAAGCATTATCACTACTACATTGACTACAGATCTTTCATCTACATATCAAATATCATCTGCATCAACTTCAACAC------------CTATTTCTAACACAG------CTACCATAAGCACTTTCATCACTTCCAATCCAAATACA---TCGTATGTTTCCACCTCTTACAATACAT------------------------------------CGATAATTTTAAGCACATCAACGGAAACTAACGTTGGCTCTACGGAAATAAACGTTACTTCCAT---------GAATACTTCAGCAACAAGCAACGTCACGTCAAATGT------------------------CACCATAACTCCAACCGTAGTAACAAGTACAACTATCAGTAACATATCATCTG---TAAGTTATGAAATGTTCAA---TA------CGACAATTAATAATACACAAGACTGTG---ATGAATGTCAACCAATAAAAGTAAATAAAACAGATATCGAAGCAGAAGAATGGACCAATGTTACCATACAAAGCAATTTCACGATACCCCATTGTCACAAGGTGGTTTGGATGCGACAATATAATCTAACCACACATGGAGATTATTTCCCAATACGATATAAACGTCC-------------TGTTAAAGGTGCCTTGTATTCACGT--GAAATATGCGGCCATACATACACACACAATCTTCTACACTCATATGACTTATGCATCTCGT---GCGACAACGGAACACTACATCTCTACGGTGTAAACACAACTCATTCAGGTAGATATACTGCACGTTGTCATATCTATGAACATAATGA---TCACGGCACACATGAGGACAAAAATTTTAATCTGATTATATATCCTAGAAATAATACTAACAATACCAACGGTATCTGGATATGTC-----------CACGAC----------CTACAAAAGACGAAGCCCAAGAAAACAACCAGAGTGAAGAAAAGC---ATCTAACAACGACGGACAATTCTGTTTCCCATAAACGAAACCATTACCCAAGAACTTCCCACCGCAGTGCCTGGACTGTTACGTTGCTTTGTGTCGCCTGTATACTCCTATTTTTTTTTCGACGTCTTTTTAATAAAAAATACCGTATGTTGGATGACACCGTTAGCGAATCGGAATTTATTGTACGGTATAACCCAGAGCATGAAGATTGA

BE/24/2011 ATGCGTGGCATTTGGAAGCCAGGAAGTAACCTAATATACAGA---TTTATATGCGTACTTACTATCTGGACTCCTTTGTACAGAAGTAAATGTGAGAACACTACTAATGC---------------TTCATCCACCGCTGAATCTACAAGCATTATCACTACTACATTGACTACAGATCTTTCATCTACATATCAAATATCATCTGCATCAACTTCAACAC------------CTATTTCTAACACAG------CTACCATAAGCACTTTCATCACTTCCAATCCAAATACA---TCGTATGTTTCCACCTCTTACAATACAT------------------------------------CGATAATTTTAAGCACATCAACGGAAACTAACGTTGGCTCTACGGAAATAAACGTTACTTCCAT---------GAATACTTCAGCAACAAGCAACGTCACGTCAAATGT------------------------CACCATAACTCCAACCGTAGTAACAAGTACAACTATCAGTAACATATCATCTG---TAAGTTATGAAATGTTCAA---TA------CGACAATTAATAATACACAAGACTGTG---ATGAATGTCAACCAATAAAAGTAAATAAAACAGATATCGAAGCAGAAGAATGGACCAATGTTACCATACAAAGCAATTTCACGATACCCCATTGTCACAAGGTGGTTTGGATGCGACAATATAATCTAACCACACATGGAGATTATTTCCCAATACGATATAAACGTCC-------------TGTTAAAGGTGCCTTGTATTCACGT--GAAATATGCGGCCATACATACACACACAATCTTCTACACTCATATGACTTATGCATCTCGT---GCGACAACGGAACACTACATCTCTACGGTGTAAACACAACTCATTCAGGTAGATATACTGCACGTTGTCATATCTATGAACATAATGA---TCACGGCACACATGAGGACAAAAATTTTAATCTGATTATATATCCTAGAAATAATACTAACAATACCAACGGTATCTGGATATGTC-----------CACGAC----------CTACAAAAGACGAAGCCCAAGAAAACAACCAGAGTGAAGAAAAGC---ATCTAACAACGACGGACAATTCTGTTTCCCATAAACGAAACCATTACCCAAGAACTTCCCACCGCAGTGCCTGGACTGTTACGTTGCTTTGTGTCGCCTGTATACTCCTATTTTTTTTTCGACGTCTTTTTAATAAAAAATACCGTATGTTGGATGACACCGTTAGCGAATCGGAATTTATTGTACGGTATAACCCAGAGCATGAAGATTGA

BE/14/2011 ATGCGTGGCATTTGGAAGCCAGGAAGTAACCTAATATACAGA---TTTATATGCGTACTTACTATCTGGACTCCTTTGTACAGAAGTAAATGTGAGAACACTACTAATGC---------------TTCATCCACCGCTGAATCTACAAGCATTATCACTACTACATTGACTACAGATCTTTCATCTACATATCAAATATCATCTGCATCAACTTCAACAC------------CTATTTCTAACACAG------CTACCATAAGCACTTTCATCACTTCCAATCCAAATACA---TCGTATGTTTCCACCTCTTACAATACAT------------------------------------CGATAATTTTAAGCACATCAACGGAAACTAACGTTGGCTCTACGGAAATAAACGTTACTTCCAT---------GAATACTTCAGCAACAAGCAACGTCACGTCAAATGT------------------------CACCATAACTCCAACCGTAGTAACAAGTACAACTATCAGTAACATATCATCTG---TAAGTTATGAAATGTTCAA---TA------CGACAATTAATAATACACAAGACTGTG---ATGAATGTCAACCAATAAAAGTAAATAAAACAGATATCGAAGCAGAAGAATGGACCAATGTTACCATACAAAGCAATTTCACGATACCCCATTGTCACAAGGTGGTTTGGATGCGACAATATAATCTAACCACACATGGAGATTATTTCCCAATACGATATAAACGTCC-------------TGTTAAAGGTGCCTTGTATTCACGT--GAAATATGCGGCCATACATACACACACAATCTTCTACACTCATATGACTTATGCATCTCGT---GCGACAACGGAACACTACATCTCTACGGTGTAAACACAACTCATTCAGGTAGATATACTGCACGTTGTCATATCTATGAACATAATGA---TCACGGCACACATGAGGACAAAAATTTTAATCTGATTATATATCCTAGAAATAATACTAACAATACCAACGGTATCTGGATATGTC-----------CACGAC----------CTACAAAAGACGAAGCCCAAGAAAACAACCAGAGTGAAGAAAAGC---ATCTAACAACGACGGACAATTCTGTTTCCCATAAACGAAACCATTACCCAAGAACTTCCCACCGCAGTGCCTGGACTGTTACGTTGCTTTGTGTCGCCTGTATACTCCTATTTTTTTTTCGACGTCTTTTTAATAAAAAATACCGTATGTTGGATGACACCGTTAGCGAATCGGAATTTATTGTACGGTATAACCCAGAGCATGAAGATTGA

BE/37/2011 ATGCGTGGCATTTGGAAGCCAGGAAGTAACCTAATATACAGA---TTTATATGCGTACTTACTATCTGGACTCCTTTGTACAGAAGTAAATGTGAGAACACTACTAATGC---------------TTCATCCACCGCTGAATCTACAAGCATTATCACTACTACATTGACTACAGATCTTTCATCTACATATCAAATATCATCTGCATCAACTTCAACAC------------CTATTTCTAACACAG------CTACCATAAGCACTTTCATCACTTCCAATCCAAATACA---TCGTATGTTTCCACCTCTTACAATACAT------------------------------------CGATAATTTTAAGCACATCAACGGAAACTAACGTTGGCTCTACGGAAATAAACGTTACTTCCAT---------GAATACTTCAGCAACAAGCAACGTCACGTCAAATGT------------------------CACCATAACTCCAACCGTAGTAACAAGTACAACTATCAGTAACATATCATCTG---TAAGTTATGAAATGTTCAA---TA------CGACAATTAATAATACACAAGACTGTG---ATGAATGTCAACCAATAAAAGTAAATAAAACAGATATCGAAGCAGAAGAATGGACCAATGTTACCATACAAAGCAATTTCACGATACCCCATTGTCACAAGGTGGTTTGGATGCGACAATATAATCTAACCACACATGGAGATTATTTCCCAATACGATATAAACGTCC-------------TGTTAAAGGTGCCTTGTATTCACGT--GAAATATGCGGCCATACATACACACACAATCTTCTACACTCATATGACTTATGCATCTCGT---GCGACAACGGAACACTACATCTCTACGGTGTAAACACAACTCATTCAGGTAGATATACTGCACGTTGTCATATCTATGAACATAATGA---TCACGGCACACATGAGGACAAAAATTTTAATCTGATTATATATCCTAGAAATAATACTAACAATACCAACGGTATCTGGATATGTC-----------CACGAC----------CTACAAAAGACGAAGCCCAAGAAAACAACCAGAGTGAAGAAAAGC---ATCTAACAACGACGGACAATTCTGTTTCCCATAAACGAAACCATTACCCAAGAACTTCCCACCGCAGTGCCTGGACTGTTACGTTGCTTTGTGTCGCCTGTATACTCCTATTTTTTTTTCGACGTCTTTTTAATAAAAAATACCGTATGTTGGATGACACCGTTAGCGAATCGGAATTTATTGTACGGTATAACCCAGAGCATGAAGATTGA

BE/1/2012 ATGCGTGGCATTTGGAAGCCAGGAAGTAACCTAATATACAGA---TTTATATGCGTACTTACTATCTGGACTCCTTTGTACAGAAGTAAATGTGAGAACACTACTAATGC---------------TTCATCCACCGCTGAATCTACAAGCATTATCACTACTACATTGACTACAGATCTTTCATCTACATATCAAATATCATCTGCATCAACTTCAACAC------------CTATTTCTAACACAG------CTACCATAAGCACTTTCATCACTTCCAATCCAAATACA---TCGTATGTTTCCACCTCTTACAATACAT------------------------------------CGATAATTTTAAGCACATCAACGGAAACTAACGTTGGCTCTACGGAAATAAACGTTACTTCCAT---------GAATACTTCAGCAACAAGCAACGTCACGTCAAATGT------------------------CACCATAACTCCAACCGTAGTAACAAGTACAACTATCAGTAACATATCATCTG---TAAGTTATGAAATGTTCAA---TA------CGACAATTAATAATACACAAGACTGTG---ATGAATGTCAACCAATAAAAGTAAATAAAACAGATATCGAAGCAGAAGAATGGACCAATGTTACCATACAAAGCAATTTCACGATACCCCATTGTCACAAGGTGGTTTGGATGCGACAATATAATCTAACCACACATGGAGATTATTTCCCAATACGATATAAACGTCC-------------TGTTAAAGGTGCCTTGTATTCACGT--GAAATATGCGGCCATACATACACACACAATCTTCTACACTCATATGACTTATGCATCTCGT---GCGACAACGGAACACTACATCTCTACGGTGTAAACACAACTCATTCAGGTAGATATACTGCACGTTGTCATATCTATGAACATAATGA---TCACGGCACACATGAGGACAAAAATTTTAATCTGATTATATATCCTAGAAATAATACTAACAATACCAACGGTATCTGGATATGTC-----------CACGAC----------CTACAAAAGACGAAGCCCAAGAAAACAACCAGAGTGAAGAAAAGC---ATCTAACAACGACGGACAATTCTGTTTCCCATAAACGAAACCATTACCCAAGAACTTCCCACCGCAGTGCCTGGACTGTTACGTTGCTTTGTGTCGCCTGTATACTCCTATTTTTTTTTCGACGTCTTTTTAATAAAAAATACCGTATGTTGGATGACACCGTTAGCGAATCGGAATTTATTGTACGGTATAACCCAGAGCATGAAGATTGA

BE/17/2011 ATGCGTGGCATTTGGAAGCCAGGAAGTAACCTAATATACAGA---TTTATATGCGTACTTACTATCTGGACTCCTTTGTACAGAAGTAAATGTGAGAACACTACTAATGC---------------TTCATCCACCGCTGAATCTACAAGCATTATCACTACTACATTGACTACAGATCTTTCATCTACATATCAAATATCATCTGCATCAACTTCAACAC------------CTATTTCTAACACAG------CTACCATAAGCACTTTCATCACTTCCAATCCAAATACA---TCGTATGTTTCCACCTCTTACAATACAT------------------------------------CGATAATTTTAAGCACATCAACGGAAACTAACGTTGGCTCTACGGAAATAAACGTTACTTCCAT---------GAATACTTCAGCAACAAGCAACGTCACGTCAAATGT------------------------CACCATAACTCCAACCGTAGTAACAAGTACAACTATCAGTAACATATCATCTG---TAAGTTATGAAATGTTCAA---TA------CGACAATTAATAATACACAAGACTGTG---ATGAATGTCAACCAATAAAAGTAAATAAAACAGATATCGAAGCAGAAGAATGGACCAATGTTACCATACAAAGCAATTTCACGATACCCCATTGTCACAAGGTGGTTTGGATGCGACAATATAATCTAACCACACATGGAGATTATTTCCCAATACGATATAAACGTCC-------------TGTTAAAGGTGCCTTGTATTCACGT--GAAATATGCGGCCATACATACACACACAATCTTCTACACTCATATGACTTATGCATCTCGT---GCGACAACGGAACACTACATCTCTACGGTGTAAACACAACTCATTCAGGTAGATATACTGCACGTTGTCATATCTATGAACATAATGA---TCACGGCACACATGAGGACAAAAATTTTAATCTGATTATATATCCTAGAAATAATACTAACAATACCAACGGTATCTGGATATGTC-----------CACGAC----------CTACAAAAGACGAAGCCCAAGAAAACAACCAGAGTGAAGAAAAGC---ATCTAACAACGACGGACAATTCTGTTTCCCATAAACGAAACCATTACCCAAGAACTTCCCACCGCAGTGCCTGGACTGTTACGTTGCTTTGTGTCGCCTGTATACTCCTATTTTTTTTTCGACGTCTTTTTAATAAAAAATACCGTATGTTGGATGACACCGTTAGCGAATCGGAATTTATTGTACGGTATAACCCAGAGCATGAAGATTGA

Pat_A ATGCGTGGCATTTGGAAGCCAGGAAGTAACCTAATATACAGA---TTTATATGCGTACTTACTATCTGGACTCCTTTGTACAGAAGTAAATGTGAGAACACTACTAATGC---------------TTCATCCACCGCTGAATCTACAAGCATTATCACTACTACATTGACTACAGATCTTTCATCTACATATCAAATATCATCTGCATCAACTTCAACAC------------CTATTTCTAACACAG------CTACCATAAGCACTTTCATCACTTCCAATCCAAATACA---TCGTATGTTTCCACCTCTTACAATACAT------------------------------------CGATAATTTTAAGCACATCAACGGAAACTAACGTTGGCTCTACGGAAATAAACGTTACTTCCAT---------GAATACTTCAGCAACAAGCAACGTCACGTCAAATGT------------------------CACCATAACTCCAACCGTAGTAACAAGTACAACTATCAGTAACATATCATCTG---TAAGTTATGAAATGTTCAA---TA------CGACAATTAATAATACACAAGACTGTG---ATGAATGTCAACCAATAAAAGTAAATAAAACAGATATCGAAGCAGAAGAATGGACCAATGTTACCATACAAAGCAATTTCACGATACCCCATTGTCACAAGGTGGTTTGGATGCGACAATATAATCTAACCACACATGGAGATTATTTCCCAATACGATATAAACGTCC-------------TGTTAAAGGTGCCTTGTATTCACGT--GAAATATGCGGCCATACATACACACACAATCTTCTACACTCATATGACTTATGCATCTCGT---GCGACAACGGAACACTACATCTCTACGGTGTAAACACAACTCATTCAGGTAGATATACTGCACGTTGTCATATCTATGAACATAATGA---TCACGGCACACATGAGGACAAAAATTTTAATCTGATTATATATCCTAGAAATAATACTAACAATACCAACGGTATCTGGATATGTC-----------CACGAC----------CTACAAAAGACGAAGCCCAAGAAAACAACCAGAGTGAAGAAAAGC---ATCTAACAACGACGGACAATTCTGTTTCCCATAAACGAAACCATTACCCAAGAACTTCCCACCGCAGTGCCTGGACTGTTACGTTGCTTTGTGTCGCCTGTATACTCCTATTTTTTTTTCGACGTCTTTTTAATAAAAAATACCGTATGTTGGATGACACCGTTAGCGAATCGGAATTTATTGTACGGTATAACCCAGAGCATGAAGATTGA

HANRTR1A ATGCGTGGCATTTGGAAGCCAGGAAGTAACCTAATATACAGA---TTTATATGCGTACTTACTATCTGGACTCCTTTGTACAGAAGTAAATGTGAGAACACTACTAATGC---------------TTCATCCACCGCTGAATCTACAAGCATTATCACTACTACATTGACTACAGATCTTTCATCTACATATCAAATATCATCTGCATCAACTTCAACAC------------CTATTTCTAACACAG------CTACCATAAGCACTTTCATCACTTCCAATCCAAATACA---TCGTATGTTTCCACCTCTTACAATACAT------------------------------------CGATAATTTTAAGCACATCAACGGAAACTAACGTTGGCTCTACGGAAATAAACGTTACTTCCAT---------GAATACTTCAGCAACAAGCAACGTCACGTCAAATGT------------------------CACCATAACTCCAACCGTAGTAACAAGTACAACTATCAGTAACATATCATCTG---TAAGTTATGAAATGTTCAA---TA------CGACAATTAATAATACACAAGACTGTG---ATGAATGTCAACCAATAAAAGTAAATAAAACAGATATCGAAGCAGAAGAATGGACCAATGTTACCATACAAAGCAATTTCACGATACCCCATTGTCACAAGGTGGTTTGGATGCGACAATATAATCTAACCACACATGGAGATTATTTCCCAATACGATATAAACGTCC-------------TGTTAAAGGTGCCTTGTATTCACGT--GAAATATGCGGCCATACATACACACACAATCTTCTACACTCATATGACTTATGCATCTCGT---GCGACAACGGAACACTACATCTCTACGGTGTAAACACAACTCATTCAGGTAGATATACTGCACGTTGTCATATCTATGAACATAATGA---TCACGGCACACATGAGGACAAAAATTTTAATCTGATTATATATCCTAGAAATAATACTAACAATACCAACGGTATCTGGATATGTC-----------CACGAC----------CTACAAAAGACGAAGCCCAAGAAAACAACCAGAGTGAAGAAAAGC---ATCTAACAACGACGGACAATTCTGTTTCCCATAAACGAAACCATTACCCAAGAACTTCCCACCGCAGTGCCTGGACTGTTACGTTGCTTTGTGTCGCCTGTATACTCCTATTTTTTTTTCGACGTCTTTTTAATAAAAAATACCGTATGTTGGATGACACCGTTAGCGAATCGGAATTTATTGTACGGTATAACCCAGAGCATGAAGATTGA

HANRTR5 ATGCGTGGCATTTGGAAGCCAGGAAGTAACCTAATATACAGA---TTTATATGCGTACTTACTATCTGGACTCCTTTGTACAGAAGTAAATGTGAGAACACTACTAATGC---------------TTCATCCACCGCTGAATCTACAAGCATTATCACTACTACATTGACTACAGATCTTTCATCTACATATCAAATATCATCTGCATCAACTTCAACAC------------CTATTTCTAACACAG------CTACCATAAGCACTTTCATCACTTCCAATCCAAATACA---TCGTATGTTTCCACCTCTTACAATACAT------------------------------------CGATAATTTTAAGCACATCAACGGAAACTAACGTTGGCTCTACGGAAATAAACGTTACTTCCAT---------GAATACTTCAGCAACAAGCAACGTCACGTCAAATGT------------------------CACCATAACTCCAACCGTAGTAACAAGTACAACTATCAGTAACATATCATCTG---TAAGTTATGAAATGTTCAA---TA------CGACAATTAATAATACACAAGACTGTG---ATGAATGTCAACCAATAAAAGTAAATAAAACAGATATCGAAGCAGAAGAATGGACCAATGTTACCATACAAAGCAATTTCACGATACCCCATTGTCACAAGGTGGTTTGGATGCGACAATATAATCTAACCACACATGGAGATTATTTCCCAATACGATATAAACGTCC-------------TGTTAAAGGTGCCTTGTATTCACGT--GAAATATGCGGCCATACATACACACACAATCTTCTACACTCATATGACTTATGCATCTCGT---GCGACAACGGAACACTACATCTCTACGGTGTAAACACAACTCATTCAGGTAGATATACTGCACGTTGTCATATCTATGAACATAATGA---TCACGGCACACATGAGGACAAAAATTTTAATCTGATTATATATCCTAGAAATAATACTAACAATACCAACGGTATCTGGATATGTC-----------CACGAC----------CTACAAAAGACGAAGCCCAAGAAAACAACCAGAGTGAAGAAAAGC---ATCTAACAACGACGGACAATTCTGTTTCCCATAAACGAAACCATTACCCAAGAACTTCCCACCGCAGTGCCTGGACTGTTACGTTGCTTTGTGTCGCCTGTATACTCCTATTTTTTTTTCGACGTCTTTTTAATAAAAAATACCGTATGTTGGATGACACCGTTAGCGAATCGGAATTTATTGTACGGTATAACCCAGAGCATGAAGATTGA

UKNEQAS2 ATGCGTGGCATTTGGAAGCCAGGAAGTAACCTAATATACAGA---TTTATATGCGTACTTACTATCTGGACTCCTTTGTACAGAAGTAAATGTGAGAACACTACTAATGC---------------TTCATCCACCGCTGAATCTACAAGCATTATCACTACTACATTGACTACAGATCTTTCATCTACATATCAAATATCATCTGCATCAACTTCAACAC------------CTATTTCTAACACAG------CTACCATAAGCACTTTCATCACTTCCAATCCAAATACA---TCGTATGTTTCCACCTCTTACAATACAT------------------------------------CGATAATTTTAAGCACATCAACGGAAACTAACGTTGGCTCTACGGAAATAAACGTTACTTCCAT---------GAATACTTCAGCAACAAGCAACGTCACGTCAAATGT------------------------CACCATAACTCCAACCGTAGTAACAAGTACAACTATCAGTAACATATCATCTG---TAAGTTATGAAATGTTCAA---TA------CGACAATTAATAATACACAAGACTGTG---ATGAATGTCAACCAATAAAAGTAAATAAAACAGATATCGAAGCAGAAGAATGGACCAATGTTACCATACAAAGCAATTTCACGATACCCCATTGTCACAAGGTGGTTTGGATGCGACAATATAATCTAACCACACATGGAGATTATTTCCCAATACGATATAAACGTCC-------------TGTTAAAGGTGCCTTGTATTCACGT--GAAATATGCGGCCATACATACACACACAATCTTCTACACTCATATGACTTATGCATCTCGT---GCGACAACGGAACACTACATCTCTACGGTGTAAACACAACTCATTCAGGTAGATATACTGCACGTTGTCATATCTATGAACATAATGA---TCACGGCACACATGAGGACAAAAATTTTAATCTGATTATATATCCTAGAAATAATACTAACAATACCAACGGTATCTGGATATGTC-----------CACGAC----------CTACAAAAGACGAAGCCCAAGAAAACAACCAGAGTGAAGAAAAGC---ATCTAACAACGACGGACAATTCTGTTTCCCATAAACGAAACCATTGCCCAAGAACTTCCCACCGCAGTGCCTGGACTGTTACGTTGCTTTGTGTCGCCTGTATACTCCTATTTTTTTTTCGACGTCTTTTTAATAAAAAATACCGTATGTTGGATGACACCGTTAGCGAATCGGAATTTATTGTACGGTATAACCCAGAGCATGAAGATTGA

BE/5/2011 ATGCGTGGCATTTGGAAGCCAGGAAGTAACCTAATATACAGA---TTGATATGCGTACTTACTATCTGGACTCCTTTGTACAGAAGTAAATGTGAGAACACTACTAATTT---------------TTCATCCACCGCTGAATCTACAAGCATTATCACTACTACATTGACTACAGATCTTTCATCTACATATCAAACATTATCTGCATTAACTTCAACAC------------CTATTTCTAACACAG------CTACCACAAGCACTTTCATCACTTCCAATCCAAATACA---TCGTATGCTTCCACCTCTTACAATACAT------------------------------------CGATAATTTTAAGCACATCAACGGAAATTAACGTTGGCTCTACGGAAATAAACGTTACTTTCAT---------GAATACTTCAGCAACAAGCAACGTCACGTCACATGTCACAGACATGAAAGTAACAACAGTCACCATAACTCCGACCGTAGTAACAAGTACAACTATCAGTAACATATCATCTG---TAAGTTATGAAATGTTCAA---TA------CGACAATTAATAATACACAAGACCGTG---ATAAATGCCAACTAATAAAAGTAAATAAAACAGACATCGAAGCAGAAGAATGGACCAATGTTACCATACAAAGCAATTTCACGATACCCCATTGTCACAAGGTGGTTTGGATGCGACAATATAATCTAACCACACATGGAGATTATTTCCCAATACGACATAAACGTCC-------------TGTTAAAGGTGCCTTGTATTCACGT--GAAATATGCGGCCATACATACACACACAATCTTCTACACTCATATGACTTATGCATCTCGT---GCGACAACGGAACACTACATCTCTACGGTGTAAACACAACTCATTCAGGTAGATATACTGCACGTTGTCATATTTATGAACATAATGA---TCACGGCACACATGAGGACAAAAATTTTAATCTGATTATATATCCTAGAAATAATACTAACAATACCAACGGTATCTGGATATGTC-----------CACGAC----------CTACAAAAGACGAAGCCCAAGAAAACAATCAGGGTGAAGAAAAAC---ATCTAACAACGACGGACGACTCCGTTTCCCATAAACGAAACCATTACCCAAGAACCTCACACCGCAGTGCTTGGACTGTTACGTTGCTTTGTGTCGCCTGTATACTCCTATTTTTTTTTCGACGTCTTTTTAACAAAAAATACCGTATATTGGATGACACCGTCAGCGAATCGGAATTTATTGTACGGTATAACCCAGAGCATGAAGATTGA
[truncated: 3,481,035 more chars]
