# Supplementary material for: Human Cytomegalovirus Genomes Sequenced Directly From Clinical Material: Variation, Multiple-Strain Infection, Recombination, and Gene Loss
Source: J Infect Dis. 2019 May 2;220(5):781–91. doi: 10.1093/infdis/jiz208 (PMC6667795; doi:10.1093/infdis/jiz208)
Supplement: jiz208_suppl_Supplementary_Figure_2 [file jiz208_suppl_supplementary_figure_2.docx]

**Supplementary Figure 2.** Maximum likelihood phylogeny reconstructions for the hypervariable HCMV genes used for genotyping by motif read-matching. The nucleotide sequences in **Supplementary Figure 1** were aligned using ClustalW (RL5A) or Muscle (all others) within MEGA v6.06 (<https://www.megasoftware.net>), omitting any designated as recombinants. Unrooted trees were calculated using MEGA with the Tamura 3-parameter model and invariant sites (RL5A) or five discrete gamma categories (all others), omitting gapped sites with <95% coverage. The trees were tested using the bootstrap method with 100 replicates, and visualised in FigTree v1.4.4 (<https://github.com/rambaut/figtree/releases>) with branches collapsed into genotypes (G). Combined genotypes are due to related sequences within more broadly related groups, and were due in some instances to deletions. Bootstrap values are indicated at the nodes according to the colour scale at the top left. The scale in each panel indicates substitutions per nucleotide averaged over the alignment length.

RL13

RL12

RL6

RL5A

UL74

UL120

UL146

UL139

UL73

UL11

UL9

UL1
